# Genome and Phylogenetic Analysis of Genes Involved in the Immune System of *Solea senegalensis* – Potential Applications in Aquaculture

**DOI:** 10.3389/fgene.2019.00529

**Published:** 2019-06-11

**Authors:** Aglaya García-Angulo, Manuel A. Merlo, María E. Rodríguez, Silvia Portela-Bens, Thomas Liehr, Laureana Rebordinos

**Affiliations:** ^1^Área de Genética, Facultad de Ciencias del Mar y Ambientales, Universidad de Cádiz, Cádiz, Spain; ^2^Institute of Human Genetics, Jena University Hospital, Friedrich Schiller University Jena, Jena, Germany

**Keywords:** *Solea senegalensis*, bacterial artificial chromosome, immune system, aquaculture, syntenic conservation

## Abstract

Global aquaculture production continues to increase rapidly. One of the most important species of marine fish currently cultivated in Southern Europe is *Solea senegalensis*, reaching more than 300 Tn in 2017. In the present work, 14 Bacterial Artificial Chromosome (BAC) clones containing candidate genes involved in the immune system (*b2m*, *il10*, *tlr3*, *tap1*, *tnf*α, *tlr8*, *trim25*, *lysg*, *irf5*, *hmgb2*, *calr*, *trim16*, and *mx*), were examined and compared with other species using multicolor Fluorescence *in situ* Hybridization (mFISH), massive sequencing and bioinformatic analysis to determine the genomic surroundings and syntenic chromosomal conservation of the genomic region contained in each BAC clone. The mFISH showed that the groups of genes *hmgb2-trim25-irf5-b2m*; *tlr3-lysg*; *tnfα-tap1*, and *il10-mx-trim16* were co-localized on the same chromosomes. Synteny results suggested that the studied BACs are placed in a smaller number of chromosomes in *S. senegalensis* that in other species. Phylogenetic analyses suggested that the evolutionary rate of immune system genes studied is similar among the taxa studied, given that the clustering obtained was in accordance with the accepted phylogenetic relationships among these species. This study contributes to a better understanding of the structure and function of the immune system of the Senegalese sole, which is essential for the development of new technologies and products to improve fish health and productivity.

## Introduction

Global aquaculture production continues to increase rapidly, yet only a small proportion of the aquatic animals and plants being produced are obtained from managed breeding and improvement programs (MacKenzie and Jentoft, 2016). However, the accelerated growth of aquaculture has resulted in adverse effects to the natural environment and to human health. This concern is illustrated by the widespread and, in some cases, unrestricted use of prophylactic antibiotics in this industry, with the objective of preventing bacterial infections resulting from sanitary shortcomings in fish rearing. This practice has resulted in the emergence of antibiotic-resistant bacteria in aquaculture environments, the increase of antibiotic resistance in fish pathogens, the transfer of these resistance determinants to bacteria affecting land animals and to human pathogens, and alterations of the bacterial microbiome in both sediments and water column (Han et al., 2017). All of these are serious and undesirable outcomes.

A viable alternative for avoiding chemicals and preventing economic impact is the administration of immunostimulants, prebiotics and probiotics, which act to reinforce the innate immune system of the farmed fish (Nayak, 2010). A selection program is also an important tool for optimizing the immune capacity of the stocks, developing new technologies and products to improve productivity and to overcome the misuse of antibiotics. Thus, it is essential to understand in depth the structure and function of the fish immune system. However, few studies deal with the immune system in commercially important fish species.

*Solea senegalensis* is a flatfish species belonging to the Pleuronectiformes order, which comprises about 570 species. It is distributed along the northwestern coast of Africa, as far north as the southwestern coast of the Iberian Peninsula, including the Mediterranean Sea (Díaz-Ferguson et al., 2007). Commercial production of *S. senegalensis* started in the early 1980’s and this species is considered a promising candidate for the diversification of aquaculture (Chairi et al., 2010; Padrós et al., 2011). In the last 10 years, the production of *S. senegalensis* in Spain has increased from 32 to 747.15 Tn, which illustrates the rapid growth of interest in production of the species (FishStat, FAO, 2019).

Several studies have already been carried out to improve the production of the Senegalese sole. The high mortality rates at different phases of production and the high incidence of diseases, particularly pasteurellosis and flexibacteriosis, have been critical in recognizing the need advocating for better production methods for the sole (Morais et al., 2014). Comprehensive study of the genes involved in disease resistance should greatly facilitate the solution of these problems.

The elaboration of an integrated genetic map would provide complete information about the localization and structure of genes of interest. This information could be used for comparative genomics purposes, and would constitute the scientific basis for developing improvement programs. In the case of *S. senegalensis*, the mapping of its genome has been carried out in recent years, using markers such as the minor and major ribosomal genes and other repetitive sequences were first localized using FISH techniques (Cross et al., 2006; Manchado et al., 2006). The elaboration of a BAC library in *S. senegalensis* has allowed researchers to localize single copy genes (Ponce et al., 2011), and to integrate the cytogenetic map with the physical map obtained by BAC sequencing (García-Cegarra et al., 2013; Merlo et al., 2017; Portela-Bens et al., 2017). In addition, linkage maps were also created in *S. senegalensis* (Molina-Luzón et al., 2014) and in the closely related species *Solea solea* (Diopere et al., 2014). A preliminary draft genome for a *S. senegalensis* female has been published and consisted of 34176 scaffolds with a N50 of 85 kb. Furthermore, this draft genome contained 209 out of the 274 ultra-conserved core eukaryotic genes, with a completeness of 84.3% and an average number of orthologues of 1.31, considering the number of eukaryotic genes discovered into the scaffolds (Manchado et al., 2016).

In *S. senegalensis*, the gene expression of various genes related to the immune system has been examined, including hepcidin, lysozyme g-type and the TNF gene family (Salas-Leiton et al., 2010, 2012; Núñez-Díaz et al., 2016). An exhaustive expression analysis of genes relevant for the immune system was also undertaken in the closely related species *S. solea* (Ferraresso et al., 2016). However, knowledge of the gene structure, genomic characterization and localization of immune-related genes is limited. Studies of this kind have been carried out only with the g-type lysozyme (Ponce et al., 2011), myxovirus resistance protein 1, immunoglobulin superfamily member 9b, and semaphorin 7a (García-Cegarra et al., 2013).

In this work, the localization and the genomic organization of 14 BAC clones containing immune-related genes was assessed. Seven out of the 14 BAC clones contain well-known immune-related genes, such as the g-type lysozyme (*lysg*), myxovirus resistance 1 (*mx1*), toll-like receptors 3 and 8 (*tlr3* and *tlr8*), beta-2-microglobulin (*b2m*), interferon regulatory factor 5 (*irf5*) and tumor necrosis factor α (*tnf*α). Another four BAC clones were chosen for their relationship with the immune system found in the bibliography, such as antigen peptide transporter 1 (*tap1*) (Pinto et al., 2011) interleukin-10 (*il10*) (Zou et al., 2003), and two BAC clones with calreticulin (*calr*) (Wang et al., 2018). The remaining three were anonymous BAC clones that, in the sequencing and annotation process, were found to contain immune-related genes, such as tripartite motif-containing proteins 16 and 25 (*trim16* and *trim25*) and high mobility group protein B2 (*hmgb2*). The genes studied belong to both the innate and acquired immune system. The objective of this study was to carry out an analysis of micro-synteny, comparative mapping and phylogenetics between *S. senegalensis* and relevant aquaculture species. This will allow deepening the knowledge about the structure of the genome and evolutionary trends of the immune system within flatfish species. The results would facilitate future work related to quantitative trait loci (QTL) and gene expression.

## Materials and Methods

### PCR Screening of the *Solea senegalensis* BAC Library

A 4D-PCR methodology (Asakawa et al., 1997) was carried out to find and isolate clones bearing targeted gene sequences from a BAC library previously constructed in *S. senegalensis* (García-Cegarra et al., 2013). Fourteen BAC clones containing immune-related genes were isolated (Table 1). The thirteen candidate genes used to isolate BACs were lysozyme g type (*lysg*), calreticulin (*calr*), myxovirus resistance 1 (*mx1*), toll-like receptors 3, and 8 (*tlr3*, and *tlr8*), interferon regulatory factor 5 (*irf5*), beta-2 microglobulin (*b2m*), antigen peptide transporter 1 (*tap1*), interleukin-10 (*il10)*, tumor necrosis factor α (*tnf* α), tripartite motif-containing protein 25 (*trim25*), high mobility group protein B2 (*hmgb2*) and tripartite motif-containing protein 16 (*trim16*). The PCR conditions were the same as those described in García-Cegarra et al. (2013). BACs are named after the name of the harboring candidate gene.

**Table 1:** BAC clones analyzed and gene annotation.

Name of
BAC	Genes annotated	References
38N10	*b2m, b2ml, tbim4, irak3, cdnf, hspa14, prl2, net1, asb13a, gdi2*	This work
42P4	*il19, il10, mapkapk2a, dirk3, rassf5, ikbke, srgap2, fam72a*	This work
29D4	*sorbs2a, tlr3, cyp4v2, mtnr1aa*	This work
53D20	*hes2, hes5, notch1, tap1, brd2, hla-drb1, vmo1*	This work
71N11	*pycard, scn1b, tnfα, sh3bp5la, mgat1, rnf183, dhx16, ppt2, fli1b, etv2, dnajc28*	This work
30J4	*pcdh8, ednrb, cog3, mid1, arhgap6, frmpd4, tlr7, tlr8, tyb12, egfl6, nlrc3, gcgr, wdr90, rhot2, h1f0, rhbdl1, wdr24, anks3, c8orf33, h3, gper1*	Merlo et al., 2017
60P19	*supt16h, mrc1, fam234, trim25, mrpl48, pea15, casq1b, c1q, btr12*	This work
4N21	*mcoln1, pgrp2, lysg*	Ponce et al., 2011
52G10	*mical3, mical3, lta4h, arf4, gcc1, dennd6, atp6v1f, irf5, tpno3, opn1sw1, calu, dgki, creb312, ssbp, lamb1*	This work
4D15	*ppme1, rnf150, inpp4b, hmgb2, dlg4*	This work
5K5, 10L10	*rx2, calr, eps15l1, klf2, ap1m1, tpm4, rab8a, cib3, slc1a3*	Merlo et al., 2017; García-Angulo et al., 2018
31C1	*rbpjl, trim16, matn4, or51d1, or52r1, kens*	This work
12K16	*mx*	García-Cegarra et al., 2013


*The immune-related genes are underlined.*

### BAC Clone Sequencing and Annotation

Positive BAC clones were isolated using the Large Construct Kit (Qiagen, Hilden, Germany), and sent for sequencing using the Illumina sequencing platform (Illumina, San Diego, CA, United States). The sequences were generated on the Miseq equipment, with a configuration of 300 cycles of paired end reads (Lifesequencing S.L., Valencia). The reads were *de novo* assembled using SPAdes software version 3.11.1. The functional and structural annotation of the gene sequences identified in each BAC clone was carried out in a semi-automated process. Proteins and expressed sequence tags (ESTs) from *S. senegalensis* and related species were compared. The homologous sequences obtained were used to obtain the best predictions for gene annotation. Finally, all the information available was used to create plausible models and, when possible, functional information was added. Using the Apollo genome editor (Lewis et al., 2002), Signal map software (Roche Applied Science, Penzberg, Germany), and Geneious R11 (Kearse et al., 2012), the results were individually completed and adjusted in the final editing process of the annotation. All BAC clones have been deposited in the GenBank database under the accession numbers AC278047 to AC278120. The structure of some of the genes was compared with those of seven other representative fish species, i.e., *Danio rerio* (zebrafish), *Oreochromis niloticus* (tilapia), *Gasterosteus aculeatus* (stickleback), *Seriola dumerili* (greater amberjack), *Seriola lalandi dorsalis* (yellowtail amberjack), *Scophthalmus maximus* (turbot), and *Cynoglossus semilaevis* (tongue sole).

Cross-species genome comparisons were carried out at two levels. At the first level, a micro-synteny study was performed using the ENSEMBL database and the NCBI platform. The order of the contigs within each BAC clone of *S. senegalensis* was estimated using the information provided by these programs. The seven species used for this comparison were the same as those listed above. All BAC clones were analyzed with the exception of the *mx* BAC clone, where only one gene was found, and the *trim16* BAC clone. In the schematic figures each gene is represented by a different color; white color indicates a gene that is different from that found in the Senegalese sole.

At the second level of comparison, a synteny analysis was performed using the CIRCOS software (Krzywinski et al., 2009). The five species used in this analysis are *C. semilaevis, S. maximus, O. niloticus, G*. *aculeatus* and *D. rerio.* The flatfish *Paralichthys olivaceus* has not been included because the genome assembly is not at chromosome level. These sequences are available in the ENSEMBL database and the NCBI platform. In the case of *S. dumerilii* and *S. lalandi dorsalis*, the synteny analysis could not be done because the complete genomes were not available in these databases. In the figures the locations of the genes that make up the BAC clones of *S. senegalensis* were compared with the location they presented in the other species studied, so that the relationship between the chromosomes of both species appears in the figures. Each BAC clone was represented by a different color so that co-localizations and reorganizations of genes could be better observed.

### Cytogenetic Mapping

Chromosome preparations were obtained according to García-Angulo et al. (2018). To prepare FISH probes, BAC clones were grown on LB containing chloramphenicol, at 37°C, overnight. BAC-DNA was extracted using the BACMAX^TM^ DNA purification kit (Epicenter Biotechnologies, Madison, United States), following the manufacturer’s instructions. Insert presence was evaluated by digestion with *Eco*RI and agarose gel electrophoresis (0.8%). Probes were amplified by DOP-PCR and then labeled by a conventional PCR using four different fluorochromes, i.e., Texas Red (Life Technologies, Carlsbad, California, United States), Spectrum Orange, Fluorescein isothiocyanate (FITC) (Abbott Molecular/ENZO, Illinois, United States), and diethylaminocoumarin (DEAC) (Vysis, Downers Grove, United States), using the protocol described in Liehr (2009).

Chromosome preparations were pre-treated with pepsin solution at 37°C and fixed with paraformaldehyde solution. Finally, preparations were dehydrated with ethanol in a concentration series of 70%, 90%, and 100%, and air-dried. Hybridization and post-hybridization treatment was according to Portela-Bens et al. (2017).

Slides were visualized with a fluorescence microscope (Olympus BX51 and/or Zeiss Axioplan using software of MetaSystems, Altlussheim, Germany) equipped with a digital CCD camera (Olympus DP70) to capture the images.

### Phylogenetic Analysis

Before concatenation, the sections with the highest homogeneity in each gene were taken and the substitution saturation degree was also examined in each gene using saturation plots with transitions (s) and transversions (v) implemented in the DAMBE6 software (Xia, 2017). The distance model used was GTR. Saturation is inferred when the index of substitution saturation (*I_SS_*) is either larger or not significantly smaller than the critical value (*I_SS.C_*). Finally, for phylogenetic analysis were chosen those genes representatives of different immune pathways and present in a wide number of species, in addition to do not present significative substitution saturation. Under these requirements, up to five immune-system genes (*tlr3, tlr8, nlrc3, calr, ikbke*) were concatenated to perform the phylogenetic analysis. Thirty-four species were included to generate the phylogenetic tree; twenty-two were fish species, including the target species *S. senegalensis*; ten mammal species, one reptile species and, additionally, *Latimeria chalumnae* was included as an outgroup to root the tree. The sequences were aligned using the MAFFT program (Katoh and Toh, 2008) following an iterative method of 100 iterations. The final alignment consisted in a total of 7806 positions, in which 2354 were for *tlr3*, 1725 for *tlr8*, 1772 for *nlrc3*, 1284 for *calr* and 671 for *ikbke*. The PhyML 3.0 program (Guindon et al., 2010) was used to determine the best-fit phylogenetic model and then to run the model. The resulting best-fit model was the Generalized Time-Reversible (GTR) model (Tavaré, 1986), considering the proportion of invariable sites (+I) and gamma distribution (+G). The statistic used for model selection was the Akaike information criterion (AIC), the value of which was 255435.93458, and the -LnL was -127384.510441. Branch support was tested by the fast likelihood-based method using aLRT SH-like (Anisimova et al., 2011). Finally, the tree was edited in the MEGA7 program (Kumar et al., 2016).

## Results

### BAC Clone Sequencing and Annotation

Of the 14 BAC clones analyzed, nine BAC clones were sequenced with a total of 80 genes annotated, and the other five BAC clones had been sequenced previously (Ponce et al., 2011; García-Cegarra et al., 2013; Merlo et al., 2017; García-Angulo et al., 2018). In total, 109 genes were annotated and 24 of the 109 genes (22.01%) were found to be related to the immune system (Table 1).

### Cytogenetic Mapping

**FIGURE 1:**
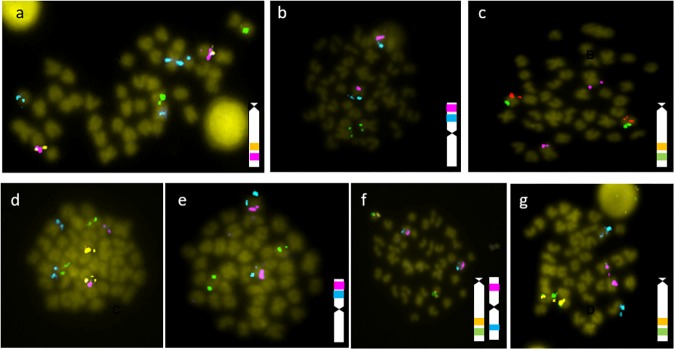
Results of mFISH of the BACs isolated with the following candidate genes: **(a)** *tlr8* (blue), *lysg* (green), *tap1* (pink), *tnf*α (orange); **(b)** *irf5* (blue), *calr* (green), *b2m* (red); **(c)** *trim 16* (green), *mx* (orange), *calr* (pink); **(d)** *irf5* (blue), *tlr3* (green); *tnf*α (orange), *mx* (pink); **(e)** *hmgb2* (blue), *lysg* (green), *b2m* (pink); **(f)** *trim25* (blue), *tnf*α (green), *tap1* (orange), *b2m* (pink); **(g)** *il10* (blue), *tnf*α (green), *tap1* (orange), *b2m* (pink). In those cases where two or more probes co-localize in one chromosome, a diagrammatic representation is included.

**FIGURE 2:**
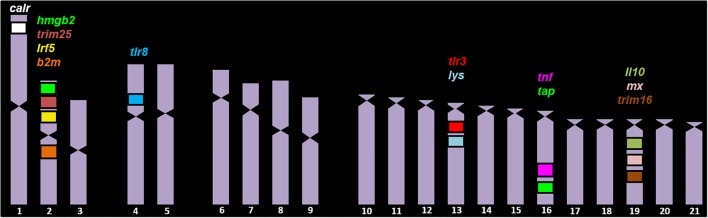
BAC clones analyzed in the chromosomes of *S. senegalensis.* On chromosome 1 the BAC clone *calr* was localized; on chromosome 2 there were four BAC clones, with the genes *b2ml*, *b2m*, *irak3*, *trim25, hmgb2*, and *irf5*. On chromosomes 4 and 12 the BAC clone 30J4 was localized, with the genes *tlr8, tlr7, and nlrc3*. The BAC clones 4N21 and 29D4, with the *lysg* and *tlr3* candidate genes, respectively, were found on chromosome 13. On chromosome 16, the BAC clones 53D20 and 71N11 were localized, with the genes *tap*, *hla-drb1*, *tnf*α and *pycard*. Lastly on chromosome 19 the BAC clones 42P4, 12K6 and 31C1 were localized, with the genes *il10, il19, mx, trim16, ikbke*.

Using mFISH, the 14 BAC clones were localized on six different chromosome pairs. (Figure 1, 2). BAC clones *hmgb2, b2m, irf5*, and *trim25* co-localized in a metacentric chromosome pair. The BAC clones *tlr3* and *lysg* co-localized in one acrocentric chromosome pair. The BAC clones *tap 1* and *tnf*α co-localized in a second acrocentric chromosome pair; and, lastly, the BAC clones *il10, mx* and *trim16* co-localized in a third acrocentric chromosome pair. Conversely, the BAC clone *calr* showed a signal on the largest metacentric chromosome, different from that in which the genes *hmgb2, b2m, irf5*, and *trim25* were co-located. The BAC clone *tlr8* showed a signal in two different chromosomes pairs, one stronger signal in a submetacentric pair and the other weaker signal in an acrocentric chromosome pair. Only the most intense signal was considered.

### Comparative Mapping

**FIGURE 3:**
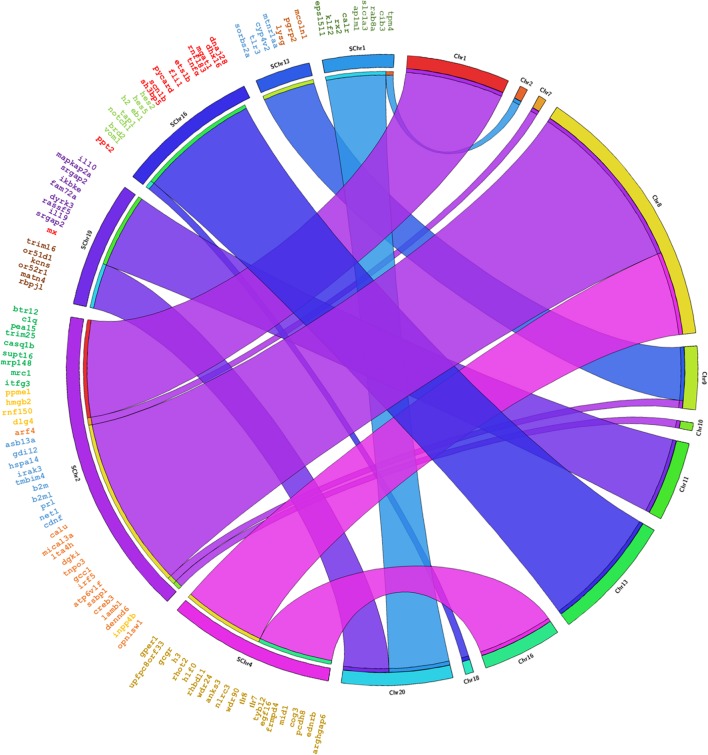
Circos analysis in the species *C. semilaevis.* On the left side the distribution of the BAC clones of Senegalese sole distributed in chromosomes can be observed. Each BAC clone is represented in a different color. The genes found by annotation are indicated within each BAC clone and their corresponding localization in the *C. semilaevis* chromosomes is denoted by crossing lines. The BAC clones analyzed are given in Table 1.

**FIGURE 4:**
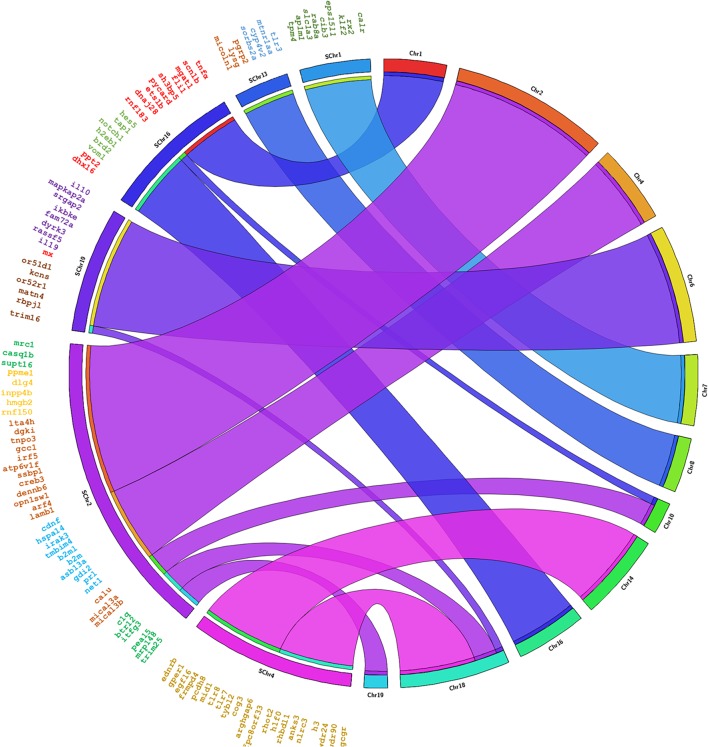
Circos analysis in the species *S. maximus.* On the left side the distribution of the BAC clones of Senegalese sole distributed in chromosomes can be observed. Each BAC clone is represented in a different color. The genes found by annotation are indicated within each BAC clone and their corresponding localization in the *S. maximus* chromosomes are denoted by crossing lines. The BAC clones analyzed are given in Table 1.

The genes annotated in Senegalese sole were found in eleven chromosomes in *C. semilaevis*, *S. maximus* and *O. niloticus*, in nineteen chromosomes in *D. rerio*, and in eleven and five scaffolds in *G. aculeatus* (Figure 3, 4 and Supplementary Figures S1–S3). The closest species was *C. semilaevis*: 96.36% of the genes of *S. senegalensis* were found in seven chromosomes of *C. semilaevis* (Figure 3). Moreover, *D. rerio* was the species with the largest number of rearrangements (Supplementary Figure S1).

Considering the comparison with *C. semilaevis*, large clusters of genes were conserved with *S. senegalensis.* Hence, chromosome 2 of Senegalese sole seems to correspond mainly with chromosomes 1 and 8 of *C. semilaevis*; chromosome 4 with chromosomes 8 and 16; and chromosome 19 with chromosomes 11 and 20 (Figure 3). *S. maximus* also presented large conserved regions with respect to *S. senegalensis*; chromosomes 1, 13 and 19 of Senegalese sole seem to correspond to chromosomes 7, 8 and 6 of *S. maximus*, respectively (Figure 4). The comparison with *O. niloticus, G. aculeatus* and *D. rerio* showed more gene rearrangements (Supplementary Figures S1–S3).

Several gene co-localizations observed in *S. senegalensis* also appeared in other species, as the cases of *il10-mx-trim16* in *S. maximus* and *O. niloticus*, *tlr7-tlr8* in all species, *tlr3-lysg* in *C. semilaevis*, *S. maximus* and *G. aculeatus*, *tnfα-tap1* in *C. semilaevis, O. niloticus* and *G. aculeatus*, *trim25-hmgb2* in *C. semilaevis* and *G. aculeatus*, *b2m-irf5* in *C. semilaevis* and *D. rerio*, *b2ml-irf5* in *O. niloticus*, or *hmgb2-irf5* in *S. maximus*.

### Micro-Synteny

**FIGURE 5:**
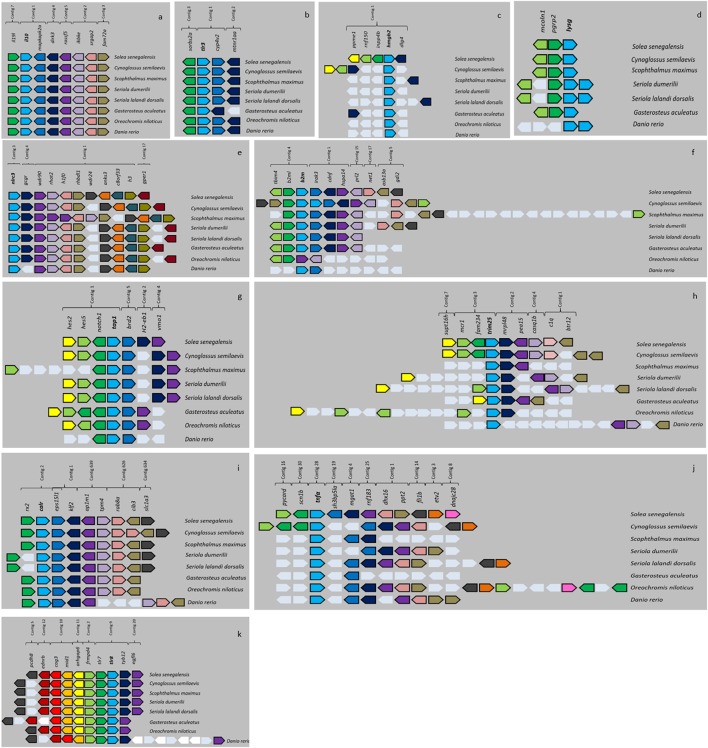
Micro-synteny of 11 BAC clones. Genes of the species *C. semilaevis*, *S. maximus, S. lalandi dorsalis, S. dumerili, G. aculeatus, O. niloticus, and D. rerio* are represented in different colors. White color indicates non-synteny genes. **(a)** BAC clone *il10*; **(b)** BAC clone *tlr3*; **(c)** BAC clone *hmgb2*; **(d)** BAC clone *lysg*; **(e)** BAC clone *nlrc3*; **(f)** BAC clone *b2m*; **(g)** BAC clone *tap1*; **(h)** BAC clone *trim25*; **(i)** BAC clone *calr*; **(j)** BAC clone *tnf alfa*; **(k)** BAC clone *tlr8.*

The micro-synteny analysis showed that many candidate genes have conserved genomic surroundings, and that, among the genomic regions analyzed, *C. semilaevis* is the species with greatest homology. The region surrounding genes *il10*, *tlr3, tlr8, nlrc3, and calr* were highly conserved in all species (Figure 5a,b,k,e,i). The micro-synteny of BAC clone *b2m* showed that gene *b2m* presented one paralog gene (*b2ml*) in all the species analyzed apart from *D. rerio.* Curiously, in *O. niloticus* the paralog gene was present but not the candidate gene (Figure 5f). The most gene rearrangements within the same genomic structure were observed in the micro-synteny of BAC clone *hmgb2* (Figure 5c).

In some of the BAC clones analyzed, other genes whose function is involved in the immune system were also found. In the BAC clone *il10*, the genes *il19* and *ikbke* appeared in the same region (Figure 5a). In the BAC clone *tap1*, the gene *hla-drb1* was found (Figure 5g). In the BAC clone *tnf*α, the gene *pycard* was observed (Figure 5j). In the BAC clone *tlr8*, the genes *tlr7* and *nlrc3* appeared (Figure 5e,k). In the BAC clone *lysg*, the gene *pgrp2* was found (Figure 5d). In the BAC clone *b2m*, the genes *b2m*, *b2ml* and *irak3* appeared (Figure 5f). Lastly, in the BAC clone *trim25*, genes *c1q* and *btr12* were found (Figure 5h). These genomic architectures were not found in all species: only the group of genes *il10-il19-ikbke* (Figure 5a) and the tandem *tlr8-tlr7* were preserved among all the species analyzed (Figure 5k).

### Phylogenetic Analysis

**FIGURE 6:**
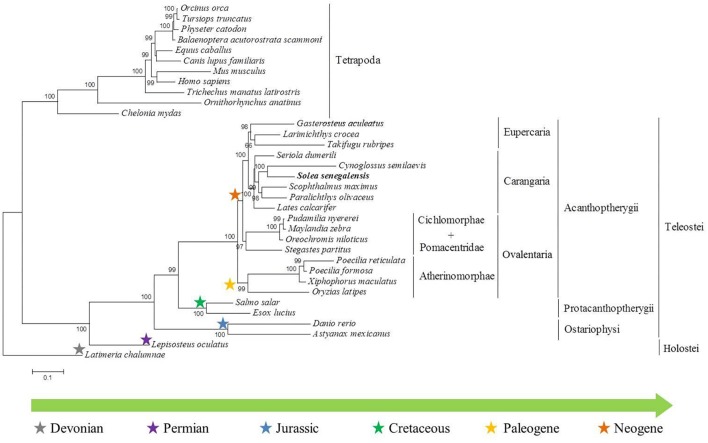
Phylogenetic tree constructed from five immune-system genes (*tlr3, tlr8, nlrc3, calr, ikbke*) of thirty-four different species.

To carry out the phylogenetic analysis, up to five candidate genes were concatenated (Supplementary Data Sheet S1). The resulting alignment had 7806 positions, where 5808 and 4975 were variable and parsimony-informative, respectively. The nucleotide frequencies were similar: f(A) = 0.26617; f(C) = 0.27001; f(G) = 0.24585; f(T) = 0.21796; and GTR relative rate parameters were A < – > C 1.30911; A < – > G 3.60083; A < – > T 1.25710; C < – > G 1.05237; C < – > T 4.73877; G < – > T 1.00000, with the proportion of invariable sites at 0.191. Results of substitution saturation tests (Xia et al., 2003) for each gene and for the concatenated alignment did not show any significant saturation, since *I_SS_* indices were lower than *I_SS.C_* values in all cases (Supplementary Data Sheet S2). The phylogenetic tree showed a good resolution and robust branch support. The phylogeny clearly separated two main clusters: ray-finned fishes and tetrapods (Figure 6). The cluster of tetrapods was divided into mammals and reptiles, and the cluster of ray-finned fishes was divided into Holostei and Teleostei. Within the group of teleosts, *S. senegalensis* appeared included in a subgroup together with the species *C. semilaevis, P. olivaceus, S. maximus, Lates calcarifer* and *S. dumerili.* All these species belong to the Carangaria group.

## Discussion

### Annotation of Immune-Related Genes

From the BAC library of *S. senegalensis* 16 genes related to the immune system have been obtained. These genes, together with those already annotated, make a total of 24 relevant genes (Table 1). The use of BAC libraries has proven to be helpful for characterizing the genome in different species, including the fish species *Larimichthys crocea* (Ao et al., 2015), *S. maximus* (Taboada et al., 2014), *Ictalurus punctatus* (Xu et al., 2007), *Oncorhynchus mykiss* (Palti et al., 2009), and bivalve species (Zhang et al., 2011; Cross et al., 2018).

Seventeen of 24 of the annotated genes related to the immune system are part of the NF-κB signaling pathway or JAK-STAT signaling pathway, two routes for the immune response. Up to twelve genes have been annotated for the first pathway, including the genes *tlr*, *nlrc3*, *lysg*, *pgrp2*, *il*, *tnf*α, *irak3*, *ikbke*, and *pycard*.

Toll-like receptors (*tlr*) are a class of pattern recognition receptors (PRR) and their function is to recognize microbial pathogens. In fish species, the *tlr* genes exhibit distinctive features and large diversity; these differences are probably derived from the diverse evolutionary history of this group and the distinctive environments that they occupy (Palti, 2011). This feature makes this kind of genes good candidates for immunity-response improvement of the aquaculture stocks. The *tlr8* gene was found to be composed by only one exon in *S. senegalensis*, as also occurs in *C. semilaevis* and *G. aculeatus*. However, in *S. maximus*, *S. dumerili* and *D. rerio*, *tlr8* is composed by two exons and one intron, which could indicate an intron losing process in different lineages during the teleost species evolution. Another class of PRR has been annotated within the same BAC clone as that of genes *tlr7* and *tlr8*, the gene *nlrc3*. Whereas TLR proteins are extracellular PRRs that recognize extracellular PAMPs, NLR proteins are intracellular PRRs that recognize intracellular PAMPs (Sha et al., 2009). Two additional PRRs were annotated in other BAC clone, the genes *lysg* and *pgrp2*. Lysozyme is a conserved molecule in teleosts (Sun et al., 2006) and is an important enzyme of the innate immunity response to bacterial infection. Many factors, such as stress and infection, sexual maturity, nutrition, toxic substances, and others, have been studied in relation to the activity levels of lysozyme in fish (Saurabh and Sahoo, 2008). The PGRP2 protein, as lysozyme, also recognizes and hydrolyzes the peptidoglycan layer (Choi et al., 2019).

The remaining genes of the NF-κB signaling pathway do not participate as PRRs. Instead, the function is downstream of the pathway. The genes *il10* and *il19* encode for two members of the IL-10 cytokines family, which includes IL-10, IL-19, IL-20, IL-22, IL-24, and IL-26 (Lutfalla et al., 2003). The IL-10 cytokine plays an important role as an anti-inflammatory agent in the innate and adaptive immune system, and IL-19 is a pro-inflammatory cytokine of the innate immune system (Hofmann et al., 2012). The *il19* gene of *S. senegalensis* showed a conserved structure across different teleost species, since it is structured in 5 exons and 4 introns; the exception is *G. aculeatus*, which shows an additional exon. *tnf*α, as well as *il10* and *il19*, is another cytokine that is secreted by activated immune-related cells upon induction by various pathogens - parasitic, bacterial and viral (Salazar-Mather and Hokeness, 2006). The *irak3* gene encodes for an inteleukin-1 associated-receptor kinase, which, in the orange-spotted grouper (*Epinephelus coioides*) has been proved to induce the NF-κB activation through TLR signaling (Li et al., 2018). It has been reported in humans that *ikbke* and *pycard* genes act as positive and negative regulators, respectively, of the NF-κB activation. In addition, *ikbke* also participates in the JAK-STAT signaling pathway (Sarkar et al., 2006; Liu et al., 2019).

Aside from *ikbke*, another five annotated genes participate in the JAK-STAT signaling pathway for the immunity response. Interferons (IFN) are another type of cytokine that are involved in key aspects of the host defense mechanisms (Reboul et al., 1999). Interferon regulatory factors (IRF) were originally identified as transcription factors participating in the regulation of interferon expression (Mamane et al., 1999). It is known that, in *C. semilaevis*, the *irf5* gene may play a role in the immune defense, primarily against intracellular pathogens (Zhang et al., 2015). The *irf5* gene structure was conserved across representative species of the Pleuronectiformes order and in *O. niloticus* and *G. aculeatus*, composed by 8 exons and 7 introns. Non-etheless, the two representative species of the *Seriola* genus showed a derived structure composed by 6 exons and 5 introns, but more analyses needs to be done in order to ascertain if this structure is plesiomorphic within the Carangidae family. *D. rerio* also showed a derived structure composed by 9 exons and 8 introns, which could be representative of such ancient lineage of teleosts. *Trim16*, *trim25* and *btr12* genes present orthologs in mammals, but *btr* genes come from the *trim39* gene of mammals. Both *trim16* and *trim25* are considered the genes from which the so-called *fintrims*, specific to teleosts, diverged (van der Aa et al., 2009). These three genes belong to the class IV subgroup of TRIM proteins, which are involved in antiviral immunity of the IFN signaling cascade (Boudinot et al., 2011). Two different types of Mx were observed in the European seabass (*Dicentrarchus labrax*), and both showed antiviral activity, but with different intensity and spatial and temporal patterns (Novel et al., 2013).

Calreticulin is a calcium-binding protein with an important role in the assembly and expression of Major Histocompatibility Complex (MHC) class I molecules (Raghavan et al., 2013). Two calreticulin types that may function as a PRR have been described in the flatfish *C. semilaevis*, thus demonstrating the antiviral and antibacterial activity of that molecule (Wang et al., 2018).

Regarding the MHC molecules, four additional genes have been annotated. MHC molecules are members of the immunoglobulin superfamily that present pathogen peptides of infected cells and thus initiate the generation of adaptive immunity to pathogens (Zhu et al., 2013). The *b2m* gene codifies for the beta subunit of the MHC class I and different paralog copies have been observed in several fish species (Kondo et al., 2010; Sun et al., 2015). Two types of *b2m* genes have been found in *S. senegalensis* and adjacent to each other. This can be observed in genome databases of fish species, in which the two adjacent copies are annotated as *b2m* and *b2ml*. Both genes presented different gene structures in *S. senegalensis*, since in *b2m* two exons and one intron were identified, and in *b2ml* 3 exons and 2 introns. The structure of *b2ml* was conserved across the other representative teleost species, but not the structure of the *b2m* gene, which is composed by 3 exons and 2 exons in those species. An expression analysis would be able to conclude definitively if this gene is undergoing a pseudogenization process. The *tap1* gene plays an essential role in the antigen presentation MHC class I pathway, transporting peptides from cytosol to the lumen of the endoplasmic reticulum (ER), where the peptides are loaded to MHC class I (Pinto et al., 2011). In the same BAC clone where the *tap1* gene is located, another MHC-related gene, *hla-drb1* was found, which encodes for the beta subunit of the MHC class II. In primates five different families of MHC class II genes, including the DR family, have been described (Satta et al., 1996).

The *c1q* gene belongs to the C1 component of the complement system pathway, and binds immunoglobulins attached to pathogen surfaces; it subsequently activates a complement cascade that culminates in the elimination of the infectious agents (Chen et al., 2018).

The *hmgb2* gene belongs to a gene family that encodes non-histone chromosomal proteins, and the HMGB2 protein has been demonstrated to display an antibacterial activity in fish due to its ability to bind pathogen DNA (Wang et al., 2019). The *hmgb2* gene has 4 exons and 3 introns in *S. senegalensis*, a structure that is also observed in the other representative teleost species.

### Structural Genomics of the Immune System

The 24 annotated genes related to the immune system are distributed in 28.571% of the chromosome complement of *S. senegalensis*. This was the lowest value in comparison with those of *C. semilaevis* (33.333%), *O. niloticus* (40.909%), *S. maximus* (45.455%), *G. aculeatus* (47.619%), and *D. rerio* (52%). These data clearly show a grouping tendency of immune system genes in the two Soleoidei species, and in *S. senegalensis* in particular. Such grouping could be a consequence of the reduction and compaction trend observed in the Pleuronectiformes evolution (Cerdá and Manchado, 2013). Moreover, more than 50% of the genes studied fell into two chromosome pairs, which could reflect a certain degree of chromosome specialization in order to facilitate the immune response of the organism. Altogether, non-random proximity patterns could be formed in a way that provides functional advantages in the genomic architecture (Cremer and Cremer, 2010). A study carried out in 2008 indicated that the selection of groups of genes of the immune system could have been an important factor that affected the reorganization of the vertebrate genome (Makino and McLysaght, 2008). However, further analysis including new immune-related genes will be required to prove this hypothesis.

The hybridizations agree with the results obtained for the BAC clones *tlr8, calr, lysg* and *mx* that had already been located (Ponce et al., 2011; García-Cegarra et al., 2013; Merlo et al., 2017; García-Angulo et al., 2018). In our study, candidate genes *tlr3* and *tlr8* were found in different chromosomes. This result has also been observed in all the species studied and in other Pleuronectiformes species such as *P. olivaceus* (Hwang et al., 2011). The *tlr7* and *tlr8* locus is highly conserved in vertebrates and these genes are located together in the chromosomes of mammals, birds and fishes (Rauta et al., 2014).

The gene *nlrc3* was located within the BAC clone *tlr8*, but, in all the species analyzed, the gene *nlrc3* was found in a chromosome different from the genes *tlr7* and *tlr8*. However, in Tetraodontiformes species (*Tetraodon nigroviridis* and *Takifugu rubripes*) the genes *tlr7* and *nlrc3* were in the same chromosome, which may be due to the compact structure of the genome in the Tetraodontiformes species, since these species have genomes that are among the most compact known in vertebrates (Jaillon et al., 2004). As mentioned before, this same trend could have taken place in the Pleuronectiformes evolution.

As can be deduced from the fish genome databases, the gene *tlr3* is linked with genes *lysg* and *pgrp2*, and this linkage is conserved throughout many bony fishes, thus representing a genomic cluster that evolves together. As discussed before, the three genes share similar functions, so the linkage could represent an advantage for a more effective immune response. It has been postulated that a conserved group of genes could indicate a functional cluster (Overbeek et al., 1999). The *calreticulin* BAC clone appears as a region strongly conserved in all the species studied at the level of macro-synteny and micro-synteny, so it could represent another functional cluster. This same situation has been observed with the genes belonging to BAC clone *il10*, which show a highly conserved synteny in all the species analyzed, including the *il19* gene and, in some species, the *mx* gene. The linkage between *il10* and *il19* has been described in other fish species (Lutfalla et al., 2003). Another example of conserved linkage among fish species is the *tap1-tnf*α genes. In *S. senegalensis* the *irf5* gene co-localized with the genes *b2m, trim25*, and *hmgb2* in one metacentric chromosome. Interestingly, this result was not observed in any other of the species studied, although a tendency to co-localize two-to-two was observed.

The results also show that large parts of the genomic regions tend to be conserved in the species most closely related to *S. senegalensis*, such as the Pleuronectiformes *C. semilaevis* and *S. maximus*, with *C. semilaevis* being the species that presents the most homology between the genomic regions analyzed. Regardless of the genetic distance between the species, a large conserved region of genes present in chromosome 2 of *S. senegalensis* was found in only one chromosome in the rest of the species analyzed. An exception was observed in stickleback; this is because part of the stickleback genome is assembled at the scaffold level.

The results of micro-synteny revealed a paralog of *b2m* in most of the species analyzed. It is clear that gene duplication and subsequent diversification have played a major role in the evolution of diversity in molecules of the MHC (Andersson, 1996). In studies carried out in bivalve species such as *Crassostrea gigas*, the expansion of several gene families related to defense pathways, including protein folding, oxidation and anti-oxidation, apoptosis and immune responses, has been observed (Wang et al., 2012).

### Immune System Phylogenetics

The phylogeny results clearly separated two main clusters: ray-finned fishes and tetrapods. The coelacanth was used to root the tree and, as expected, the genetic sequence analyzed is closer to the tetrapods than to the actinopterygian fish. The innate immune system is phylogenetically older than the adaptive system, and it is found, in some form, in all multicellular organisms, whereas the adaptive system is found in all vertebrates except jawless animals (Zarkadis et al., 2001). The concatenated gene sequences used in the phylogenetic analysis belong to both the innate and adaptive immune systems, so it was not possible to set an invertebrate as an outgroup species. However, several studies indicate that the coelacanth provides the ideal outgroup sequence against which tetrapod genomes can be measured (Noonan et al., 2004).

The cluster of tetrapods is divided into mammals and reptiles; the cluster of ray-finned fishes is divided into holostei and teleostei. Ray-finned fishes (Actinopterygii) diverged from the lineage of lobe-finned fishes (Sarcopterygii) about 450 million years ago (Christoffels et al., 2004). The actinopterygians, in turn, are divided into two main groups: holostei, the only representative of which in this tree is *Lepisosteus oculatus*; and teleostei in which group the other fish analyzed are included. *L. oculatus* appears Paleozoic era and the first fossil record dates from the Late Permian period. The genome of *L. oculatus* has a very low evolution rate and it was sequenced to help connect teleost biomedicine to human biology because its lineage represents the unduplicated sister group of teleosts (Anderson et al., 2012).

As these results show, within the clade of the teleosts, the phylogenetic tree is divided into three main groups. In the first group (superorder Ostariophysi), *Astyanax mexicanus* and *D. rerio* belong to the cohort Otomorpha and are separated from the cohort Euteleosteomorpha (the rest of those with radiated fins). In the second group (superorder Protacanthoptherygii), *Esox lucius* belongs to the order of Esociformes; *Salmo salar* belongs to the order of Salmoniformes. The third group (superorder Acanthoptherygii) is composed of several subgroups: Ovalentaria, Carangaria, and Eupercaria groups.

Similar clustering, with some exceptions, is observed in a phylogeny based on concatenated sequences related to sex determination and reproduction (Portela-Bens et al., 2017). However, other phylogenies made with one immune system-related sequence show unexpected fish-species groupings, like *c1q* (Zeng et al., 2015), and *hmgb2* (Wang et al., 2019). It was established that, for phylogenetics, the concatenation approach yields more accurate trees, even when the different concatenated sequences evolve with different substitution patterns (Gadagkar et al., 2005). The result presented here represents a novel phylogeny based on the concatenation of several immune-related genes of fishes. Moreover, the fish immune system has contributed significantly to a better understanding of the evolutionary history of the immune system (Rauta et al., 2012).

## Conclusion

Based on the results obtained, it can be concluded that the immune system genes studied tend to be grouped together in the genome of *S. senegalensis*, as the 24 immune-related genes annotated were located in only six chromosome pairs. The second conclusion is that *S. senegalensis*, and the Soleoidei suborder in general, show a higher degree of grouping in the immune-related genes, which could represent an evolutionary advantage. In addition, it seems that large parts of these genomic regions tend to be conserved in the species most closely related to *S. senegalensis*, particularly the Pleuronectiformes *C. semilaevis* and *S. maximus*; and that the rate of variability of the immune system genes studied is not high.

## Ethics Statement

The experimental procedures are ac-cording to the recommendation of the University of Cádiz (Spain) for the use of laboratory animals and the Guidelines of the European Union Council (86/609/EU).

## Author Contributions

AG-A and TL carried out the multiple FISH. AG-A drafted the manuscript. MR and SP-B isolated the BACs and carried out the bioinformatic analysis. MM constructed the phylogenetic tree and helped with the discussion and drafting. LR conceived and coordinated the study, participated in its design, discussed the results and corrected the manuscript. All authors read and approved the manuscript.

## Conflict of Interest Statement

The authors declare that the research was conducted in the absence of any commercial or financial relationships that could be construed as a potential conflict of interest.

## Supplementary Material

Figure S1 Circos analysis in the species *D. rerio.* On the left side the distribution of the BAC clones of Senegalese sole distributed in chromosomes can be observed. Each BAC clone is represented in a different color. The genes found by annotation are indicated within each BAC clone and their corresponding localization in the *D. rerio* chromosomes are denoted by crossing lines. The BAC clones analyzed are given in Table 1.

Figure S2 Circos analysis in the species *O. niloticus.* On the left side the distribution of the BAC clones of Senegalese sole distributed in chromosomes can be observed. Each BAC clone is represented in a different color. The genes found by annotation are indicated within each BAC clone and their corresponding localization in the *O. niloticus* chromosomes are denoted by crossing lines. The clones analyzed are given in Table 1.

Figure S3 Circos analysis in the species *G. aculeatus.* On the left side the distribution of the BAC clones of Senegalese sole distributed in chromosomes can be observed. Each BAC clone is represented in a different color. The genes found by annotation are indicated within each BAC clone and their corresponding localization in the *G. aculeatus* chromosomes are denoted by crossing lines. The BAC clones analyzed are given in Table 1.

DATA SHEET S1 Sequences used for the construction of the phylogenetic tree.

DATA SHEET S2 Saturation indices and plots with transitions (s) and transversions (v) for each gene and in the concatenated sequence.
