# Supplementary figures and images for: Genome and Phylogenetic Analysis of Genes Involved in the Immune System of Solea senegalensis – Potential Applications in Aquaculture
Source: Front Genet. 2019 Jun 11;10:529. doi: 10.3389/fgene.2019.00529 (PMC6579814; doi:10.3389/fgene.2019.00529)

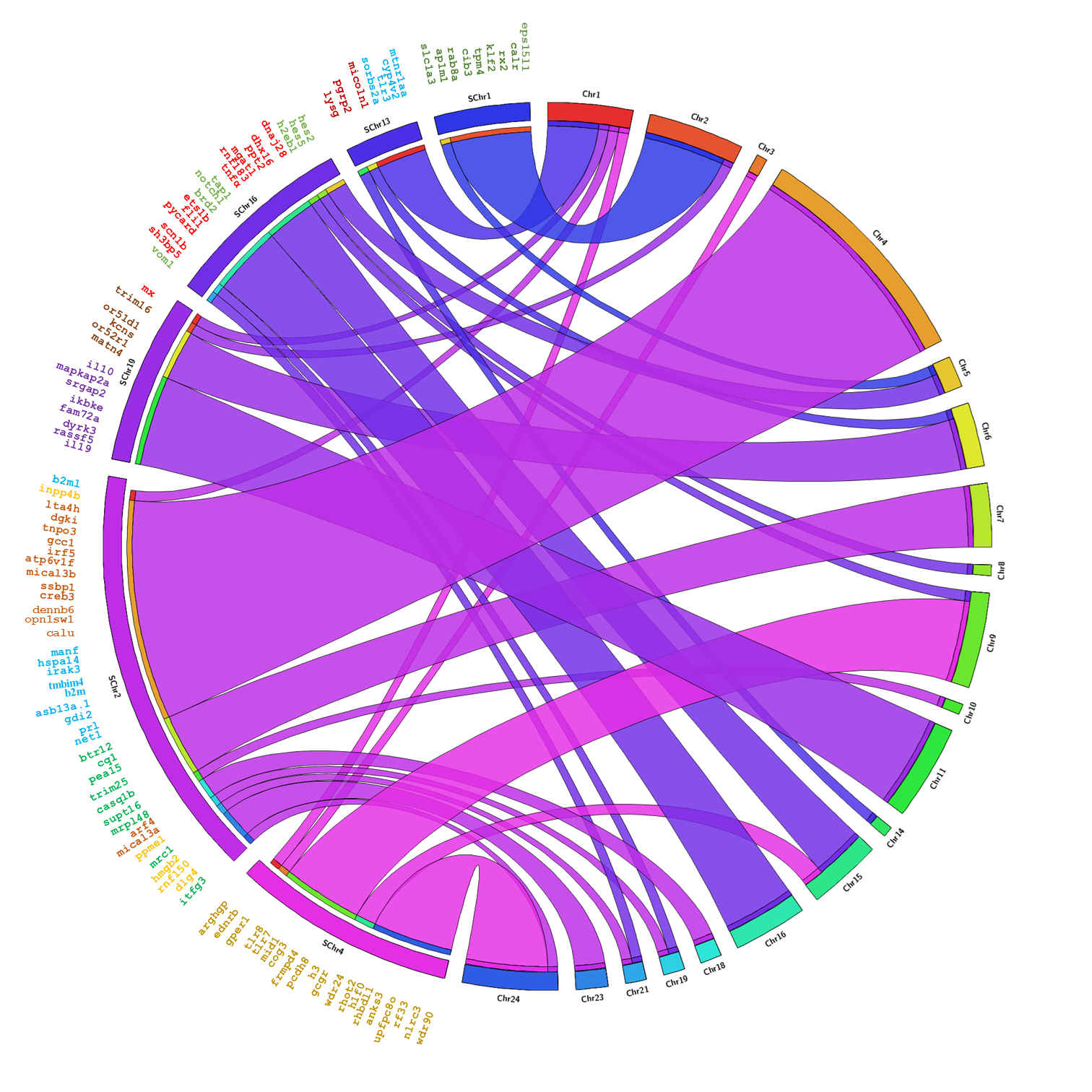

Supplement: Figure S1 — Circos analysis in the species D. rerio. On the left side the distribution of the BAC clones of Senegalese sole distributed in chromosomes can be observed. Each BAC clone is represented in a different color. The genes found by annotation are indicated within each BAC clone and their corresponding localization in the D. rerio chromosomes are denoted by crossing lines. The BAC clones analyzed are given in Table 1. [file Image_1.TIF]

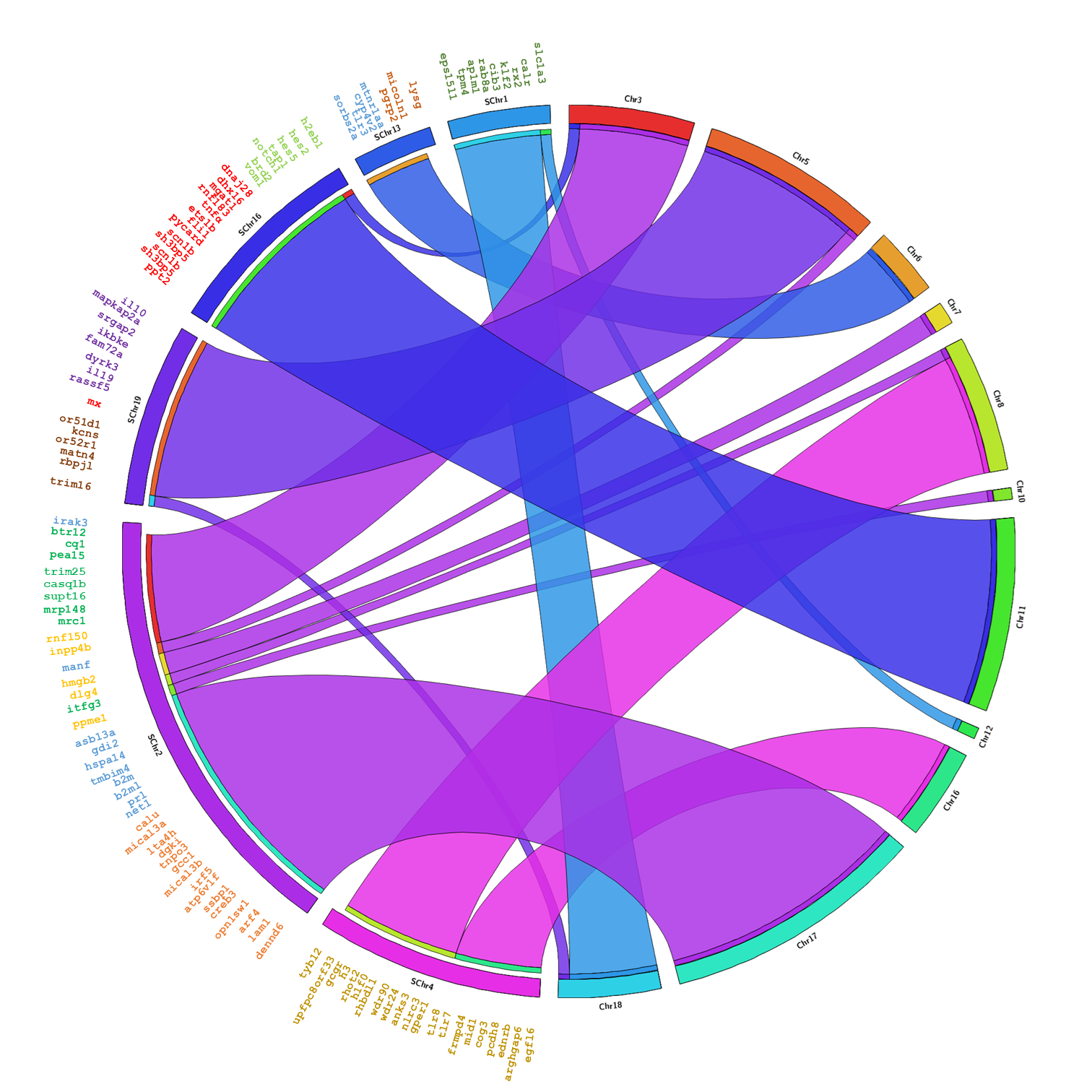

Supplement: Figure S2 — Circos analysis in the species O. niloticus. On the left side the distribution of the BAC clones of Senegalese sole distributed in chromosomes can be observed. Each BAC clone is represented in a different color. The genes found by annotation are indicated within each BAC clone and their corresponding localization in the O. niloticus chromosomes are denoted by crossing lines. The clones analyzed are given in Table 1. [file Image_2.TIF]

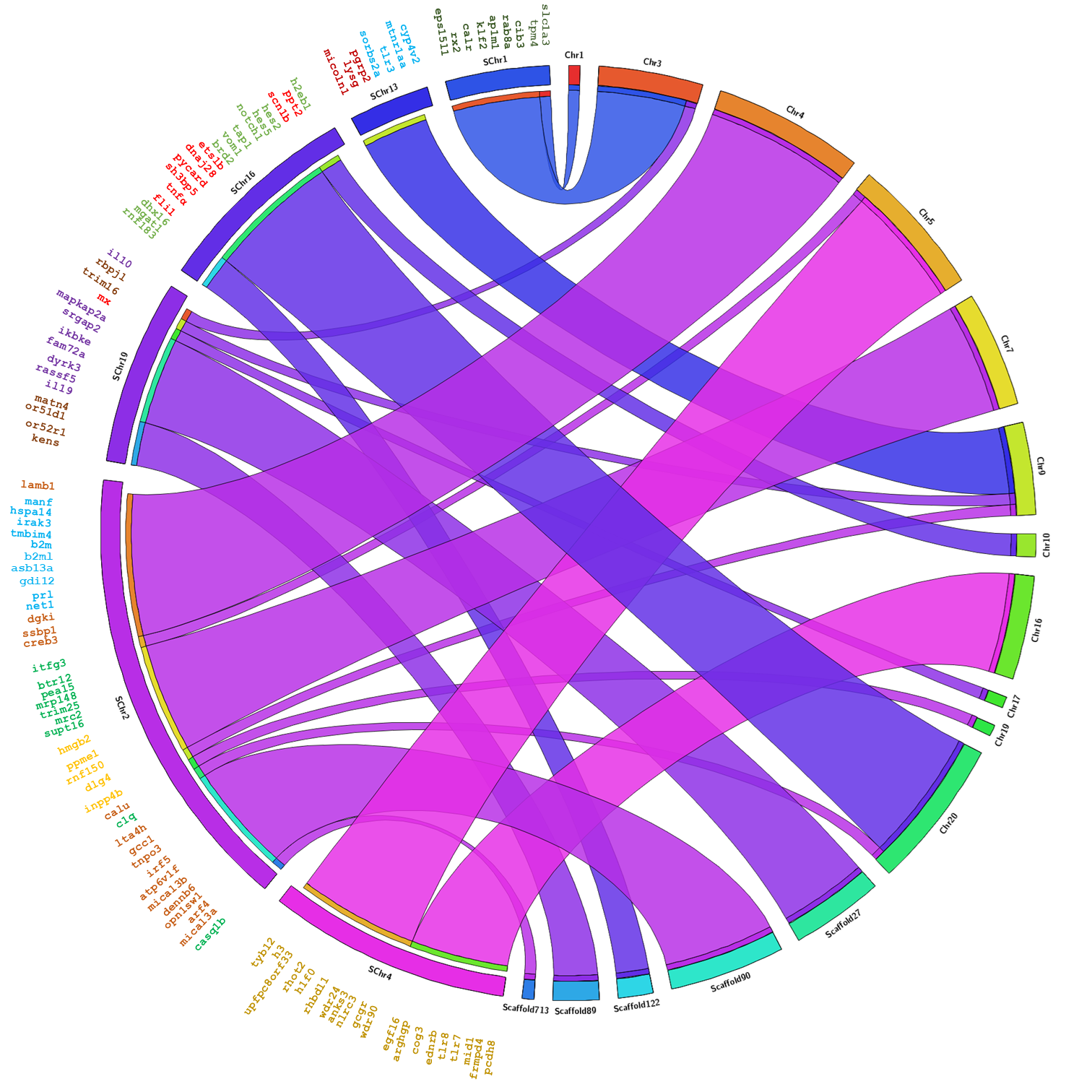

Supplement: Figure S3 — Circos analysis in the species G. aculeatus. On the left side the distribution of the BAC clones of Senegalese sole distributed in chromosomes can be observed. Each BAC clone is represented in a different color. The genes found by annotation are indicated within each BAC clone and their corresponding localization in the G. aculeatus chromosomes are denoted by crossing lines. The BAC clones analyzed are given in Table 1. [file Image_3.TIF]
