# Supplementary material for: Genome and Phylogenetic Analysis of Genes Involved in the Immune System of Solea senegalensis – Potential Applications in Aquaculture
Source: Front Genet. 2019 Jun 11;10:529. doi: 10.3389/fgene.2019.00529 (PMC6579814; doi:10.3389/fgene.2019.00529)
Supplement: DATA SHEET S1 — Sequences used for the construction of the phylogenetic tree. [file Data_Sheet_1.PDF]

```
>Poecilia formosa tlr3-tlr8-nlrc3-calr-ikbke
CCTTCTTCTTGCTTCAGAACCTGAAATATCTTGACGTCTCCATGAACAACCTGAAGTCAGTCAGGCTCGG
CTCTCAGCCTCAGCTGCCCAGCTTGGAAACATTGGAACTCAAACACAATAACTTCAACACTCTGAAAAGT
AATGATTTTTCCTTCCTGAAGGATTCACCCCTCCATGTCATCAGCCTGATGTTTTCTGTGTCCCTAGAAA
AATTGGAGCCTGGTTGCTTTAAGCCTGTTCCAGGCCTGCGTATGTTATTCCTCGATGGTAGCAGTAAGGG
CTTCATGTTCTTGTCTGACCTCTGTTCAGAGCTGTCTGAAACAGCAATCAGTGCCATGTTTCTGAGGAAC
ATTGGGCTGAAGAATCTGACAAACAAAACCTTCTCAGGTTTGAAGAAAACAAACCTAACCCTTCTGGATC
TGTCTGGCAATAGAATTGGCATAATTGAAGATGGCTCCTTTCAGTCGCTATCTGAACTTCAGACTCTTAA
CTTGACTGGCAACAGCATTAAGCACCTGACACAAAAAACTTTTCAAGGCTTAAAAATGTTGAAAAACCTA
CAGCTGACTAAAGCGCTAGTGAAAAGTCAGAAACCCACAACCCCTGTTATTGAGGATTTCACATTTCAAC
CACTAAGCAACCTTGAGACCCTGATGTTACAAAACACTCCATTTGTCAACATCACAGAGAATACCTTCAA
AGGTCTGATGAGTCTGAAAGATCTTGACTTGAGCTGGAGCAATTCTATATTCACAAGCAAAAAACTCTCC
AGTAAGTTCTTCGTCTCTCTTGCCGATTCACCCCTCAGGAAGTTAAACCTAGAAGGAAATGCTTTGGTGG
GAATTGAACCCGGGACCTTCTCCTGTCTGAAAAGTCTCTCCATTCTTTTTCTTGATCGTAATTATATTCG
TCAAACATTCAGCGGCAAAGAATTGGGAGGTCTCGCTCAGTTACAAGAGCTTCACATAAGCAATAACGTC
CAGCAAATTAATCTGACCGCAGAGACATTTGCCAGTGTGACTGATCTTAGAGTGCTGATGCTGGGAAAGA
GTCTGCAAGCTGAAACCTTGAACCAGGATTCGTCTCCGTTTCGGCCTTTGTCCAACCTCACTGTATTGGA
TCTCAGCAACAACAACATTGCGAACATCAGAGAGAATTTGTTAGAGGGGCTTGAGAACCTGCGGGTCCTG
AAGCTCCAACATAACAACTTAGTCAGGTTGTGGAAGATGGCAAACCCTGGTGGGCCTGTCCTGTTTCTCA
AATACACACCGAGGCTGGTAAGCTTTGAGATGGATTTCAATGGAATGGATGAGATTCCAAGAGAAGCTTT
CAGGAATTTGAATCAACTCAGGTACCTTAGCTTTAGCAACAATCTTTTGAACAATTTACAGGACTCGGTG
TTAGACGACCTTACGTCCCTTAAGGTTTTACGTCTGGAGAAGAATTTTATCACTTCTGTCAGGCCAGAGG
TGTTCAGAACTCCCATGAGCAACCTCAGTGTCCTTGTCATGGGAAAAAACCCGTTCGACTGTACCTGTGA
GAGCATCCTTTGGTTTGCCACGTGGTTAAACAAAACTAACGCTAGCGTGCCAGGTCTCAGAGACCAGTAC
ATGTGCAACACCCCAATACTTTACTTCAATCATTCCATCATGGACTTTGACCAACTTTCTTGCAAAGACA
TGGCCCCATTCCAGGCTTTTTATGTGCTAAACAGCACAATTGTTTTAATGCTGATTCTCACTGCCCTTAT
TTATCGATTCCATGGCTGGAGGATCCTGTTCTACTGGAACATATTGATCAATCGTACATTAGGATTTAGT
GATGCCAAAGTGGAGGAGGGCAGAGAGTTTGAGTATGACGCTTATGTCATACACGCTGAGGAAGATACAC
GCTGGGTGGAGAGACGACTGGTTCCTTTGGAGAACGAAACCTGCAGGTTTTGCTTGGAGGATCGAGATTC
TGTCATCGGCATGTTCCAGGCTGACTCCATCGTGAATAATATGAAAAAATCTAGAAAAATCTTGTTTGTG
GTGACTGATCGTCTTCTCAACGATCCCTGGTGTAGACGATTTAAAGCCCACCATGCACTTCAACAGGTCA
TCGAAGCCAGCAGAGACTCGGTTGTCCTGATTTTTCTGCAGGATGTGCACGACTACAAGTTGTCTCGCTC
ACTTTTCCTCCGCAGAGGCATGCTGCATCAAAGGTGCGTCCTGAACTGGCCTGCGCAGAAGGAGAGGATC
CCAGCCTTCAACCAGAAGCTTCTCATTGCTCTGGGAATGACTAATCGACTGCAGGAGCTTATAAAGCAAC
AGTGCTTTAACTCTGGACGGGTTCTTGTCCTCAGTTCAAATAACTTGTTCTTCATCTCACAAAAGCAATT
TGAAGGTTATGGAGATATTGCGTGTCTGAATCTATCCAGCAATGGATTCTCTGCTGCACCAAATGGCACA
GAGTTCTCATTACTGCCTAATTTGACCTACTTGGACCTGTCCTTCAATAAGATTGATCTGGCTTATGACA
ATGCCTTCCAGGAACTGAAGAAACTCGAAGTGCTAGATCTCAGTTGGAATGCCCACTACTTTAAATCTTT
TGGAGTAACACATAATTTAAACTTCCTAAAAAATTTGCCTATCCTGAGAGTGCTGAATATGAGTCATAAC
TCAATTTCCACTTTAACAACAAAGCAGATGCACAGCAGATCTTTAGCAGAACTACAATTTACACATAACA
AGCTTGGAACTCTTTGGAAAGTAGGTGATGACTCATATAAGACTCTTTTTACCAACCTGACTAATTTGAC
AGTTTTGGATATTTCGTGGAACGACATTGAAAAAATTCCTGATGATGTTTATGAAAAGCTCCCTCGCAAC
CTAACTGAACTACGAATAAATAATAACAAAATGACAGATTTTTCATGGGACAGTCTGTCTTATTTCCACC
GGCTTAAAATTTTAGACTTAAGTTACAATTCGTTGTCCAACATAGCAGGTATTGAAACAGTTACTAGTTT
CTCTTTGACTTTTCTTTATCTGACTCACAATCAGATATGTCACCTGGAAAGTTTCTATTTCAAAGGCATG
AAAAGCCTTGAGACTCTCAGCCTCGGCTCCAACAAGCTGGCTATCATCAATCAAACCACCTTCGAGCCCA
GACCGGAGAATCTTAAGACTTTATTTTTACAGAAAAATCCATTTCAATGTACATGTGATTCTTTGGATTT
CCTTGTATGGATTGGAAAACGAGAAATAAGGATTCCTAGATTGACAACTGAGGTGAGATGTGAAATGCCA
TTAAAGGACAGGAACAAGCCACTTATATACTTTGACATTGATCAATGTGTAAATGACGATATGGCGTTCA
AGATGTACGTTCTCACAACATTCTTCCTTATTTCCTTTATGTTTGTGGCAACAGTTGCTCACTTATTTTA
CTGGGATGCTTCTTATGTCTTGCACTACGTCAAGGCTAGACTGAAGGGATACAGAGCCTTGTCCTCACAG
GAAAATATTTACGACATCTTTGTGACTTATGACACCAAGGATCCACGTGTCACTGAGTGGGTAATGATGA
ATCTGCGGGTGAAATTGGAAGAGGAGGGCGATAAGCTTCTTCCTTTGTGCTTGGAGGAGAGAGACTGGCC
TCTAGGAGTCCCTCTAGTGGATAACCTTACACAGAGCATCCAAAACAGCCGCAAAACCCTGTTTGTCTTG
ACAGAGGGCTACGTTAAGACTGGGGTGTTCAGACTGGCAATGTATCTGGCCCACCAAAGACTGCTGGATG
AAAATGTGGATGTGATTGTTCTGCTGATGCTCGAACCTGTCCTGCAACATTCTCACTTCCTACGCCTCAG
GAGGAGGCTTTGTGAGAAAAGTGTTGTGGAGTGGCCCAGAACTGCAGCTGCTGAGCCCTGGTTCTGGCAG
AACTTGAGAAGTGTTGTAAAAGTGGATAATCAAATTATGTACAGCAAAACTTATAAAAAGTTTGGAGGCC
CATAAGATCCTAATGGTGATGTGCTACATACCATGCATTTGCTGGATGCTAGCAGAGACGTTGCTGTACA
TCATGCAGAGCGGGACACAGGAGAGCCTGCCTAAGACCTGCACTGAGCTGTACGCCCATTTCTGCTCCAT
GAAAGCAGAAGTAGGGGAACCAAGAGGCAGGGAGCCTGTAAAGTCTGAGCAGCATCATGCGACCAACCGC
```

AAACTGCTGGGCAACCTGGGACGCCTGGCCTTCTACAGCCTCCTGAAGCACAAGTACACGTTCAGCGAGC
AGGACCTCAGGGCCTACGGGATAGACTTACTGCAAACTCAGTGCAGCCTTGGAGTTGGAGTTCTGGTCCG
GACGGAGTCCACCATACACACCACTTACAGATTTACACACCTGACCCTGCAGGAGTTTCTTGCAGCTACG
TTTTATCATGTTTCCTCCAAGCGGGCCATCTTTGACCTGTTCTCAGAGAGCACCATGTCCTGGCCCAAGA
TCGGGTTCCAGAACCACTTCAGAAGCGCCTTCCAACACTCGCAGCAAGCCGAAGACGGCCACCTGGATCT
GTTTGTGCGCTTCCTGACAGGCTTGCTGTGCCCAACAACGATGAAACCTCTGGCTGGGCTCCTGGCTCTC
GGGAAAGACGATGGCAGCCAGAAGGTTTGGGCTGGCGGGTTTTTACACAGCCTCCTGTCCAGCGTGGGTT
CGGTGGTGTCCCTCCGAGCCGTCAACCTGGCCTACTGCTTACAAGAGCTGCAACACGGCGAGCTGCTCCG
GAGCGTAGAGGAGGACCTGCGGCAGGGCAGCTTGGCTGGAAAACTGACGCGGCCGCACTGCGTGGTGCTG
GGCTTCCTGCTGCACGTCTCTCCAGAGTGCAGTGAACAAACCAACCTGACAGGATGTCTGAACTACCCAA
CGGTGAAAAGTTTACTCCCACAGCTGCTGTACTGCACCCATCTCAGGCTGGAGAATAATAACTTCAAGGA
CGATGTCATGGAGCTGCTCGGGAGCCTCCTGAGCGCCAAAGACTGCCACATCCGGAAAATAAGTTTGGCC
GACAATGTCATCAGCAACAAAGGAGCCAAAGCTCTGAGCCGAGCCCTGCTGGTGAACCGAACCCTGACCG
CCATCAACTTAAAGAACAACAGCATTGGATCCAAAGGGGCAAAGTTTCTGGCAGAGGCTCTGAAAATGAA
CCAAGCCTTGGTATCAATCAACCTGCAGAATAACTCCATCGGGGAAGAAGGAGCTCAGGCCATCGCAGAC
GTCCTGCAGTCCAACCGCAAACTCGTGTCTCTGAACATGCGGAAAAATACAATTGGCGCAGGAGGAGCCA
AGAGGATCGCAGAGGCGCTGAAGACAAACCGAACTCTTACAAAGTTGATACTCTGCAGTAACCAGCTGGG
GGACAAAGGAACCGTGGCTCTGGCTGAAGCTCTGACTTTCAACCACACGCTGCTGTCGCTCCAACTTCAG
AGTAACTCGATCAGCAACAAGGGAATGACGGGCCTGACCAAAGCGCTGAGGCTCAACCGGGGACTCGTCT
CTCTGAATTTGAGGGAGAACTCCATCGGGGTGGAGGGAGCCAGGAACATGGCTCACGCTCTGCAGGAGAA
CAACTCTCTGCAAAACCTCGATCTCACAGCTAACCTGCTGCACGACGACGGGGTTCAAGCAATCGCTGGA
GTTTCTGGACGGTGATGAATGGAGAAGTCGCTGGGTGAACTCCAAGCATAAGTCTGACTATGGAGAGTGG
AAACTGACTGCTGGAAACTTCTATGGAGATGCTGAAAAAGACAAAGGTCTGCAGACGAGCCAAGATGCCC
GCTTCTACGCCGTCTCTGCCAGCTTCGACCCGTTCAGCAACGAGGGGAAGTCTTTGGTCATTCAGTTTAC
GGTCAAGCATGAGCAGAAGATCGACTGTGGTGGCGGCTACGTGAAGGTCTTCCCTGCTGACTTGGAGCAG
ACTGAAATGCATGGGGAATCCTCATACTACATCATGTTTGGCCCTGACATCTGTGGCTACAGCACCAAGA
AAGTCCACGTCATCTTTAACTACAAGGGCAAGAATCACTTGATCAAGAAGGAGATTAAGTGCAAGGACGA
TGAGCTGACCCACCTGTACACGCTGATCCTGAACCCAGATCAGACCTACGAGGTGAAGATTGACAACGAG
AAAGTGGAATCTGGCAGTCTGGAGGAAGACTGGGACTTCCTGCCTCCCAAGAAAATTAAGGATCCCGAAG
CCAAGAAACCAGAAGACTGGGATGACCGCCCCAAGATCGACGACGCCGATGACACAAAACCCGAGGACTG
GGATAAACCTGAAAACATTCCTGATCCTGATGCTAAGAAGCCTGAAGACTGGGATGAAGACATGGATGGA
GAATGGGAGCCACCAATGATCCCCAACCCAGAGTACAAAGGAGAGTGGAAACCCAAACAGATCGACAACC
CCAACTACAAGGGAGTCTGGGTTCATCCTGAAATTGACAATCCCGACTACAGTCCCGACTCCACCATCTA
CAAGTTCGACAACATTGGCGTTCTTGGCCTTGACCTTTGGCAGGTGAAATCTGGTACGATCTTTGACAAC
TTTTTGATCACCGACGACGTGAAGGAAGCTGAAGAAATCGGGAAGGAGACGTGGGGCGTGACGAAGGAAC
CGGAGAAGAAAATGAAGCAAGAGCAGGATGAGGTGAAGCGAAAAGAGGAAGAGGAGAAGAACAAAGAGCA
AGCCACTGAGGGCGATGATGACGACGATGAGGTGGAAGAGGACGAAGAGGAGGATGAAGATGCAAAGGAC
GAGACTCATACAGAAAATGAAGACGACGAGTCAAAGCCCAAAGATGAGCAAACAAAGCAGAAGGTCCTGG
TGATGGAGTATTGTTCAGGAGGGAGTCTGCTCAACCTGCTCGAGGACCCAGAAAACGCCTTTGGCTTGCC
TGAAGCAGAGTTCCTCACTGTCTTGCAGTGCATAGTGCAGGGTATGAATCATCTGCGGGAATACGGAGTA
GTGCATCGGGACATCAAGCCGGGGAACATAATGAGGCAGGTTGGAGAGGACGGCAAGTCCGTCTACAAGC
TGACGGACTTCGGAGCGGCGCGAGAGCTGGAAGACGACGAGAAGTTTGTGTCCATTTATGGAACTGAGGA
ATATCTGCACCCAGACATGTACGAACGCGCTGTGCTGCGCAAGCCTCATCAGAAATCCTACGGCGTGAGC
GTTGACTTGTGGAGCATCGGCGTGACCTTGTACCACTCCGCCACGGGGAGTCTCCCTTTCACACCTTTCG
AAGGGCCCCGTAAAAACAAACTCACCATGTACAAGATAACCACAGAGAAGCCCATGGGAGCGATAGCCGG
CACGCAGCGGGCGGAAGGCGGTTCTATAGAGTGGAGCTACCACTTGCCTTACAGCTGCCAGCTTTCACAG
GGTCTGAAAGCCCTTCTAGTTCCCGTCCTCGCAGGCATAATG

>Xiphophorus maculatus tlr3-tlr8-nlrc3-calr-ikbke
CCTTCTCCTTGCTTCAGAACCTGAAATATCTTGACGTCTCCATGAACAACCTGAAGTCAGCCAGGCTTGG
TTCACAGCCTCAGCTGCCCAGCTTGGAAACCTTAGAACTCAAACACAATAACTTCAACACTCTGAAAAGG
GAGGATTTCTCCTTCCTGAAGGATTCACCCCTCCATGTCATCAACCTGATGTCGTCTGTGTCCCTACAAA
AATTGGAGCCTGGTTGCTTTAAGCCTATTCCAGGCCTGCGTATGTTATTCCTCAATGGTAGCAGAAAGGG
CTTCATGTTCATATCTGACCTCTGTTCAGAGCTGTCTGAAACAGCAATCAGTGCCATGTCTCTGAGGAAC
ATCGGGCTGACGAATCTGACAAACAAAACCTTCTCAGGTTTGAAGAAAACAAACCTAACCCTTCTGGACC
TGTCCGGCAACAGCATTGAGATAATTGAAGATGGCTCCTTTCAGTGGCTCTCTGAACTTCAGATTCTTAA
CTTGACTGGCAACGGCATTAAGCACCTGACACGAGAAACTTTTAAAGGCTTAAAAATGTTGAAAAACCTA
CAGCTGACTAAAGCGCTGGTGAAAAGTCAGAAACCCTCTCTTCCTGTTATTGAGGATTTCACGTTTCAAC
CACTAAGCAACCTTGAGACTCTGGTGTTACAAAACACTCCGTTTGTCAACATCACAGAGAATACCTTCAA
AGGTCTGATGAGTCTCACAGATCTTGACTTGAGCTGGAGCAATTCTATATTCACAAGCAAAAAACTCTCC
AGTAAGTTTTTTGTCTCTCTTGCCGATTCACCCCTCAGAAAGTTAAACCTAGAAGGAAACGCTTTGGTGG

GAATTGAACCCGGGAGCTTCTCCTGTCTGAAAAGTCTCTCCATTCTTCTTCTTGATCGCAATTATATTCA
TCAAACATTCAGCGGCAAAGAATTGGAAGGTCTTGCTCAGTTACAAGAGCTTCACATAAGCAATAACGTC
CAGCAAATTAATCTGACCGCGCAGACATTCGCCAGTGTGACTGATCTTAGAGTGCTGATGCTGGGAAAGA
GTCTGCAAGCCGAAACCTTGAACCAGGATTCCTCTCCGTTTTGGCCTTTGTCCAACCTCACTGTATTGGA
TCTCAGCAACAACAACATTGCGAACATCAGAGAGAATTTGTTAGAGGGGCTTGAGAACCTCCAGGTCCTG
AAGCTCCAACATAACAACTTAGTCCGGTTGTGGAAGACGGCAAACCCTGGTGGGCCTGTCCTGTTTCTCA
AACACACACCGAGGCTGGTAAGCTTTGAGATGGATTTCAATGGAATGGATGAGATCCCAAGAGAAGCTTT
CAGGAATTTGAATCAACTCAGGGAGCTTAACTTTAGCAACAATCTTTTGAACAATTTACGGGACTCGGTG
TTAGACGACCTTACGTCCCTTAAGGTTTTACGTCTGGAGAAGAATTTTATCACCTCTGTCAGGCCAGAGG
TGTTCAGAACTCCCATGAGCAACCTCAGTGTCCTTGTCATGGGAAAAAACCCATTCGACTGTACCTGCGA
GAGCATCCTGTGGTTTGTCACGTGGTTGAACAAAACTAACGCTAGTGTGCCAGGACTCAGAGACCAGTAC
ATGTGCAACACCCCCATACTTTACTTCAATCACTCCATCATGGACTTTGACCAACTTTCTTGCAAAGACA
TGGCCCCATTCCAGGCCTTTTATGTGCTGAACAGCACAGCCGTTTTAATGCTGATTCTCACTGCCCTTAT
TTACCGATTCCACGGCTGGAGGATCCTGTTCTACTGGAACATATTGATCAATCGCACATTAGGATTTAGT
GACGCCAAAGTGGAGGAGGGCAGAGAGTTTGAGTATGACGCTTATGTCATACACGCTGAGGAAGACACCC
GCTGGGTGGAGAGACGACTGGTTCCTTTGGAGAACGAAACCTGCAGGTTTTGCTTGGATGATCGAGATTC
TGACATCGGCATGTTCCAGGCTGACTCCATCGTGAATAATATGAGAAAATCTAGAAAAATCTTGTTTGTG
GTGACCGAGCGTCTTCTCAACGATCCCTGGTGTAGACAATTTAAAGCCCACCATGCACTTCAACAGGTCA
TCGAAGCCAGCAGAGACTCGGTTGTCCTGATTTTTCTGCAGGATGTGCACGACTACAAGTTGTCTCGCTC
ACTTTTCCTCCGCAGAGGCATGCTGCGTCCAAGCTGCGTCCTCAACTGGCCTCCGCAGAAGGAGAGGATC
CCTGCCTTCAACCAGAAGCTCCTCATTGCTCTGGGAATGACTAATCGACTGCAGGAGCTTATAAAGCAAC
AGTGCTTTAACGCTGGACGGGTTCTTGTCCTCAGTTCAAATAACTTGTTCTTTATTTCACAAAAGCAATT
TGAAGGTTATGGAGATATTGCGTGTCTGAATCTATCTAGCAATGGATTCTCTGCTGCACCAAATGGCACA
GAGTTCTCATTACTGCCTAATTTGACCTACTTGGACCTGTCCTTCAATAAGATTGATCTGGCTTATGACA
ATGCCTTCCAGGAACTGAAGAAACTCGAAGTGCTAGACCTCAGTTGGAATGACCACTATTTTCAATCTTT
TGGAGTAACACATAATTTAAACTTCCTAAAATATTTGCCTGTCCTGAGAGTGCTGAATATGAGTCATAAC
TCAATTTCTACTTTAACAACTAAACAGATGCACAGCAGATCTTTAGAAGAACTACAATTTACACATAACA
AGCTAGGGACTCTTTGGAAAGTAGGTGATGACTCATATAAGACTCTTTTTACCAACCTCACTAATTTGAC
AGTTTTAGATATTTCGTGGAATGACATTGAAAAAATTCCTGATGATGTTTATGAAAAGCTCCCTCGCAAC
CTAATTGAACTACGAATAAGTAATAACAAAATGACAGACTTTTCATGGGACAGTCTGACTTATTTCCATC
AGCTTAAAATTTTAGACTTAAGTTACAATTTGTTGTCCAACATAGCAGGTATTAAAACAGTTAGTAGTTT
CTCTTTGACTTTTCTTTATCTGACTCACAATCAGATATGTCACCTGGAAAGTTTCTTTTTCAAAGGCATG
AAAAGCCTTGAGACTCTGAGCCTCAGCTCCAACAAGCTGGCTATCATCAATCAATCCACCTTCCAGCCCA
AACCGGAGAATCTTAAGACTTTATTTTTACAGAAAAATCCATTTCAATGTACATGTGATTCTTTGGATTT
CCTTGTATGGATTGGAAAACGACAAATAAGGATTCCTAGATTAACAACTGAGGTGAGATGTGCAATGCCA
TTAAAGGACAGGAACAAGCCACTTATATACTTTGACATTGATCAATGTGTAAATGACGATATGGCATTCA
AGATGTACGTTTTCACAACATTCTTCCTTATTTTCTTCATGTTTGTGGCAACAGTTGCTCACTTATTTTA
CTGGGATGCTTCTTATGTTTTGCACTATGTCCAGGCTAAACTGAAGGGATACACAGCCTTGTCCTCACAG
GAAAATATTTACGACATCTTTGTGACTTATGACACCAAAGATCCACGTGTCTCTGAGTGGGTAATGATGA
ATCTGCGGGTGAAATTGGAAGAGGAGGGCAATAAACTTCTTCCTTTGTGCTTGGAGGAGAGAGACTGGCC
TCCAGGAGTCCCACTAGTGGATAACCTTACACAGAGTATCCAAAACAGTCGCAAAACCCTGTTTGTCTTG
ACGGAGGGCTACGTTAAGACTGGGGTGTTCAGACTGGCAATGTATATGGCCCACCAGAGACTGCTGGATG
AAAATGAGGATGTGATTGTTCTGCTGATGCTCGAACCTGTCCTGCAACATTCTCACTTCTTGCGCCTCAG
GAGGAGGCTTTGTGAGAAAAGTGTTGTGGAGTGGCCCAGGACTCCAGCTGCTGAGCCCTGGTTCTGGCAG
AACTTGAGAAGTGTTGTAAAAGTGGATAATCAAATTATGTACAGCAAAACGTATAAAAAGTTTGGAGGCC
CATAAGATACTAATGGTGATGTGCTACATACCATGCATTTGCTGGATGCTAGCAGAGACGTTGCTGTACA
TCATGCAGAGCGGGACACAGGAAAGCCTGCCTAAGACCTGCACTGAGCTGTATGCCCATTTCTGCTCCAT
GAAAGCAGAAGTAGGTGAACCAAGAGGTAGGGAGCCTGTAAAATCTGAGCAGCATCATGCAACCAATCGC
AAACTGCTGGGCAACCTTGGACGACTGGCCTTCTACAGCCTCCTGAAACATAAATACACATTCAGCGAGC
AGGACCTCAGGGCTTATGGGATAGACTTACTGCAAACTCAATGCAGCCTTGGAGTTGGAGTTCTCATCCG
AATGGAGTCTACCATACACACCACTTACAGGTTTACACACCTGACCCTGCAGGAGTTTCTTGCAGCTACG
TTTTATCATGTTTCCTCCAAGCGGGCCATCTTTGACCTGTTCTCAGAGAGCACAATGTCATGGCCCAAGA
TTGGGTTCCAAAACCACTTCAGAAGCGCCTTTCAACACTCGCAGCAAGCTGAAGACGGTCACTTGGATCT
ATTTGTGCGCTTCCTGACAGGCTTGCTGTGCCCAACAACGATAAAACCTCTGGCTGGGCTCTTGGCTCTC
GGGAAAGACGATGGAAGCCAGAAGGTTTGGGCTGGAGGGTTTTTACAAAGCCTCCTGTCCAGCGTGGGTT
CGGTGGTGTCCCTCCGTTCTGTCAACCTGGCCTATTGTTTACAAGAGCTGCAACATAGCGAGCTGCTCCG
GAGTGTAGAGGAGGACCTACGGCAGGGCAGCTTGGCTGGAAAACTGACACGGCCACATTGTGTGGTGCTG
GGTTTCCTGCTGCATGTCTCTCTAGAGTGCAGTGAACAAACCAACCTGACAGGATGTCTGAACTACCCAA
CGGTGAAAAGTTTACTCCCACAGCTGCTGTACTGCACCCATCTCAGGCTGGAGAATAATAACTTCAAGGA
CGACGTCATGGAGCTGCTCGGGAGCCTCCTGAGCGCCAAAGACTGCCACATCCGGAAAATAAGTTTGGCG
GACAACGTCATCAGCAACAAAGGAGCCAAAGCCCTGAGCCGAGCCCTGCTGGTGAATCGAACTCTAACCT

CTATCAACTTAAAGAACAACAACATTGGATCAAAAGGGGCAAAGTTTCTGGCTGAGGCTCTGAAAATGAA
CCAAGCCTTGGTATCAATCAACCTGCAGAATAACTCCATCGGGGAAGAAGGAGCTCAGGCCATCGCAGAC
GTCCTGCAGTCCAACCGCAACTCGTGTCTCTAAACATGCGGAAAAATACAATTGGTGCGGGAGGAGCCAA
GAGGATCGCAGAGGCGCTGAAGACAAACCGGACTCTTACAAAGTTGATACTCTGCAGTAACCAACTGGGG
GACAAAGGAACCGTCGCTCTGGCTGAAGCTCTGACTTTCAACCACACGTTGCTGTCGCTTCAACTTCAGA
GTAACTCGATCAGCAACAAGGGAATGACGGGCCTGACCAAAGCGCTGCGGCTCAACCGGGGACTCGTCTC
TCTGAATTTAAGGGAGAACTCCATTGGGGTGGAGGGAGCCAGGAACATGGCTCACGCTCTGCAGGAGAAC
AACTCTCTGCAGAACCTCGATCTCACAGCTAACCTGCTACACGACGACGGGGTTCAAGCAATTGCTGGAG
TTTCTGGACGGTGATGAATGGAGAAGTCGCTGGGTGAACTCCAAGCACAAGTCTGACTATGGAGAGTGGA
AACTGACTGCTGGAAACTTCTATGGAGATGCTGAAAAAGACAAAGGTCTGCAGACGAGCCAAGATGCCCG
CTTCTACGCCGTCTCTGCCCGCTTCGACCCGTTCAGCAACGAGGGGAAGCCTTTGGTCATTCAGTTTACA
GTCAAGCATGAGCAGAAGATCGACTGTGGTGGCGGCTACGTGAAGGTCTTCCCCTCTGACTTGGAGCAGA
CCGAAATGCATGGGGAATCCTCGTACTACATCATGTTTGGCCCCGACATCTGTGGCTACAGCACCAAGAA
AGTTCACGTCATCTTTAACTACAAGGGCAAGAATCACCTGATCAAAAAAGAGATTAAGTGCAAGGATGAT
GAGCTGACCCACCTGTACACGCTGATCCTGAACCCAGATCAGACCTACGAGGTGAAGATTGACAATGAGA
AGGTGGAATCTGGCAGCTTGGAGGAAGACTGGGACTTCCTGCCTCCCATGAAAATTAAGGATCCTGAAGC
CAAGAAACCAGAAGACTGGGATGACCGCCCCAAGATTGACGATGCCGATGACACAAAACCTGAGGATTGG
GATAAACCTGAGAACATTCCTGATCCTGATGCTAAGAAACCTGAAGACTGGGATGAAGACATGGATGGAG
AATGGGAGCCACCGATGATCCCCAACCCAGAGTACAAAGGAGAGTGGAAACCCAAACAGATCGACAACCC
AAACTACAAGGGAGTCTGGGTGCATCCTGAAATTGACAATCCCGACTACAGTCCTGACTCCGCCATCTAC
AAGTTCGACAACGTTGGCGTTCTTGGCCTTGACCTTTGGCAGGTGAAATCCGGTACGATCTTTGACAACT
TTGTGATCACCGACGACGTGAAGGAAGCAGAAGAAATCGGGAAGGAGACGTGGGGTGTGACGAAGGAACC
AGAGAAGAAAATGAAGCAAGAGCAGGATGAGGTGAAGAGAAAAGAGGAAGAGGAGAAGAACAAAGAGCAA
GCCACTGAGGGCGATGAAGACAACGACGAAGACGAAGAGGTGGAAGAGGACGAAGAGGAGGATGACGATG
CAAAGGACGAGATTCTTACAGAAAATGAAGATGACGAGTCAAAGCCCAAAGATGAGCAAACAAAGCAGAA
GGTCCTGGTGATGGAGTATTGTTCAGGAGGGAGTCTGCTCACCCTGCTTGAGGACCCAGAAAACGCATTT
GGCTTGCCTGAAGCAGAGTACCTCACTGTATTGCAGTGCGTAGTGCAGGGTATGAATCATCTGCGGGAAT
ACGGAGTAGTGCATCGGGACATTAAGCCGGGAAACATAATGAGGCAGGTTGGAGAGGACGGCAAGTCCGT
CTACAAGCTGACGGACTTTGGAGCAGCGAGAGAGCTGGAAGACGACGAGAAGTTTATGTCCATTTATGGA
ACTGAGGAATATCTGCACCCAGACATGTATGAACGCGCTGTGCTCCGTAAGCCTCATCAGAAATCCTACG
GAGTGAGTGTTGACTTGTGGAGCATCGGCGTGACCTTGTACCACTCTGCCACAGGAAGTCTCCCTTTCAC
ACCTTTTGAAGGACCCCGTAAAAACAAACTCACCATGTACAAGATAACCACAGAGAAACCCACGGGAGCA
ATAGCCGGCACACAGCGAGTGGAAGGTGGTCCTATAGAGTGGAGCTACCACTTACCTTACAGCTGCCAGC
TTTCACAGGGTCTGAAAGCCCTTCTAGTTCCCGTCCTCGCAGGAATAATG

>Poecilia reticulata tlr3-tlr8-nlrc3-calr-ikbke
CCTTCTCCTTGCTTCAGAACCTGAAATATCTTGACGTCTCCATGAACAACCTGAAGTCAGCCAGGCTCGG
CTCACAGCCTCAGCTGCCCAGCTTGGAAACCTTGGAACTCAAACACAATAACTTCAACACTCTGAAAAGT
GATGATTTTGCCTTCCTGAAGGATTCACCCCTCCATGTCATCAGCCTGATGTTTTCTGTGTCCCTAGAAA
AATTGGAGCCTGGTTGCTTTAAGCCTGTTCCAGGCCTGCGTATGTTATTCCTCGATGGTAGCAGTAAGGG
CTTCATGTTCATATCTGACCTCTGTTCAGAGCTGTCTGAAACGGCAATCAGTGCCATGTTTCTGAGGGAC
ATCGGGCTGAAGAATCTGACAAACAAAACCTTCTCAGGTTTGAAGAAAACAAACCTAAACCTTCTGGATC
TGTCTGGCAATAGGATTGGCATAATTGAAGATGGCTCCTTTCAGTGGCTGTCTGAACTTCAGACTCTTAA
CTTGACTGGCAACAGCATTCAGCACCTGACACAAAAAACTTTTCAAGGCTTAAAAATGTTGAAAAACCTA
CAGCTGACTAAAGCGCTGGTGAAAAGTCAGAAACCCTCAACCCCTGTTATTGAGGATTTCACGTTTCAAC
CACTAAGCAACCTTGAGACCCTGGTGTTAAAAAACACCCCATTTGTCAACATCACAGAGAATACCTTCAA
AGGTCTGATGAGTCTGAAAGATCTTGACTTGAGCTGGAGCAATTCTATATTCACAAGCAAAAAACTCTCC
AGTAAATTCTTCGACTCTCTTGCCGATTCACCCCTCAAGAAGTTGAACCTAGAAGGAAATGCTTTGGTGG
GAATTGAACCCGGGACCTTCTCCTGTTTGAAAAGTCTCTCCATTCTTCTTCTTGATCGTAATTATATTCG
TCAAACATTCAGCGGCAAAGAATTGGAAGGTCTCGCTCAGTTACAAGAGCTCCGCATAAGCAATAACGTC
CAGCAAATTAATCTGACCGCGGAGACATTTGCCAGTGTGACTGATCTTAGAGTGCTGATGCTGGGAAAGA
GTCTGCAAGCTGAAACCTTGAACCAGGATTCCTCTCCGTTTCGGCCTTTGTCCAACCTCACTGTATTGGA
TCTCAGCAACAACAACATTGCGAACATCAGAGAGAATTTGTTAGAGGGGCTTGAGAACCTCCGGGTCCTG
AAGCTCCAACATAACAACTTAGTCAGGTTGTGGAAGATGGCAAACCCTGGTGGGCCTGTCCTGTTTCTCA
AATACACACTGAGGCTGGTAAGCTTTGAGATGGATTTCAATGGAATGGACGAGATTCCAAGAGAAGCTTT
CAGGAATTTGAATCAACTCAGGTACCTTAGCTTTAGCAACAATCTTTTGAACAATTTACGGGACTCGGTG
TTAGACGACCTTACGTCCCTTAAGGTTTTACATCTGGAGAAGAATTTTATCACTTCTGTCAGGCCAGAGG
TGTTCAGAGCTCCCATGAGCAACCTCAGTCTCCTTGTCATGGGAAAAAACCCGTTCGACTGTACCTGCGA
GAGCATCCTTTGGTTTGTCACGTGGTTGAACAAAACTAACGCTAGCGTGCCAGGTCTCAGAGACCAGTAC
ATGTGCAACACCCCGATACTTTACTTCAATCATTCCATCATGGACTTTGACCAACTTTCTTGCAAAGACA
TGACCCCATTCCAGGCCTTTTATGTGCTAAACAGCACAGCTGTTTTAATGCTGATTCTCACTGCCCTTAT

TTATCGATTCCACGGCTGGAGGATCCTGTTCTACTGGAACATATTGATCAATCGCACATTAGGATTTAGT
GACGCCAAAGTGGAGGAGGGCAGAGAGTTTGAGTATGACGCTTATGTCATACACGCTGATGAAGACACAC
GCTGGGTGGAGAGACGACTGGTTCCTTTGGAGAATGAAACCTGCAGGTTTTGCTTGGAGGGTCGAGATTC
TGTCATCGGCATGTTCCAGGCTGACTCCATCGTGAATAATATGAGAAAATCTAGAAAAATCTTGTTTGTG
GTGACCGATCGTCTTCTCAATGATCCCTGGTGTAGACGATTTAAAGCCCACCATGCACTTCAGCAGGTCA
TCGAAGCCAGCAGAGACTCGGTTGTCCTGATTTTTCTGCAGGATGTGCACGACCACAAGTTGTCTCGCTC
ACTTTTCCTCCGCAGAGGCATGCTGCGTCAAAGCTGCGTCCTGAACTGGCCTCCGCAGAAGGAGAGGATC
CCAGCCTTCAACCAGAAACTTCTCATTGCTCTGGGAATGACTAATCGACTGCAGGAGCTTATAAAGCAAC
AGTGCTTTGACTCTGGACGGGTTCTTGTCCTCAGTTCAAATAACTTGTTCTTCATCTCACAAAAACAATT
TGAAGGTTATGGAGATATTGCATGTCTGAATCTATCCAGCAATGGATTCTCTGCTGCACCAAATGGCACA
GAGTTCTCATTACTGCCTAATTTGACCTACTTGGACCTGTCCTTCAAGGGCTACGTTAAGACTGGGGTGT
TCAGACTGGCAATGTATCTGGCCCACCAGAGACTGCTGGATGAAAATGTGGATGTGATTGTTCTCCTGAT
GCTTGAACCTGTCTTGCAACATTCTCACTTCCTGCGCCTCAGGAGGAGGCTTTGTGAGAAAAGTGTTGTG
GAGTGGCCCAGAACTGCAGCTGCTGAGCCCTGGTTCTGGCAGAACTTGAGAAGTGTTGTAAAAGTGGATA
ATCAAATTATGTACAGCAAAACTTATAAAAAGTTTTTCAGCAGCAAGTAGGGATAAAGATAATAATTCTT
GAGAATAACCTAGTAATTGTTTAAGCATATTAAACATGTCATTATTTGGTATATATGTCATTGTGACTGT
GATACTTAGCAACTTTGATCCTGCACTTTTGAAGTAATAAAGTGGAGATAGTATTGTACATGACCATGTT
TGCATGTGTAAGTTAAAGAGGGTGGCTGTCAAAGGCAATAAGTATTTCATATCAAGAAATAAACAAACAC
GTAATCACTGACCGAGTTTACTTCTCGTTAAAAGTGACCTAAAATTGTCTGTGTATATTTAGTTTTTCCA
TTGTTCCTGTTCTTCAGACTGTCATGATATGAAGACAATTTAAGATGTTACTTTGGCTCATCAAACAGAC
CCACATATTTCAATTTAATTAACTATTTTTAATTTTTACAGGACAAATTCACAACACTTGTTATTATTAG
ACACTTTAGAAATACAAATAGTCCTAGTTATCAAACCACGCCGTCAAATTCTTTTCATTTTTGAAATGGG
ACGTTTTCTGTTCTAGGAAATCAAGAATTGTATTAAGTCATTGACATGCAAAATATCAGACTGGGGTTAT
TAGGTTTGCCATCATCGTGAGTGTTTCAGGGTTGAGTTGTGGAGGCCCATAAGACCCTAATGGTGATGTG
CTACATACCTTGCATCTGCTGGATGCTAGCAGAGACGTTGCTGTACATCATGCAGAGCGGGTCACAGGAG
AACCTGCCTAAGACCTGCACTGAGCTGTGCGCCCATTTCTGCTCCATGAAAGCAGAAGTAGGGGAACCAA
GAGGCAGGGAGCCTGTGAAGTCTGAGCAGCATCATGCGACCAACCGCAAACTGCTGGGCAACCTGGGACG
ACTGGCCTTCTACAGCCTCCTGAAGCACAAGTACACGTTCAGCGAGCCGGACCTCAGGGCCTACGGGATA
GACTCACTGCAAACTCAGTGCAGCCTGGGAGTCGGAGTTCTGATCCGGACCGAGTCCACCATACACACCA
CTTACAGATTCACACACCTGACCCTGCAGGAGTTCCTTGCAGCTACGTTTTATCACGTTTCCTCCAAGCG
GGCCATCTTTGACCTGTTCTCAGAGAGCACCATGTCCTGGCCCAAGATCGGGTTCCAGAACCACTTCAGA
AGCGCCTTCCAACACTCGCAGCAAGCCGAAGACGGCCACCTGGATCTGTTTGTGCGCTTCCTGACGGGCT
TGCTGTGCCCAACTACGATGAAACCTCTGGCTGGGCTCTTGGCTCTCGGGAAAGACGACGGCAGCCAGAA
GGTTTGGGCCGGAGGGTTTTTACAGAGCCTCCTGTCCAGCGTGGGTTCGGTGGTGTCCCTCCGAGCCGTC
AACCTGGCCTACTGCCTGCAAGAGCTGCAACACGGCGAGCTGCTCCGGAGCGTAGAGGAGGACCTGCGGC
AGGGCAGCTTGGCTGGAAAGCTGACGCGGCCGCACTGCGTGGTGCTGGGCTTCCTGCTGCACGTCTCCCC
AGAGTGCAGCGAGCAAACCAACCTGACGGGGTGTCTGAACTACCCAACGGTGAAAAGTTTACTCCCACAG
CTGCTGTACTGCACCCATCTCAGGCTGGAGAATAATAACTTCAAGGACGACGTCATGGAGCTGCTCGGGA
GCCTCCTGAGCGCCAAAGACTGCCACATCCGGAAAATAAGTTTGGCCGACAACGTCATCAGCACCAAAGG
AGCCAAAGCCCTGAGCCGAGCCCTGCTGGTGAACCGAACCCTGACCGCCATCAACTTGAAGAACAACAGC
ATTGGATCAAAAGGGGCAAAGTTTCTGGCAGAGGCTCTGAAAATGAACCAAGCCTTGGTGTCAATCAACC
TGCAGAATAACTCCATCGGGGAAGAAGGAGCTCAGGCCATCGCAGACGTCCTGCAGTCCAACCGCAAGCT
GGTGTCTCTGAACATGCGGAAAAATGCAGTCGGCGCAGGAGGAGCCAAGAGGATCGCAGAGGCGCTGAAG
ACAAACCGAACTCTCACAAAGTTGATACTCTGCAGTAACCAGCTGGGGGACAAAGGAACCGTGGCTCTGG
CTGAAGCTCTGACGTTCAACCACACGCTGCTGTCGCTCCAACTGCAGAGCAACTCGATCAGCAACAAGGG
CATGACGGGCCTGACCAAAGCGCTGAGGCTCAACCGGGGGCTCGTCTCTCTGAATCTGAGAGAGAACTCC
ATCGGGGTGGAGGGAGCCAGGAACATGGCTCAGGCTCTGCAGGAGAACAACTCTCTGCAGAACCTGGATC
TCACAGCTAACCTGCTGCACGACGACGGGGTTCAAGCAATCGCTGGAGTTTCTGGACGGTGATGAATGGA
GAAGTCGCTGGGTGAACTCCAAGCAYAAGTCTGACTATGGAGAGTGGAAACTGACTGCTGGAAACTTCTA
TGGAGATGCTGAAAAAGACAAAGGTCTGCAGACGAGCCAAGACGCCCGCTTCTACGCCGTCTCTGCCAGC
TTCGACCCGTTCAGCAACGAGGGGAAGCCTTTGGTCATTCAGTTTACGGTCAAGCATGAGCAGAAGATCG
ACTGCGGTGGCGGCTACGTGAAGGTCTTCCCCGCTGACTTGGAGCAGACCGAATTGCACGGGGAATCCTC
ATACTACATCATGTTTGGCCCTGACATCTGTGGCTACAGCACCAAGAAAGTCCACGTCATCTTTAACTAC
AAGGGCAAGAATCACCTGATCAAGAAAGAGATTAAGTGCAAGGATGATGAGCTGACCCACCTGTATACAC
TGATCCTGAACCCAGATCAGACCTACGAGGTGAAGATCGACAACGAGAAGGTGGAATCTGGCAGTCTGGA
GGAAGACTGGGACTTCCTGCCTCCCAAGAAAATTAAGGATCCCGAAGCCAAGAAACCAGAAGACTGGGAT
GACCGCCCCAAGATCGACGATGCCGATGACACAAAACCCGAGGACTGGGATAAACCTGAAAACATTCCTG
ATCCTGATGCTAAGAAGCCTGAAGACTGGGATGAAGACATGGATGGAGAATGGGAGCCACCGATGATCCC
CAACCCAGAGTACAAAGGAGAGTGGAAACCCAAACAGATCGACAACCCCAACTACAAGGGAGCCTGGGTC
CATCCTGAAATTGACAATCCCGACTACAGTCCCGACTCCACCATCTACAAGTTTGACAACATCGGCGTTC
TTGGCCTTGACCTTTGGCAGGTGAAATCTGGTACGATCTTTGACAACTTTGTGATCGCCGACGACGTGAA

GGAAGCTGAAGAAATCGGGAAGGAGACGTGGGGCGTGACGAAGGAACCGGAGAAGAAAATGAAGCAAGAG
CAGGATGAGGTGAAACGAAAAGAGGAAGAGGAGAAGAACAAAGAGCAAGCCACTGAGGGCGATGAAGACG
ACGATGATGACGACGAGGTGGAAGAGGACGAAGAGGAGGATGACGATGCAAAGGACGAGACTCTTACAGA
AAATGAAGACGACGAGTCAAAGCCCAAAGATGAGCAAACAAAGCAGAAGGTCCTGGTGATGGAGTATTGT
TCAGGAGGGAGTCTGCTCAACCTGCTCGAGGACCCAGAAAACGCCTTTGGCTTGCCTGAAGCAGAGTTCC
TCACTGTCTTGCAGTGCATAGTGCAGGGTATGAATCATCTGCGGGAATACGGAGTAGTGCATCGGGACAT
CAAGCCGGGGAACATAATGAGGCAGGTTGGAGAGGACGGCAAGTCCGTCTACAAGCTGACAGACTTCGGA
GCGGCGCGAGAGCTGGAAGACGACGAGAAGTTTGTGTCCATTTATGGAACTGAGGAATATCTGCACCCAG
ACATGTATGAACGCGCTGTGCTGCGCAAGCCTCATCAGAAATCCTACGGCGTGAGCGTAGACTTGTGGAG
CATCGGCGTGACCTTGTACCACTCCGCCACAGGGAGTCTCCCTTTCACACCTTTCGAAGGACCCCGTAAA
AACAAACTCACCATGTACAAGATAACCACAGAGAAGCCCATGGGAGCGATAGCCGGCACGCAGCGGGTGG
AAGGCGGTTCTATAGAGTGGAGCTACCACTTACCTTACAGCTGCCAGCTTTCACAGGGTCTGAAAGCCCT
TCTAGTTCCCGTCCTCGCAGGCATAATG

>Cynoglossus semilaevis tlr3-tlr8-nlrc3-calr-ikbke
CCTTCTCTGCGTTAAAGAGCCTGGAATTCCTCGATATCTCCAATAACAACCTGCAATCAGCCAAGCTTGG
CTCCCAGCCTCAGATGCCCAGTCTTTTAAACCTCAACCTTGCGTTCAGCGTGTTCACCACGCTGAAGAAG
GACGACTTCACTTTCCTGCGCCTCTCACCGTTTTTGCAAGTTCTAAATCTCTCATCTGTGCCTTTGAAAA
GTTTGGAGCCTGGTTGTTTTAAACCAATCGCAGGCCTGCATGCTGTCATTATGGATGGAAGCAATATAGG
AAGTCCAGTTATATCCCAACTCTGCTCTGAACTGGGGGAGACGTCTGTCCAAAGTTTGTCTCTTCGGAAG
ATGAAGCTGGTCACACTCAAGAACACAACCCTGTCGGGACTGCAGAAAACCAACCTAACGTTTCTCGACC
TGTCTCAAAATGACATGACAAAAGTTGAAGATGGCTCATTTCAGTGGCTGTCGAAACTTGAGATTCTAAA
GTTGTCCGATAACAACTTCAAGCACCTGAACAAGTTCACTTTTCGAGGCCTCAAAAGTCTCAAGAAACTC
CAACTGACTAAGGCTCTAGTGAAAACACGTTCCACGCCTATTGTTGATGATTTTACTTTTCAACCACTGG
GCGCACTGGAGAGTTTAATTTTACAGAGAACTGCCGTATGGGAAATCACTGAGCACACATTCACCGGATT
AGTAAGTCTTCAAGAGCTTGATATGAGTTGGAGCAGTTATGCGTCACTCAGGATAATTACCAACAAGACC
TTTGTCTCTCTTGCCGGTTCGCCTCTCAGAACCTTAAATTTGACTGGCACAGCCATAACAAGGATCAATC
CTGGAAGCTTTTCACATTTGAGAAACCTTACAGTTCTCCTCTTGGATTTTAATTTCATACAGCAAACATT
CACTGGCTCCGAGTTCGAAGGCCTAACCCGAGTCGAGGAAATGCACATGAGCAATAACCACTGGAAAGTC
AAAGTGAGCTCTACTTCATTCCTTCATGTGCCCAATCTTAGGGTCCTGACATTGGGCAAAAGTCTCAACA
ACTCAGCAGTGAACCTGGATGTGGATCCCTCTCCGTTCAAACCCTTGACCAACCTCACCTTTCTGGATCT
TAGCAACAACAATATTGCAAACTTCAGAGAGAATTTGTTGGAGGGCCTCATAAACCTGGAGGTGCTAAAG
CTCCAACACAACAACTTAGCACGCCTGTGGAAGAGTGCTAACCCAGGTGGTCCTGTGCTGTTTCTCAAAG
GGGCACAGAACCTAGTGACCTTACAGCTGGACAGTAATGGGCTGGATGAGATACCACGCGGGGCTCTAAG
AGGTTTGAGGAACCTGAGTAAACTATACCTGGCAAATAATCTCCTGAACAGTCTGAAAGACTCAATATTT
GACGATCTGGTGGCTCTGCAAGTGTTAAGTTTGCAGAAGAACCTGATCACGACCGTGAAGCCCGAAGTAT
TCAAGGCGCCCACCAGTAATCTCAGTGTACTCTTAATGGATAAAAACCCATTCGACTGCACGTGTGAAAG
CATCCAGTGGTTCGTGACGTGGTTGAACGAGACAAATCTGACCGCTGTGCCTGACCTCAGTGACCAGTAC
ATGTGCAACACTCCACTGGCTTATTTTAACCACTCAATCATGACATTTGACACTTACCCCTGCAAAGACA
TGGTGCCATTTCAGGCTCTTTACATATTCAGCAGCACAGCTGTATTGCTGCTGATGGCAACAGCGTTTTT
AGTGCGCTTCCAGGGCTGGAGAATTCAGTTCTATTGGAACATACTGATCAGTCGGACATTGGGGTTCACA
AACAGAGATGGCAGCAGGGAATTTCAGTATGACGCCTACATCATCCATGCTGAGGACGACACACACTGGG
TGGAGAGGGTAATAGTTCCCTTAGAGAATAATAACTGCAGTTTCTATCTGGAAAGTCGGGACGCCTTACC
TGGGATGCCACAACTTCAAAATATTGTTGACAATATGAGGGAATCCCGGAAAATCTTGTTTGTCGTCTCG
GAACATCTTCTCAACGATCCTATGTGTAGAAGATTTACAGCCCATCATGCACTTCACCAGGTCATCGAAG
AGAGCAGAGACTCTGTGGTTCTGGTGTTCCTGCAGGATGTGCACGACTTTAAGTTATCCAGCTCGCTCTT
CCTCCGCAGGGGGATGTTGCGCTCGCGCTGTATCCTGGAGTGGCCCGGACATAAGGAGCGACTTCAAGCA
TTTCATCAGAAGCTTCTCATAGCACTCGGCCTGACTAATCAATTCAGGATGCTCATCAAGCCAGAGTGCT
ACCAAGCTGGCCGAGTGCTAATCCTCAGCTCAAATAATCTTTTCTTCATTTCTCCAGAGCAGTTCCAAGG
CTATGGAAACATCGCCTGCCTCAATCTGTCTGCAAATGGCTTTTCAGCAGCACTTAACGGCACAGAGTTC
TCTTCCCTGCCCAACCTGACCTACCTGGATCTGTCGTACAACAAGATAGACCTGGTCTACGACAATGCCT
TCAAAGAACTACAGAAACTCGAAGTCCTTGATCTCAGTTACAACTCACACTACTTTAAGTCATACGGGAT
CACGCATAATTTGCATTTCATACAAAATCTGCCGGTTCTGCGAGTGCTCAATATGAGTCACAATAGCATT
TCCACGTTAACGACAAAAATGTTGAAAAGCAACTCCTTGTCAGAACTTCGGTTCACAAATAATGACCTTG
GGTCCTTGTGGAAGGACAAAGAGGTCAGGTACATCCAGCTTTTTGCCTACCTTACTAATCTGACATTGTT
GGACATCTCCAATAACAGGCTAACCAGGATTCCAGACTGTGTTTATCAACTGCTTCCTTCTCAGCTCACC
AAACTATACATACAACACAACAGTCTGGCTGATTTTAAATGGAATGAACTAAAGCATTTACCTCGACTTC
AGATTTTAGACCTGAGCCACAATTCTCTGTCCTTTCTGATTAATTTAAACTCAAACATGAGCCAGACTCT
GACTTTTCTTGACTTAAGCAATAACCAAATCGTCCACCTAGATGAGGGATTGATAAATGGTCTCAAAAGC
CTCAGGGGGCTCAGACTTAGCTACAACAAACTCACTATCATTAACGAGTCTACCTTTGTGAGCGGACCTG
AAAATCCTCTTCAGACTTTGTTCTTGGATGGAAACCCTTTCCAGTGTACCTGTGATTCCTTAGATTTCTT

CCTTTGGATTGGAAAAAGTGACATTAAAATCCCTAGACTGACCTACGAAGTGAAATGTGACTCACCAGCT
AATCAGAAAGGACAAGCACTGATATACTTTGACGTCAACCAGTGCGTAAACGACAGCAAAGCTAGGTTGA
TGTACATCCTCTCCAGCTCCTTCATTATTGCCTTCATGTTTGTCACTACCGTTGCTCACTTGTTTTACTG
GGATGCTTCCTATGTAATTCACTATTTGAAAGCTAAAATGAAGGGCTACAGATCACTGTATTCCTCTGAC
AGCTTCTATGAAGTCTTTGTGACCTACGACACCACAGACCCACATGTCTCTGAGTGGGTGATGAGAAATT
TACGAGTGAAGCTGGAAGACGAAGGAGAGAAACATCTTCCCCACAGCATCCGATACAGCCGCAAGACTCT
TTTCGTCCTGACGAAGGACTACGTCAAGACGGGGGTTTTCAAGCTGGCCATTTATCTGGCACACCAGCGA
CTGCTGGATGAAAATGTGGACGTGATTGTTCTGCTGATGCTGGAACCTGTCCTGCAGCACTCTCACTTCC
TCCGGCTGAGGAGGAGACTCTATGAGAAGAGTGTTATAGAGTGGCCAAAGACAGCAGCTGCACAGCCCTG
GTTCTGGCAAAACCTGAGGAATGTGGTCCGAGTGGATAATCGCTTCGTGTGCAACAAAACATATTCAAAA
TACGGAGGAGCATAAAAATGTCATGGTCATGTGTTACATACCCAGTATTTGCTGGATTGTAGCGGATACT
CTAGTATACATTATGCAGGGTGGAGCACAGGATGGTCTGCCAAGAACTTGCTCTGAGCTGTACGCACATT
TCTGCGCCATGAAAGCAGAAGTCGGAGAACCCAGAGGCAGGGAGCCCGTCAGATCAGAGCAGCTCCATGG
GAACAATCGTAAGCTGCTGTGGAGTCTTGGACGAATGGCATTTTATGGGCTTCTTAAACACAAGTACGCG
TTCAGTGAGCTGGACCTCAGGGCCAACGGAATTGATCCCCTGTTGAGCCAAAGCAGTTTTGGTACAGGTG
TTCTGGTTCGAGAGAAGTCGGCTGTGCTGATGACATACCGATTTATTCATCTGAGCCTGCAGGAGTTTCT
TGCAGCCACTTTTTATCTTGTCTCCTCCAAGCGTGCAATCTTTGACCTATTCTCAGAGAGTGCCATGTCA
TGGCCCAAAATCGGCTTTCAAAACCACTTCAAAAATGCCTTTCAGCACACGCAGCAGGCTGAAGGTGGGC
ACTTGGACGTGTTTGCGCGCTTCCTGACAGGTCTGCTGTGTCCAGTGGCTCTGAGGCCTCTTGCTGGGCT
TCTGTCTCTGGGTAAAGATGATGGTTCACAGAAAACCTGGGCGGCAGGTTATTTACACAGCCTCCTGGTC
AGCGGGGGTGCTGTGGTGTCCCTGCGTACTGTCAACCTGGCATATTGTTTACAGGAGCTGCAGCACACTG
AGCTGCTGCGGAGCGTGGAGGAGGATTTAAGGCTTGGCAGCCTGTCAGGGAAGTTATCGCGGGCTCAGTG
CGTGGTGCTGGGATATCTCCTGCATGTGTCTCCAGAGTGCAGCGAACAGACCAACCTATCAGGCTCTTTG
AACTACTCTACAGTCAAGTGTTTGCTCCCACAGTTGTTCTACTGCAGCCACCTCAGGTTAGAGAACAATC
ACTTCAAAGATGATGTCATGGAGCTGCTGGGAAGTCTGCTCAGTGCCAAGGACTGCCATATTGTGAAGTT
AAGCTTGGCAGAGAATTCCATTGGCAGTAAAGGAGCGAAAGCACTGAGTCGAGCTCTGTTGGTGAATAGA
ACTCTGACGTCGCTCAATCTGCGGAACAACAACATTGGTTCTAAAGGTGCGAGGTTTTTGGCAGAAGCTC
TGAAAATGAACCAAGTTCTGGTGTCGGTCAACTTTCAGAGCAACAACATTGAGGAGGAGGGCGCCCAAGC
CCTCGCAGAAGTGCTGCAGTGCAACCGCAAACTGGTGTCTCTGAATCTCAGCAAGAACACAGTTGGAGGA
GGTGGAGCCAAAAAAGTTGCAGAAGCCCTGAAGATGAACCAAACCCTGACAAAGTTGATCCTTTCCAGCA
ACCACCTCGGGGACAAAGGAACGGTTGCACTCGCAGGGGCTTTGACACTCAACCACAGTCTGCTCTCACT
TGAACTGCAGAGTAACTCCATTAGCAACAGAGGGATGGCAGCCTTAACAAGTGCACTACGGCAGAACCAC
GGCCTTGTCTCCTTGAATTTGCGGGAGAACTCAATCGGAGTGGAAGGTGCGAAGAATATGGCTCGCGCCC
TCCGAGAGAACAGCTCTCTGCAGGACCTCGACCTCACAGCCAACCTGTTGCACGACGAAGGCGTTCAAGC
TATAGCTGAAATTCGACGATGGAGATGCATGGACGAGTCGCTGGGTCCAGTCAAAACACAAGTCCGACTA
TGGCGAATGGAAACTGACTGCTGGAAAGTTCTACGGAGATGCTGAAGCTGATAAAGGTGTCCAGACCAGC
CAGGATGCTCGCTTTTACGCCATGTCATCCCGCTTTGACTCTTTCAGCAATGAGGGGAAGCCCGTGGTCA
TCCAGTTTACTGTCAAACATGAGCAGAAGATCGACTGTGGTGGTGGATACGTCAAGGTGTTCCCCTCTGA
CCTAGATCAGACTGACATGCATGGAGACTCGCAGTATTACATCATGTTTGGCCCTGACATCTGTGGCTAC
AGCACCAAGAAGGTTCATGTCATCTTCAACTACAAGGGTCAGAACCACCTTGTCAAGAAAGACATCAAAT
GCAAGGACGATGAACTGACCCACCTGTACACACTGATTCTAAACCCAGACCAGACCTACGAGGTGAGGAT
CGACAATGAGAAAGTGGAGTCTGGGTCTCTGGAGGATGACTGGGACATGCTGCCTGCAAAAACGATCAAG
GATCCTGAGGCCAAGAAGCCTAGTGACTGGGATGACAGATCCCAGATCGATGACCCCACTGACACCAAAC
CTGAGGACTGGGACAAGCCAGAGACCATTCCAGACCCTGATGCCAAGAAGCCTGATGACTGGGAAGACGA
CATGGACGGAGAATGGGAACCTCCCATGATCACCAACCCTGAATACAAGGGAGAATGGAAACCCAAACTT
ATTGAAAACCCAGATTACAAGGGAGCCTGGGTTCACCCTGAGATTGATAATCCAGAGTACACTCATGATG
CCACAATGTACAAGTTTGACAGCATTGGAGTTCTTGGTCTGGACCTGTGGCAGGTAAAATCTGGAACAAT
CTTCGACAACTTCATAATCACAGATGATGTAAAAGAAGCTGAGGACTTTGGCAAGGAAACATGGGGAGTC
ACAAAGGGACCAGAGAAGAAGATGAAGGAGGAACAGGAGGACATGGAGAGGAAGCTGAGAGAGGAGGAGG
AAAAGAGCAAAAAGAAGGATGATGAAGATGAAAATGATGAGGAAGACGATGAAGATGAGGAGGGTAATGA
GGAAGATGAAGCGGAGCAGGGGGAGGAGGTAGAGGAGGACGCAGGAACAGAAGAGGAGGACACTAAACAC
AAGGATGATCCCTCTAAGCAGAAGGTACTGGTGATGGAATATTGTTCAGGTGGAAGTCTGCTCAGTCTGC
TGGAGGAACCGGAGAATGCCTTCGGCCTCCCTGAGCCTGAGTTCCTCACGGTGCTACAGTGTGTAGTGCA
GGGGATGAACCACCTACGTGAGAACGGAGTGGTTCACAGAGACATCAAGCCGGGCAACATCATGCGTCAG
GTGGGAGAGGACGGCAGGTCTGTTTACAAACTGACAGACTTTGGAGCAGCAAGAGAGCTGGAGGATGATG
AGAAGTTTATGTCCATCTACGGGACTGAGGAATATCTGCATCCAGACATGTATGAACGCGCAGTGTTGCG
TAAACCTCACCAGAAGTCCTATGGAGTGAGCGTTGACTTGTGGAGCATTGGTGTGACATTTTACCATACT
GCCACTGGGAGTCTCCCTTTCACACCCTTCGAAGGACCTCGCAGAAACAAACCTCTGATGTTTAAAATAA
CTACACAGAAACCCACAGATGCCATCGCTGGAATACAGCGGGTGGAGGGTGGACCTATAGACTGGAGCTA
CGACCTGCCTCACAGCTGCCAACTGTCCCTGGGTCTGAAGGCGCTCTTAGTCCCAGTCCTAGCAGGTATC
CTG

>Takifugu rubripes tlr3-tlr8-nlrc3-calr-ikbke
CTTTCTCCGGCCTGCAGAAGCTGACGTATCTGGACGTTTCCTTAAACAACCTGCAGTCGGCCAGGCTGGG
CTCACGGCCTCAGCTGCCCGCTCTGGTCAACCTCAGCCTGGCGCAAAATGGCATCACCGTCCTGAAGAGA
GATGACTTTTCCTTCCTGAATCACTCGTCCTCTCTCCGAGTGCTCAACCTGTCATCGGTGCCCCTGAAAA
CGCTGGAGCCCGGTTGCCTGAAGCCCATTTCGGGCCTGAGTACTTTAATCTTGGACGGGAGCAACATGGG
CGCTCTGGTGATCGCTGGAATCTGCTCGCAGTTGTCCGGGACGGCCATCAGCAGCCTGTCTCTGCGGAAC
ATGAAGCTGGCCTCTCTCCCAAACACCACCTTTGCAGGACTCCAGCAAACCAACCTGAGCTGCCTGGATC
TGTCCCGTAACGGCCTGGGGAAAATCGAAAACGGCTCATTTCAGTGGCTGCCACGACTCGAGACCCTAAT
TTTAATGGAGAACAACATCAAGCATCTGACCCGAGACACGTTTCAGGGTCTCCAGAGTCTGAAAACCCTC
CAGCTGACAAAGGCTCTGGTGAAAGGTCACACCTCTGCCACCCCCATTATTGACAACTTCTCCTTCCAGC
CTTTAACGACCCTGGAGAGTTTGGTATTACAGAAAACTGCAATTCGGGAAATTACAACGCAGACGTTCAC
GGGCTTGACCAGTCTGAAGGAGCTGGACCTGAGCTGGAGTAGCTGTCTCTCACTCAAGACCATTACTAAC
GAGACCTTCCTGTCTCTGGCTGCTTCTCCACTCAGAAAGCTGAATCTGACGGGAGCGGCGGTAGTGCAGA
TCAGTCCTGGAGGCTTCTCCACCTTAAAGAGCCTCACTGTTCTACTCTTGGATTCCAACTTCATCAAACA
AACCCTGACTGGCAGAGAGTTCGAAGGCTTGGGTCAGCTGGAGGAGATTCACATGTCTCTGAACTTCCAG
AAAGTCAATCTAAGCTCCGCCTCTTTTGCTGCTGTGCCCCGACTGAAGGTCCTGACTCTGGGGAAGAGCC
TGACCAGCACGGCCCTGAACGTGGATCCCTCTCCGTTCAGCCCCCTGGTCAATCTCACCTTCCTGGACCT
CAGCAACAACAACATCGCCAACATCAGGAGGACCTTGCTCAAAGGTCTGGTTAACCTCAGGGTGCTGAAG
CTGCAGCACAACAACTTTGCCCGTTTATGGAAGAACAACAATGTGGGTGGGCCGGTAATGTTTCTGCAGG
ACACGCTGAAACTGAAGACCCTACTGATGGACAGCAACGGGCTGGATGAGATCCCAGCTGGGGCTCTGAG
AGGACTAAGAGAGCTGCAGGAGCTGAGCCTGGGCAACAACCTCCTCAACAGCCTGCGGCCGTCTGTCTTC
GATGACCTGATCTCCCTGCGCGCTTTGTTTTTACAGAAGAACGTGATCACGTCAGTGAGGCCCGAAGTGT
TTGAAACGCCCTTGAGCAACCTCAGCCTGCTCGTCATGGGCAAGAATCCCTACGACTGCACGTGCGAGAG
CATCCTGTGGTACGCCACTTGGCTGAACAACACCAACACCACCAGCGTGCCGGACCTGGCGGAGCAGTAC
ACGTGCAACACCCCGCTGACCTACTTCAACCGCTCCATCATGACGTTCGACCCCCTTTCCTGCAAAGACA
TGACCCCCTTCCGGGCCCTGTATGTGGTGAGCAGCACCACCGTGATCTTGCTGCTGACGGTCGCGCTCTT
TATGCGATTCCATGGATGGAGGATTCGGTTCTACTGGAATATCGTGGTCAACCGCACTTTGGGATTCAGC
GACGCCACGGTGGAGGAGGGCCGTCAGTTTGAGTACGACGCCTACGTCATACACGCCGAGGACGACGGCG
GCTGGGTGGAGAGGCGGCTGCTGCCGTTGGAGAATGGGAGGTGCAAGTTTTGCTTGGAGGTTCGAGATTC
AAACCTCGGAATGTCGCAGTTGGAATCCATCGTCAACAACATAAGGAACTCCCGGAAAATCCTGTTCGTT
GTCACCGAAACGCTCCTCATGGATCCCTGGTGTCGACGGTTCACGGCTCATCAGGCTCTGCACCAGGTGA
TCGAAGCCAGCAGGGACTCCGTGGTCCTGGTCTTCCTGCAGGACGTCCACGACTACAAGCTGTCTCGGAC
GCTCTTCCTCCGCAGGGGCATGCTGCGCTCCTGCTGTGTCCTGTACTGGCCCGTCCATAAAGAGAGGGTC
CCAGCGTTCCACCAAAAGCTCCTCATAGCGCTCGGCATGACCAACCGCTTGCAGGACCTTATCAAGCAAG
AGTGCTTCGATGCTGGAAAAGTGCTGAGCCTTAACTCGAATAACTTCCTCTTTATTTCCACAAAGGAGTT
TGAAGGAGATATTAAGTGTCTCAATCTCTCGAGAAATGGATTCGCACCAGCGCTCAATGGGACAGAGTTT
TCCTTCCTGCCTAATTTGACGTACTTAGATCTGTCATTCAATAAAATTGATCTGGCCTACAGCTTGGCAT
TCAACGACCTAAAGAAGCTACAAGTGCTGGACCTCAGTTACAATCCTCACTACTTTAATGTACAAGGGAT
AACCCATAAAGTTAACTTCTTAAGAAACCTGCCGGTCCTCAGGGTGCTGAATATGAGCCACAATGACATT
TCCACACTGACTACAAAATACATGGAAAGCAAGTCTTTAGCGGAGCTTCGCTTCACACACAATTATCTTG
GGACTCTTTGGAAAGAAAATGATCTTTCCTACAAGAAACTTTTTACTAAACTTACTAATTTGACCATTTT
AGACATATCCTTCAACCAAATTATAAAGATCCCTGATGAGATGTATAAACATTTGCCACAGAACCTCACC
ACACTGATCATAAGTCACAACTTCCTGACTGATTTTAAATGGAACAAGCTTATATTTTTACCACAGATTA
AAGTTTTAGACCTCAGCTTCAACCGTTTGACCAACGTGACAGGTATACACATTGCCCACACCCTGACACT
CCTCAACCTCAAACATAATGGCATTTCTCACTTGGATGATGGATTTCTAATGGGTGCAAAAAGACTTCAG
GTTCTCAATCTCAAATCCAACCAACTCACCACCATTAACGAATCCACGTTCCAGCCCAGACCTGAGAATC
AGTTCCAGACTTTGTACCTGGAGGAAAACCCCTTTCAGTGTACATGTGATTTACTAGATTTCATCTTATG
GATCGAAAACAGTGACGTAAAAATCCCCGGGCTCGCCACGGATGTGAAATGTGATGCACCTGCAAATCAA
AAGGGTCGTGTTCTGATTAAGTTTGACATTACTCAGTGTGTAAACAACAGTGAGGCATTCTTGATCTACA
TCCTTACCAGTTCCTTTGTTATTGGTTTTATGCTTGTGACCACTGTGGCCCATCTTTTTTACTGGGATGC
CACCTATGTCCTACACTATATGAAAGCTAAGTTAAAGGGATACAGCTCTTTGAACTCATCAGAGATTTTG
TATGGTGCTTTTGTGACCTACGACACCAGAGATCCACATGTCTCTGAATGGGTGATGAAGAACCTTCTGG
TGAAACTAGAGGAGGAAGGAGAGAAGAACCTTCCTCTGTGTCTGGAGGAGAGGGACTGGACCCCCGGAGT
ACCGCTGGTAGACAACCTCACTCAGAGCATCCGATACAGTCGGAAGACCCTATTTGTCCTGACGCAGGAT
TACGTCAAGACCGGGATTTTCAAGATGGCCATGTATCTGGCACACCAGAGACTGCTTGATGAAAATGTGG
ATGTGATTGTTTTGCTGTTGCTGGAACCTGTTCTGCAGCATTCTCACTTCCTTCGTCTGCGACGGAGACT
ATGCGGTGAAAGTGTAGTGGACTGGCCGAGAACAGCTGCCGCAGAACCCTGGTTTTGGCAAAACCTGAGG
AATGTGGTCAGGGTGGAAAATCAGGTTATGTACAACAAAAATTATTCAAAGTACGGAGTCTCATAAAATG
TTAATGGTGATGTGCTCCATTCCATGTATTTGCTGGATACTAGCTGAAACTCTGACGTATATCATACAAA
GTGAAACACACGAGAGCCTACCAAAGACCTGCACTGAGCTGTATGCTCAATTTTGCTCAATGAAAGCAGA

AATAGGTGAACCAAGAGGCAGAGAGCCACCGAAAATGGAGCACGGGAGCAATCGTAAACTGCTGGGGAAT
CTTGGACGTCTAGCATTTTATGGGCTCCTCAAACGCAAGTACACCTTCAGTGAGCAGGATCTCAAAGCCT
ACGGCATAGATTCGCTCTTAACTCAGTGTAGCCTTGGTGCTGGAGTTGTTGTCCGGGAGGAGTCATTCAT
ACACACAACATACCGGTTTACTCATTTGACTATCCAGGAGTTTCTTGCGGCTACTTTCTACCATGCTTCC
TCAAAGCGGGCTATTTTTGATCTATTCTCGGAAAACACAATGTCTTGGCCCAAGATTGGTTTCCAGAACC
ACTTCAAATGTGCCTTTCAGCACTCCCAGCAGGCTGAAGATGGCCACTTAGACGTGTTTGTGCGCTTTCT
AGCGGGCCTTCTGTCACCAGTAACGCTGAAAACTCTGGCTGGGCTTCTGGCTGTTGGAAAAGATGACGGA
AATCACAAGACATGGGCATCGGGGTTCCTACAAGGCATTTTGGGCAGTGGAGGTGCAGTTGTGTCTCTTC
GTACAGTCAATCTGGCATATTGTTTACAAGAATTGCAACATTCTGAGCTGTTGCGCAGCGTGGAGGAGGA
CCTACGCCTCGGTAGCCTCACAGGGAAGTTGACACGAGCTCACTGTGTTGTGTTGGGCTACCTGCTGCAT
GTGTCTGCTGAGTGCAGTAAGGAGACCAACCTCCCAGGGTGTCTGAACTACTCAACTGTGAAGTGTTTGC
TGCCCCAGCTGCTCTATTGTAGCCACCTGAGGTTGGAGAGTAATAATTTCAAAGATGATGTCATGGAGCT
GCTGGGGAGCCTCTTGAGTGCTAAAGACTGCCAAATCCAGAAGATGAGTTTGGCAGAGAATGCGATCAGC
AACAAAGGAGCCAAAGCCCTGAGTCGAGCCCTCTTGGTCAACCGCACATTGACTTCACTCAATCTCCGGA
ATAACAACATCGGCTCTAAAGGTGCAAGGTTTCTGGCAGAGGCTCTAAAAATGAACCAAGCCCTGGTGTC
ACTGAACTTGCAGAGCAATGGCATTGACGAGGCTGGTGCCGAAGCCCTGGCAGAGGTACTGCAGTGTAAC
CGCAAGCTGGTGACTCTGAATATGCAGAAGAACATAGTCGGTGCAGGAGGAGCCAAAAGGATTGCAGATG
CCCTGAAGACAAACAAGACTCTCACAGAGTTGATGATTTGTAGCAACCAGCTGGGTGACAAAGGAACGGC
GGCTCTGGCGGAAGCATTGACAGTCAACCACACCCTGCTTTCCCTTCAACTTCAGAGCAACTCGATCAGC
AATCGAGGGATGACGGCTTTAACCAAAGCACTGAGTCTGAACCGTGGGCTCGTCTCACTGAATTTGAGGG
AGAACTCCATAGGTGTGGAGGGGGCCAGGAACATGGCCAAAGCACTCCAGAAAAACAGCTCTCTACAAGA
CCTTGATCTCACAGCTGACCTGCTGCATGATGACGGAGTTCAAGCTATCGCTGGAGTTTCTGGACGGTGA
TGCTTGGGAAAGTCGCTGGGTAAACTCCAAACACAAATCTGACTATGGGGTGTTGAAACTCACTGCTGGT
AACTTCTATGGAGATGCTGAGAAAGACAAAGGTCTTCAGACAAGCCAGGATGCTCGTTTTTATGCTACGT
CTGCTCGTTTTGAACCATTCAGCAATGAGGGCAAGCCCGTGGTAATTCAGTTTACTGTCAAACACGAGCA
GAAGATCGACTGCGGCGGTGGCTACGTCAAGGTTTTCCCATCAGACTTGGATCAGACTGATATGCATGGA
GACTCTCAATACTACATTATGTTTGGTCCGGATATATGCGGCTACAGCACCAAGAAAGTCCATGTTATCT
TTAATTACAAAGGCAAGAATCATCTCATCAAGAAGGAAATTAAATGCAAGGATGATGAACTCACCCACCT
TTACACACTGATCCTGAATCCAGATCAAACTTATATTGTTAAGATTGACAATGAGAAAGTTGAGTCTGGC
AGTCTGGAAGAGGACTGGGACTTTCTGCCCTCAAAGAAAATTAAGGACCCTGAAGCCAAGAAGCCAGAGG
ACTGGGACGATCGCGCCAAGATTGACGATGCTGATGACACCAAGCCTGAGGACTGGGACAAACCTGAAAA
CATTCCCGACCCTGATGCCAAAAAGCCTGATGACTGGGATGAGGATATGGATGGAGAGTGGGAGCCACCT
ATGATCCCTAATCCAGAATACAAGGGCGAGTGGAAACCCAAGCAAATTGACAACCCCAACTACAAAGGAC
CCTGGGTGCACCCCGAGATCGATAACCCCGAATACAGCGCCGATTCCACCATCTACAAGTTCGACAGCGT
TTCCGTTCTCGGTCTGGATCTGTGGCAGGTGAAATCTGGCACCATCTTTGACAACTTCCTGATCACAGAT
GATGTGAAGGAAGCAGAGGACATTGGAAACGAGACATGGGGTGTGACAAAGGCTGAACCAGAGAAGAAAA
TGAAACAGGAGCAAGATGACCTGAAACGCAAGGAAGAGGAAGAAAAGAACAAAGAACAGGGCACCGAGGC
AGATGATGATGATGACGAAGATGATGACGACGACGAAGGAGATGAGGACCTCGACGAAGAACCAAAGGAA
GAAGTAGACGACTCGCTCTCGGAAACAGAAGAGGAGGCCCTGGACCCCAAAGATGATCCGTCCAAGCAGA
AGATCCTGGTGATGGAATACTGTTCAGGTGGAAGTCTGCTCACTGTACTGGAGGAGCCAGAAAATGCCTT
TGGCCTTCCTGAAACAGAGTTCCTCACAGTTTTACAGTGTGTCGTCCGGGGGATGAACCACCTCCGTGAA
AATGGAGTGGTGCACAGGGACATTAAGCCGGGCAACATCATGCGGCAAGCTGGGGAAGACGGCAAGTCCG
TCTATAAGCTGACTGACTTTGGTGCGGCGAGAGAGCTGGAAGATGATGAAAAGTTCATTTCCATCTATGG
AACTGAAGAATATCTGCATCCAGACATGTACGAACGCGCCGTGTTGCGTAAGCCTCATCAGAAATCCTAC
GGAGTGAGCGTGGACCTGTGGAGTATCGGCGTGACGTTTTACCACGCTGCCACCGGGAGTCTCCCCTTCA
TACCGTATGGAGGACCCAGAAAGAACAAACTGACCATGTTTAAAATAACAACGGAGAAGCCAACGGGCGC
CATTTCTGGATTACAGCGCGTTGCAGACGGATCCATAGAGTGGAGCTACAGCCTCCCCCACAGCTGCCAA
CTGTCACAGGGCTTGAAAGTGCTGCTGGTTCCAGTGCTCGCTGGTATAATG

>Larimichthys crocea tlr3-tlr8-nlrc3-calr-ikbke
CCTTCTCTGCACTACAGAGCCTGAAATTTCTTGATGTTTCCATAAACAAACTAAAATCAGCCAAGCTCGG
CACTCAACCTCAGTTGCCCAACCTGGTGAACCTCAATCTGGCATCCAATGACTTCACCACTCTGAAGAAA
GATGACTTTTCGTTCCTTAACCATTCGACCAATCTACAAGTCCTCAACCTGTCATCGGTGCCTCTAAAAA
TATTGGAGCCTGGTTGCTTTAAGCCCATTTCAAGACTACATACTTTAATCATGGATAAGAGCAACATGGG
CACCCTGGTTATCGCTAAACTCTGCAAAGAGTTGTCAGGGACAGCCATTGATGCCCTGTCTCTTCGGAAC
ATGAATCTTGTCACCCTCGCAAACACAACCTTTGCAGGGCTACAGAATACATCTCTAACCTTTCTGGATC
TGTCCCATAATGACATGGTTAAAATTGAAGAAGGTTCATTTCAGTGGTTGACCAGACTTCAGACTCTGAT
TTTGGAGGACAATAAAATCAAGCATCTGACCAAGGACACATTTCAGGGCCTCAAAAGTTTGAAAAAACTC
CAACTGACAAAAGCTGTGGGGAAAAGTCATACCTCTGCTAAAATTGATGATTTCTCCTTTCAACCATTAA
GTGCCCTGGAGAGTTTGATATTACAGAACAGTAGAGTTCAGGATATCACAAAACACACATTTACAGGTTT
GACAAGTCTTAAAGAACTTGATGTGAGCTGGAGTACCTCACTCAAAAACATCAACAACGAGACCTTAGCT

TCACTAGCAGGATCACCTCTCAGAACGCTTAATTTGACAGGAACAGCTATAGCACAAATTAGTCCTGGGG
CCTTCTCCGTTTTCAGAAACCTCACTACTCTTTTTCTAAATCACAACTTCATCAGCCAAATCTTCACTGG
CAAAGAATTTGAGGGACTGGACCAAGTTCGAGAGCTTTACATGGGCAATAACTACATGAAAGTCAATCTG
AGCTCTACATCCTTTGTTAATGTGCCCAATCTTAGGGTCCTGACTTTAGAAAAAAGCCTTACAGTCAAAG
CTTTGGACTTGCATCCATCTCCATTCAAACCCCTGCCCAACCTCACCATCCTGGATCTCAGCAACAACAA
CATTGCTAACATCAGAGAGGATTTGTTGGAGGGGCTTGTGAACCTGAAGGTGCTGAAGCTCCAACACAAT
AACTTAGCCCGTTTGTGGAAGAATGCCAACCTAGGTGGTCCAGTGTTGTTTCTCAAAGGAGCACCAAACT
TGAAAACCTTACTGATGGACAGTAACGGACTGGATGAAATCCCAGCAGGGGGTCTGAGAGGGCTGCATGA
CCTCCGTGAACTAAGCCTGGGTTCCAATCTTCTCAATAATCTCAAGGACTCAGTGTTTGATGATCTGAAC
TCACTGGAGGTTTTATTTTTAGAGAAGAATACGATCACAGCTGTGAGGCCCGAAGTGTTCAAAACTCCCA
TGAGCAACCTTAGCCTGCTCATCATGGGCAAAAATCCATTTGATTGCACATGTGAGAGCATCCTTTGGTT
TGTAACATGGTTGAATAACACAAATATGACCAATGTGCCAGGTCTCAGGGAACAGTATATGTGCAACACT
CCGCTAGCTTACTTTAACCACTCCATCATGGATTTTGACGGCCTCTCGTGCAAAGATATGACCCCATTTC
AGGCTCTTTACATACTGAGCAGCACAGCTGTTATCATGCTTATTGTTACCGCACTTCTGGTACGGTTCCA
TGGCTGGAGGATTCAGTTTTATTGTAACATACTGATCAATCGCACATTAGGATTTAGTGACGCCAAAGCT
GAAGAGGGTAGGCTATTTAAGTATGATGCTTACGTCATACATGCAGAGGAAGATGCCATTTGGGTGGAGA
GAAGTATGACCCCCTTAGAGAAATCTGAAAATTGCAAGTTTTGTTTGGAGGATCGAGATTCAGTCGCTGG
CATGTCACAGCTTGAATCCATTGTGGATAATATGAGGAGGTCTCGAAAGATCTTGTTTGTCATCACTGAA
AGTCTTCTCAGAGATCCCTGGTGTAGACGATTTACAGCCCATCATGCACTTCACCAGGTCATTGAAGCTA
GCAGGGACTCTGTGGTTCTGGTTTTCCTGCAGGATGTTCACGACTACAAGTTATCCCGTTCACTCTTCCT
ACGCAGGGGCATGTTACGTCCATGCTGCATCCTGGACTGGCCTATTCACAAGGAGAGGATACCGGCCTTT
CGCCAGAAGCTCCTCATAGCACTTGGCATGACTAATCGATTGCAGGAGTTTGTCAAGGCAGAATGCTTCA
ACGCCGGACGAGTGCTAATCCTCAGCTCAAATAATCTCTTCTTCATTTCTCTGAAGCAATTTGACGGCTA
TGGCGATATTGCATGTCTTAACCTCTCAGGAAATGGATTTAGCTCAGCACTTAACGGAACAGAGTTCTCC
TTGCTGCCTAATCTGACATACCTGGACCTGTCATGCAATAAGATCGATTTGGCCTATGACCACGCCTTCA
CAGAACTAAAGAAACTAGAAGTACTAGACCTCAGTCACAATTCTCACTACTTTGAAGCGTTTGGGGTAAC
CAATAATTTAAATTTTACAAAAAATCTGCCCGTCCTGAGAGTGCTGAATATGAGTCATAACTCCATTTCC
AGATTAACGACAAAACAGATGTACAGCAAATCATTAAACGAGCTTCGGTTTTCGAATAATAAGCTCGGGA
TTCTTTGGAAAGAAAGAGATGGCTCATATAAGATGCTTTTTACCAATCTTACTAATTTGACCATTTTAGA
TATATCCTACAACGGCATCACAAAGATCCCGGACAACATTTATGAATATTTGCCACGCAATCTTACCAAA
CTATACATAAATCACAACTCCCTTACTGACTTTGAATGGAACTATCTGAAGTTTTTCCATCAACTTCAAA
CTTTAGACCTGAGCTACAATAAGTTACGTCATGTGAGAGGTATAAACTCAAACTTCACCCACACTAATCT
GACATTCCTTGACCTGAGTCATAATAATATTTTCCACTTGGACAATGGACTTTTTAAGCATTTAGAAAAC
CTTAAGGCTCTTAGCCTTAGCTACAACAAACTGACCATCATCAATCAATCCACCTTCCAATTGGGACCAG
AGAATCAGATCAAGACTTTGTACTTGCAGAGAAACCCGTTCCAGTGTACCTGCGATTCACTAGATTTCAT
TTTGTGGATTGAAGACAGTGATATAAAGATCCCTCGACTGACCACTGAAGTGATATGTGTCACACCAGAA
AACCAGAGGGGTCAAGCTCTGATAGGTTTTGACATTAACCAGTGTGTAAATGACAGCGAGGCGTTGTTGA
TCTACATTGTCACACATGCCTGCATTGGTATTTTTATGTTTGTTACAACTGTTGCTCACTTGTTTTACTG
GGACGCCTCCTATGTCCTGCACTATATGAAAGCTAAGATGAAGGGATACAGCCCCTTGAGCTCGCCAGAC
AGTGCTTATGATGTCTTTGTGACTTACGACACCACAGACCCGCATGTTTCTGAGTGGGTGATGAGGAATC
TGCGAGTGAAACTAGAAGAGGAAGGAGAGAAGCATCTTCCTCTGTGCCTGGAAGAGAGGGATTGGCCTCC
TGGAGTCCCGTTGGTGGATAACCTCACTCAGAGCATCCGATACAGCCACAAGACGCTGTTTGTCTTAACG
GAGGGCTACGTCAAGACCGGGGTTTTCAAGCTGGCGATGTATCTGGCCCACCAAAGACTGCTGGATGAAA
ACGTGGATGTGATCGTGCTGCTGATGTTGGAGCCCGTGCTGCAGCACTCTCACTTCCTGCGCCTGAGGAG
GAGGCTGTGTGGGAAAAGTGTCGTGGAGTGGCCGAGAACGGCGGCCGCAGAGCCCTGGTTTTGGCAAAAC
CTGAGGAATGTGGTCAGAGTGGACAATCAGGCCATGTACAACAAGGCTTATTCAAAGTACGGAGTCCAAT
AATATCTTCATGGTGATGTGCTACATACCATGCATTTGCTGGATAGTAGCAGATACCCTGATGTACATCA
TGCAGAGTGAAACACCAGAGAGCCTTCCAAGGACTTGTACTGAGCTGTACGCTCACTTTTGCTCCATGAA
AGCAGAAGTAGGTGAACCAAGAGGCAGGGAGCCTGTAAAAATGGAGCAGCTCCATGGGAACAACCGCAAA
CTGCTGGGGAATCTTGGACGACTGGCGTTTTATGGGCTCCTCAAACACAAGTACACCTTCAGTGAGCAGG
ACCTCAGGGCCTATGGGATAGATCTGCTGTTAACTCAGTGCAGCCTTGGTTCTGGAGTTCTTGTTCGGGA
GGAGTCGAGCATATACACAACATACCGGTTCACTCATTTGACTCTCCAGGAGTTCCTTGCAGCTACTTTC
TACCATGTATCCTCCAAGCGGGCCATCTTTGACCTATTCTCAGAGAGCACCATGTCTTGGCCCAAGATCG
GTTTCCAGAACCACTTTAGAAGTGCCTTTCAGCATTCTCAACAGGCTGAAGATGGTCACTTGGATGTGTT
TGTGCGCTTCCTGACAGGCCTGCTGTCCTCAGTGGTACTGAAACCTCTCGCTGGGCTTCTTGCTCTCGGG
AAAGAAGATGGCAATCAGAAGGCTTGGGCAGCAGGGTTTTTACAAAGCCTCTTGGTCAGCGGGGGTGCTG
TTGTGTCCCTGCGTGCGGTCAACCTGGCTTACTGTTTGCAGGAGCTGCAACACACAGAGCTACTGAGGAG
CGTGGAAGAAGATTTACAGCTTGGTAACCTAGCAGGGAAGTTAACACGGGCTCATTGTGTTGTGCTGGGC
TACCTGCTGCATGTGTCTCCAGATTGCAGTGAACGGACAAACCTAACAGGCTCTCTGACCTACACAACAG
TGAAATGTTTGCTTCCACAGTTGCTATACTGCAGCCATCTCAGGTTAGAGAATAATCACTTCAAAGATGA
TGTCATGGAATTGCTGGGAAGCCTTCTGAGCGCCAAAGACTGCCATATCCAAAAGATGAGTTTGGCAGAC

AATGCCATCAGCAACAAAGGTGCCAAAGCCCTGAGTCGAGCCCTCTTAGTGAACCGGACGCTAACATCTC
TCAATCTTCGGAACAACAAAATCGGCTCTAAAGGTGCAAAGTTCCTGGCAGAGGCTCTGAAAATGAACCA
AGTCCTGGTGTCAGTAAACTTCCAGAACAATGCTATTGAGGAGGAAGGTGCTCAGGCCATCGCAGAAATA
CTGCAGTGCAACCGCAAACTGGTGTCCCTGAATGTACAGAAGAACACAATTGGAGCAGGAGGAGCCAAAA
GGATTGCAGATGCGCTCAAGACAAACCGGACTCTCACAAAACTGATTCTTTGTAGTAACCAGCTCGGGGA
CAAAGGAACAATCGCTCTGGCGGAGGCTTTGACAGTTAACCACACTCTGCTCTCGCTACAACTTCAGAGT
AACTCAATTAGCAACAAAGGGATGACAGCCTTAACCAAAGCACTAAGGTTGAACCGTGGCCTTGTGTCCT
TGAATTTAAGGGAGAACTCCATCGGTGTGGAAGGAGCGAAGAACATGGCCCACGCCCTCCATGAAAACAA
CTCTCTACAAGAGCTGGATCTCACAGCTAACCTGCTGCATGATGAAGGGGTTCAAGCTATTGCTGGAGTT
TCTGGACGGTGATGAGTGGAGAAGTCGCTGGATAAACTCCAAACACAAATCTGACTACGGAGAGTGGAAG
CTGACGGCTGGAAACTTTTATGGAGATGCCGAGAAAGACAAAGGTCTGCAGACAAGCCAGGATGCTCGTT
TCTATGCTGCCTCTGCCCGCTTTGACCCTTTCAGCAACGAGGGCAAGTCTCTGGTCATTCAGTTTACAGT
CAAGCATGAGCAAAAGATCGACTGTGGCGGCGGCTACGTGAAGGTCTTCCCCGCTGACTTGGAACAGACC
GAGATGCATGGAGAATCCTCATACTACATAATGTTTGGCCCTGATATCTGTGGATACAGCACCAAGAAAG
TCCACGTCATCTTCAATTACAAGGGCAAGAATCACCTCATCAAGAAAGAAATTAAGTGCAAGGATGATGA
GCTGACCCACCTGTACACACTCATCCTGAATCCAGATCAGACCTATGAGGTGAAGATTGACAACGAGAAG
GTAGAATCTGGCAGTCTGGAGGAAGACTGGGACTTCCTGCCCCCAAAGAAAATTAAGGACCCCGAAGCCA
AGAAGCCAGAGGACTGGGACGATCGTGCCAAGATTGATGATGCTGACGACACCAAGCCAGAGGACTGGGA
CAAAGCTGAAAACATCCCAGACCCTGATGCCAAAAAGCCTGAAGATTGGGATGAGGATATGGACGGAGAG
TGGGAGCCACCCATGATCCCCAACCCAGAGTACAAGGGTGAATGGAAACCAAAGCAGATCGACAACCCCG
ACTACAAAGGATCTTGGGTGCATCCTGAGATCGACAATCCTGAATACAGCCCTGATTCAAACATTTACAA
GTTTGACAACATTGGTGTCCTTGGTCTTGATCTCTGGCAGGTGAAATCGGGTACCATCTTCGATAACTTT
GTAATCACAGACGATGTGAAGGAAGCAGATGACATTGCAAAGGAAACATGGGGTGTGACAAAGGAACCAG
AGAGAAAAATGAAACAAGAGATAGATGACCTGAAACGAAAAGAAGAGGAGGAGAAGACCAAAGAACAAGA
TACTGAAGCTGCTGATGAGGAGGACGAGGAAGAAGAGGAGGAGGATGAAGACGCAGATGAGGAAGAGCCA
AAGGATGAGATAGAGGAGGCACTTTCAGAAATGGAGGAGGAGGACGTAAACCAGAAAGATGAGCCCTCCA
AACAAAAGGTGCTGGTCATGGAGTATTGTTCAGGAGGAAGTCTGCTCGGCCTGCTCGAGGAGCCAGAAAA
TGCCTTTGGCCTGCCTGAAACAGAGTTCCTCACAGTTTTACAGTGTGTAGTTCAGGGGATGAACCACCTG
CGGGAAAACGGAGTGGTGCACCGGGACATTAAGCCAGGCAACATCATGAGGCAGGTCGGGGAGGACGGCA
AGTCTGTTTATAAGCTGACCGACTTTGGAGCGGCAAGAGAGCTGGAAGATGACGAGAAGTTTCTGTCTAT
CTATGGAACGGAAGAGTATCTGCATCCAGACATGTATGAGCGTGCCGTGCTGCGGAAGCCTCATCAGAAA
TCCTATGGCGTGAGCGTAGACCTGTGGAGTATCGGTGTGACGTTTTACCACGCTGCCACTGGGAGTCTTC
CCTTCATACCGTACGGAGGACCTCGCAGGAACAAGCCCACCATGTACAAAATAACGACAGAGAAGCCTAT
GGGGGCAATATCTGGAATACAGCGGGTGCTGGATGGACAGATAGAGTGGAGCTACCACCTACCTCACAGC
TGCCAGCTCTCACAGGGTCTGACCGTGCAGCTGGTTCCAGTACTTGCAGGTATACTG

>Lates calcarifer tlr3-tlr8-nlrc3-calr-ikbke
CCTTCTCTGGACTACAGAACCTGCAATTTCTTGATGTTTCCATGAACAAACTGCTGTCAGCCAAGCTTGG
CTCTCAGCCTCAGCTGCCCAGCCTAGTGAACCTCAATCTGGGATTCAATGACTTCACTGCTCTGAGGAAA
GATGACTTTTCTTTCCTTGGCCATTCATCCTCCCTGCAAGTTCTCAGTCTGTCATCTGTGCCTCTAAAAA
CATTGGAACCTGGTTGCTTTAAGCCCATTTCAGGCCTACATACTTTAATCATGGATGGGAGCAAAATGGG
CACTCAAGATATTTCCAAAATCTGCTTAGAGCTGTCAGGGACATCCATTGATGCCCTGTCTCTTCGGAAT
ATGAAGCTGGTCACACTCACAAACAAGACATTTGCAGGGCTGCAGATGACAAATCTAACATTTCTTGATC
TGTCCCATAACGGAATGGGTAAAATTGAGGAGGGCTCATTTAAGTGGTTGTCAAAACTTCAGACTCTAAC
TCTGGCCAACAACAACATCAAGCACCTGACCAAGGACACATTTCAGGGGCTCAAAAGTTTGAAAAAACTT
CAACTGACAAAAGCTCTGGTGAAAAGTCATACCTCCTCTACACCGATTATCGATGATTTTTCTTTTCAAC
CTCTAAGTGCTCTGGAGATTTTAATGCTAAATGAAACTGCAGTTCGGGAAATCACGGAGCACACCTTTAC
AGGCTTGAAAAATCTTCAAGAACTTGATATGAGCTGGAGTAGTTGTGCATCATTCAGAATCATCACCAAC
AAGACCTTTGTCTCACTTGCAGGGTCACCTCTCAGAATACTAAATCTGACAGCAACAGCTATAACCCAGA
TTAATCCTGGAAGCTTCTCCCTTTTGAGAAACCTCACCACTCTTCTACTAGATTATAATTTTATAGACCA
GATTCTCACTGGCAAAGAGTTTGAAGGCATGGGTCAGGTTGAAGAGATTCACATGACCAATAACCACCAG
AAAGTCAGACTAAGCACAACTTCATTTGTTAATGTGCCCAATCTTAGGGTCCTGACTTTGGGAAAAAGTC
TTAACACCACAGCGGAGGGCCTGGATGTGGATCCCTCTCCATTCAAGCCCCTGCTCAACCTCACCTTCCT
GGATCTCAGTAACAACAACATTGCTAACATCAGAGAGAGTATGTTGGAGGGACTTGTGAACCTGAAGGTG
CTGAAGCTCCAACACAATAATTTAGCCCGGTTGTGGAAGAGTGTCAACCCAGGTGGGCCAGTGTTGTTCC
TCAAAGGGGCACAGAACTTGATGTCCTTACAGATGGATAGTAATGGGCTGGATGAAATTCCAGACGGAGC
TCTGAGAGGTTTGAGTAACCTCAGTCAACTAAGCCTTGGATACAATCTCCTAAATAGTCTTAAGGGCTCA
GTTTTCAATGATCTGAAATCATTGCAGGTTTTACGTTTACAGAAGAATCTGATCACAACTGTGAGGCCTG
AAGTGTTCAAAACTGTCATGAGCAACCTCAGCCTACTTGTCATGGACAAAAATCCATTTGACTGCACATG
TGAGAGCATCCTGTGGTTTGTGACATGGTTGAATAATACAAATATGACCACTGTGCCAGATCTCAGGGAC
CAGTATAGGTGCAACACTCCACTTGCTTACTTTAACCGCTCAATCATGGATTTTGACACCCTCTCTTGCA

AAGATATGACCCCATTTCAGGCTCTTTACATACTGAGCAGCACAGCTGTTATGATGCTGATTGCAACAGC
TCTTCTGGTACGGTTCCAGGGCTGGAGAATTCAATTTTATTGGAACATACTCATCAATCGTACATTAGGA
TTTAGTGATGCCAAAGTTGAAGAGGGCAGGGAATTCACGTATGATGCTTATATCATACATGCAGAGGAGG
ACAGCAGCTGGGTGGAGAGGATGGTGATCCCCTTAGAGAATGAAAAGTGCACATTTTATTTGGAGGATCG
AGATTCAGTCCCTGGGATGTCACAGCTTGAATCTATTGTGGATAATATGAGAAAGTCCAGGAAAATCTTG
TTTGTCATCACTGAAAGTCTTCTCAATGATCCCTGGTGTAGACGATTTAAAGCCCATCACGCACTTCACC
AGGTCATTGAAGCCAGCAGGGACTCTGTGGTTCTGGTCTTCTTGCAGGATGTGCACGACTACAAGTTATC
TCGCTCACTCTTCATCCGCAGGGGCATGTTGCGCAAGCGTTGCATTTTGGAATGGCACGGCCACAAAGAG
AGGGTGCCAGCCTTTCACCAGGAGCTCCTCATAGCACTTGGCATGACTAATCGATTGAGGGATCTTATCA
AGCAAGAGTGCTTTGACTCTGGTCGAGTGCTAATCCTCAGCTCAAATAATCTGTTCTTCATTTCTCCAAA
ACAATTTGAGGGCTATGGAAATATTGCATGTCTCAACCTCTCAGGAAACGGATTTTCGGCAGCACTTAAT
GGCACAGAGTTTTCTTCACTGCCTAATCTGACCTACCTGGACCTGTCATTCAATAAAATAGATCTTGCAT
ATGACAATGCCTTCATAGAACTACAGAAACTAAAAGTACTAGACCTCAGTTATAATCCGCACTACTTCAA
ATCAACGGGGGTAACACATAATTTGAATTTCATGAAAAATCTGCCTGCTCTGAGAGTGCTGAATATGAGT
CATAACGCAATTTCCATCTTAACAACAAAACAGATGCACAGCAAATCATTATCTGAACTTCAGTTTACAG
ATAATAATCTTGGGACTCTTTGGAAAGAGAGAGATGGCTCATATGAGATGCTTTTTACTAATCTTACTAA
TCTGACAATTTTAGATATATCCCAAAACAGAATAACAAAGATTCCAGATAATGTTTATGAACGTTTGCCA
CATAATCTCACCAAACTACGCATAAGCGGTAACTCCCTCACTGATTTTAAATGGAACAGACTGAAGTGTT
TTCATCAACTTCAAATTTTAGACCTGAGTTTTAATTCTTTATCTCATGTGAAAGGTATAAACTCAAACAT
CACCCAAAGTTTGACTTTCCTTGATCTGAGTCATAACCACATTTTCCACTTGGATGATGGATTTATAAAG
GGTCCGAAAAGCCTTACAACTCTTAGCCTTAGCAACAATAAACTGACCACAATCAATCAATCTACCCTCC
TGTCAATATCAAATAATCAGATTAAGACTTTGTTCTTGCAGCAAAACCCATTCCAGTGTACCTGTTATTC
ATTTGAGTTCATTCTGTGGATTGAAAATAGTAATGTAAAGATCCCGAGACTGACCACTAAGGTGAAATGT
GCCACACCAGAAAACCAGAAGGGTAGAGCACTAATATACTTTGACATTAACCAGTGTGTTAATGACAGTC
AGGCATTCCAGATCTACATTCTTATCACTTCCTTTATTTTTGCTTTTATGTTTGTGACAACTGTTGCTCA
CTTATTTTACTGGGATGCTTCATATGTCGTACACTTTATGAAAGCTAGGTTAAAGGGATACAGATCCTTG
AACTCACCAGAGAGTGTTTATGATGTCTTTGTGACTTATGACACCAGAGATCCACATGTCTCTGAGTGGG
TGATGAAAAATCTGCGGGTGAAACTGGAAGAGGAAGGAGAGACACATCTCCCTTTATGTCTGGAGGAGAG
GGATTGGCCCCTAGGAGTCCCCCTGGTGGACAACCTCACTCAGAGCATCCAATACAGTCGCAAGACCCTT
TTTGTCTTAACAGAGGGCTACGTTAAGACTGGGGTTTTCAAGCTAGCAATGTATCTGGCCCATCAAAGAC
TGCTGGATGAAAATGTGGATGTGATTGTGCTGCTGATGCTAGAGCCCGTCCTACAGCACTCTCACTTCCT
CCGTCTGAGGAGGAGGCTGTGTGAGAAAAGTGTTGTAGAGTGGCCGAGAACAGCAGCTGCAGAGGCTTGG
TTTTGGCAAAATCTGCGGAGTGTTGTCAGAGTAGACAATCAGATTATGTACAACAAGACTTATTCAAAGT
ACGGAGTCCAATAAGATCTTAATGGTGATGTGCTACATACCGTGCATTTGCTGGATAGTAGCTGATACCC
TGATGTACATCATGCAGAGCGGAACACAGGAAAGCCTTCCGAGGACTTGCACTGAGCTCTACGGCCACTT
CTGTTCCATGAAGGCAGAGGTAGGCGAACCAAGAGGCAGAGAGCCTGTAAAAATGGAGCAGCTTCATGGG
AGCAATCGTAAACTGCTGGGAAATCTTGGAAGACTGGCCTTTTATGGGCTCCTCAAACACAAATACACCT
TCAGTGAGCAGGACCTCAGGGCCTATGGGATCGACCTGTTGTTAACTCAGAGCAGTCTTGGTGCAGGGAT
TCTCATTCGGGAGGAGTCAGCCATATACACAACATACCGCTTCACCCATCTTACTCTGCAGGAGTTTCTT
GCCGCTACTTTCTACCATGTGTCCTCCAAGCGAGCCATCTTTGACTTGTTCTCAGAAAGCACCATGTCTT
GGCCCAAGATCGGCTTCCAGAACCACTTCAGGAGCGCCTTTCAACATGCACAGCAAGCTGAAGATGGCCA
TTTGGATGTGTTTGCACGCTTCCTGACAGGCCTGCTGTGCCCAGCAGCGCTGAAACCTCTTGCTGGACTT
TTGGCCCTTGGGAAAGATGATGGTAATCAGAAGGCATGGGCAGCAGGGTTTTTACAAGGCCTCTTGGTCA
GCGGGGGTGCTGTGGTGTCTCTGCGTGCAGTCAATGTGGCTTACTGTTTACAAGAGCTGCAACACACAGA
GCTGTTGCGTAGTGTAGAGGAGGATTTACGGCTTGGTAGCTTGGCGGGGAAGTTATCACGGGCTCACTGT
GTTGTGCTGGGCTACCTGCTGCATGTGTCTCCAGAATGCAGCGAACAGACAAACCTGACTGGCTCTTTGA
ATTACACCACAGTGAAATGTTTGCTCCCACAGTTGCTGTACTGCAGCCATCTCAGGCTAGAGAATAATCA
GTTCAAAGATGATGTCATGGAATTGCTGGGAAGTCTCCTGAGCGCCAAAGACTGCCATATTCAGAGGATT
AGTTTGGCAGACAACGCCGTCAGCAACAAAGGTGCCAAAGCGCTGAGTCGAGCCCTCTTAGTGAATCGGA
CACTAACGTCTCTCAATCTCCGGAACAACAACATTGGCTCTAAAGGTGCAAAGTTCCTGGCAGAAGCTCT
GAAAATGAACCAAGTCCTGGTATCAGTCAACTTTCAGAACAATGCCATCGAGGAGGAGGGTGCTCAGGCC
CTTGCTGAAGTACTGCAGTGCAACCGTAAATTGGTGTCTCTGAACATAAGGAAAAACACAATCGGAGCAG
GAGGAGCCAAGAGGATTGCAGATGCGCTGAAGACAAATCGGACTCTGACAAAGCTGATTCTTTGTAGTAA
CCAGCTCGGGGATAAAGGAACCATCGCTCTGGCAGAGGCTTTGGCGCTCAACCACACTCTGCTCTCACTT
CAACTTCAGAGTAACTCGATTAGCAACAGGGGGATGACCGCCTTAACCAAAGCACTCAGGCTGAACCGTG
GCCTTGTCTCCTTGAATTTAAGGGAGAACTCGATTGGAGTGGAGGGAGCGAAGAACATGGCCCATGCTCT
CTATGAAAACAGCTCTCTGCAGGACCTCGATCTCACAGCCAACCTGTTGCACGATGAAGGGGTTCAGGCT
ATTGCTGGAGTTTCTGGACGGCGATGAATGGAAAAGTCGCTGGGTGAACTCCAAACACAAGTCTGACTAT
GGAGAGTGGAAACTGACGGCTGGGAACTTCTATGGAGATGCTGAGAAAGACAAAGGTCTCCAGACAAGCC
AGGATGCTCGTTTCTATGCTACCTCTGCCCGCTTTGAGCCTTTCAGCAACGAGGGCAAGTCTTTGGTCAT
TCAGTTTACAGTCAAGCATGAGCAAAAGATCGACTGCGGTGGTGGCTATGTGAAGGTCTTCCCTGCAGAT

TTAGATCAGGCTGACATGCATGGAGACTCCTCATACTACATCATGTTTGGTCCTGATATCTGTGGCTACA
GCACAAAGAAAGTCCACGTTATCTTCAATTACAAGGGCAAGAATCATCTCATCAAGAAAGAGATTAAGTG
CAAGGATGATGAGCTGTCCCACCTGTACACACTGATCCTGAACCCAGATCAGACCTATGAGGTGAAGATT
GACAATGAGAAGGTAGAATCTGGCAGTCTGGAGGACGACTGGGACTTCCTGCCTCCCAAGAAAATTAAGG
ACCCCGAAGCTAAGAAGCCAGAGGACTGGGATGATCGTGCCAAGATTGACGACGCTGATGACACCAAGCC
TGAGGACTGGGACAAACCTGAAAATATTCCAGACCCTGATGCTAAAAAGCCTGAAGACTGGGATGAGGAT
ATGGACGGAGAGTGGGAACCACCCATGATCCCCAACCCAGAGTACAAGGGAGAATGGAAACCCAAACAGA
TTGACAACCCCAACTACAAAGGACCCTGGGTGCATCCTGAAATTGACAATCCTGAATACAGTCCTGATTC
AAACATCTACAAGTTTGACAACATTGGTATTTTGGGTCTTGACCTTTGGCAGGTGAAATCTGGAACCATC
TTTGACAACTTCTTGATCACAGACGATGTGAAGGAAGCAGAGGACATTGCAAAGGAGACATGGGGCGTGA
CAAAGGAACCAGAGAGGAAAATGAAGCAGGAGCAAGACGACCTGAAACGAAAAGAAGAGGAGGAGAAGAA
CAAAGAACAAGACACTGAAGCTGATGATGATGAAGATGAAGACGCAGATGAGGATGAAGAAGAAGAGGAT
GAAGAAACAAAGGAAGACATAGAGGAGGCACGTTCAGACATGGAAGAAGAGGAAGCAAAGCCTAAAGATG
AGCCCTCCAAACAGAAAGTGCTGGTGATGGAGTACTGTTCAGGAGGAAGTCTGCTCAGCCTGCTGGAGGA
GCCAGAAAACGCCTTTGGCCTGCCTGAAACAGAATTCCTCACCGTATTGCAGTGTGTAGTGCAGGGGATG
AACCACCTGCGTGAAAACGGAGTGGTACACCGGGACATTAAGCCAGGCAACATCATGCGACAGGTCGGGG
AGGACGGCAAGTCAGTTTATAAGCTGACAGACTTTGGAGCAGCAAGAGAGCTAGATGATGATGAGAAGTT
TGTGTCCATATATGGAACTGAAGAGTATCTGCATCCAGACATGTATGAACGTGCTGTGCTGCGTAAGGCT
CATCAGAAATCCTACGGGGTGAATGTTGACTTGTGGAGTATTGGTGTGACATTTTACCATGCTGCCACAG
GGAGTCTTCCCTTCACGCCATATGAAGGACCCCGCAGGAACAAACCCATCATGTTCAAAATAACCACAGA
GAAACCTATGGGTGCAATAGCTGGAATACAGAGGGTAGAGGGCGGACCCATAGAGTGGAGCTACCACCTA
CCTCACAGCTGCCAATTGTCACAGGGTCTGAGGGTGCAGCTAGTTCCAGTGTTAGCAGGCATACTG

>Seriola dumerili tlr3-tlr8-nlrc3-calr-ikbke
CCTTCTCTTCACTAAAGAACCTGCAATTTCTTGATGTTTCCACAAACAAACTGCCTTCAGCCAAGCTCAG
CTCTCAGCCTCAGCTGCCCAGCCTTGTGAACCTCAATCTGGCATTCAACGACTTCAGCACTCTGGAGAAA
GATGACTTTTCTTTCCTTAACCATTCACCCTTTCTACGAGTCCTCAATCTCTCGACTCTGCCTCTAAAAA
AATTGGAGCCCGGTTGCCTTGATCCCATTTCAGGCCTACATACGTTAATCATGGATGGGAGTAATATGGA
CAGTCAGGTCATTTCCAAACTCTGCTTAGAGTTGTCAGGGACATCCATTGAGACCCTGTCTCTTCAGAAG
GTAAAGCTGGTCACACTCTTGAACACAACCTTTACAGGGCTGCAGAAAGCAAACTTAACCTTTCTGGATC
TGTCCCATAATGGCATGGGTAAAATTGAGGAAGGCTCATTTCAGTGGCTGCCCAGACTTCAGATTTTAAT
TTTGGCCAACAACAACATCAAGCACCTGACCAAGGACACATTTCAGGGGCTCAAAAGTCTGAAAAAACTC
CAAATGACAAAAGCTCTGGTGAGAGGTCATACCTCCTCAACCCCGATTATTGATGATTTCTCCTTCCAAC
CATTAAGTGCCCTGGAGAGTTTAACATTACAGCAAACTGCAATTCGGGAAATCACAGCGAACACCTTTAC
AGGTTTGACAAGCCTTCAGGAACTTGATATGAGCTGGAGTACTTGTGCATCGCTCAGAAGCATCAGTAAC
AAAACCTTAGTCTCTCTCGCAGGATCGCCTCTCAGACAGCTAAATCTGAGAGGAACAGCTATAGGAGAGA
TTAATCCTGGAAGCTTCTCTGTTTTGGGAAACCTCACCATCCTCCTTCTAGATCATAATTTTATAAGTCA
AATTCTCACTGGCGAAGAGTTTAAAGGCCTTGGTCAGGTTCAAGAGTTACACATGAGCGATAACTACCAG
AAAGTCAAACTCAGCTCCACTTCATTTGTTAAGGTGCCCAATCTTAGGGTCCTGACTTTGGGAAAAAGCA
TTAATATGACAGCTTCCAAACTGGATCCCTCTCTATTCAAGCCCCTGTCCAACCTCACCGTCCTGGATCT
CAGTAACAACAACATCGCAAACATCATAGAGAATACGTTTGAGGGGCTTGTGAACCTGAAGGTGCTGAAG
CTCCAACACAATAACTTAGCCCGGTTGTGGAAGAGTGCCAACCTAGGTGGGCCAGTGTTGTTTCTCAAAG
GGGTACAGAACTTGATCACCTTACAGATGGATAGTAACGGGCTGGATGAGATCCCAGAGGAGGCTCTGAG
AGGTTTGAGCAACCTCAGTGAACTAAGTCTCTCAAGCAATCTCCTAAACAGTTTTAAGGACTCCGTTTTT
GATGATCTAACATCATTGCGTGTTTTACGTTTACAGAAGAACCTGATCACAACTGTGAGGCCCGAAGTGT
TCAAAACTCCGATGAGAAACCTCAGCCTACTTGTCATGGACAAAAATCCATTTGACTGCACATGTGAGAG
CATCCTGTGGTTTGTCAGATGGTTGAATAACACAAATGTTACCAGATTGCCAAACCTCAGGGACCAGTAT
ACATGCAACACTCCACGTGCTTACTTCAATCAATCAGTCATGGATTTTGACACCCTCTCTTGCAAAGATA
TGACCCCTTTTCAGGCTCTTTACGTACTGAGCAGCACAGTTGTCCTAATGCTGATGGTAACTGCACTTGT
GGTGCGGTTCCATGGCTGGAGGATTCAGTTTTATTGGAACATACTGATCAATCGCACATTAGGATTTAGT
GACGCCAGCGTTGAAGAGGGCAGGGAATTTGAATATGACGCTTACATCATACATGCAGAAGAAGACAGCC
GCTGGGTGGAAAGGATGATGGTCCCCTTAGAGAATGAAAGGTGCAGGTTTTGTTTGCAGGACCGAGATGG
AGTCCCTGGAATGCCACTGCTTGAATCCATTGTGGATAATATGAAAATGTCCAGGAAAATCTTGTTCGTC
GTCACTGAAAGTCTTCTCAAAGATCCCTGGTGTAGACGATTTAAAGCCCACCACGCACTTAACGAGGTCA
TTGAAGCCAGCAGGGACTCTGTGGTTCTGGTCTTCCTGCAGGATGTGCACGACTACAAGTTGTCTCGCTC
ACTCTTCCTCCGCAGGGGCATGTTGCGCTCGAGATGCATCCTGGACTGGCATTGTCATAAGGAGAGGGTG
CCGGCCTTTCACCAGAAGCTCCTCATTGCACTTGGCATGACAAATCGATTGAAGGAGTGACTTATCAAGG
AAGAGTGCTTTGAATTGGGTCGAGTGCTAATCCTCAGCTCAAATAATCTGTTCTTCATTTCCCCAGAGCA
ATTTAACGGCTATGGAGATATTGCATGCCTCAACCTCTCAAGAAATGGCTTTTCAGCAGCACTTAATGGC
ACAGAGTTCTCTTTGCTCCCTAATCTGACATACCTGGACCTGTCATTCAATAGGATTGATCTGGCCTATG
ACAACGCCTTCAAAGAACTAAAGAAACTACAAGTACTAGACCTCAGTTACAACGAGCACTACTTTAAAGC

ATATGGTATAACGCATAATTTAAATTTTACAAAAAATCTGCCTGTTCTGAGGGTGCTGAATATGAGTCAT
AATGCCATTTCCACACTGACAACAAAACAGATGTACAGCGAATCATTATCAGAACTGCAGTTTACAGATA
ATAATCTTGGGACTCTTTGGAAAGAAAGGGATGGCTCATACAAAATGCTTTTCACTAATCTCATTAATTT
GACCATTTTAGATATATCCCAAAACCACATTGCAAAGATTCCTGTTGATGTTTATAAATATTTGCCACGT
AACCTCACCAAACTACGTATAAGCCATAACGGGCTTGCTGACTTTAGATGGGACTCGCTGAGGTATTTCC
ATCAACTTCAAATTTTAGACCTGAGCTTCAATTCTTTATCTGACGTGACAGGTATAAACTCAAACATCAC
TCAGACTTTGACTTTTCTTGACCTGAATCACAACCGTATTTTCCAGTTGGACAATGGATTTATAAAGGGT
CCCAAAAGCCTTAAGACTCTTAGCCTTAGCAACAACAAACTGACCACCATCAATCAATCCACCTTCCAGT
CCAGACCTGATAATCAGATTCAGACTTTGTTCTTGCAGGGAAACCCATTCCAGTGTACCTGTGATTTATT
AGATTTCATTCTATGGATTGAAAAAAGTGAAGTAAATATCCCGAGACTGACCACTATGGTGACATGTCAC
ACACCAGCAAACCAGAAGGGTCAAGCACTGATATACTTTGACATTAACCAGTGTGTAAATGACAGTCAAG
CATTCCTGGTCTACATTGTCACAACTTCCTTCATTATTGCTTTTATGATTGTGGCAACTGTTGCTCACTT
ATTTTACTGGGACGCTTCCTATGTAATTCACTATATGAAAGCTAAGTTGAAGGGATACAGATCCTTGAGC
TCACCAGACAGCGTTTATGATGTCTTTGTGACTTATGACACCAGAGATCCACATGTCTCTGAGTGGGTGA
TGAGAAATCTGCGGTTGAAACTGGAGGATGAAGGGGAGAAGCATCTTCCTTTGTGTCTGGAGGAGAGGGA
TTGGCCCCCAGGAGTCCCACTGGTGGAGAACCTCACTCAGAGCATCCGATACAGTCGCAAGACCCTTTTT
GTCTTAACTGAGGACTATGTTAAGACTGGGGTTTTCAAGCTGGCAATGTATCTAGCTCACCAAAGACTTC
TGGATGAAAATTTGGATGTGATTGTGCTGCTGATGCTGGAGCCTGTTCTGCAGCACTCTCACTTCCTGCG
CCTGAGGAGGAGGCTGTGTGGGAAAAGTGTTGTAGAGTGGCCGAGAACAGCAGCGGCAGAGCCCTGGTTT
TGGCAAAACCTTAGAAATGTTGTCAGAGTAGACAATCAGGTGATATACAACAAGACTTATTCAAAGTACG
TGATGTGCTACATACCATGCATTTGCTGGATAGTAGCTGATACTCTCATGTACATCATGCAGAGTGAAAC
ACAGGAGAGCCTTCCAAGGACTTGCACTGAGCTTTACGCCCACTTCTGTTCCATGAAGGCAGAAGTAGGC
GAACCAAGAGGCAGGGAGCCTGTGAAAACGGAGCAGCTTCATGGAAGCAATCGTAAACTGCTGGGGAATC
TTGGACGACTGGCATTTTATGGGCTCCTCAAACACAAGTACACCTTCAGTGAGCAGGACCTCAGGGCCTA
TGGGATAGATCTACTGTTAACTCAAGGCAGTCTTGGTGCAGGAATTCTTGTTCGGGAGGAGTCAGCCATA
TACACAACCTATCGGTTCACACATCTGACTCTGCAGGAGTTTCTTGCAGCTACTTTCTACCATATCTCCT
CCAAGCGAGCCATCTTTGACTTGTTCTCAGAGAGCACCATGTCCTGGCCTAAGATCGGTTTCCAGAATCA
CTTCAGAAGTGCCTTTCAGCATGCACAACAGGCTGAAGAAGGTCATTTGGATGTGTTTGTGCGCTTCCTG
ACAGGCCTGCTGTGCCCAGTGGCACTGAAACCTCTCGCTGGGCTTTTGGCCCTTGGGAAAGATGATGGAA
ATCAGAAGGGCTGGGCAGCAGGATTTTTACAAGGCCTCTTGGACAGCGGGGGTGCTGTGGTGTCCCTGCG
TGCAGTCAACCTGGCTTATTGTTTACAAGAACTGCAACACACAGAGCTGTTGAGGAATGTAGAGGATGAT
TTACGGCTTGGTAGTCTGGCAGGGAAGTTAAGTCGGGCTCATTGTGTTGTGCTGGGCTACCTGCTGCATG
TGTCTCCAGAGTGCAGCGAACAGACCAACCTTACAGGCTCTCTGAACTACGCCACAGTGAAATGTTTGCT
CCCACAGTTGCTGTACTGCAGCCATCTCAGGTTGGAGAATAATCACTTCAAAGATGATGTCATGGAATTG
CTGGGAAGCCTCCTGAGCGCCAAAGACTGCCATATTCAGAAGATAAGTTTGGCAGAAAACGCCATCAGCA
ACAAAGGAGCCAAAGCACTGAGCCGAGCCCTGTTGGTGAACCGGACACTAATTTCTCTCAATCTCCGGAA
CAACAATATCGGCTCCAAAGGTGCAAAGTTCCTGGCAGAAGCTCTGAAAATGAACCAAGTCCTGGTATCG
ATCAACTTCCAGAACAATGCCATTGAGGAGGAAGGAGCTCAGGCCCTTGCAGAAGTATTGCAGTGCAACC
GCAAATTGGTGTCGCTGAATGTACGGAAGAATACAATTGGAGCAGGTGGAGCCAAGAGGATTGCGAATGC
ACTGAAGACAAACCGGACTCTCACAAAGCTGATCCTTTGTAGCAACCAGCTTGGGGACAAAGGAACAGTG
GCCCTGGCAGAGGCTTTGACACTCAACCACACTCTGCTCTCGCTTCAACTTCAGAGTAACTCGATCAGCA
ACAGGGGGATGACTGCCTTAACCAAAGCACTCAGGCTGAACCGTGGCCTTGTCTCCTTAAATCTCACAGC
CAACCTCTTGCATGATGATGGTGTTCAGGCTATAGCTGGAGTTTCTGGACGGTGATGACTGGAGAAGTCG
CTGGGTGAACTCCAAACACAAGTCTGACTATGGGGAATGGAAACTGACGGCTGGCAGCTTTTACGGAGAT
GCTGAGAAAGACAAAGGTCTCCAGACAAGCCAGGATGCTCGTTTCTATGCTGCCTCTGCCCGTTTTGAGC
CTTTCAGCAACGAGGGCAAGTCTGTGGTCTTTCAGTTTACGGTCAAGCATGAGCAAAAGATTGACTGTGG
TGGTGGTTATGTGAAGATCTTCCCTGCTGATTTGGATCAGGCTGAAATGCATGGAGAATCCTCATACTAC
ATTATGTTTGGTCCTGATATCTGTGGCTACAGCACCAAGAAAGTCCATGTCATCTTCAATTACAAGGGAA
AGAATCACCTCATTAAGAAAGAGATTAAGTGCAAGGATGATGAGCTGAGCCACCTGTACACGCTGATCCT
GAATCCAGATCAGACCTATGAGGTGAAGATTGACAACGAGAAGGTAGAATCTGGCAGCCTGGAGGAGGAC
TGGGACTTCCTGCCTCCTAAGAAAATTAAGGACCCCGAAGCCAAGAAGCCAGAGGACTGGGATGATCGTG
CCAAGATTGATGATGCTGATGATACCAAACCTGAGGACTGGGACAAACCTGAAAACATTCCAGACCCTGA
TGCTAAAAAGCCTGAAGACTGGGATGAGGATATGGACGGCGAGTGGGAGCCACCCATGATCCCCAACCCA
GAATACAAGGGAGAATGGAAACCCAAACAGATCGACAACCCCAACTACAAAGGAGCATGGGTGCATCCTG
AGATTGACAATCCTGAATACAGTGCTGATTCAAACATCTACAAGTTTGACAGCATTGGTATTTTAGGTCT
CGATCTTTGGCAGGTGAAATCTGGTACCATCTTTGACAACTTCCTGATCACAGATGACGTGAAGGAAGCA
GAAGACATTGCAAAGGAGACATGGGGTGTGACAAAGGAGCCAGAGAGGAAAATGAAGCAGGAGCAAGACG
ACCTGAAACGAAAAGAAGAGGAGGAGAAGAACAAAGAACAAGACACTGAGGGTGGTGATGAAGAGGAAGA
GGAAGAAGAAGAGGAAGAAGAGGAGGAGGAGGAAGAAACAAAGGACGACCTAGAAGAAGCTCTTTCAGAA
ATGGATGATGAGGAAGTAAAGCATAAAGATGAGCCCTCTAAGCAGAAGGTGCTGGTGATGGAGTATTGTT
CAGGAGGAAGTCTGCTCAGCCTGCTGGAGGAGCCAGAAAACGCCTTTGGCCTGCCTGAAACAGAATTCCT

CACTGTGTTACAGTGTGTAGTATTCCTCTCTCTGCTCTCTTTAGTGCAGGGGATGAACCACCTGCGTGAA
AACGGAGTGGTACACCGGGACATTAAGCCAGGCAACATCATGCGGCAAGTCGGGGAGGACGGCAAGTCTG
TTTATAAGCTGACGGACTTTGGAGCAGCAAGAGAGCTGGAGGATGATGAGAAGTTTGTTTCTATCTATGG
AACAGAAGAGTATCTGCATCCAGACATGTATGAACGTGCTGTGCTGCGCAAGGCTCATCAGAAGTCCTAT
GGAGTGAGTGTTGACTTGTGGAGTATTGGTGTGACATTTTACCATGCTGCCACTGGGAGTCTTCCCTTCA
CACCGTATGAAGGACCCCGTAGGAACAAGCCCATAATGTTCAAAATAACTACAGAGAAACCTGTGGGTGC
AATAGCTGGAGTACAGCGAGTGGAGGGTGGACCTATAGAGTGGAGCTACCACCTACCTCACAGCTGCCAA
CTGTCACAGGGCCTGAGGGTGCAGCTAGTCCCAGTGTTAGCAGGTATACTG

>Scophthalmus maximus tlr3-tlr8-nlrc3-calr-ikbke
CCTTCTCCGCCCTAAAGAATCTGAAATCCCTTGACGTCTCCATGAACAAACTGGCTTCGGCCCAGCTCTC
CTCGCAGCCTCAGCTGCCCAGCCTGGTGAACCTCAATCTGGCATTCAATGACTTCACCGCTCTGAAGAAA
GATGACTTTTCTTTCCTCGACAACTCATCCTTCCTACAACTCCTCAATCTCTCGGCCGTGTCTCTGAAAA
CATTGGAGCCTGGTTGCCTTCGGCCCATTTCCGGCCTACGCACTTTAATCATGGATGGAAGTAATATGGG
CATTCTGGTTCTTTTCAAACTCTGCACAGAGCTGTCGAGGACAGCCATTGATGCCCTGTCTCTCCGGAAG
ATGAAGCTAGTCACCCTCACAAACACAACCTTTACCGGGCTGCAGAGAGCAAATCTAACCTTTTTGGATC
TGTCCCACAATGGCATGGGTAAAATCGAAGAGGGGTCATTCCAGTGGCTCTCCAGACTTCAGACTCTGAT
TTTGGCCGACAACAACCTCAAGCATCTGACCAAGGGCACATTTCAGGGGCTCAAGGCTTTGAAAGAACTC
CAACTGACAAAAGCCCTGGTGAAAAGTCATACATCCTCCACCCCGATAATCGACGATTTCTCTTTCGAGC
CATTGAGCGCACTGGAGAGCTTATCGTTACGGCAAACCTCTGTTCGGGAAATCACAGGGCACACATTTAC
AGGCATGACAAGTCTCAAAGAACTGGATATGAGCTGGAGTAGCTACACGTCGCTCAGAATCATCACCAAA
CAGACCTTAGTCTCACTTGCAGGATCACCTCTCGTGAAGCTAAATCTGACAGGAACAGCGATTACACAGA
TTAATCCTGGAAGCTTTGCGTGCATGAGAAACCTCACCACTCTTCTACTAGATTTCAATTTCATACAGCA
GACTCTCACTGGCAAAGAGTTCGAAGGCCTGGGTGAGGTTCAGGAGCTGCACATGACCAATAACCACTGG
AAAGTCAGACTAAGCTCCACTTCGTTCGTCAATGTGCCCAATCTTAGGGTCCTGACTTTGGGCAAAAGTC
TCAACAACACAGCTTTGAACGTGGATGTGGATCCCTCGCCATTCAAGCCCCTGTGCAACCTCACCTTCTT
GGATCTCAGCAATAACAACATAGCGAACATCAGAGGGAGTGTTTTGGAGGGCCTTGTGAACCTGAAGGTG
CTGAAGCTCCAACACAATAACTTAGCCCGGCTGTGGAAGAGTGCCAACCCAGGTGGGCCGGTGCTGTTTC
TCAAAGGGGCACCGAACCTGGTGACCTTACAGATGGACAGTAACGGGCTGGATGAGATCCCGGAGGGTGC
TTTGAGAGGTCTGAGTAGCCTCTGGGAGCTAAGCCTCGCGACCAATCTCCTGAACAATCTTAGGTACTCG
GTTTTTGACGATCTGAAATCATTGCGGGTTCTACGTTTACAGAAGAACCTGATCACATCCGTCAACCCCG
AAGTGTTCAAAACTCCCATGAGCAACCTCAGTCTACTTGTCATGGACAAAAATCCTTTTGACTGCACATG
TGAGAGCATCCTGTGGTTTGTGACGTGGTTGAATAACACAAACACCACCTTTGTGCCAGATCTCGGGGAC
CAGTATATTTGCAACACGCCACTGGCTTACTATAACCACTCAATCATGGATTTTGAAACCCTCTCTTGCA
AAGACATGACCCCATTTCAGGCTCTTTACATACTGACCAGCACAACGGTCATGGCGGTGATTGTAACGGC
ACTTTTGGTGCGGTTCCAAGGCTGGAGGATTCAGTTTTATTGGAACATACTGATCAGTCGGACGCTAGGA
TTTAGCGATGCCAATATTGACGAGGGCAGGCACTTTGAGTACGACGCTTACATCATACATGCTGAGGAAG
ACAGCAGATGGGTGGAGAGGATGATGGTCCCCTTGGAAAACGAGAACTGCAGGTTTTACCTGGAGGATCG
AGACGCTGTCCCTGGGGTGGCGCTGCTCGAATCTATCATCGATAATATGAGAAGGTCCAGGAAGATCTTG
TTTGTGGTCACCGAACGTCTCCTCAAGGATCCCTGGTGTCGTCGATTCAAAGCCCATCAGGCACTTCACC
AGGTCATCGAGGCCAGCAGGGACTCTGTGGTTCTGGTCTTCCTGCAGGACGTGCATGACTACAAGTTATC
TCGCTCACTCTACCTCCGCAGGGGCATGTTGCGCCGCTGTTGCATCGTGGAATGGCACGGCGGCCATAAG
GAGAGGGTGGCGGCCTTCCACCAGAAGCTCCTCATAGCACTCGGCATGACCAATCGATTGATGGAGTGAC
TTGTCAAGCAAGAGTGCTTTGACTCTGGCCAAATGCTAATCCTCAGCTCAAATAATCTGTTCTTCATTTC
TCCAAAGCAGTTTGACGGCTATGAAAATATCGCATGTCTCAACCTCTCTGGAAACGGATTTTCAGCAGCA
CTCAATGGCACAGAGTTCTCCCTGCTGCCCAACCTGACATACCTGGACCTGTCATTCAACAAGATTGATC
TGGCCTATGACCACGCCTTCAAAGAGCTGGAGAAACTACAAGTACTAGACCTCAGTTACAATTCACACTA
CTTTCAAGCATACGGGGTAACGCACAATTTAAATTTCCTAAAAAATCTGCCCGCTCTGAGAGTGCTGAAT
ATGAGTCACAATGCCATTTCCACTTTGACAACAAAACAGTTGTACAGCACATCTTTGACAGAACTTCAGT
TTACAAATAATTATCTTGGAACCCTTTGGAAAGTAAGAGATGCCACATACACAATGCTTTTTACTAATCT
TACTAATTTGACATTTTTAGATATATCCCAAAACAAACTTCAAAATATTCCCGACGAAGTTTATGAATAT
TTTCCACGTAACCTCACCAAACTGTGCATAAGCCGCAACTTGCTCACAGATTTTAAATGGGAGGCGCTGA
AGTTTTTCCATCAACTTCAAATTTTAGATCTGAGCTTTAACTCTATATCTGCCGTGGCTGCTATAAACTC
GAGCATCACGAAAACTTTGACTTTGCTTGACTTGAGTCACAACCAGATTTTCCACTTGGACGATGGATTT
ATTAAGGGTCCGAAAAGCTTCAAGACTCTCATCCTTAGCTTCAACCTACTGAACACCATCAATCAGACTA
CCTTCCAGACGAGATCCGAGGATTCGATTCAGACCTTGTTCTTGCAGGGAAATCCGTTCCAGTGTATCTG
TGATTCATTCGATTTCATTCTGTGGATTGAAAACAGTCAAATAAAAATCCCCAGACTGACCGCTGAGGTG
ACATGTGACACACCGGCAAACCAGAAGGATCAACCGCTGATATACTTTGACATTACCCAGTGTGTAAATG
ACAGTCAGGCATTCCATGTATACATTCTCACAACTTCCTTCATTATTGCTTTTATGTTTGTGGCAACTGT
TGCTCACTTATTTTACTGGGATGCCTCCTATGTATTACACTATATGAGAGCAAAGTTGAAAGGATACAGA
TCCTTGAACTCCCCGGACAGCTTTTATAATTTGTTCGTGACTTACGACACCAGAGATCCACACGTCTCTG

AGTGGGTGATGAGAAATCTCCTGGTGAAACTGGAGGAGGAGGGAGAGAAGCATATTCCTCTGTGTCTGGA
GGAGAGGGATTGGACGCCGGGAGTCCCGGTCATGGACAACCTCACTCACAGCATCCAATACAGTAGCAAG
ACCCTGTTTGTCCTCACGGAGGGCTACGTTAAGACTGGGGTTTTCAAGCTGGCAATGTATCTAGCCCACC
AGAGACTACTAGACGAAAATGTGGATGTGATCGTTCTGCTGATGCTGGAGCCCGTCCTGCAGCACTCGCA
CTTCCTGCGCCTGAGGAGGAGGCTGTGCGGGAGGAGCGTGGTGGAGTGGCCGAGAACAGCGGCCGCAGAG
CCCTGGTTTTGGCAAAACCTGAGGAACGTCGTCAGGGTGGACAACCAGGTCATGTACAACAAGACTTATT
CAAAGTACGGAGTCCTACAAGATCATAATGGTGATGTGCTACATACCAAGCATTTGCTGGATAGTAGCTA
ATACTCTGATGTACATCATGCAAAGTGGAACACAGGAAAGTCTTCCAAGGACTTGCACTGAGCTCTATGC
CCACTTCTGTTCCATGAAGGCAGAAGTAGGCGAACCAAGAGGCAGGGAGCATGTAAAAATGGAGCAGCTT
CATGGGAGCAATCGAAAACTGCTGGGGAATCTTGGGCGATTGGCCTTTTATGGGCTCCTCAAACACAAGT
ACAGCTTCAGTGAGCAGGACCTCAGGGCCTATGGGATAGATCTACAGTTAACTCAAAGCAGTTTTGGTGC
CGGAGTTCTAGTTCGGGAAGAGTCGACCATATACATAACTTATCGGTTCACTCATCTGACTCTGCAGGAG
TTTCTTGCAGCTACATTTTACCATGTATCCTCCAAACGGGCTATCTTTGACTTGTTCTCAGAAAGCACCA
TGTCCTGGCCCAAGATTGGTTTCCAGAACCACTTCAGAAGTGCCTTTCAACGTGCGCAGCAAGCTGAAGA
TGGACATTTGGATGTGTTTGTGCGTTTCCTGACAGGCCTGTTGTGCCCAGTCACACTGAAACCTCTCGCT
GGGCTTTTGTCCCTTGGGAGAGATGATGGTAGTCAGAAGGCCTGGGCAGCAGGGTTTTTACAAGGCCTCT
TGGTCAGCGGGGGTGTTGTTGTGTCCCTGCGCACAGTCAATCTGGCTTATTGTTTACAGGAGTTGCAACA
CACAGAGCTGTTGCGGAGTGTGGAGGAAGATTTACGGCTTGGTAGCCTTGCAGGGAAGTTAACATTGGCT
CACTGTGTTGTGCTTGGATATCTGCTGCATGTGTCTCCAGAGTGCAGTGAACAGACCAACCTAACAGGCT
CTCTGAACTACACCACAGTGAAATGTTTGCTCCCACAGTTGCTGTACTGCAGTCATCTCAGGTTAGAGAA
TAATCACTTTAAAGACGACATCATGGAATTGCTGGGAAGCCTGCTCAGTGCCAAAGACTGCCACATTCAG
AAGATTAGTTTGGCAGAGAACTCCATTAGCAACAAAGGTGCCAAAGCACTGAGTCGAGCACTGTTGGTGA
ACCGGACACTAACTTCTCTCAATCTCCGGAACAACAACATCGGCTCCAAAGGCGCAAGGTTCCTGGCAGA
AGCACTGAAAATGAACCAAGTCCTGGTATCGATCAACTTCCAGAACAATGCCATCGAGGAGGAAGGTGCT
CGAGCCCTCGCAGAAGTGCTACAGTGCAATCGCAAATTGGTGTCTCTGAACATACGGAAGAATGCGGTCG
GAGCAGGAGGAGCCAAAAGGATTGCTGACGCGCTGAAGACCAACCGTACCCTCACAAATCTGATTCTTTG
CAGCAACCAGCTTGGGGATAAAGGGACAATCGCTCTGGCAGAGGCTTTGACGCTCAACCACACTCTACTC
TCACTTCAACTTCAGAGTAACTCAATCAGTAACAGGGGGATGGCGGCCTTGACCAAAGCTCTTAGGCTGA
ACCGTGGTCTTGTCTCTTTGAATTTAAGGGAGAACTCGATTGGGGTGGATGGAGCCAAGAACATGGCTCA
TGCACTCCATGAAAACAACTCTCTACAGGACCTTGATCTCACAGCCAACCTGTTGCATGATGAAGGGGTT
CAAGCTTTAGCTGGAGTTCCTGGACGGTGACGAGTGGACAAGTCGCTGGGTCACCTCCAAACACAAGTCT
GACTATGGAGAGTGGAAACTGACGGCTGGTAACTTCTACGGAGATGCTGAGAAAGACAAAGGTCTTCAGA
CGAGTCAGGATGCTCGTTTCTACGCTACCTCGGCCCGTTTTGAGCCTTTCAGCAACGAGGGCAAGTCTGT
GGTCATTCAGTTTTCAGTCAAGCACGAGCAGAAGATTGACTGTGGTGGGGGCTACGTGAAGGTCTTCCCC
GCAGATCTGGATCAGGCCAACATGCACGGAGAATCCTCGTACTACGTCATGTTTGGTCCTGATATCTGTG
GCTATAGCACCAAGAAAGTCCACGTGATCTTCAATTACAAGGGCAAGAATCACCTCATCAAGAAAGAGAT
CAAGTGCAAGGACGATGAGCTGACCCACATGTACACACTGATCCTGAATCCCGATCAGACCTATGAAGTG
AAAATTGACAACGAGAAGGTAGAATCTGGCAGTCTGGAGGAAGACTGGGACTTCCTGCCTCCCAAGAAAA
TTAAGGACCCTGAAGCCAAGAAGCCAGAGGACTGGGATGATCGTGCCAAGATCGACGATTCTGATGACAC
TAAGCCTGAGGAGTGGGACAAAGCTGAGAACATTCCAGACCCTGATGCTAAAAAGCCTGAAGACTGGGAC
GAGGATATGGACGGAGAGTGGGAGCCACCCATGATCCCCAACCCAGAGTACAAGGGAGAATGGAAACCCA
AACAGATCGACAACCCCGACTACAAAGGAGCATGGGTGCATCCTGAGATTGACAACCCTGAATACAGTGC
CGATTCAAACATCTACAAGTTTGACAACTTTGGTGTTTTAGGTCTTGATCTTTGGCAGGTGAAATCTGGC
ACCATCTTCGACAACTTCCTCATCACAGACGATGTGAAGGAAGCAGAAGACATTGCAAATGAGACATGGG
GTGTGACAAAGGAACCAGAGAGGAAAATGAAGCAGGAGCAAGACGACCTGAAACGAAAAGAAGAGGAGGA
GAAGACCAAAGAACAAGATACTGAGGCCGAGGAGGAAGAAGATGAAGACGTAGACGAGGAGGAAGAAGAG
GAGGAGGAAGAAACAAAAGATGAATTAGAGGAGGCACTTTCAGAAATGGAGGAGGAGGAAGCAAAAATTA
AAGATGAGCCCTCTAAGCAGAAGGTGCTGGTGATGGAGTATTGTTCAGGAGGGAGTCTGCTAAGCCTGCT
GGAGGAGCCAGAAAATGCCTTCGGCCTGCCAGAAAAAGAGTTCCTTACCGTACTGCAGTGTGTAGTGCAG
GGGATGAACCACCTGCGTGAAAATGGCGTGGTACACCGGGACATCAAGCCAGGCAACATCATGCGGCAGG
TCGGCGACGATGGCAAGTCTGTTTATAAGCTGACTGACTTTGGAGCAGCGAGAGAGCTGGAGGATGATGA
GAAGTTTGTGTCTATCTATGGAACTGAAGAGTATCTGCATCCAGACATGTATGAACGTGCAGTGCTGCGT
AAGGCTCATCAGAAATCTTACGGAGTGAGTGTTGACTTGTGGAGTATTGGTGTGACGTTTTACCATGCTG
CCACCGGGAGTCTTCCCTTCACACCGTATGGAGGACCTCGCAGGAACAAGCTCACCATGTTCAAAATAAC
TACAGAGAAACCTGTGGGTGCAATAGCTGGAATCCAGCGTGCAGAGAGCGGACCTATAGAGTGGATCTAC
CACCTACCTCACAACTGCCAACTGTCCCGCGGTCTGAGGGTGCAGCTGGTCCCAGTGTTAGCAGGTATTT
TG

>Paralichthys olivaceus tlr3-tlr8-nlrc3-calr-ikbke
CCTTCTCTGCACTAAAGAATCTGAAATCTCTCGATGTTTCCATGAACAAAATGGTGTCAGCCAAGCTCAG
CTCTCGGCCTCAGCTGCCCAAACTGGTGTCCCTCAATCTGGCATTGAACGATTTCACCACACTGAAGAAA

GAAGACTTCTATTTCCTTGAAAATTCAGCACTCCAAGTCCTTGATCTCTCATCCGTGCCTCTAAAAACAT
TGGAGCCTGGTTGCCTTAAGCCCATTTCAGGCCTGCGTACTTTAATCATGGATGGGAGCAATATGGGTGC
TCAGGTTCTTTCCAAACTCTGCTCTGAGCTGTCGGAGACATCTATCGATGCCTTGTCTCTACGAAAAAAT
AACCTGGTCACACTCACAAACTTGACCTTTGCAGGGCTGCAGAAAGGAAATCTAACCTTTCTGGATCTGT
CCCATAACAGCATGGGGAAAATACTGGAAGGCTCATTTCAGTGGCTCCCCAGACTTCAGACTCTAATTTT
GGCTGACAACAATATCAAGCACCTGACCAAGGCCACGTTTCAGGGGCTCAAAAGTTTGACAAAACTCACA
CTGACAAAAGCCCTGGTGAAAGGTCGAACCTCCGCCACCCCAATAATAGATGATTTCTCTTTTCAACCAT
TAAGCACCCTGGAGAGTTTAATGTTACAGCAAACTGCAATTCGGGAAATCACAGCACAAACATTTACAGG
CTTGACAAGTCTTAAAGAGCTTGATATGAGCTGGTGTAGTTGCATATCACTCCGAGATATCACCAACAAA
ACTTTATTGTCACTTGCAGGATCGCCTCTCAGAAAACTAAATCTGACAGCAACAGCTATGACAAACATAA
ATCCTGGAAGCTTCTCCTTTTTGACAAACCTCACCAATCTTCTTCTAGATTTAAATTACTTACAGCAAAC
TCTCACTGGAAAAGAGTTTGAAGGCCTGGGTCAGATTCAGGAGATTCACATGACCTACAACCACTGGAAA
ATCCAGCTGAGCTCCACCTCATTCATCAATGTGCCCAACCTTAGGGTCCTGACATTGGGAAAAAGTCTGA
ACAGCAATGCTTTGAATCTGGATCCATCACCATTCCAGCCCCTGTCCAACCTCACCTACCTGGATCTCAG
TAACAACAACATCGCAAACATCAGACAGGATATGTTGGAAGGGCTTGAGAACCTGAAGGTGCTGAAGCTC
CAACACAACAATTTGGCGCGGTTGTGGAAGCACGCCAACCCAGGTGGGCCGGTGATGTTTCTGAAAGGGG
CAGCAAAGTTGATGACCTTATGGTTGGATAACAACGGGCTGGATGAGATCCCAGAGGAGGCTCTGAAAGG
TTTGAGCAACCTCAGGGAGCTAAGCCTCTCAAACAATCTCCTCAACAGTCTGAAGGACTCTGTGTTTGAT
GATCTGAAGTCCTTGCGGGTTTTGCGTTTACAGAAGAACCTGATCACAACTGTGAAGCCCGAAGTGTTCA
GCACTCCCATGAGCAACCTCAGCCTACTGGTCATGGACAAAAATCCATTTGACTGCACATGTGAGAGCAT
CCTGTGGTTTGTGACGTGGTTGAACAACACAAACATGACCAGTGTGCCAGACCTCAGCGACCAGTATATG
TGCAACACTCCCCTTACCTACTTTAATCACTCAATCCTGATGTTTGACACCCTCTCCTGCAAAGATATGA
CCCCATTTCAGGCTCTCTACATACTGAGCAGCACCGCAGTCTTGATGCTGATGGTATCGGCGCTGTTTGT
GAGGTTCCACGGCTGGAGGATTCAGTTCTACTGGAACATACTGGTCAGTCGCACGTTAGGATTTAGTGAT
GCCAGTGTTGAAGAGGGCAGGGAATTTCAGTATGACGCTTACATCATACATGCAGAGAGGGACAGCAGAT
GGGTGGAGAGAGTGATGGTCCCCTTAGAGAATGAAAAGTGCAGGTTCTATTTGGAGGATCGAGATGCAGT
CCCTGGGGTGTCGATGATCGAATCCATCATGGAGAACATGAGAAGGTCCAGGAAAATCTTGTTTGTCGTC
ACTGAAAGTCTTCTCAGGGATCCCTGGTGTAGACGATTCAAAGCCCACCATGCACTGCACCAGGTCATTG
AGGCCAGCAGGGACTCTGTGGTTCTGGTCTTCCTGCAGAATGTGCACGACTACAAGTTATCTCGCACGCT
CTTCCTCCGCAGGGGCATGTTGCGGCCATGTTGTATCTTGGAATGGCCCGAAGATAAGGATAGGGTGCTG
GCCTTTCACCAGAAACTCCTCATTGCACTTGGTATGACCAATAGATTCAAGGAGTTTGTCAAGAAAGAGT
GCTATGAATCAGGCCGAATTTTAATCCTCAGCTCAAATAATATCTTTTTCATTTCTCCAAAGCAATTTGA
GGGCTATGAAAATATTGCATGTCTTAACCTCTCAGGAAATGGATTTTCTGCAGCACTTAATGGCACAGAG
TTCTCCTCGCTACCTAATCTGACATACCTGGACCTATCATTTAACAAGATTGACCTGGCCTATGACAATG
CCTTCAAAGAACTGAAGAAACTTCAAGTACTCGACCTCAGTTACAACCCACATTACTTTCAGTCATATGG
GATAACGCACAATTTAAATTTCATAAAAAACCTGCCTGTTCTGAGAGTGCTGAATATGAGTCACAATGCC
ATTTCCACATCATCAACAAAACAACTGTACAGCCAATCTTTGTCAGAACTTCAGTTTACAAATAACTTTC
TTGGAACTCTTTGGAAAGAAAAGGATGTCACATACAAGCAGCTTTTTACTCAACTCACTAATTTGACATA
TTTAGATATATCCGAAAACCAAATTAAAAAAATTCCAGATGATGTATATCAATATTTTCCCCGTAACCTC
ACCAAACTATGCATAAGCCATAACTTACTCACTGGTTTTAAATGGGACGCAATGTCATATTTCCATCAAC
TTCAAGTCTTAGACCTGAGCTTCAATTCTTTATCTGACGTGACGGGTATAAACTTGAGCTTCACTCAGAC
TTTGACTTTCCTTGACTTGAGCCATAACAAAATTTTCCACCTGGACGATGGATTCATAAAGGGTCCGAAG
AGCCTTAGCATTCTCAGTCTTAGCTACAACAAACTCACCACCATCAATCAATCTACCTTCCAGTCCAGAC
CTGAAAATCAGATTAAGATTTTGCACTTGCAGAATAATCCGTTCCAATGTACCTGTGACTCTATCGATTT
CATTCTGTGGATTGAAGCCAGTAAAGTAAAAATCCCAAGACTGACCACTGAAGTGAAATGTGACACGCCA
GCAAACCAGAAGGATCACATACTGATATACTTTGATATAAAACCGTGTGTAGATGACAGTAAGGCATTAC
GGATGTACATCCTCACTACCTCCTTCATTATTGCTTTTATGTTTGTGGCAACTACTGCTCACTTATTTTA
CTGGGATGCCTCCTATGTGATACACTATATGAAAGCTAAGCTGAAGGGGTACAGTTCCTTGAATTCATCA
GACAGCTTTTATGATGTCTTTGTGACTTATGACAACAGAGATCCACATGTCTCTGAGTGGGTAATGAGCA
ATCTGCGGGTGAAACTGGAAGAGGAAGGAGAGAAGCATCTCCCTCTGTGTCTGGAGGAGAGGGACTGGGT
CCCAGGAGTCCCGGTGCTGGACAACCTCACCCACAGCATCCGATACAGTCGCAAGACCCTGTTTGTCTTA
ACGGAGGGCTACGTTAAGACCGGGATTTTCAAGCTTGCGATGTATCTGGCCCACCAACGACTACTGGATG
AAAATGTGGACGTGATTGTGCTGCTGATGCTTGAGCCTGTCCTGCAGCACTCACAGTTCCTGCGCCTCAG
GAGGAGGCTGTGCGGGAAAAGTGTTGTGGAGTGGCCGAGAACAGCGGCCGCGGAGCCCTGGTTCTGGGTA
AACCTGAGGAATGTCGTCAGGGTGGACAATCAAGTTATGTACAACAAGACTTATTCAAAGTACGGAGTCC
CATAAGATTCTAATGGTGATGTGCTACATACCGTGCATTTGCTGGATAGTAGCTGATACTTTGATGTACA
TCATGCAGAGTGGAACACAAGAAAGCCTTCCAAGGACTTGCACTGAGCTTTACGCCCACTTCTGTTCCAT
GAAAGCAGAGGTTGGCGAACCAAGAGGCAGGGAGAATGTAAAACTGGAGCAGCTTCATGGGAGTAATCGT
AAACTGCTTGGGAATCTTGGACGACTGGCCTTTTATGGGCTCCTCAAGCACAAGTACAGCTTCAGTGAGC
AGGACCTCAGGGCTTATGGAATAGATCTACTCTTGACTCAGAGCAGTCTTGGTACAGGAGTTCTTGTTCG
GGAGGAGACGGCCATATGCGTAACATACCGGTTCACTCATCTGACTCTGCAGGAGTTCCTTGCAGCAACT

TTCTACCATGTTTCCTCCAAGCGGGCAATCTTTGACTTGTTCTCGGAAAGCACCATGTCCTGGCCCAAAA
TTGGTTTCCAAAACCACTTCAGAAGTGCCTTTCAGCATGCACAACAAGCTGAAGATGGGCATTTGGATGT
GTTTGTGCGCTTTCTGACAGGCCTGCTGTGCCCAGCAGCACTGAAACCTCTTGCTGGGCTATTATCCCTC
GGGAAAGATGATGGTAATCAAAAGGCCTGGGCAGCAGGGTTTTTACAAAGCCTCTTGGTCAGCGGGGGTG
TGGTGGTGTCCCTGCGCACAGTCAACCTGGCTTATTGTTTACAGGAGCTGCAACACATAGAGCTGCTGCG
GAGTGTAGAGGAAGATTTAAGGCTTGGTAGCCTTGCAGGGAAATTAACACGGGCTCACTGTGTTGTGCTG
GGATATCTGCTGCACGTGTCTCCAGAGTGCAGTGAACAGACCAACCTAACAGGCTCTTTGAACTACGCCA
CAGTGAAATGTTTGCTCCCACAGCTGCTGTACTGCAGCAACCTCAGGTTAGAGAACAATCACTTCAAAGA
TGACGTCATGGAATTACTGGGGAGCCTGCTAAGTGCCAAAGACTGCCACATTCAGAGGATAAGTTTGGCA
GAGAACGCCATCAGCAACAAAGGTGCCAAAGCACTGAGTCGAGCCCTCCTGGTGAACCGGACTCTAACTT
CTCTGAATCTCCGGAACAACAACATCGGCTCTAAAGGTGCAAAGTTTCTGGCAGAAGCTCTGAAAATGAA
CCAAGTCCTGGTATCAATCAACTTTCAGAACAACGCCATTGAGGAGGAAGGGGCTCAAGCCCTCGCAGAA
GTGCTGCAGTGCAATCGCAAACTGGTGTCTCTGAATTTACGGAAGAACTCAGTCAGAGCAGGAGGAGCCA
AAGGGATTGCGGATGCGCTGAAGACAAACCGGACTCTTACCAAACTGATCCTTTGTAGCAACCAGCTTGG
AGATAAAGGAACAATTGCCCTGGCAGAGGCTTTGACTCACAACCACACTCTGCTCTCACTTCAACTTCAG
AGTAACTCAATCAGCAACAGGGGGATGACTGCCTTAACCAAAGCACTCAGGCTGAACCGAGGTCTTGTCT
CCTTAAATTTAAGGGAGAACTCGATCGGGGTGGAGGGAGCAAAGAACATGGCTCATGCACTCCATGAAAA
CAACTCTCTACAGGACCTTGATCTCACAGCCAACCTGTTGCACGATGAAGGAGTTCAAGCTATAGCAGGA
GTTCCTGGACGGTGATGAATGGAGAAGTCGCTGGGTGAACTCCAAACACAAGTCTGACTATGGAGAGTGG
AAACTGACGGCTGGGAGCTTCTACGGAGATGCAGAGAAAGACAAAGGTCTCCAGACCAGCCAGGATGCTC
GTTTCTATGCCTCCTCAGCCCGTTTTGAGCCTTTCAGCAACGAGGGCAAGTCTTTGGTCATTCAGTTTAC
TGTAAAGCACGAGCAGAAGATTGACTGCGGGGGTGGCTACGTGAAGGTCTTCCCTGCTGATTTGGATCAG
GCTGCGATGCATGGAGAATCCTCATACCACATCATGTTTGGTCCTGATATCTGTGGCTACAGCACAAAGA
AAGTCCATGTTATCTTCAATTACAAAGGCAAGAATCATCTAATCAAGAAAGAGATTAAGTGCAAGGATGA
TGAGCTGAGCCACCTGTACACACTGATCCTGAATCCAGATCAGACCTATGAGGTGAAAATTGACAATGAG
AAGGTGGAATCTGGCAGTCTGGAGGAAGACTGGGACTTCCTGCCTCCCAAGAAAGTAAAGGACCCTGAGG
CCAAGAAGCCAGAGGACTGGGATGATCGTGCCAAGATTGACGATGCTGATGACACCAAGCCTGAGGAGTG
GGACAAACCTGAAAACATTCCAGACCCTGATGCTAAAAAGCCTGAAGACTGGGACGAGGATATGGATGGA
GAGTGGGAGCCACCCATGATCCCCAACCCAGAGTACAAGGGAGAATGGAAACCCAAACAGATTGACAACC
CCAACTACAAAGGATCATGGGTGCATCCTGAGATTGACAATCCTGAATACAGCGCAGATTCCAGCATCTA
CAAGTTTGATAGCATTGGAGTTTTAGGTCTTGATCTGTGGCAGGTGAAATCCGGGACCATTTTTGACAAC
TTCCTCATCTCAGATGACGTGAAGGAAGCAGAAGACATAGGAAATGAGACATGGGGTGCAACAAAGGAAC
CAGAGAGGAAAATGAAGCAGGAGCAAGATGACCTGAAACGAAAAGAAGAGGAGGAGAAGAACAAAGAACA
AGATACTGAAGCTGATGAAGAAGAAGACGAAGAAGCTGCAGCAGATGAGGAAGAGGACGACGAAGAAGAG
GACGAGGAGGAAGATACAAAGGATGAAATGGAAGAGGCACTTTCAGAAATGGAGGAGGAGGAAGCAAAAC
TTAAAGATGAGCCCTCTAAGCAGAAGGTGCTGGTGATGGAGTATTGTTCTGGAGGGAGTTTGCTCAGCCT
GCTGGAGGAGCCTGAAAATGCCTTTGGCCTGCCAGAAACAGAGTTCCTCACTGTACTGGAGTGTGTAGTG
CAGGGGATGAACCACCTGCGTGAAAATGGAGTGGTACACCGGGACATTAAGCCGGGTAACATCATGAGGC
AAGTCGGGGAGGACAGGAAGTCTGTTTACAAGCTGACAGACTTTGGAGCAGCAAGAGAGCTGGATGATGA
TGAGAAGTTTATGTCCATCTATGGGACTGAAGAGTACCTGCATCCAGACATGTATGAGCGTGCTGTGCTC
CGCAAGCATCATCAGAAATCCTATGGAGTGAATGTGGACCTGTGGAGTATTGGTGTGACATTTTACCATG
CTGCCACTGGGAGTCTTCCCTTCACACCGTATGAAGGACCCCGCAGGAACAAGCCCACCATGTACAAAAT
AACGACAGAGAAACCAATGAGTGCAATAGCAGGGATTCAGCGTGTGGAGGGAGGACCTATAGAGTGGAGC
TACCACCTACCTCACAGCTGCCAACTGTCCCAGGGTCTGCAGGCGCAGCTAGTTCCAGTATTAGCAGGTG
TACTG

>Solea senegalensis tlr3-tlr8-nlrc3-calr-ikbke
CCTTTGCTGCTCTAAAGAACCTGAAGTTCCTCGATGTTTCCTCAAACCAGCTGCCATCAGCCAAACTGGG
CACTCAGCCTCAGCTACCCAGCCTGGTGGACCTCAGTCTGGCATTCAATGGATTCACCACACTGAGGAAG
GACGACTTCTCATTCCTGAACCATTCCCCTTTTCTGCAAACCCTCAATCTCTCGTCCGTGCCTCTAAAAA
CTTTGGAGCCTGGTTGCCTTGAGCCCATTTCAGGCCTGCGTACTTTAATTCTGGATGGGAGCAAAACTGA
CAGTTTGTTTATTTCCAAACTCTGCTCGGAGCTGGCAGGGACATCCATTGATGTCTTCTCTCTTCGGAGG
ACGACGCTAGTAACGCTCAAAAACACAACCTTTGCAGGACTGCAGAAAACAAATCTGACCTTTCTGGATT
TGTCCCAGAATGGCATGGGTACAATTGAGGATGGCTCATTTCAGTGGCTGTCCAAACTTCAGACTCTAAT
TTTGTCTGAGAACACCATCAAGCACCTTACGAAGTACACATTTCAAGGCCTCAAAAGTCTGAAACAACTC
AAAATGACAAAAGCTCTGGTGAAAGGTCATGCCACCCCGGTCATTGATGATTTCACTTTCCAACCGTTAA
GCTCCCTAGAGAGTTTAATATTACAGAGAACTGCAGTGAGGGAAATCACAGAGCACACATTTACAGGCCT
CACAAGTCTTAAAGAGATCGACATGAGTTGGGCCAGTTACTCATCACTCAGAATCATCACCGACAAGACC
TTGGTTTCACTTGCGGGATCTCCTCTCAGAGTGCTGAATCTGACTGCAACAGCTATAGCAAAGATTAATC
CTGGAAGCTTCTCATTTTTCAAAAACCTCACCATTCTTCTTCTAGATTTTAATTTCATACAGCAAACGCT
CTCTGGCAAAGAGTTTGAAGGCCTAAGTCAGGTCCAAGAGATTCACATGACCTACAACCACTGGAAAGTC

AGACTGTGCTCCACTTCATTTGTCAATGTGCCCAATCTTAGAGTCCTGACTTTGGGGAAAAGTCTTAACA
ACACAGCTGTGAACGTGGACGTGGATCCCTCACCATTCAAGCCCTTGTCCCACCTCACATTTTTGGATCT
CAGTAACAACAATATCGCTAATATCAGAGAGAGCATGTTGGATGGACTCGTGAACCTGAAGGTGCTGAAG
CTCCAACACAATAACTTGGCTCGCTTGTGGAAGAGTGCCAACCCAGGCGGGCCAGTGATGTTTCTCAAAG
GGGCACAGAACTTGATGACCTTACAGATGGACAGTAATGGGCTGGATGAAATCCCAGAGGGGGCTTTGAG
GGGTTTGATTAACCTCAGTGAACTAAGCCTCTCAAATAATCTCTTAAACAACCTGAGGGACTCCATTTTT
GAGGATCTGAAATCATTGCGGGTGTTACGTCTACAGAAGAACCTGATCACAGCTGTGAAGCCTGAAGTGT
TCCAAACTCCAATGAGCAACCTCAGCATACTTGTCATGGACAAAAACCCCTTTGACTGCACATGTGAGAG
CATCCTATGGTTTGTGACATGGCTGAATAGCACAAACAGGACATTTGTGCCTGATCTCACGGACCAGTAT
ATGTGCAACACTCCACTTGCTTACTATAATCATTCAATCACGATGTTTGACACACTGTCTTGCAAAGATA
TGACCCCATTTCAGGCTCTTTACATACTGAGCAGCACAGCTGTCATGGTGTTGATGGCAACAGCGTTTTT
GGTGCGATTCCAGGGCTGGAGAATTCAGTTTTATTGGAACATACTGGTCAGCCGCACGTTAGGATTTAGT
GATGCCAATGCTGAAGAGAGCAGGGAGTTTGAGTATGATGCCTACATCATACACACAGAGGAAGACAAGC
GCTGGGTGGACAGGATGATAGTTCCCTTAGAGAATGAAAAGTGCAGCTTTTACTTGGAGCATCGAGACGC
CATCCCTGGGACGCCACAGCTGAATTCCATCGTGGATAATATTAGACGGTCCAGGAAAATCGTGTTTGTC
ATCTCTGAAAGTCTTCTCAATGATCCCTGGTGTCAAGGATTTACAGCCCACCAGGCACTCCACCAGGTCA
TTGAAGCCAGCAGGGACTCTGTGGTCCTGGTCTTTCTGCAGGATGTGCACGACTACAAGTTATCTCGCTC
ACTCTTTATACGCAGAGGCATGTTGCGCTCGTGTTGTATCCTGGAGTGGCATTGCCATAAGGAGAGGATA
GCCGCCTTTCACCAGAGGCTCCTTATAGCACTTGGCATGACTAATCGATTAAGGGAGCTTGTCAAGCAAG
AGTGCTATAACTCTGGTCGAGTACTAATCCTCAGCTCCAATAATCTGTTCTTCATTTCTCCACAGCAGTT
TGTTGGCTATGGAAATATTGCATGTCTCAACCTCTCCGGAAATGGATTTTCAGCAGCACTTAATGGCACA
GAGTTCTCTTCGCTGCCGAATCTGACATACCTAGACCTATCTTTTAACAAGATTGATCTGGCCTATGACA
ATGCCTTCAAAGAACTACAAAATCTACAAGTGCTAGACCTCAGTTACAACTCACACTACTTTGAAGCATA
CGGGATAACGCATAATTTAAATTTCTTAAAAAAATCTGCCAGCACTGAGATGCTTAATATGAGTCACAAT
TCCATTTCCACATTAACAACAAAACAGCTGTGTAGCAAATCTTTGAGCGAACTTCAGTTTACACATAATA
ATCTTGGCTACATTTGGAAAGACAGAGATCGCGCATACAATAAGCTTTTCACTTCTCTTACTAATTTGAC
ATATCTAGATATATCCAAAAACCAAATTAAAAAGATCCCAGACCATATTTATCAATATTTACCACGTAAC
CTCACCAAACTATGCATAAGCAATAACTTGCTCACTGAATTTAAATGGGACGCATTAAACTATTTCCATC
AACTTCAAATTTTAGACCTGAGTTCCAATTCTTTAGTTTACGTGACAGGTATAAACTCAAGCATTACTCA
GACTTTGACTTTCCTCGATTTGAGTGGTAACCAAATTTTCCACTTGGATGACGGGCTCATAAATGGTCCG
AAAAGCCTTGAGACTCTTAGCCTTAGTGCCAACAAACTCACCACCATCAATCAATCGACCTTCCAACCAA
ACACCAATAATCAGATCCATACTTTGTACTTGCAGGGAAACCCATTTGAATGCACCTGTGATTCATTAGA
TTTTATCCTGTGGATGGAAAACAGTAAAATAAAAATCCCAAGCCTGACCACTGAGGTGACGTGCGACACA
CCAGAAAACCAGAAAGGTCGAGCATTGATATACTTTGACATTAACCAGTGTGTAAATGACAGCGAGGCCT
TCCTGTTATACATTCTCACAACTTTCTTCATCACTGCTTTTATGTTTGCGGCAACAGTTGCTCACCTATT
TTACTGGGATGCGTCATATGTAATACACTATATGAAAGCTAAGTTGAAGGGATACAAATCTTTGAACTCA
ACAGACAGTTACTATGGTGTCTTTGTGACATATGACACCAAAGATCCACATGTCTCTGAATGGGTAATGA
GAAATCTGCGGGTGAAACTGGAAGAGGAAGGAGAGAAACATCTCCCACTGTGTCTGGAGGAGAGGGACTG
GGTCCCAGGAGTCCCCCTGGTGGACAACCTCACTCATAGCATCAGATACAGTCGCAAGACCCTATTTGTC
TTAACAGAGGACTATGTTAAGACTGGGATTTTCAAGCTGGCAATGTATCTGGCCCACCAAAGGCTACTGG
ATGAAAATGTGGACGTGATTGTTCTGCTGATGCTCGAGCCAGTCCTGCAACACTCTCACTTCCTGCGCCT
GAGGAGAAGGGTGTGTGGAAAAAGTGTTGTAGAGTGGCCGAAGACAGCGGCCGCAGAGCCTTGGTTTTGG
CAAAACCTGAGGAATGTTGTCAGAGTGGACAATCAGTTTATGTACAACAAGATCTATTCAAAGTACGGAA
GCCCATAAGAACTTGATGGTGTTGTGCTACATACCATGCATCTGCTGGATTGTAGCTGATACTCTTGTGT
ACATCATGCAGAGTGGACCACAGGAAAGCCTTCCAAGGACTTACACTGAGCTCTACGCACATTTCTGTTC
CATGAAGGCAGAAGTAGGCGAACCAAGAGGGCGGGAGCCTGTGAAAATGGAGCAACTTCACGGGAGCAAT
CGGAAACTGCTGGGGAATCTCGGACGACTGGCATTTTATGGACTCCTCAAACACAGGTACACCTTCAGTG
AGCAGGACCTCAGGGCCTATGGGATAGATCTTCTGCTAACTCAAAGCAGTCTTGGTGCGGGGGTTCTTGT
TCGAGAGGAGTCGGCCGTTTGCACGACATACAAATTCATTCATCTGAGTTTGCAGGAGTTCCTCGCAGCC
ACATTCTACCATAACTCGTCCAAGCGGGCAATCTTTGACTTGTTCTCAGAAAGCAGTATGTCGTGGCCAA
AGATTGGTTTCCAGAACCATTTCAGAAGTGCCGTGCAGCACGCACAACAAGCGGAAGATGGACATTTGGA
TGTGTTTGTGCGCTTCCTCACAGGCCTTTTGTGCCCAGCAACACTGAGAAGTCTCGCTGGGCTTTTGTCC
CTTGGAAAAGATGACGGCAATCAGAAGGCCTGGGCAGCAGGGTTTCTGCAAGGCCTGTTGGTCAGCGGTG
GTGCGGTGGTGTCCCTGCGTACAATCAACCTGGCGTACTGTTTACAGGAGCTGCAGCACACAGAGTTGTT
GCGGAGTGTGGAGGAAGATCTGCGGCTTGGCAGCCTTGGAGGGAAGTTGTCGCGGGCTCAGTGCGTCGTG
CTGGGATACCTGTTGCACGTGTCTCCAGAGTGCAGTGAACACACCAACTTAACAGGCTCACTGAATTACA
CCACAGTGAAATGTTTGCTTCCACAGTTGCTGTACTGCAGCCATCTCAGGTTAGAGAATAACCATTTCAA
AGATGACGTCATGGAATTGCTGGGAAGCCTCCTGAGTGCCAAAGATTGCCATATTAACAAGTTAAGTTAT
GACCTCATTTATTCACCACTCAGTTTGGCAGATAACGCCATCAGCAACAAAGGAGCCAAAGCGCTGAGTC
GAGCTCTCCTGGTGAACCGAACACTAACATCACTCAGTCTCCAAAACAACAACATCGGCTCTAAAGGTGC
AAAGTTCTTGGCAGAAGCTCTGAAAATGAATCAAGTGCTGGTATCGGTCAACTTTCAGAACAACTCCATT

GAAGAGGAGGGCGCTCAGGCGCTCGCAGAAGTGCTGCAGTGCAACCGCAAACTGGTCTCTCTGAATCTGA
GGAAGAACACAGTCGGCGCAGGAGGAGCCAAAAGAATCGCAAATGCACTGAAGATGAACCAAACGCTCAC
AAACCTCATTCTTTGTAGTAACCACCTCGGGGACAAAGGAACGATCGCTCTGGCAGAGGCTTTGACGTTT
AACCACACGCTTCTCTCGCTTCACAACAGAGGGATGACTGCCTTAACCAGTGCACTCAGACTCAACCACG
GTCTCGTCTCCTTGAAGTGCGTATCAAAGCCTGTGACAGCGAACTCAATCGGAGTGGAAGGAGCAAAGAG
TATGGCACATGCTCTCCATGAGAACAACTCTCTACAAGACCTCGATCTTACAGCCAACCTGCTGCACGAT
GAAGGCGTTCAAGCTATAGCCGGTGCTTCTGTGTTCAGATGAATGGAGAAGTCGCTGGGTGAACTCCAAA
CACAAGTCTGACTATGGAGAGTGGAAACTGACCGCTGGTAACTTCTACGGAGATGCTGAGAATGACAAAG
GTCTGCAGACGAGCCAGGACGCTCGTTTCTATGCTGCCTCTGCACGCTTCGAGCCTTTCAGCAATGAGGG
CAAGAGCTTAGTCGTTCAGTTTACGGTCAAGCACGAGCAGAAGATCGACTGTGGCGGTGGCTACGTGAAG
GTGTTCCCTGCTGATTTAGAACAGACAGATATGCACGGAGATTCCACTTACTACATCATGTTTGGTCCTG
ATATCTGTGGCTACAGCACCAAGAAAGTCCACGTCATCTTCAATTACAAGGGCAAGAATCACCTCATCAA
GAAAGAGGTCAAGTGCAAGGATGATGAGCTGACCCACCTGTACACACTGATCCTGAATCCAGATCAGACC
TACGAGGTGAAAATTGACAATGAGAAGGTAGAATCTGGTAGTCTGGAGGAAGACTGGGACTTCTTGCCTC
CAAAGAAAATCAAAGATCCTGAGGCCAAGAAGCCAGAGGACTGGGATGACCGTGCCAAGATTGATGATCC
TGAAGATGCCATGCCTGAGGACTGGGAGAAACCTGAAAACATTCCTGACCCAGATGCTAAAAAGCCAGAA
GACTGGGACGAGGATATGGATGGAGAGTGGGAGCCGCCCATGATCCCCAACCCCGAATACAAGGGGGAAT
GGAAGCCCAAACAGATTGACAACCCCGACTACAAAGGAGCATGGGTGCATCCTGAGATAGACAATCCTGA
ATACAGTGCTGATTCCAACATCTACAAGTTTGACAACATTGGTGTTTTGGGTCTTGATCTTTGGCAGGTG
AAATCAGGCACCATCTTTGACAACTTCCTGATCTCAGATGACGTGAAGGAAGCAGAAGACATTGGAAATG
AGACATGGGGTGTGACAAAGGAACCAGAGAGGAAAATGAAGCAGGAGCAAGATGACCTGAAACAGAAAGA
GGAGCAGGAGAAGAACAAGGAACAAGATACTGAAGCTGAAGCTGACGATGATGAAAACGAAGATGTAAAA
GAGGAGGAAGACATGAAGGATGAATTAGAGGAGGCACTTTCAGAAATGGAGGATGAGGAAGCAAAGCTTA
AAGATGAGCCGTCGAAGCAGAAGGTGCTGGTGATGGAGTATTGTTCAGGAGGAAGTTTGCTGAGTCTGCT
CGAGGAACCAGAAAATGCCTTTGGCCTGCCTGAAACGGAGTTCCTCACTGTATTGCAGTGTGTCGTGCAG
GGGATGAACCACTTGCGTGAGAACGGCGTGGTTCACAGAGACATAAAGCCAGGCAACATCATGCGGCAGG
TCGGGGAGGACGGCAAGTCCGTTTATAAGCTGACGGACTTCGGAGCAGCAAGAGAGCTGGAGGATGATGA
GAAGTTTGTGTCCATCTACGGAACTGAAGAGTATCTGCATCCAGACATGTATGAACGTGCCGTCTTGCGT
AAGTCTCACCAGAAATCCTACGGAGTGAGTGTTGACTTGTGGAGTATTGGTGTGACATTTTATCACGCAG
CCACTGGGAGTCTCCCCTTTACACCATTTGAAGGACCTCGAAGGAACAAGCCAACCATGTATAAAATAAC
TACTGAGAAACCTATGGGCGCAATAGCTGGAATACAGCGGGTGGAGGGTGGACCTATCGAGTGGAGCTAC
CACCTTCCTCACAGCTGCCAACTGTCTCAGAGGTCTGAGGGTGCTGCTGGTTCCAGTGTTAGCAGGTATA
CTG

>Maylandia zebra tlr3-tlr8-nlrc3-calr-ikbke
CCTTCTCGTCACTGCAGAACCTAACCTTTCTAGATGTTTCTAAAAACAAACTGCAGTCACCCAAGCTCGG
CTCCCAGCCTCAGCTGCCCAGCCTGGTGAACTTCAGCCTGGCATTCAATGACTTCACCACTCTGAAGAAA
GATGACTTTTCATTTCTTGACCATTCATCCTCTCTGCAAGTCCTCAACCTGTCATCTGTGTTTTTAAAAA
CATTGGAGCCCGGCTGCTTTCAGCACATTTCAGGCCTGCGCACTTTAATCATGGATGGGGGCAACTTGGG
AACTCTGATGGTTTCCAAACTGTGCTCAGAGTTGTCTGGGACAGCCATCGATGCTCTCTCTCTTCGGAAA
ATGAGTCTAGTCATGGTCACAAACAAAGTCTTTACAGGACTGCAGAAGACAAATCTAACCTTCTTGGATC
TGTCCAGTAATGGCATGGGTAAAATTGAAGACGGCTCATTTCAGTGGCTATCTAAACTTCAAACTCTAAA
TTTGTCGCACAATAACATTAAGCACCTAACCAATGGGACATTTCAGGGGCTCAATAGATTGAAAAAACTG
GCACTTACAGAAGCACTGGTGAAAGGTCGTACCGCTGCCCCAGTTATTGATGATTTTGCTTTCCAGCCAT
TAGGCATGCTAGAGAGTTTGATGTTACAGAGGACTGCAATCCGGGAAATCGGAGAGCACACCTTTGCAGG
CTTAAAAAGTCTTAAAAAACTTGACATAAGCTGGATTAGCTGTCCGTCACTCCGAAACATCACCAACAAG
ACCTTAGCATCTCTTGCAGATTCACCAGTCAGATGGCTAAATCTTATAAATACAAATATAGCGCAGATCA
ATCCCGGAAGCTTCTCTGTTTTGCGAAACCTCACTGTTCTTCTTCTAGATTACAACCATATCAAGCAAAC
TTTGACAGGCAGAGAATTCGAAGGCCTAGATCAGGTTCAGGAAATTCACATGAGCAATAACTTCCAGTCA
ATTGACCTAAGCTCCAGTTCCTTTATTAATGTGCCCAACCTTAGAGTCCTGACTTTGGGGAGAAGTCTTA
AAGCTTTAGCCCTAAACCTAGATCACTCTCCATTCAAACCCCTGACCAACCTCTCTGTCTTGGATCTCAG
CAACAACAATGTTGCTAACATCAAAGACAATCTGCTAGAGGGGCTTGTAAACCTAAAGGTGTTGAAACTC
CAGCACAACAACTTGGCCCGACTGTGGAAGAGTGCCAACCTTGGTGGGCCGGTGTTGTTTCTCAAAGATG
CGCAGAGGTTGGAATCCTTACAGATGGATTATAATGGACTGGATGAGATCCCACTGAAGGCTTTGAAAGG
TTTGACTCACCTCAAGGAGCTAAGCCTTAGCAACAATCTCATAAATAATCTCAAGGACTCTGTTTTCGAT
GACCTGAAGTCGCTGCAGGTATTACGTTTTGAGAAGAACTTGATCACAAGCGTGAGACCTGAAGTGTTTC
GTACTCCCATGAGCAACCTCACCCAGCTTATCATGGGCAGAAATCCATTCGACTGCACATGCGAGAGCAT
CCTGTGGTTCGTGACGTGGTTGAATACCACAAATACGACGAGTGTGCCAAATGTCAGGGACGAGTATGTG
TGCAACACTCCACGAGCTTACTTTAACCACTCCATCATGGATTTCGACCCCCTTTCTTGCAAAGACATGA
CCCCATTTCAGACTCTTTACATAGTGAGCAGCACAGCCGTCATCTTGCTAATTGTAACCGCACTCACGGT
GCGGTTTCATGACTGGAGGATCCACTTTTACTGGAACATAATGATCAATCGCACATTAGGATTCAGTGAT

GCCAAAGTTGACGAAGGCAGAGAATATGAGTACGACGCTTATGTCATACGTACGGAGGAAGACTCCAGTT
GGGTGGAGAGGAGGTTGGTCCCTTTAGAGAATGAAAAGTGCCAGTTTTGTTTAGAGGATCGAGATTCAGT
CGCTGGCATGTCACAGGTGGAATCCATTGTGACAAATATGAAAAAGTCCAGAAAAATCATGTTTGTCGTC
ACCGAAAGTCTCCTCAAAGATCCCTGGTGTAGACGATTTAAAGTCTATCATGCACTTCAACAAGTCATCG
AAGAAAGCAGGGACTCAGTCATCCTGGTCTTTCTTCAGGATGTGCACGATCACAAGTTGTTTCACTCACT
CTTCCTCCGCAGAGGAATGTTACGTTCACGTTGCATTCTGGACTGGCCTGTCCATAAGGAAAGGATACCA
GCATTTCACCAGAAGCTTCTCATTGCACTCGGCTTGACTAATCGGCTGAAGGACCTAATAAAGCAAGAGT
GCTTTGACTCTGGACGAGTGCTCATCCTCAGCTCAAATAATCTGTTCTTCATTTCTCCAAAGCTATTTGA
GGGATATGGAAATATTTCATGTCTCAACCTCTCCAGAAATGGATTTTCACAGGCACTTAATGGCACAGAG
TTCAAGATGTTGCCTAATCTGACATATCTGGACTTATCTTTCAATAAGGTCGATATGGCCTATAACAACG
CCTTCAAAGAACTAAAGAAATTACAAGTACTAGACCTCAGTTACAATCCACACTACTTTAAAGCATTTGG
GGTAACGCTCAATTTAAATTTTACACAAAATCTACCTGTGCTAAGAGTCCTTAATATGAGTCATAATGAG
ATTTCCACACTGACAACAAAAGAGATGTATAGCAAATCATTAGCAGAACTTATATTTGCACAAAATCATC
TAGGGAAACTTTGGAAAGACAGGGATAGCACATACAAGAAACTTTTCACTAATCTCTCTAATTTGACAAT
TTTAGATATATCCTTTAATGGCATTGCAAAGATTCCAGATGATGTTTATGAATATTTGCCACATAACCTC
ACCACACTGCGCATAAGTCATAACTCACTCAGTGATGTTAATTGGGACAAACTGCCTTTCCATCAACTTC
AAATTTTGGACCTAAGTTACAATTCTCTGTCTACTTTAACAACTATTAACTCAAACATCACACAAACTTT
GACTTTCCTTGACCTGAGTCATAATCACATTTTCCATGCAGACAATTGTTTTTTAAAGAGTCTAAAAAGC
CTCACGACTCTAAGCCTGAGTAACAACAAATTGACTATTGTCAATGAAACTACCTTCAAATCAGGACCTG
CAAACCTGACTCTGTTCCTACAAGGAAATCCATTTGAGTGTACTTGTGATTCCATAGACTTTATTTTATG
GATTGAACAGAATGATGTAAAGATCCCAAGACTGACCACTCAGGTGACATGTAACACACCAGTGAACCAG
AGGAACAAAATACTGATAAGTTTTAACATTAGTCAGTGTGTAAATGCGAAACAGGCAAAGCAGATCTACG
CTCTAACAACTTCTATCATTATTCTTTTCATGATTGTTTCAACAGTTGCTCACTTATTTTACTGGGATGC
TTCCTATGTCCTACACTATATGAAAGCTAAGCTGAAAGGGTACAGATCCTTGAACTCGCCAGATACTATT
TATGATGTCTTTGTGACATATGATACAAAAGACCCACAGGTCTCTGAGTGGGTGATGAGCAATCTGCGGG
TGCAACTGGAGGAAGAGGGAGACAAGATACATCCACTGTGTCTGGAAGAGAGGGACTGGCCCCCAGGAGT
CCCACTGGTGGACAACCTCACCCAGAGCATCCAGTACAGTCGCAAGACTCTGTTTGTCTTAACAAAGGGC
TATGTTAAGACCGGTGCTTTCAAGCTAGCAATGTATCTGGCCCACCAAAGACTGCTGGATGAAAATGTGG
ATGTGATAGTACTGCTAATGCTGGAGCCTGTCCTGCAGCATTCTCACTTTCTGCGCTTGAGGAAGCGACT
GTGCGAGAGCAGTGTTGTAGAGTGGCCGCGAACAGCAGCCGCAGAGCCCTGGTTTTGGCAAAACCTGAGA
ACTGTCATAAGAGTAGATAATCAAGTGATGTACAACAAGACCTATTCAAAATACGGAATCCAATAAGATC
TTGATGGTGATGTGCTACATACCCTGCATTTGCTGGATAGTAGCTGATACTTTGTTATACATCATGCAGA
ATGAAGCACAGGAGAGTCTTCCGAGGACTTGCACTGAGCTGTATGCCAATTTCTGTTCAATGAAAGCAGA
ATTGGGCGAACCAAGAGGCAGAGAGCCTGTAAAAACGGAGCAACTTCACGGGAGCAATCGGAAACTGCTG
GGGAATCTCGGACGACTGGCTTTCTATGGGCTTCTTAAACACAAGTATGTCTTCAGTGAACAAGACCTCA
AGAACTATGGGATTGAGCTACTGCTGACTCAAAGCAGTCTCGGTTCTGGAGTTCTTGTTCGAGAGGAGTC
AACCATCAGCACGACGTTCCGCTTCACACATTTGACTCTGCAGGAGTTTCTTGCAGCTACCTTCTACCAT
GTTTCCTCAAAGCGGGCAATCTTTGACTTGTTCTCTGAGAGCTCCATGTCCTGGCCCAAGATTGGTTTCC
AGAACCACTTCAGAAGTGCCTTTCAGCACTCACAACAAGCTGAGGATGGTCACCTGGATGTGTTCGTACG
CTTCCTAACAGGCCTGCTGTGCTCAGCTGCAATCAAACCTCTTGCTGGACTTCTGGCCCTCGGGAAAGAC
GATGGCAACAACAAGGCATGGGCAGCAGGGTTTTTGCAGGGTCTGTTGGTCAGCGGGGGTGCTGTGGTGT
CCCTGCGTGCAGTCAACCTAGCTTACTGTTTACAGGAGCTGCAGCACACAGAGATGCTGCGGAGCGTAGA
GGAGGATTTACGGCTCAGCAGTCTAGCGGGAAAGTTAACGCGGCCTCACTGTGTGGTGCTGGGTTACCTG
CTCCACGTGTCTCCGGAGTGCAGCGAACAAACCAACCTGACAGCCTCTCTGAACGCCTCGACAGTGAAAT
GTTTGCTCCCGCAGCTGCTGTACTGTAGGCATCTAAGGTTGGAGAATAATCACTTTAAAGATGATGTCAT
GGAGTTGCTGGGAAGTCTCCTGAGTGCCAAAGACTGCCATATCCAAAAAATAAGTCTGGCGGACAATGCC
ATCAGCAACAAAGGAGCCAAAGCCCTGAGTCGAGCCCTCTTGGTGAACCGCACGCTGACTTCTCTCAATC
TTCGGAATAACAACATCGGCTCCAAAGGTGCAAAGTTCCTAGCAGAGGCTCTGAAAATGAACCAAGTCCT
GACATCAATCAACTTTCAGAACAACGCCATCGAGGAGGAGGGTGCTCAGTCGCTTGCAGAAGTGCTGCAG
TGCAACCGCAAACTGGTGTCTCTGAATATACGGAAGAATACAATCGCAGCAGGTGGAGCCAAAAGGATTG
CAGAAGCACTGAAGACCAACCGGACTCTCACAAAGTTGATCCTTTGTGGTAATCAGCTTGGGGATAAAGG
GACGGTAGCTCTGGCCGAGGCTTTGGCGGTCAACCACACTCTTCTCTCACTCCAACTTCAGAGTAACTCG
ATCAGCAACAAGGGCATGACAGCCTTAACCAAAGCACTGAGGCTGAACCATGGCCTTGTCTCCTTGAATC
TGAGGGAGAACTCCATTGGGGTAGAGGGAGCAAAGAACATGGCCCATGCCCTCCACGAGAACAACACTCT
GCAGGACCTCGATCTCACAGCCAACCTTTTGCATGATGACGGAGTTCAAGCTATCGCTAGAGTTTCTGGA
TGGTGATGAATGGAGAAGTCGCTGGGTGAACTCCAAGCACAAGTCAGACTACGGAGAGTGGAAGCTCACG
GCTGGCGACTTCTATGGCGATGCAGAGAAAGACAAAGGTCTGCAGACAAGCCAGGATGCACGTTTCTATG
CGGCCTCTGCCCGTTTTGATTCTTTCAGCAATGAGGGTAAGCCTTTGGTCATCCAGTTTACAGTCAAGCA
TGAGCAGAAAATCGACTGTGGTGGTGGCTACGTGAAGGTCTTTCCTGCGGACTTGGAGCAGACTGAAATG
CATGGAGAATCCTCTTACTACATTATGTTTGGCCCTGACATTTGTGGCTACAGCACCAAGAAAGTTCATG
TAATTTTTAATTACAAAGGCAAGAATAATCTCATTAAGAAAGAGATTAAATGCAAGGATGATGAGCTGAC

CCACCTGTACACACTAATCCTGAATCCAGATCAAACCTATGAAGTAAAAATCGATAATGAGAAGGTGGAA
TCTGGGAGTCTGGAGGATGATTGGGACTTTCTGCCTCCTAAGAAAATTAAGGACCCTGAAGCCAAGAAGC
CAGAGGATTGGGATGACCGTGCCAAGATTGATGATCCTAGTGACGCAAAGCCTGAGGACTGGGACAAGCC
CGAGAATATTCCAGATCCTGATGCTAAAAAGCCTGAAGACTGGGATGAGGATATGGACGGAGAGTGGGAG
CCACCCATGATCCCCAACCCAGAGTACAAAGGAGAATGGAAGCCCAAGCAGATTGATAACCCCAACTACA
AAGGAGCCTGGGTGCATCCTGAAATTGACAATCCTGAATACAGCCCAGACTCGAACATCTACAAGTTTGA
CAACATTGGTGTTTTGGGTCTTGATCTTTGGCAGGTGAAATCTGGTACAATCTTTGACAACTTCCTGATT
ACAGATGACGTGAAAGAGGCAGAAGAGATTGGAAAGGAGACATGGGGTGTGACAAAGGAGCCAGAGAGAA
AAATGAAACAGGAGCAAGATGACCTGAAACGAAAAGAGGAGGAGGAGAAGAACAAAGAGCAGGACACTGA
AGCTGCTGATGATGAAGATGAAGAAGAAGAAGAAGAGGAGGAGGAGGAAGAAGAAGAAACGAAAGAAGAC
ACAGACGAGGCACTTTCAGAAACAGATGAAGAAGAAGGAAAGCTTAAAGATGAGCCCTCTAAACAGAAGG
TGCTGGTGATGGAGTATTGTTCAGGAGGGAGTCTCCTAAGCCTGCTCGAGGAGCCGGAAAACGCCTTTGG
TCTCGCTGAAACAGAGTTCCTCACAGTATTGCAGTGTATAGTGCAAGGTATGAATCACCTGCGGGAAAAT
GGAGTGGTACACCGGGACATTAAGCCAGGGAACATAATGCGGCAGGTTGGGGAGGACGGCAGGTCTGTTT
ACAAGCTGACAGACTTTGGAGCAGCAAGAGAGCTCGAAGATGACGAGAAGTTTGTCTCCATTTATGGAAC
TGAAGAGTATCTGCACCCAGACATGTATGAGCGCGCAGTGCTACGTAAGCCTCATCATAAATCTTACGGG
GTGAGTGTCGACCTGTGGAGTATTGGTGTGACCTTATACCACGCTGCCACTGGGAGTCTTCCCTTCACAC
CATATGAAGGACCACGCAGGAACAAGCCCATTATGTTCAAAATAACCACAGAGAAACCTATGGGGGCGAT
AGCTGGAATACAACGGAGAGAGGGCGGACCTATAGACTGGAGCTATCACCTACCTCACAGCTGCCAGCTG
TCACAGGGTCTAAGGGTGCAGCTGGTTCCAGTACTAGCAGGTATAATG

>Pundamilia nyererei tlr3-tlr8-nlrc3-calr-ikbke
CCTTCTCGTCACTGCAGAACCTAACCTTTCTAGATGTTTCTAAAAACAAACTGCAGTCACCCAAGCTCGG
CTCCCAGCCTCAGCTGCCCAGCCTGGTGAACTTCAGCCTGGCATTCAATGACTTCACCACTCTGAAGAAA
GATGACTTCTCATTTCTTGACCATTCATCCTCTCTGCAAGTCCTCAACCTGTCGTCTGTGTTTTTAAAAA
CATTGGAGCCCGGCTGCTTTCAGCACATTTCAGGCCTGCGCACTTTAATCATGGATGGGGGCAACTTGGG
AACTCTGATGGTTTCCAAACTGTGCTCAGAGTTGTCTGGGACAGCCATCGATGCTCTCTCTCTTCGGAAA
ATGAGTCTAGTCATGGTCACAAACAAAGTCTTTACAGGACTGCAGAAGACAAATCTAACCTTCTTGGATC
TGTCCAGTAATGGCATGGGTAAAATTGAAGACGGCTCATTTCAGTGGCTATCTAAACTTCAAACTCTAAA
TTTGTCGCACAATAACATTAAGCACCTAACCAACGGGACATTTCAGGGGCTCAATAGATTGAAAAAACTG
GCACTTACAGAAGCACTGGTGAAAGGTCGTACCGCTTCCCCAGTTATTGATGATTTTGCTTTCCAGCCAT
TAGGCATGCTAGAGAGTTTGATGTTACAGAGGACTGCAATCCGGGAAATCGGAGAGCACACCTTTGCAGG
CTTAAAAAGTCTTAAAAAACTTGACATAAGCTGGATTAGCTGTCCGTCACTCCGAAACATCACCAACAAG
ACCTTAGCATCTCTTGCAGATTCACCAGTCAGATGGCTAAATCTTATAAATACAAATATAGTGCAGATCA
ATCCCGGAAGCTTCTCTGTTTTGCGAAACCTCACCGTTCTTCTTCTAGATTACAACCATATCAAGCAAAC
TTTGACAGGCAGAGAATTCGAAGGCCTAGATCAGGTTCAGGAAATTCACATGAGCAATAACTTCCAGTCA
ATTGACCTAAGCTCCAGTTCCTTTATTAATGTGCCCAACCTTAGAGTCCTGACTTTGGGGAGAAGTCTTA
AAGCTTTAGCCCTAAACCTAGATCACTCTCCATTCAAACCCCTGACCAACCTCTCTGTCTTGGATCTCAG
CAACAACAATGTTGCTAACATCAAAGACAATCTGCTAGAGGGGCTTGTAAACCTAAAGGTGTTGAAACTC
CAGCACAACAACTTGGCCCGACTGTGGAAGAGTGCCAACCTTGGTGGGCCGGTGTTGTTTCTCAAAGATG
CGCAGAGGTTGGAATCCTTACAGATGGATTATAATGGACTGGATGAGATCCCACTGAAGGCTTTGAAAGG
TTTGACTCACCTCAAGGAGCTAAGCCTTAGCAACAATCTCATAAATAATCTCAAGGACTCTGTTTTCGAT
GACCTGAAGTCGCTGCAGGTATTACGTTTTGAGAAGAACTTGATCACAAGCGTGAGACCTGAAGTGTTTC
GTACTCCCATGAGCAACCTCACCCAGCTTATCATGGGCAGAAATCCATTCGACTGCACATGCGAGAGCAT
CCTGTGGTTCGTGACGTGGTTGAATACCACAAATACGACGAGTGTGCCAAATGTCAGGGACGACTATGTG
TGCAACACTCCACGAGCTTACTTTAACCACTCCATCATGGATTTCGACCCCCTTTCTTGCAAAGACATGA
CCCCATTTCAGACTCTTTACATAGTGAGCAGCACAGCCGTCATCTTGCTAATTGTAACCGCACTCACGGT
GCGGTTTCATGACTGGAGGATCCACTTTTACTGGAACATAGTGATCAATCGCACATTAGGATTCAGTGAT
GCCAAAGTTGACGAAGGCAGAGAATATGAGTACGACGCTTATGTCATACGTACGGAGGAAGACTCCAGTT
GGGTGGAGAGGAGGTTGGTCCCTTTAGAGAATGAAAAGTGCCAGTTTTGTTTAGAGGATCGAGATTCAGT
CGCTGGCATGTCACAGGTGGAATCCATTGTGACAAATATGAAAAAGTCCAGAAAAATCATGTTTGTCGTC
ACCGAAAGTCTTCTCAAAGATCCCTGGTGTAGACGATTTAAAGTCTATCATGCACTTCAACAAGTCATCG
AAGAAAGCAGGGACTCAGTAATCCTGGTCTTTCTTCAGGATGTGCATGACCACAAGTTGTTTCACTCACT
CTTCCTCCGCAGAGGAATGTTACGTTCACGTTGCATTCTGGACTGGCCTGTCCATAAGGAAAGGATACCA
GCATTTCACCAGAAGCTTCTCATTGCGCTCGGCTTGACTAATCGGCTGAAGGACAATACAAAGCAAGAAT
GCGCCAATTCTGGACGAGTGCTCATCCTCAGCTCAAATAATCTGTTCTTCATTTCTCCAAAGCAGTTTGT
GGGCTATGGAAATATTTCATGTCTGAACCTCTCCAGAAATGGATTTTCACAGGCACTTAATGGCACAGAG
TTCAAGATGTTGCCTAATCTGACATATCTGGACTTATCCTTCAATAGGGTTGATCTGGCCTATAACAACG
CCTTCAAAGAACTAAAGAAATTACAAGTACTAGACCTCAGTTACAATTCACACTACTTTAAAGCATTTGG
GGTAACGCTCAATTTAAATTTTACACAAAATCTACCTGTGCTAAGAGTCCTTAATATGAGTCATAATGAG
ATTTCCACACTGACAACAAAAGAGATGTATAGCAAATCATTAGCAGAACTTATATTTGCACAAAATCATC

TAGGGAAACTTTGGAAAGACAGGGATAGCTCATACAAGAAACTTTTCACTAATCTCTCTAATTTGACAGT
TTTAGATATATCCTTTAATGGCATTGCAAAGATTCCAGATGATGTTTATGAATATTTGCCACATAACCTC
ACCACACTGCGCATAAGTCATAACTGCCTCAGTGATGTTAATTGGGACAAACTGCCTTTCCATCAACTTC
AAATTTTGGACCTAAGTTACAATTCTCTGTCTACTTTAACAACTATTAACTCAAACATCACACAAACTTT
GACTTTCCTTGACCTGAGTCATAATCACATTTTCCATGCAGACAATTGTTTTTTAAAGAGTCTAAAAAGC
CTCACGACTCTAAGCCTGAGTAACAACAAATTGACTATTGTCAATGAAACTACCTTCAAATCAGGACCTG
CAAACCTGACTCTGTTCCTACAAGGAAATCCATTCGAGTGTACTTGTGATTTGTTAGACTTCGTCTTGTG
GATTGAACAGAGTGATGTAAAGATCCCAAGACTCACCACTCAGGTGAATTGTAACACACCAGTGAACTTA
AAGGGACAAGGATTGATACATTTTAGCATTCATCAGTGTATAAATCCCAGTCAGGCATTTCAGATCTACA
CTCTAACAACTTCTATCATTATTCTTTTCATGGTTGTTTCAACAGTTGCTCACTTATTTTACTGGGATGC
TTCCTATGTCCTACACTATATGAAAGCTAAGCTGAAAGGGTACAGATCCTTGAACTCGCCAGATACTATT
TATGATGTCTTTGTGACATATGACACAAAAGACCCACACGTCTCTGAGTGGGTGATGAGCAATCTGCGGG
TGCAGCTGGAGGAAGAGGGAGACAAGATACATCCACTGTGTCTGGAAGAGAGGGACTGGCCCCCAGGAGT
CCCACTGGTGGACAACCTCACCCAGAGCATCCAGTACAGTCGCAAGACTCTGTTTGTCTTAACAAAGGGC
TACGTTAAGACCGGTGCTTTCAAGCTAGCAATGTATCTGGCCCACCAAAGACTGCTGGATGAAAATGTGG
ATGTGATAGTACTGCTAATGCTGGAGCCTGTCCTGCAGCATTCTCACTTTCTGCGCTTGAGGAAGCGACT
GTGCGAGAGCAGTGTTGTAGAGTGGCCGCGAACAGCAGCCGCAGAGCCCTGGTTTTGGCAAAACCTGCAA
AGTGTCATAAGAGTAGATAATCAAGTGATGTACAACAAGACCTATTCAAAATACGGAATCCAATAAGATC
TTGATGGTGATGTGCTACATACCCTGCATTTGCTGGATAGTAGCTGATACTTTGTTATACATCATGCAGA
ATGAAGCACAGGAGAGTCTTCCAAGGACTTGCACTGAGCTGTATGCCAATTTCTGTTCAATGAAAGCAGA
ATTGGGCGAACCAAGAGGCAGAGAGCCTGTAAAAACGGAGCAACTTCACGGGAGCAATCGGAAACTGCTG
GGGAATCTCGGACGACTGGCTTTCTATGGGCTTCTTAAACACAAGTATGTCTTCAGTGAACAAGACCTCA
AGAACTATGGGATTGATATACTGCTGACTCAAAGCAGTCTCGGTTCTGGAGTTCTTGTTCGAGAGGAGTC
AACCATCAACACGACGTTCCGCTTCACACATTTGACTCTGCAGGAGTTTCTTGCAGCTACCTTCTACCAT
GTTTCTTCAAAGCGGGCAATCTTTGACTTGTTCTCTGAGAGCTCCATGTCCTGGCCCAAGATTGGTTTCC
AGAACCACTTCAGAAGTGCCTTTCAGCACTCACAACAAGCTGAGGATGGTCACCTGGATGTGTTCGTACG
CTTCCTAACAGGCCTGCTGTGCTCAGCTGCAATCAAACCTCTTGCTGGACTTCTGGCCCTCGGGAAAGAC
GATGGCAACAACAAGGCATGGGCAGCAGGGTTTTTGCAGGGTCTGTTGGTCAGCGGGGGTGCTGTGGTCT
CCCTGCGTGCAGTCAACCTAGCGTACTGTTTACAGGAGCTGCAGCACACAGAGATGCTGCGGAGCGTAGA
GGAGGATTTACGGCTCAGCAGTCTAGCGGGAAAGTTAACGCGGCCTCACTGTGTGGTGCTGGGTTACCTG
CTCCACGTGTCTCCGGAGTGCAGCGAACAAACCAACCTGACAGCCTCTCTGAACGCCTCGACAGTGAAAT
GTTTGCTCCCGCAGCTGCTGTACTGTAGGCATCTAAGGTTGGAGAATAATCACTTTAAAGATGATGTCAT
GGAGTTGCTGGGAAGTCTCCTGAGTGCCAAAGACTGCCATATCCAAAAAATAAGTCTGGCGGACAATGCC
ATCAGCAACAAAGGAGCCAAAGCCCTGAGTCGAGCCCTCTTGGTGAACCGCACGCTGACTTCTCTCAATC
TTCGGAATAACAACATCGGCTCCAAAGGTGCAAAGTTCCTAGCAGAGGCTCTGAAAATGAACCAAGTCCT
GACATCAATCAACTTTCAGAACAACGCCATCGAGGAGGAGGGTGCTCAGTCGCTTGCAGAAGTGCTGCAG
TGCAACCGCAAACTGGTGTCTCTGAATATACGGAAGAATACAATCGCAGCAGGTGGAGCCAAAAGGATTG
CAGAAGCACTGAAGACCAACCGGACTCTCACAAAGTTGATTCTTTGTGGTAATCAGCTTGGGGATAAAGG
GACGGTAGCTCTGGCCGAGGCTTTGGCGGTCAACCACACTCTTCTCTCACTCCAACTTCAGAGTAACTCG
ATCAGCAACAAGGGCATGACAGCCTTAACCAAAGCACTGAGGCTGAACCATGGCCTTGTCTCCTTGAATC
TGAGGGAGAACTCCATTGGGGTAGAGGGAGCAAAGAACATGGCCCATGCCCTCCACGAGAACAACACTCT
GCAGGACCTCGATCTCACAGCCAACCTTTTGCATGATGACGGAGTTCAAGCTATCGCTAGAGTTTCTGGA
TGGTGATGAATGGAGAAGTCGCTGGGTGAACTCCAAGCACAAGTCAGACTACGGAGAGTGGAAGCTCACG
GCTGGCGACTTCTATGGCGATGCAGAGAAAGACAAAGGTCTGCAGACAAGCCAGGATGCACGTTTCTATG
CGGCCTCTGCCCGTTTTGATTCTTTCAGCAATGAGGGTAAGCCTTTGGTCATCCAGTTTACAGTCAAGCA
TGAGCAGAAAATCGACTGTGGTGGTGGCTACGTGAAGGTCTTTCCTGCGGACTTGGAGCAGACTGAAATG
CATGGAGAATCCTCTTACTACATTATGTTTGGCCCTGACATTTGTGGCTACAGCACCAAGAAAGTTCATG
TAATTTTTAATTACAAAGGCAAGAATAATCTCATTAAGAAAGAGATTAAATGCAAGGATGATGAGCTGAC
CCACCTGTACACACTAATCCTGAATCCAGATCAAACCTATGAAGTAAAAATCGATAATGAGAAGGTGGAA
TCTGGGAGTCTGGAGGATGATTGGGACTTTCTGCCTCCTAAGAAAATTAAGGACCCTGAAGCCAAGAAGC
CAGAGGATTGGGATGACCGTGCCAAGATTGATGATCCTAGCGACGCAAAGCCTGAGGACTGGGACAAGCC
CGAGAATATTCCAGATCCTGATGCTAAAAAGCCTGAAGACTGGGATGAGGATATGGACGGAGAGTGGGAG
CCACCCATGATCCCCAACCCAGAGTACAAAGGAGAATGGAAGCCCAAGCAGATTGATAACCCCAACTACA
AAGGAGCCTGGGTGCATCCTGAAATTGACAATCCTGAATACAGCCCAGACTCGAACATCTACAAGTTTGA
CAACATTGGTGTTTTGGGTCTTGATCTTTGGCAGGTGAAATCTGGTACAATCTTTGACAACTTCCTGATT
ACAGATGACGTGAAAGAGGCAGAAGAGATTGGAAAGGAGACATGGGGTGTGACAAAGGAGCCAGAGAGAA
AAATGAAACAGGAGCAAGATGACCTGAAACGAAAAGAGGAGGAGGAGAAGAACAAAGAGCAGGACACTGA
AGCTGCTGATGATGAAGATGAAGAAGAAGAAGAGGAGGAGGAGGAAGAAGAAGAAACGAAAGAAGACACA
GACGAGGCACTTTCAGAAACAGATGAAGAAGAAGGAAAGCTTAAAGATGAGCCCTCTAAACAGAAGGTGC
TGGTGATGGAGTATTGTTCAGGAGGGAGTCTCCTAAGCCTGCTCGAGGAGCCGGAAAATGCCTTTGGTCT
CGCTGAAACAGAGTTCCTCACAGTATTGCAGTGTATAGTGCAAGGTATGAATCACCTGCGGGAAAATGGA

GTGGTACACCGGGACATTAAGCCAGGGAACATAATGCGGCAGGTTGGGGAGGACGGCAGGTCTGTTTACA
AGCTGACAGACTTTGGAGCAGCAAGAGAGCTCGAAGATGACGAGAAGTTTGTCTCCATTTATGGAACTGA
AGAGTATCTGCACCCAGACATGTATGAGCGCGCAGTGCTACGTAAGCCTCATCATAAATCTTACGGGGTG
AGTGTCGACCTGTGGAGTATTGGTGTGACCTTATACCACGCTGCCACTGGGAGTCTTCCCTTCACACCAT
ATGAAGGACCACGCAGGAACAAGCCCATTATGTTCAAAATAACCACAGAGAAACCTATGGGGGCGATAGC
TGGAATACAACGGAGAGAGGGCGGACCTATAGACTGGAGCTATCACCTACCTCACAGCTGCCAGCTGTCA
CAGGGTCTAAGGGTGCAGCTGGTTCCAGTACTAGCAGGTATAATG

>Oreochromis niloticus tlr3-tlr8-nlrc3-calr-ikbke
CCTTCTCGTCACTGCAGAACCTAACCTTTCTAGATGTTTCCAAAAACAAACTGCAGTCACCCAAGCTCGG
CTCTCAGCCTCAGCTGCCCAGCCTGGTGAACTTCAGCCTGGCATTCAATGAGTTCACCACTCTGAAGAAA
GATGACTTTTCATTTCTTGACCATTCATCCTCTCTGCAAGTCCTCAACCTGTCATCTGTGCCTTTAAAAA
CATTGGAGCCCGGCTGCTTTCAGGCCATTTCAGGCCTGCGCACTTTAATCATGGATGGGGGCAACTTGGG
AACTCTGATGGTTTCCAAACTGTGCTCAGAGTTGTCTGGGACAGCCATTGATGCTCTCTCTCTTCGGAAA
ATGAATCTGGTCATGGTCACAAACAAAACCTTTACGGGACTGCAGAAGACAAATCTAACCTTCTTGGATC
TGTCCAGTAATGCCATGGGTCAAATTGAAGAAGGCTCATTTCAGTGGCTATCTAATCTTCAGACTCTAAA
TTTGTCGCACAATAACATCAAGCACCTGACCAACAGGACATTTCAGGGGCTCAGTAGATTGAAAAAACTG
GCACTTACAGAAGCACTGGTGAAAGGTCGTACCTCTTCCCCAGTTATCGATGATTTTGCTTTCCAGCCAT
TAGGCATGCTAGAGAGTTTGATGTTACAGAGGACTGCAATCAGGGAAATCGGAGAGCACACCTTTGCAGG
CTTGAAAAGTCTTAAAAAACTTGACATAAGCTGGATTAACTGTCCGTCACTCCGAAACATCACCAACAAG
ACCTTAGCGTCTCTTGCAGATTCACCAGTCAGATGGCTAAATCTCATAAATACAAATATAGCGCAGATCA
ATCCCGGAAGCTTCTCTGCTTTGAGAAACCTCACCGTTCTTCTTCTAGATTACAACCATATCAAGCAAAC
TTTGACAGGCAGAGAATTCGAAGGCCTAGATCAGGTTCAGGAGATTCACATGAGCAATAACTTCCAGTCA
ATTGACCTAAGCTCCAGTTCCTTTGTTAATGTGCCCAACCTTAGAGTCCTGACTTTGGGGAGAAGTCTTA
AAGCTTTAGCCCTAAACCTAGATCACTCTCCATTCAAACCCCTGACCAACCTCTCTGTCTTGGATCTCAG
CAACAACAATGTTGCTAACATCAAAGACAATCTGCTAGAGGGGCTTGCAAACCTAAAGGTGTTGAAACTC
CAGCACAACAACTTGGCCCGACTGTGGAAGAGTGCCAACCTTGGTGGGCCGGTGTTGTTTCTCAAAGATG
CGCAGAGGTTGGAATCCTTACAGATGGATTATAATGGACTGGATGAGATCCCACTGAAGGCTTTGAAAGG
TTTGACTCACCTCAGGGAGCTAAGCCTTAGCAACAATCTCATAAACAGTCTCAAGGACTCTGTTTTCGAT
GACCTGAAGTCACTGCAGGTATTACGTTTTGAGAAGAACTCGATCACAAGCGTGAGACCTGAAGTGTTTC
GTACTCCCATGAGCAACCTCACCCAGCTTATCATGGGCAGAAATCCATTCGACTGCACGTGCGAGAGCAT
CCTGTGGTTCGTGACGTGGTTGAATACCACAAATACGACGAGTGTGCCAAATGTCAGGGACGAGTATGTG
TGCAACACTCCACGAGCTTACTTTAACCACTCCATCATGGATTTCGACCCCCTTTCTTGCAAAGACATGA
CCCCATTTCAGACTCTTTACATAGTGAGCAGCACAGCCGTCATCATGCTAATTGTAACCGCACTCACGGT
GCGGTTTCATGGCTGGAGGATCCACTTTTACTGGAACATAATGATCAATCGCACATTAGGATTCAGTGAT
GCCAAAGTTGACGAAGGCAGAGAATATGAGTACGACGCTTATGTCATACGTGCAGAGGAAGACTCCAGTT
GGGTGGAGAGGAGGTTGGTCCCTTTAGAGAATGAAAAGTGCCAGTTTTGTTTAGAGGATCGAGATTCAGT
CGCTGGCATGTCACAGGTGGAATCCATTGTGACAAATATGAAAAAGTCCAGAAAAATCATGTTTGTCGTC
ACCGAAAGTCTCCTCAAAGATCCCTGGTGTAGACGATTTAAAGTCTATCATGCGCTTCAGCAAGTCATCG
AAGAAAGCAGGGACTCAGTCATCCTGGTCTTTCTTCAGGATGTGCACGACCACAAGTTGTTTCACTCACT
CTTCCTCCGCAGAGGAATGCTACGTCCACGTTGCATTCTGGACTGGCCTGTCCATAAGGAAAGGATAGCA
GCATTTCACCAGAAGCTTCTCATTGCGCTCGGCTTGACTAATCGGCTGAAGGACAATACAAAGCAAGAAT
GCGCTAACTCTGGACGAGTGCTCATCCTCAGCTCAAATAATCTGTTCTTCATTTCGCCAAAGCAGTTTGA
GGGATATGGAAATATTTCATGTCTAAACCTCTCCAGAAATGGATTTTCACAGGCACTTAATGGCACAGAG
TTCAAGATGTTGCCTAATCTGACATATCTGGACTTATCCTTCAATAGGGTTGATCTGGCCTATAACAACG
CCTTCAAAGAACTAAAGAAATTACAAGTACTAGACCTCAGTTACAATTCACACTACTTTGAAGCATTTGG
GGTAACGCTTAATTTAAATTTTACACAAAATCTACCTGTGCTAAGAGTGCTTAATATGAGTCATAATGAG
ATTTCCACACTGACAACTAAAGAAATGTATAGCAAATCATTAGCAGAACTTATATTTGCACAAAATCATC
TAGGGAAACTTTGGAAAGACAGAGATGGCTCATACAAGAAACTTTTCACTAATCTTTCTAATTTGACAGT
TTTAGATATATCCTCTAATGGCATTGCAAAGATTCCAGATGATGTTTATGAATATTTGCCACATAACCTC
ACCACACTGCGCATAAGTCATAACTTACTCAGTGATGTTAATTGGGACAAACTGCCTTTCCATCAACTTC
AAATTATGGACCTAAGTTACAATTCTCTGTCTACTTTAACAACTATTAACTCAAACATGGCCCAAACTTT
GACTTTCCTTGACCTGAGTCATAATCACATTTTCCATGCAGACAATTGTTTTTTAAAGAGTCTAAAAAGC
CTCATGACTCTCAGCCTGAGTAACAACAAATTGACTATTGTCAATGAAACTACCTTCAAATCAGGACCTG
CAAACCTGACTCTGTTCCTACAAGGAAATCCATTTGAGTGTACTTGTGATTTGTTAGACTTCGTTTTATG
GATTGAACAGAGTGATGTAAAGATCCCAAGACTGACCACTCAGGTGACATGTAACACACCAGTGAACTTA
AAGGGAGAAGGATTGATACATTTTAGCATTCATCAGTGTGTAAATCCCAGTCAGGCATTCCAGATCTACA
CTCTAACAACTTCTATCATTATTCTTTTCATGATTGTTTCAACAGTTGCTCACTTATTTTACTGGGATGC
TTCCTATGTCCTACACTATATAAAAGCTAAGCTGAAAGGGTACACATCCTTTAACTCGCCAGATACTATT
TATGATGTCTTTGTGACATATGACACAAAAGACCCACAGGTCTCTGAGTGGGTGATGAGCAATCTGCGGG
TACAACTGGAGGAAGAGGGAGACAAGTATCATCCATTGTGTCTGGAAGAGAGGGACTGGCCCCCAGGAGT

CCCACTGGTGGACAACCTCACCCAGAGCATCCAGTACAGTCGCAAGACTCTGTTTGTCTTAACAAAGGGC
TACGTTAAGACCGGTGCTTTCAAGCTGGCAATGTATATGGCCCACCAAAGACTGCTGGATGAAAATGTGG
ATGTGATAGTACTGCTAATGCTGGAGCCTGTCCTGCAGCATTCTCACTTTCTGCGCTTGAGGAAGCGACT
GTGTGAAAGCAGTGTTGTAGAGTGGCCACGAACAGCAGCAGCAGAGCCCTGGTTTTGGCAAAACCTGCGA
AGTGTCATAAGAGTAGATAATCAAGTGATGTACAACAAGACTTATTCAAAATACGGAATCCAATAAGATC
TTGATGGTGATGTGCTATATACCCTGTATTTGCTGGATAGTAGCTGACACTTTGTTATACATCATGCAGA
ATGAAGCACAGGAGACTCTTCCGAGGACTTGCACTGAGCTGTATGCCAATTTCTGTTCAATGAAAGCAGA
ATTGGGCGAACCAAGAGGCAGAGAGCCTGTAAAAATGGAGCAACTTCACGGGAGCAATCGGAAACTGCTG
GGGAATCTCGGACGACTGGCTTTCTATGGGCTTCTTAAACACAAGTATGTCTTCAGTGAACAAGACCTCA
AGAACTATGGGATTGAGCTACTGGTCACTCAAAGCAGTCTCGGTTCTGGAGTTCTTGTTCGGGAGGAGTC
AACCATCAACACGACGTACCGCTTCACACATTTGACTCTGCAGGAGTTTCTTGCAGCAACTTTCTACCAT
GTTTCCTCAAAGCGGGCAATCTTTGACTTGTTCTCTGAGAGTTCCATGTCTTGGCCCAAGATTGGTTTCC
AGAACCACTTCAGAAGTGCCTTTCAGCACTCACAACAAGCTGAGGATGGTCACCTGGATGTGTTCGTACG
CTTCCTAACAGGCCTGCTGTGCTCAGCTGCAATCAAACCTCTTGCTGGACTTCTGGCCCTCGGGAAAGAC
GATGGCAACAACAAGACATGGGCAGCAGGGTTTTTGCAGGGTCTGTTGGTCAGCGGGGGTGCTGTGGTCT
CCCTGCGTGCAGTCAACCTAGCTTACTGTTTACAGGAGCTGCAGCATACAGAGATGCTGCGGAGCGTAGA
GGAGGATTTACGGCTCGGCAGTCTAGCGGGAAAGTTAACGCGGCCTCACTGTGTGGTGCTGGGTTACCTG
CTCCACGTGTCTCCGGAGTGCAGCGAACAAACCAACCTGACAGCCTCTCTGAACGCCTCGACAGTGAAAT
GTTTGCTCCCGCAGCTGCTGTACTGTAGCCATCTAAGGTTGGAGAATAATCACTTTAAAGATGATGTCAT
GGAGTTGCTGGGAAGTCTCCTGAGTGCCAAAGACTGCCATATCCAAAAAATAAGTCTGGCGGACAATGCC
ATCAGCAACAAAGGAGCCAAAGCCCTGAGTCGAGCCCTCTTGGTGAACCGCACGCTGACTTCTCTCAATC
TCCGGAATAACAACATCGGCTCCAAAGGTGCAAAGTTCCTAGCAGAGGCTCTGAAAATGAACCAAGTCCT
GACATCAATCAACTTTCAGAACAACGCCATCGAGGAGGAGGGTGCTCAGTCGCTTGCAGAAGTGCTGCAG
TGCAACCGCAAACTGGTGTCTCTGAATTTACGGAAGAATACAATCGCAGCAGGCGGAGCCAAAAGGATTG
CAGAAGCACTGAAGACCAACCGGACTCTCACAAAGTTGATTCTTTGTGGTAATCAGCTTGGGGATAAAGG
GACGGTAGCTCTGGCCGAGGCTTTGGCGGTCAACCACACTCTTCTCTCACTCCACCTTCAGAGTAACTCG
ATCAGCAACAAGGGAATGACAGCCTTAACCAAAGCACTAAGGCTGAACCATGGCCTTGTCTCCTTGAATC
TGAGGGAGAACTCCATTGGGGTAGAGGGAGCAAAGAACATGGCCCATGCCCTCCATGAGAACAACACTCT
GCAGGACCTCGATCTCACAGCCAACCTTTTACATGATGACGGAATTCAAGCTATCGCTAGAGTTTCTGGA
TGGTGATGAATGGAGAAGTCGCTGGGTGAACTCCAAGCACAAGTCAGACTATGGGGAGTGGAAACTCACG
GCTGGCGACTTCTATGGCGATGCAGAGAAAGACAAAGGTCTGCAGACAAGCCAGGATGCACGTTTCTATG
CCGTCTCTGCCCGTTTTGATTCTTTCAGCAATGAGGGTAAGCCTTTGGTCATCCAGTTTACAGTCAAGCA
TGAGCAGAAAATCGACTGTGGTGGTGGATACGTGAAGGTCTTTCCTGCGGACTTGGAACAGACTGAAATG
CATGGAGAATCCTCTTACTACATTATGTTTGGCCCTGACATTTGTGGCTACAGCACCAAGAAAGTTCATG
TAATTTTTAATTACAAAGGCAAGAATCATCTCATCAAGAAAGAGATTAAATGCAAGGATGATGAGCTGAC
CCACCTGTACACACTAATCCTGAATCCAGATCAGACCTATGAAGTAAAAATCGACAATGAGAAGGTGGAA
TCTGGCAGTCTGGAGGAGGATTGGGACTTTCTGCCCCCTAAGAAAATTAAGGACCCTGAAGCCAAGAAGC
CAGAGGATTGGGATGACCGTGCCAAGATTGATGATCCCAGTGACACCAAGCCTGAGGACTGGGACAAGCC
CGAGAATATTCCAGATCCTGATGCTAAGAAGCCTGAAGACTGGGATGAGGATATGGACGGAGAGTGGGAG
CCACCCATGATCCCCAACCCAGAGTACAAAGGAGAATGGAAGCCCAAGCAGATTGATAACCCCAACTACA
AAGGAGCCTGGGTGCATCCTGAAATTGAAAATCCTGAATACAGCCCAGACTCGAACATCTACAAGTTTGA
CAACATTGGTGTTTTGGGTCTTGATCTTTGGCAGGTGAAATCTGGTACAATCTTTGACAACTTCCTGATC
ACAGATGACGTGAAAGAGGCAGAAGAGATTGGAAAGGAGACATGGGGTGTGACAAAGGAGCCAGAGAAAA
AAATGAAACAGGAGCAAGATGACCTGAAACGAAAAGAGGAGGAGGAGAAGAACAAAGAACAGGACACTGA
AGCTGCTGCTGATGAAGATGAAGAGGAGGAGGAAGAGGAGGAGGAGGAAGAAACACAAGAAGACACAGAT
GAGGCACTTTCAGAAACGGATGAAGAAGATGCAAAGCCTAAAGATGAGCCCTCTAAACAAAAGGTGCTGG
TGATGGAGTATTGTTCAGGAGGGAGTCTCCTAAGCCTGCTCGAGGAGCCGGAAAACGCCTTTGGTCTCGC
TGAAACAGAGTTCCTCACAGTATTGCAGTGTATAGTGCAAGGTATGAATCACCTGCGGGAAAATGGAGTG
GTGCACCGGGACATTAAGCCAGGGAACATCATGCGGCAAGTTGGGGAGGACGGCAGGTCTGTTTACAAGC
TGACAGACTTTGGAGCAGCAAGAGAGCTCGAAGATGACGAGAAGTTTGTCTCCATTTATGGAACCGAAGA
GTATCTGCACCCGGACATGTATGAGCGTGCGGTGCTACGTAAGCCTCATCATAAATCTTACGGGGTGAGT
GTCGACCTGTGGAGCATTGGTGTGACCTTATACCACGCTGCCACTGGGAGTCTTCCCTTCACACCATATG
AAGGACCACGAAGGAACAAGCCCATTATGTTCAAAATAACCACAGAGAAACCTATGGGGGCGATAGCTGG
AATACAACGGGCAGAGGGCGGACCTATAGAGTGGAGCTATCACCTACCTCACAGCTGCCAGCTGTCACAG
GGTCTAAGGGTGCAGCTGGTTCCAGTACTAGCAGGTATAATG

>Stegastes partitus tlr3-tlr8-nlrc3-calr-ikbke
CGTTCTCTGGGCTGCAGAGCCTGAAATTTCTTGATGTTTCCATAAACAAACTGCAGTCAGCCACGCTTGG
CTCTCAGCCACAACTGCCCAGATTAGTGAAGCTCAATCTGGCATTCAACGAATTCACCGCTCTGAAGAGA
GAAGACTTTTCCTTCCTCAACCAGTCATCATTTCTACAACTCCTCAACCTGTCATCAGTGCCTCTAAACA
CACTGGAGACGGGTTGCTTTAAGCCTATTTCTGGCCTACGTACTTTAATACTGGATGGGAGCAAAATAGG

CACTCGGGTCATTTCCAAAGTCTGCTCAGCCCTTTCTGATACCGCCATCGATGCCCTGTCTCTTAAGAAG
ATGAATCTGATCACGCTGACAAACACAACCTTCATTGGACTGAACAAAACAAATGTAACCTTTCTGGATC
TGTCCAGTAATAGCATGAGTAAAATTGAAGATGGCTCATTCCAGTGGCTGCCTAAACTTCAAACTCTGAT
TATGTCCGACAACAACCTCAAGCGCCTGACCAACAGCTCATTTATGGGGCTCGAAAGTTTAAAAAAACTC
CAGCTGACAAAAGCACTGGTGAAAAGCTCCTCCAGCCCCATTATCAATGACTTCTCTTTCCAACCATTAA
GCTCCCTGGAGAGTTTGATATTAGCAGGAAATAAAGCTCTGAGGATCACAGAGCACACCTTTACAGGCCT
GACAAGTCTTACAGAACTTGATCTGAGCGGGAGTAGTTACACGTCACTCAAATACATGACCAACAGGACC
TTAATCTCACTTGCGGGTTCACCGCTTACAAAGCTGAATCTGATAGGAACAGATATAAACCTCATTAATA
ATGGAAGCTTCTCGTTTTTGAGAAACCTCACCATTCTTCTTTTAGATTACAACTTTATCAGGCAAAGTCT
CACTGGTGGAGAATTTGAAGGCCTGGATAAGGTTCAGGAGATTCACATGAGCAATAACTATCAGTCAGTT
ACCCTGAGTTCTGGATCATTTGTTGGCGTGCCCGGTCTTAGAGTCCTGACTTTGGGAAAAAGTCTGAGAG
CTGAAGCTTTGAACCAGGATCCCTCTCCATTCAGGTTCCTGTCCAACCTTACCATCTTGGATCTGAGCAA
CAACAACATTGCAAACATCAAAGAAAGTATGCTGGAGGGCACTGTGAACCTGAAGGTATTGAAACTCCAA
CATAACAACTTAGCCCGGCTGTGGAAGAGTGCCAACCTTGGTGGGCCAGTGTTTTTTCTCAAGTATACGC
GGAATCTGATAAACTTACAGCTGGACTATAACGGATTTGATGAGATTCCAGTGGAGGCTCTCAGAGGTTT
GACCAGCCTCGAGGAGCTAAGCCTCACCAACAATCTCTTAAACAGTCTGAAGGACTCCGTTTTTGATGAT
CTGACCTCACTGCGGGTTTTACGTTTACAGAAGAACCTGATCACAGCAGTGAGGCCTGAAGTGTTCAAAA
CTCCTATGAGCAACCTCAGCCTGCTTGTCATGGAAAAAAATCCGTTTGACTGCACGTGTGACAGCATCCT
GTGGTTCACGACATGGTTGAACAAAACAAATATGACCAGTGTGCCGGGTCTCAAGGACCAGTATAAGTGC
AACACCCCGCTAACTTACTTCAACCGCTCCATCATGGATTTTGACCCCCTCTCTTGCAAAGACATGACCC
CATTTCAGGCTCTTTACATACTGAGCAGCACAGCTGTCATCACGCTGATTGTGATCGCACTTCTGGTGCG
GTTCCATGGCTGGAGGATTCAGTTCTATTGGAATATACTGATCAATCGCACACTAGGATATAGTGATGCC
AAACTTGAAGAGGGCAGAGAATTTGAGTATGATGCTTACGTCGTATATGCAGACAGAGACGCCAGCTGGG
TGGAGAGAAGGCTGGTCCCTTTGGAGAATGAAATGTGTCGGTTTTGTTTGGAGGATCGAGACTCAGTTAT
TGGCATGCCACAGCTCGAGTCCATCGTGGATAATATGAACAAGTCCAGAAAAATCCTGTTTGTCGTCACT
GAAAGTCTTCTCAAAGATCCCTGGTGTAGACGATTTAAAGTCTACCAGGCACTTCACCAGGTCATCGAAG
CCAGCAGGGACTCTGTTGTTCTGGTCTTTCTACAGGATGTGCACGACTACAAGTTGTCTCGCTCCCTGTT
CCTCCGCAGGGGCATGTTGCGTTCATGTTGCGTCCTGGACTGGCCTGTCCATAAGGAGAGAGTACCGGCC
TTTCACCAGAAGCTCCTCATTGCGCTCGGCATGACTAACCGACTGCAGGACCTTATAAAGCAAGAGTGCT
TTGACTCTGGACGAGTGCTAATCCTCAGCTCAAATAATCTCTTCTTCATTTCTCCACACCAGTTCGAGGG
CTATGGAGATATCGCGTGCCTCAACCTCTCAGGAAATGGATTTTCAGCTGCACCTAATGGCACAGAGTTC
TCCAGCCTGCCTAATCTAACATACCTGGACCTGTCCTACAATAAGATAGATCTGGCCTATGACAACGCCT
TCAAAGAGCTCCAGAAACTACAAGTGCTAGACCTCAGTTACAATGAGCACTACTTTCAAGCTTTTGGGAT
TACACACAATTTAGATTTTACAAAAAATCTGCCCGTTCTGAGAGTGCTGAATATGAGCCACAACTCCATT
TTCACACTGACAACAAAAAAGATGTACAGCCAATCATTAGCAGAGCTTCAGTTTACATATAATCATCTTG
GGACTCTTTGGAAAGAAAAGGATGGCTCTTATAAGACACTTTTTACTAATCTTATCAATTTGACACTGTT
GGATATATCTCACAACAACATTGCAAAGATTCCAGATGACGTTTATGAACATTTACCATATAACCTCACC
ACATTACACATCAGTCATAACAAACTGATTGAGTTTGCATGGCACAACCTAAAGTATTTCCATCAACTTC
AAATTTTAGACCTGAGCTTCAATTCTTTATCTCATGTGACAAGTATCAACTCCAGCATCATCCATACTTT
GACTCACCTTGACCTGAGCCACAATAACATTTTCCACTTAGACAATGGATTCATACAGGGTCCCAGAAGC
CTTAAGACTCTCAGCCTTAGCTACAACAAACTCCCAACCATCAATCGATCTACCTTCCTGTCAGGACCTG
AAAACCCGATAAAGACTTTGTTCTTGCAGAAAAACCCATTCCAATGTACGTGTGATTCGTTAGATTTTAT
TTTATGGATTGAAGAAAGCAAAATAAAGTTTCCTAGACTGACCACTGACGTGACATGTGACACACGAGTA
AACCAGACGGTTCAACTGCTGATAAACTTTGATATTACTCAGTGTGTAAATGATAGTCTTGCATTCCAGA
TCTACATTCTCACAACTTTCTTCATTATTGTTTTTATGTTTACGGCAACAGTTGCTCACTTGTTTTACTG
GGATGCTTTCTACGCCCTACACTATGTGAAAGCTAAGCTGAAGGGATACAGATCTTTTTACTCAACAGAC
AGTTGTTATGATGTTTTTGTGACATATGACACCACAGATCCACATGTCTCCGAGTGGGTGATGGGGAATC
TGCGGGTGAAACTGGAAGAGGAAGGAGAGAAGCATCATCCTCTGTGCCTGGAGGAGAGGGATTGGCCCCC
AGGAGTCCCACTGCTGGACAACCTCACTCAGAGCATCCAATATAGTCGCAAGACCCTGTTTGTTTTGACA
GAGGGCTATGTTAAGACCGGGGTTTTCAAGCTGGCTATGTATCTGGCCCACCAAAGACTGCTGGATGAAA
GTGTGGACGTGATCGTGCTGCTGATGCTGGAGCCCGTTCTGCAGCATTCTCACTTCCTGCGCCTGAGGAG
GACACTGTGCGAGAAAAGCGTTGTGGAGTGGCCGAGAACGGCGGCCGCAGAACCCTGGTTCTGGCAAAAC
CTGAGGAACGTCGTAAGAGTAGACAATCAGGTCATGTACAGCAAGAGATATTCAAAGTACGGAGTCCCAT
AAGATCTTGATGGTGATGTGCTACATACCATGCATTTGCTGGATAGTCGCTGATACTTTGTTGTACATAA
TGCAGGGTGAAACACAGGAGAGCCTTCCAAGGACTTGCACCGAGCTGTACGCCCATTTTTGTTTCATGAA
AGCTGAGTTAGGTGAACCGAGAGGCAGGGAGCCTGTCAAAATGGAGCAGCTTCAGGGGAGCAATCGGAAA
CTGCTGGGGAATCTGGGACGACTGGCGTTCTACGGGCTCCTCAAACACAAGTACACCTTCAGTGAGCAGG
ACCTGAGGGCTTATGGGATTGATCTACTGTTAACTCAAGGCAGTCTTGGAGCTGGAGTTCTTGTTCGAGA
GGAGTCAGCCATAAACACAACATACCGCTTCACGCATTTGACTCTGCAGGAGTTTCTTGCAGCTACTTTC
TACCATGTTTCCTCCAAGCGGGCCATCTTTGACTTGTTCTCAGAGAGTACCATGTCCTGGCCCAAGATTG
GTTTCCAGAACCACTTCAGAAGTGCGTTTCAGCACTCTCAACAAGCTGAAGATGGTCACTTGGATGTGTT

TGTACGCTTCCTGACAGGCCTGCTGTGCCCAGTGGCAATCAAACCTCTCGCAGGGCTTCTGGCCCTTGGG
AAAGATGATGGCAGCCAGAAGTCATGGGCAGCAGGGTTCCTACAAGGCCTCTTGGTCAGTGGGGGTGCTG
TGGTGTCCCTGCGAGCAGTCAACCTGGCTTATTGTTTACAGGAGCTGCAGCATACGGAGCTGTTACGGAG
TGTGGAGGAGGATTTACGACTTGGTACGCTCGCTGGGAAGCTAACACGGCCTCACTGTGTAGTGCTGGGC
TACCTGCTGCACGTGTCTCCAGAGTGCAGCGAACAGACCAACTTAACATCCTCTCTGAACTACGCAACAG
TGAAATGTTTGCTCCCACAGCTGCTGTACTGTAGCCATCTCAGATTGGAGAATAATCACTTTAAAGATGA
TGTCATGGAATTACTGGGAAGCCTCCTGAGTGCCAAAGACTGCCATATCCGGAAAATAAGTTTGGCAGAG
AATTCTGTCAGCAACAAAGGTGCCAAAGCCCTGAGTCGAGCCCTCCTGGTCAATCGCACGCTAACTTCTC
TCAATCTCCGGAACAACAACATCGGCTCTAAAGGTGCAAAGTTCCTGGCGGAGGCTCTGAAAATGAACCA
AGTTCTGATATCAATCAACTTCCAGAACAACGCCATTGAGGAGGAAGGTGCTCAGGCGCTTGCAGAAGTG
CTGCAGTGCAACCGCAAACTGGTGTCTCTGAATATTCGGAAAAACACAGTCGGAGCAGGTGGAGCCAAGA
GGATTGCAGAAGCACTGAAGACAAACCGGACTCTCACGAAGCTGATTCTTTGTAGTAACCAGCTGGGGGA
CAAAGGAACAGTCGCTCTGGCAGAGGCTTTGACACTAAACCACACTCTGTTGTCGCTCCAACTTCAGAGT
AACTCAATCAGCAACCGGGGAATGACAGCCTTAACCAAAGCACTTAGGCTGAACCGTGGCCTCGTCTCCT
TGAATCTCAGGGAAAACTCCATTGGGGTGGAGGGAGCGAAGAACATGGCACATGCGCTCCATGAGAACCA
CTCTCTACAGGACCTCGATCTTACAGCCAACCTGTTACATGATGAAGGGGTTCAAGCAATAGCTGCAGTT
CCTGGATGGAGATGGATGGAAGAGCCGTTGGCTCGAGTCGAAGCACAAGTCAGACTACGGCCAGTGGAAA
CTGAGTGCTGGAAAGTTCTATGGAGATGCTGAGGCTGATAAAGGTCTCCAGACCAGCCAGGATGCCCGCT
TTTATGCCCTGTCAGCCCGCTTTGATCCCTTCAGCAATGAAGGAAAGCCCGTGGTCATCCAGTTCACTGT
CAAACATGAACAGAAGATTGACTGTGGAGGCGGCTATGTCAAGATCTTCCCCTCTGACCTCGACCAGAGC
GACATGCATGGAGATTCACAATATTACATAATGTTCGGACCTGACATTTGTGGATACAGCACGAAGAAGG
TTCACGTGATTTTCAACTACAAGGGCCAGAACTACCTCATTAAGAAAGACGTCAAATGCAAGGATGATGA
GCTGACCCACCTATACACACTGATCCTGAATCCAGACCAGACATACGAGGTAAAGATCGACAATGAGAAG
GTGGAATCTGGCTCCTTGGAGGACGACTGGGACATGCTACCTCCGAAGAAGATTAAGGACCCTGAGGCCA
AGAAGCCGACTGACTGGGATGACAGGGCCAAGATCGATGATCCCAGCGACTCCAAGCCTGAAGACTGGGA
CAAGCCAGAGACCATCCCAGACCCTGATGCCAAGAAACCTGATGACTGGGATGAGGACATGGATGGAGAA
TGGGAACCTCCCATGATCACCAACCCAGAGTATAAGGGTGAGTGGAAACCCAAGAAGATTGACAACCCTG
ACTACAAGGGAGCATGGGTCCACCCTGAGATAGACAATCCAGAGTACACTCATGAGCCCACCATGTACAA
GTTTGACAACATTGGAGTACTGGGTCTGGATCTGTGGCAGGTTAAATCTGGAACCATCTTTGACAACTTC
CTGATCACTGATGATGTCAAAGAAGCAGAGGAGTTTGGGAAGGAAACCTGGGGAGCTACTAAGGGACCAG
AGAAGAAGATGAAGGAGGAGCAGGAGGACATGGAGAGGAAACTGAGGGAGGAGGAGGAGAAGAGCAAGAA
AAAGGACACTGAAGGTGATGAGGAAGAGGAAGAGGAGGACGATGAGGAAGATGACGAGGAGGAGGGTGAG
GAAGCAGATGGTAAGCATGATGAGGAGATGGAGGAGGACGCTGGGACAGAAGAGGAGGATATCAAAGAGA
AGGATGAGCCCTCCAAACAGAAGGTGCTGGTGATGGAGTATTGTTCAGGAGGAAGTTTGCTCAGCCTGCT
GGAGGAGCCAGAAAACGCCTTTGGTCTGCCTGAAACAGAGTTCCTCACTGTATTACAGTGTGTAGTGCAG
GGTATGAACCACCTGCGGGAAAATGGTGTGGTACACCGGGACATTAAGCCAGGAAACATCATGCGGCAGG
TCGGGGAGGACGGCAAGTCTGTCTACAAGCTGACAGACTTTGGAGCAGCAAGAGAGTTAGACGATGATGA
AAAGTTTGTATCCATTTATGGAACTGAAGAGTATCTGCATCCAGACATGTATGAGCGTGCTGTGCTGCGT
AAGCCTCATCAGAAATCCTATGGAGTGAGTGTTGACCTGTGGAGTATTGGTGTAACCTTATACCATGCTG
CCACAGGGAGTCTTCCCTTCACACCGTATGAAGGACCCCGCAGGAACAAACCCACCATGTACAAAATAAC
CACAGGGAAACCTATGGGAGCAATAGCTGGCGTCCAGCGGGTGGAGGGTGGACCTATAGAGTGGAGCTAC
CACCTACCTCACAGCTGTCAGCTGTCACAGGGTCTGAGGGTGCAGCTGGTTCCAGTGCTAGCAGGGATAC
TG

>Gasterosteus aculeatus tlr3-tlr8-nlrc3-calr-ikbke
CCTTCTCTGCGCTACAGAGCCTCAGAGTCCTTGATGTGTCCAAAAACAAACTGCAGTCAGCCAGCCTCGG
CTCTCAGCCTCAGCTGCCCGGCCTTGTGAGCCTCAACCTGGCATTCAGTTACTTCACTACACTGAAGAAA
GACGACTTCTCGTATCTTAGTCATTCGTCCTTCCTGCGGGTCCTCAACATATCATCCGTGCCGCTGAAAA
CGCAGTTGGAGCCTGGCTGCTTTAAGCCCATTTCTGGCCTACGTACTTTGATCATGGATGGAAGTCAAAT
GGGCACGAATGTTATTTCTAAACTCTGTTCGGAGTTGGCGGTGACGGCCATCGATGCCTTGTCTCTTCAG
AAGACGAAGCTGATCACACTCCCGAAGGCAGCCTTTGCAGGGCTACAGAAGACAAACATAACCTTTCTGG
ATCTGTCCCGTAACACAATGGGCAAAATTGAAGGCGGTGCATTCAGCTCGCTGTCTAGACTTCAGACTCT
AATCTTGTCGGACAACAACATCACACGCCTGAGCAATGACACATTTCAGGGGCTCAATAGTTTGAAAACA
CTCCAATTGACAAAAGCCCTGGTGAAAAGTCACCGCATCCCGATTATCGAGGATTTCTCCTTCCAACCAC
TGGGTGCCCTTGAGAGTTTGATACTGCAGAGAACTGTCGTTCATGAAATTACCGAGCACACATTTTCAGG
CTTGACAAGTCTTCGGGAACTGGATTTGAGCTTCAGTAATTATACATCGCTCAAAATCATCACCAACAAG
ACGTTCGTCTCACTCGCGGGATCGCCTCTCACAAAGCTGAATCTGACATGGACGGCCATCGCAAAGATTG
ATCCTGGAAGCTTCTCCGTTTTGAGAAACCTCACCACCCTCGTTCTGGATTTCAACTTTATCAAGCAAGT
TCTCACCGGCAGAGAGTTTGAAGGCCTGGACCAGGTCGAAGAGATTCACATGTCAGTTAACCACCAGACG
GTCGGTCTGAGCTCCACGTCTTTCGTGAACGTGCCCAACCTGAGGATCCTGACTTTGGGGAGAAGTCTCA
CAGCCGAAGCGGTGAACGTGGATCCCTCTCCATTCAGTCCGCTGGCCAAACTCACGGTCCTGGATCTCAG

CAACAACAACATCGCAAACATCCGTGTGAATATGCTGGAAGGGCTCGTGAACCTCAAAGTGCTGATGCTC
CAACATAATAACTTAGCCCGTGTGTGGAAGAGCGGAAACCTAGGCGGTCCGGTGCTGTTTCTCAAAAGCG
CAGAGAGGTTGACGACCTTACATATGGACAGTAACGGGCTGGATGAGATCCCGGCAGAGGCTCTCAGTGG
GTTGGGCAACCTGCGTGAAATCAGCCTGGCCAACAACCTCCTTAACAATCTGCAGAACTCCATTTTTGAC
CATCTGAGCTCACTGCGGGTTTTAAATCTACAAAAGAACCTGCTCACAGCTGTGAGGCCACAGGTGTTCC
AAGCTCCTTTGCGCAACCTTAGCCTGCTTCTCATGGACAGAAATCCATTTGACTGCACGTGTGAGAGCAT
GGTGTGGTTCGTGAAATGGTTGAACAGTACAAATGGGACAAGAGTGCCCGGTCTCAGGGACCAGTATCTG
TGCAACACCCCGCTGGCTTACTTTAACCGCTCTGTCATGGTCTTCGACACCCTCTCCTGTAAAGATATGA
CCCCATTTCAGGCTCTTTACATACTGAGCAGCACTGTCGTCATCACCCTGATGGTATCTGCGCTTCTGGT
GCGGTTCCAAGGCTGGAGGATCCAGTTCTACTGGAACATATTGATCAACCGCGCATTAGGATTCAGCGAC
GCCGAGGTTGAAGAGGGCAGGGAGTTTGAGTACGATGCTTACGTCATCCACGCGGAGAAAGACGCCAGCT
GGGTGGAGAGGAGGATAGCCCCCCTAGAGAATGACATGTGCAGATTTTGTTTGGAGGATCGAGATTCGGT
CCCCGGCATGTCAAAGCTCGAATCCATCGTGGACAACATGAGAAAGTCCAGAAAGATCTTGTTCGTCGTC
ACTGAGACTCTTCTCAGAGATCCCTGGTGTAGGAGATTTAAAGCCCATCATGCACTTCACCAGGTCATTG
AAGCCAGCAGGGACTCTGTAGTTCTGGTCTTCCTGCAGGACGTCCACGACTTCAAGTTGTCTCGCTCCCT
CTTCCTCCGTCGGGGCATGCTGCGTTCATGCTGCGTCCTGGACTGGCCCGTCCATAAGGAGAGGGTGCCG
GCCTTTCACCAGAAGCTCCTCATAGCACTGGGCATGACAAATCGATTGCAAGAGCTTATCAAACAAGAGT
GCTTTAACTCTGGACGAGTGCTAGTCCTCAGCTCAAATAATCTGTTCTTCATTTCTCGCGAGCAATTTCA
GGGCTATGGGGATATCGCATGCCTCAACCTCTCCAGTAACGGATTCTCAGCCGCACTCAATGGAACAGAG
TTCTCCTTGCTGCCAAATCTGACATACCTAGACCTTTCATTCAATAAGATTGATCTGGCCTATAACAACG
CCTTCAAAGAACTAAAGCAACTACAAGTGCTGGACCTCAGTAACAATGACCACTACTTTAAGAAATTTGG
GATAACGCTTAATTTAAATTTCATTAAAAATCTGCCGGTTCTCAGGGTGCTGAATATGAGTCACAATTCC
ATTTCCAGGTTGACAACAAAAAGGATGTACAGCAAATCACTAACAGAACTTCAGTTTTCACATAATTTAC
TCGGGACTCTTTGGAAAGAAGAATTTGGCAGCTACAAAATGCTTTTTACTAATCTTACTAATTTGACAAT
TTTAGATATATCACACAACTGCATAAAAAAGATTCCAGAAAATGTTTATGAATATTTGCCACCTAACCTC
ACCATGTTACACGTACATCACAATTTTCTGACTGATTTTAGATGGGACAAACTGATGCGTTTCCATCAGC
TACAAATTTTAGACCTGAGCTTCAATAACATATATAATGTGACAGGTATAAACCTAAACATCTCTTTGAC
TTACCTTGACCTGAGTCATAATCACATTTTCCACTTGGAGAACGGGTTTCTGAAGGGCGCCAGGAGCCTC
TCGACTCTGATCCTTAGCAACAACAAACTAACTACAATCAATGAATCCACCTTCCAATCCAGACCTGAAT
CACAGATTCAGACCTTGTTCTTGCAGGGAAACCCATTCCAGTGTACCTGTGATTCATTAGATTTCATTCT
GTGGATTGAAAACAGTGATGTAAAGATCCCCAGACTGACCTCCAAGGTAACGTGCGACACACCAGCCAAC
ATGAAGGGTAATGCTCTGATCTACTTTGGCATTGATCAGTGTGTAAATGACAGTGAGGCGTTCATGATCT
ACATCCTCACAAATTCCTTTATTATTGTTTTCATGTTTGTGGCAACACTTGCTCACTTATTTTACTGGGA
TGCTTCCTACGTGCTTCACTATATGAAAGCCAAGCTGAAAGGATACAAATCCTTGAACTCACCAGACAGT
GTCTACGGTGTCTTCGTGATGTACGATACCAGAGATCCACATGTCTCTGAGTGGGTGATGACGAATCTGC
GAGTAAAACTGGAGGAGGAAGGAGATATGTATCTTCCTCTGTGTCTGGAGGAGAGGGATTGGGCTCTTGG
AGCCCCACTGGTGGAAAACCTCTCCCAGAGCATCCGGTACAGTAGCAAGACCCTGTTCGTCTTAACCGAG
GCCTATGTTAAGACCGGGGTGTTCAAGCTGGCGATGTGTCTGGCCCACCAACGACTGCTTGATGAAAACA
AGGACGTCATCGTCCTGCTGATGCTGGAACCCGTCCTGCAGAACTCTCACTTCCTGCGCCTGAGGAAGAG
GATGTGCAGGAAAAGTGTCTTGGAGTGGCCGAAAACACCTGCCGCAGAGCCCTGGTTTTGGACCACTTTG
AGGAACGTGGTCAAAGTGGACAACCAGGCGATATGCAACAAGACTTATAAAAAGTACGGAGTCCCATAAA
ATCCTTATGGTGATGTGCTACATACCCAGCGTCTGCTGGATCGTGGCCGAGACTCTAGTGTACATCATGC
GGAGCGAAACGGACGAGAGCCTTCCGAGGACTTGCACAGAGCTGTACGCTCACTTTTGCTCCATGAAGGC
GGAAGCCGGCGAACCGAGAGGCAGGGAGCCTGTAAAGATGGAGCAGCTCCACGGGAGCAACCGTAAACTG
CTGGGGAACCTCGGACGATTAGCCTTCTATGGGCTGCTCAAACATAAGTACACCTTCAGCGAGCAGGACC
TCAGGGCCTACGGAATAGATCTCCTGTTAGGTCAGGGCAGTCTTGGTACTGGAGTTCTTGTCCGGGAGGA
GTTGGCCATATGCACAACATACCGGTTCACTCATTTGACTCTGCAGGAGTTTCTTGCGGCTACTTTTTAC
CATATCTCCTCCAAGCGGGCCATCTTCGACCTGTTCTCAGAGAGCACCATGTCCTGGCCCAAGATTGGTT
TCCAGAACCACTTCAGAAGCGCCTTTCAGCACTCGCAACAAGCTGAAGACGGTCACTTGGATGTGTTTGT
GCGCTTCCTGACGGGCCTGCTGAGCCCGGCGGCGCTGAAGCCTCTGGCTGGGCTCCTGGCCGTGGGAAAA
GACGAGGGAAACCAGAAGGCGTTCGCCGCGGGGTTCCTACAGGGCCTCTTGGTCGGCGGAGGGGCCGTGG
TGTCCCTACGCGCAGTCAATCTGGCTTATTGTTTACACGAGCTGCAGCACGCGGAGCTGTTGCGCAGTGT
CGAGGAAGACTTGCAGCTCGGCAGCCTTGCGGGGAAGCTAACGAGGGCCCACTGCGTGGTGCTGGGGTAC
CTTCTGCACGTGTCCCCAGAGTGCAGCGAGCAGACCAACCTGACAGGCTCTCTGAACTACGCGACGGTGA
AATGTCTGCTCCCGCAGCTGCTGTACTGCAGCTATCTCAGGTTGGAGAATAATCACTTCAAAGATGATGT
CATGGAGTTGCTGGGAAGCCTGCTGAGTGCCAAAGACTGCCATATCCAGAAAATGAGTTTGGCGGAGAAC
GCCATCGGTAACAAAGGCGCCAAAGCCCTGAGTCGCGCCCTCTTGGTGAACCGGACGCTAACGTCTCTCA
ATTTGCGGAACAACAACATTGGCTCCAAAGGTACAAGGTTCCTGGCAGAAGCTCTGAAAATGAACCAAGT
CCTGGTATCAATTAACTTCCAGAACAATGCAATCGAGGAGGAAGGTGCTCAGGCCCTTGCAGAAGTACTG
CACTGCAACCGCAAACTAGTCTCTCTCAACATACGGAAGAATACAATTGGAGCTGGCGGAGCCAAAAGGA
TCGCAGAGGCGCTGAAGACGAACCGGACGCTCACAAAGCTGATTCTTTGCAGTAACCAGCTTGGGGACAG

AGGGGCGATCGCTCTGGCAGAGGCGTTGGCAGTCAACCACACTCTGCTCTCACTCCAACTTCAGAGTAAC
TCAATCAGCAACAAGGGGATGACCGCATTGACCAAAGCTCTCCGGCTCAACCGGGGCCTTGTCTCCTTGA
ACTTGAGGGAGAACTCCATCGGGGTGGAGGGAGCCAAGAACATGGCCCATGCTCTCCACGAGAACAACTG
TCTGCAGGAGCTCGATCTCACAGCCAACCTGTTGCACGATGAAGGGGTGCAAGCTATAGCCGGAGTTTCA
AGACGGCGATGAGTGGAGAAGTCGTTGGCTGAATTCCAAACACAAGTCTGACTACGGAGAGTGGAAACTG
ACGGCGGGCAACTTCTATGGAGATGCTGAGAAAGACAAAGGTCTGCAGACAAGCCAGGATGCTCGTTTCT
ATGCTACATCCGCCCGTTTTGAGCCGTTTAGCAACGAGGGGAAGCCTTTGGTCATTCAGTTTACAGTAAA
GCATGAGCAAAAGATTGACTGTGGTGGTGGCTACGTGAAGGTCTTCCCTGCTGACTTGGATCAGGCTGAC
TTGAATGGAGAATCTTCCTACTACATCATGTTTGGCCCTGATATCTGTGGCTACAGCACAAAGAAAGTCC
ACGTCATCTTCAATTACAAGGGCAAGAATCACCTCATCAAGAAAGAGATTAAGTGCAAGGATGATGAGCT
GACCCACATGTACACGCTGATCCTGAATCCAGATCAGACCTACGAGGTGAAGATTGATAACGAGAAGGTT
GAATCTGGCAGTCTGGAGGACGACTGGGACTTCCTTCCTCCCAAGACCATTAAAGACCCCGAAGCCAGTA
AGCCAGAGGACTGGGATGATCGTGCCAAGATTGACGATGCTGATGACACTAAGCCAGAGGACTGGGACAA
ACCTGAAAACTTCCCAGACCCCGATGCTAAAAAACCAAGTGACTGGGACGAGGATATGGACGGAGAGTGG
GAGCCACCAATGATCCCTAACCCAGAGTACAAGGGAGAATGGAAACCCAAACAGATCGATAACCCCAACT
TTAAGGGAACATGGGTGCATCCTGAGATTGACAATCCTGAATACAGTCCTGATTCCAACATCTACAAGTT
TGACAAGATTTCTGTTGTAGGTCTTGATCTTTGGCAGGTGAAATCCGGTACCATCTTTGACAACTTCCTC
ATCACGGACGATGTGAAGGAAGCAGAAGACATTACAATGGAGACATGGGGTGTGACAAAGGAACCTGAGA
GGAAAATGAAACAGGAGCAGGATGACTTGAAACGGAAGGAAGAGGAGGAGAAGAACAAGGAACAAGACGC
AGAGGCTGTTGATGAAGAAGTCGATGGGGAAGACTCAATGGATGACATGGAGGAGGCACTTTCCGAAATG
GATGCAGAGGAAGCAAAGCTTAATAAAGATGAGCCCTCCAAACAGAACGTGCTGGTGATGGAGTATTGTT
CAGGAGGAAGTCTACTCAGCCTGCTCGAAGAGTCGGAGAATGCCTTTGGCTTACCTGAAACCGAATTCCT
CATAGTTTTGCAGTGTGTTGTGCAGGGGATGAATCACCTGCGAGAAAATGGCGTGGTACACCGGGACATT
AAGCCAGGCAACATCATGCGGCAGGTTGGGGAGGATGGAAAGTCTGTTTATAAGCTGACTGACTTTGGAG
CAGCGAGAGTGCTGGAAGATGATGAGAAGTTTATGTCTATCTATGGAACGGAAGAGTATCTGCACCCGGA
CATGTATGAACGTGCTGTGCTGCGTAAGCCTCATCAGAAATCGTACGGAGTGAGTGTAGACCTGTGGAGT
ATCGGTGTGACATTTTACCATGCTGCCACTGGGAGTCTTCCCTTTGCCCCTTTCGGAGGACCCCGCAAGA
ACAAGCCCACCATGTACAAAATAACTACAGAGAAGCCTATGGGGGCAATAGCTGGAATACAGCGGGTGGA
GGGCGGGCAAATCGAGTGGAGCTACCACCTACCTCACAGCTGCCAGCTCTCACAGGGTCTGGCGATGCAC
CTTGTTCCGATACTAGCGGGCATCCTG

>Oryzias latipes tlr3-tlr8-nlrc3-calr-ikbke
CCTTCTCTGGTCTGCAGAACCTACAACATCTTGATGTGTCGAAGAACAAGCTGCAGTCGGCAAAGCTGGG
CTCGCGACCTCAGCTGCCCAGCTTGCGGAGTCTCTGCCTGGGACAGAATGTTTTCACCTCTTTGAAGAGT
GAGGACTTTTCTTACCTCAGTTCATCCCCGTCTCTGCAAGCTCTTAACTTGTCATACGTGTCTCTGAAAA
CAGTTGAGAGTAGATGCTTTCAGCCAATTTCAGGTCTGCGTACTTTAATCTTGGATGGGAGCGATATGAG
CTCTCAGGCAATGTCCAAACTGCTGTCAGCGCTGTCAGCGACTGCCATAGACAGTCTGTCTCTGCAGAGG
ATGAAGCTGGTTGCACTCAGAAACCTGACATTTGCCGAACTGCAGAAAACAAATCTAACCTATTTGGATC
TGTCTGCGAATGGAATGACCAATATCGAAGACGGCTCATTTCAGTGGCTTCCTAAACTCCAATCCCTAAT
TTTGTCAGATAACAACTTAAGGCGCTTGACCAGAGGAACATTTCAGGGACTTAAAATGTTGAAGAAACTG
CAGCTCGGGAAAAAACATATATCTTCTGCTGTCATTGAAGATTACGCTTTCCAACCCTTAAACAACCTGA
ATAGCTTAATGTTGGATCATACTGCCTTTTCAAAAATCACAGAAAAAACTTTCACCGGCCTGACAAGTCT
CACAGAGTTAGATTTGAGTTGGAGCAGCTACACGCAGACCCAACAGAGAAGACTCACCAACAAGACCTTT
GTCTCACTTAAAGATTCGCCTCTCAGGAAGCTGAATTTGACGGGAAACGCTCTGACTGAGCTCCAACCGG
GGAGCTTCTCTGCTCTGAGCAGTCTTGTTGTTCTCATCCTTGATTTCAATTTTATCAAGCAGACTCTGAC
AGGCAGGGAGTTTGAAGGTCTTTCTCAGATACAGGAAATTCACCTGAGCAACAACATCCAATCCATTAAC
CTGACCTCCTCGTCATTTGTGAATGTATCTAGTCTCAGAGTCCTGACTTTGGGTAAGAGCCTTAAAGCAG
AAGCCCTAAATAAGGATCCTTCTCCATTATGGCCCCTGACTAACCTCACTGTCCTGGATCTCAGCAACAA
CAACATTGCTAACATCAGGACGAACTTACTGAAGGGGCTTACGGACCTGAAGGTGCTGAAACTCCAACAC
AACAACCTAGCCCGGTTGTGGAAGATGGCTAACCTAGGTGGCCCTGTGTTGTTTTTGAAGGACACACACA
AGTTAGAAACCCTGCAAATGGATTACAATGGATTGGATGAGATTCCAGTGGGGGCTCTTCAAGGTTTGAC
GTCACTTAGTGTGCTAACCCTCAGTAACAACCTCCTAAACAATCTCAAGAACCCAGTTTTTGATGATCTG
AAGTCTCTTCGTGTGTTGACTTTAGCAAAGAACCTGATCACAAATGTTAGGCCGGAAGTGTTCCAGACTC
CCATGAGCAACCTCAGCGTGCTTTGGATGGACAAAAACCCCTTTGACTGCACCTGTGAGGGTATCCTTTG
GTTTGTGACCAGGTTGAACAACACTAATGGAACCAGTGTGCCCGGTCTGAAAGACAACTACAAATGCAAC
ACTCCTTTAATGTATTTTAACCGCTCCATCATGGATTTTGACCCCATGTCGTGCAAAGATAAAACCCCAT
TTCAGGCTCTATACATGCTGAGCAGCACAGCAGTTTTAATCATGATGGTCAGCGCACTCCTGGTTCGCTT
TCAAGGCTGGAGGATTCAGTTTTACTGGAACGTTTTGATCAATCGTACTTTAGGATTTAGTGACGCCACA
GCGGAAGAGGCCAATGGCTTTGAGTATGATGCCTACATTATACATGCTGAGGAAGATGCTGGCTGGGTGA
CAAAACAAATGACCCCTTTGGAGTCTGCAAAATACAGGTTTTGTTGGGAGGAGCGAGATTCAATCCCAGG
AATGTTCTATTTGGAATCTATTATGGAAAATATGAGAAAGTCCAGAAAAATTCTGTTTGTGGTGACAGAA

AGTCTTCTCAAAGATCCTTGGTGTAGAAGATTCAAGGTCCATCATGCCCTTCACCAGGTCATCGAAGCAA
GCAGAGACTCAGTTGTTTTGGTCTTCTTGCAAGACGTCAACGACTACAAACTGTCCCGCTCACTATTCCT
CCGCAGGGGCATGTTGCGTCCCCGTTGCATTCTTGAGTGGCCTGTTCAGAGGGAGAGAGTTCCTGCCTTT
CATCAGAAGCTGCTTATTGCACTTGGCATGACAAATCGACTGCAAAAGAAAAGAAAGCAGTGTTTTGACT
CCGGAAAAGTTCTTGTCCTCAGCTCTAATAACCTGTTCTTTATTTCTCCAAAGCAGTTTGAAGGATATGG
AGATATCGCATGTCTCAACCTCTCAGGAAATGGATTTTCAGCAGCTCTTAATGGAACAGAGTTCTCCTCA
CTGCCTAATCTGACATATTTAGACCTGTCCTACAATAAGATTGATCTGGCATATGACAACTCCTTCAAAG
AGTTAAAAAAACTAAAAGTGCTCGACCTCAGTTACAATCCACACTATTTTAAAGCATTCGGACTTACGCA
TAATCTGAATTTCTTAAAGAATCTTCCTGACCTACAGGTGCTGAACATGAGTCATAACTCCATTCACACT
TTAACAACTAAGCAGCTGCACAGCGAATCGTTGACAGAACTTCAGTTTGCATATAATTCCCTTGGCGTTC
TCTGGAAAGAAAAAGACGGTTCGTACACAAAACTTTTCACTAATCTTCACAATTTAACTGTTTTAGAAAT
ATCGCACAACAGAATTAGTAAGATCCCCGACAATGTCTATGAATCTCTGCCTCGTAACCTCACCAAACTC
AGCATTCATCATAACTTGCTGACAGATTTTTCTTGGGACAAACTGAAGTTTTTCCATCAACTTCAGATCT
TAGATCTGAGCTTCAATTCTTTGTCCTACATTGCAGGAATCAATTCAAGCATCACGCAGTCTTTGACTTT
ACTAGATCTGAGTCACAACCACATTTTCCACCTGAAAGATGGATTGTTCAATGGTCCAAAAAGTCTCCAG
ACTCTGAGTCTGAGCTTTAACAAACTGACTTTGGTTAACAGCTCGAGCTTCGGAGATGAGAACAACATCA
GGACTTTGTTCTTGCACAAGAATCCGCTCCAATGTATTTGTGATTCGTTAGATTTCATTTTATGGATCGA
AAGCAGTCCCATCAAGATCCCGAGGTTGACAACTGCAGTGACCTGTGACACGCCAGAAAACCAAAAGGGA
AAACCACTAGTTTACTTTGACATTAGGCAGTGTGTAAATGACGATCTAGCACTGAAGCTGTACATCGTCA
CAACATCTATAATTGTTGTTTTTATGCTTTTGGTAACAGCATTTCCTTTATTCTACTGGGACTTTTCGTA
CATCACACACTATTTGAAAGCTAAACTGATGGGATACAAGTCCCTGAACTTCCCAGACAATGCTTATGAT
GTCTTTGTGACATATGACACTAAAGACTCACACGTATCTGAATGGGTGATGAGCAACCTGCGGGTTAAAC
TGGAAGAAGACGGAGAAAAGCATCTTCCTATGTGTCTGGAGGAGAGAGATTGGCCCCCAGGAGTCCCGCT
GGTAGACAACCTCATGCAGAGCATCCGATACAGCCGCAAGACCCTGTTTGTTTTAACAGAGGGTTATGTT
AAGACAGGAATCTTCAAGATGGCAATGTATCTAGCACACCAGAGACTGCTGGATGAAAATGTCGATGTGA
TTGTGCTGCTCATGCTAGAGCCCGTCCTGCAGCACTCTCATTTCCTGCGCCTGAGGAAGAGGCTCTGTGG
GAAAAGTGTTGTGGAATGGCCACGGACAGCAGCTGCAGAGCCCTGGTTCTGGCAAAACCTCAAAACTGTC
ATTAGAAAAGATAATCAAGGGATGTACAACAAAACATATTCCAAATACACAGGCTCACAGTATCTTGATG
GTGATGTGCTACATACCATGCATTTGCTGGATAACAGCTGATACTTTGTTGTACATCTTGCAGAGTGGAG
CACAGGAGAGCCTTCCCAGGACTTGCACTGGCTTATATGCCCATTTTTGCTACATGAAAGCAGAATTAGG
AGAGCCAAGAGGCAGGGAACCCATTAAAACAGAGCAATTTCATGGGAGTAACCGAAAACTACTGGGGAAC
CTTGGGCGGCTGGCATTTTATGGACTCTTAAAGCACAAATACACCTTCAGCGAGCAAGACCTCAAGGCTT
ATGGGATAGATTTACAGTTAACTCAAAGCAGTCTTGGTGCTGGAGTCCTCATTCGATTGGAGTCCACCAT
CCACACAACATATCGATTCACACATTTGACTCTGCAGGAGTTTCTTGCAGCTACATTCTACCACGTTTCC
TCCAAGAGAGCCATTTTCGATCTGTTCTCAGAAAGCACCATGTCCTGGCCAAAAATTGGCTTTCAGACCC
ATTTCCGAAGTGCCTTTCAGCACTCACAACAGGCTGAAGATGGTCACTTGGATGTTTTTGTGCGATTCTT
GACAGGCCTGCTCTGCCCAACAGCACTGAAGCCTGTCGCAGGGCTTTTAGCGGCAGGAAAAGATGATGGT
AACCAGAAGGTGTGGGCAGCTGGGTTTTTGCAAGGCCTCCTGTCCAGCGGGGAAGCTGTGGTTTCACTGC
GCATTGTCAACTTGGCTTATTGCTTACATGAGCTACAACACACCGAGCTGCTGCGGAGTGTCGAGGACGA
TTTACGGCAAGAAAGCTTGGCTGGAAAGTTAACTCGACCTCACTGTGTTGTCCTGGGCTTCCTGCTGCAT
GTGTCTCCAGAGTGCAGCGACCAGACCAACCTAACTGGCTCTCTAAACTACTCAACAGTGAAAAGCTTGC
TCCCACAACTGCTTTACTGCAGCCACCTCAGGTTAGAGAATAATAACTTCAAAGATGATGTCATGGAACT
GCTGGGAAGCCTCCTTAGTGCAAAGGATTGTCATATCCAAAAATTAAGTTTGGCAGACAATGCTATCAGC
AACAAGGGAGCCAAAGCCCTGAGTCGAGCTCTTCTGGTGAATCGTACATTAACATATCTCAATCTCCGGA
ACAACAACATTGGGTCTAAAGGTGCAAAGTTCTTGGCAGAGGCTCTGAAAATGAATCAAGTCCTGACATC
AAAACTCAGCTTCCAGAACAACAGTATCGAGGAGGAGGGCGCTCAGGCCCTGGCAGAGGTTCTGCAGTGC
AATCGCAAGCTGGTGTCTCTGAATGTGCGGAAAAATAAAATTGGGGCAGATGGAGCCAAAAGGATTGCAG
ACGCACTGAAGATGAACCAAACTCTAACAAAGCTAATACTTTGCAGTAACCAGCTGGGGGACAAAGGAAC
CGTCGCTCTGGCCGAAGCGTTAAAACTCAACCAAACTCTTCTCTCACTCCAACTTCAAAGCAACTCAATC
AGCAACAGAGGAATGACTGCACTGACCAAAGCCCTCAGGTTCAACCACGGTCTAGTAACCTTGAATCTCA
GAGAGAACTCAATTGGGATTGAAGGAGCAAAGAACATGGCCCAGGCGCTCAAGGAGAACAACTCTCTGCA
AAATCTGGATCTCACGGCCAATTTATTACATGATGAAGGTGTTCAGGCGATATCTGGAGTTTCTAGACGG
CGATGAATGGAGAAGTCGCTGGGTGAACTCCAAACACAAGTCTGACTACGGAGAGTGGAAGCTGACGGCG
GGGAACTTCTACGGAGACGCAGAGAAGGACAAAGGTCTGCAGACCAGCCAAGACGCTCGCTTCTACGCCG
CCTCGGCCCGCTTCGAGCCGTTCAGCAACGAGGGGAAGTCTCTGGTCATTCAGTTCACGGTCAAGCACGA
GCAGAAGATCGACTGCGGTGGGGGCTACGTGAAGGTCTTCCCGTCCGACCTGGACCAGACCGACATGCAC
GGAGACTCCTCCTACTACGTCATGTTTGGCCCCGACATCTGCGGCTACACCACCAAGAAGGTCCACGTTA
TCTTCAATTACAAGGGCAAGAATCATCTCATCAAGAAGGAGATCAAATGCAAGGATGATGAGTTCACCCA
CCTGTACACACTGATCCTGAACCCAGACCAGACCTACCAGGTCAAGATTGACAATGAGAAGGTGGAATCT
GGAAGTCTGGAGGAGGACTGGGACTTCCTGCCCCCCAAGAAAATCAAGGACCCTGAAGCCAAGAAGCCAG
AAGATTGGGACGACCGTCCGAAGATCGATGATCCCGAAGACACCAAGCCTGAGGACTGGGAGAAACCAGA

ATACATCCCAGATCCTGACGCCAAAAAGCCTGAAGACTGGCAGGAGGACATGGATGGAGAATGGGAGCCG
CCGATGATCAGCAACCCGGAGTACAAAGGAGAATGGAAGCCCAAACAGATCGATAATCCAGACTACAAAG
GAGCCTGGGTGCATCCAGAAATCGATAACCCCGAATACAGCCCAGACGCAAACATCTACAAGTTCGACAG
CATCGGGGTTCTGGGTCTGGACCTCTGGCAGGTCAAGTCAGGGACCGTCTTCGACAACTTCCTCATCACC
GACGACGTGAAGGAAGCGGAAGACGTGGGCAAAGAGACGTGGGGCGCGACGAAGGAGCCGGAACAGAAAA
TGAAGCAAGAGCAGGACGAGTTGAAGCGAAAAGACGAAGAGGAAAAGAACAAGGACCAAGACACAGACGG
GGATGATGAAGATATTGATGAAGACGAGGAAGAATCAAAGGAAGAAGCAGAGGAGGCACTTTCAGAAACA
GATGAAGATGCTCAGGCTAAAGATGAGCCCTCTAGGCATAAAGTTCTAGTAATGGAGTTCTGCTCCGGGG
GAAGTCTGCTAAACCTCCTGGAGGAGCCAGAAAACGCCTTTGGGCTCCCCGAAACCGAGTTCCTCACTGT
CCTCCAGTGCATAGTTCAGGGGATGAATCACCTGAGAGAAAACGGGGTGGTGCATCGGGACATAAAGCCC
GGGAATATACTGCGGCAGGTCGGAGAAGACGGGAAGTCCATTTACAAGCTGACGGACTTTGGAGCAGCAA
GAGAACTGGAGGACGATGAGAAGTTTGTGTCTATTTATGGAACTGAAGAATACCTGCACCCAGACATGTA
TGAGCGAGCTGTGCTGCGAAAGCATCACCAGAAAACCTACGGCGTGAGCGTGGACCTGTGGAGCATCGGT
GTGACTTTGTACCACTCCGCCACCGGGAGTCTCCCCTTCACCCCGTTTGAAGGGCCCAGAAAGAACAAGC
CCGTCATGCACAAGATCACCAGAGAGAAGCCAATACATGCAATAGCGGGTGTACAGCGGGTGGAAGGCGG
GCCGATAGAGTGGAGCTACCATTTACCTCACAGCTGCCAGCTCTCCAGGGGTCTGAAGGTGCTGCTGGTT
CCAGTACTTGCAGGTATTATG

>Esox lucius tlr3-tlr8-nlrc3-calr-ikbke
CCTTCGCTGCTCTACAGAGTGTGACACTGCTGGATTTGTCTAATAATGACCTGAAGACAGCCAAGTTGGG
CATCCAACCTCAACTTTCAAGTCTGGAGACTCTCATCTTGTCGAGCAATGCCATATCAACCATTGAACTG
AACGACTTCAATTTTTTAACAAATTCATCTTCCCTACGAGTACTCAACCTGTCTTATCAAAAAACCCATA
TAACGTTTAAGTCTGGTTGCCTGAAGCCCATTGCTGGCATACATGAGCTAGTCATGGATGGGAACAAACT
TAGCACTGCGCTCACATCTAAACTCTGCACAGAGCTCTCTGGGACAGCAATCCGCAATATTTCCCTCCGG
TACACAGGATTAGTCACGCTTGCCAAAACAACCTTCAAAGGCCTGGTGAAGACCCAACTAATTTCACTGG
ATCTTTCCCACAATGGCATGACTAAGATCGACAATGGCTCTTTTCAGTGGCTGCCTGTTTTGGAAGTTCT
TTCTCTAGAACAGAACAACCTCAAGCGCCTGACAAGTAACACTTTCAAGGGACTAGGGAATCTGACACGC
CTGAACCTGAAAAACGCGCTATTGAAGACGTCCTCTAACCCAATTATTGACGACTTTTCCTTCCAGCCAT
TACGAGCGCTGGAGATCCTGATCATGCAGTATACAGCCTTTCGAGACATCTCAACCCACACCTTTGCAGG
TTTGATAAGTCTCCGCCAACTCCACCTGGGCTCAGCCAAATGCATCGCCCTAAAACGCATCACCAACCAG
ACCTTTGTGTCACTGGCAGCTTCCCCGCTTCAGACACTAAACCTCTCCGACACAAAGATCTCCAGTCTAG
ACCCAGGTGCCTTCTACAGCTTGGGCAACCTCACAATTCTTCGACTGGGCTTCAACTCCATTTCTCAGAC
CCTGACGGGGAAAGAATTCCAGGGTTTGGGCAAGCTGCAAGAAATCTACCTTTCCAACAACAACCAAAAA
ATCACCCTCAGCCCCACATCCTTTGCTTACGTGCCCACTCTCATAACTCTGCTGATGGGGAAAGTTCTGA
TAGGCAGCCTGGATTTGGAGCCCTCTCCATTCAAGCCTCTGTCCAACCTCAGCATCCTGGACCTCAGCAA
CAACAACATTGCCAAAATCAGCAGAGGCATTTTCGATGGACTGGAGAATCTAAAAGTGCTGAAGCTACAG
CACAACAATTTGGCTCAGGCTTGGACAAGTTTCAACCCAAGGGGGCCGGTAATGTTTCTTAAGGGGCTCT
GCAACCTAGTCGCCCTGGAGATGGACTCCAATGGTCTGGATGAGATCCCCTTGGAAGCCCTTCGTGGACT
GCCAAACCTTCAAGAGCTCAGTCTCAGCGGGAATGTCCTTAAACACTTCAAGGACTCAGTCTTCGACCAC
TTGAGCTCACTCCGTGTGCTTCGGCTCCAGAAGAATGAGATCACATCGGTGAGGAAGGAGGTGTTTGGGG
CTGTTCTGGCCAACCTCAGCCAGCTGGTCATGAATACGAACCCGTTCGACTGCACTTGTGACAGCATTCT
GTGGTTCGCAACATGGCTTAACAGAACCAATGCCAGTGTACCGGGACTCAGGGATGAGTACATATGCAAT
ACGCCACTGTCCTACTATAACCACTCAGTTCTGGAGTTTGACCCACTCTCCTGCCAGGACATGACACCAT
TCCAAGCTCTCTATGTGCTGACCACCACAGCGGTCCTCATCCTAATGGTCACTTCATTCCTGGTGCGCTT
CCACGGATGGAGGATCCAGTTCTACTGGAACGTACTGATAAACCGTACTCTAGGATTCAGCGACACCAAT
TCCAGAGAAGGAAGAGCATTCGAGTACGACGCTTACGTCGTTCATGCTGCAGATGATACTAACTGGGTGG
AAAGGAGTTTGCTCCCCCTGGAGGACAATCAGGGATATTCATTTTATCTGCAGGAGAGAGATGCAGTACC
CGGTAACGCACATCTGGACTCTATTGCAGAGAACATGAAGAAGTCCAGGAAAATCATACTTGTGGTCACT
GAAAAACTTCTTAATGACCCCTTTTGTAGACATTTCACGGCCCATCAAGCCCGGCACCAGGTTATTGAGG
ACAGCCGGGACGCTGTGGTGCTGGTGTTCTTGGAGGATGTGCACGACTACAGGCTATTCCGTCACCTGCG
CCTGCGCAGGGACATGTTGCGTCAGCGCTGCGTCGTCAGCTGGCCTGTGCAGAGGGAGAGGGTACCAGCC
TTCCACCAGAGTCTGTGCATCGCCCTCGGCACCACCAGCAGAGTTCAGTAGCTCGTAAAACCGGAGTGTT
TTGACTCTGGTCGAGTGCTGGTACTTAGTTCAAATAATATCTTCTTCATCTCCCCAAAGCAGTTTGACGG
CTATGGCGATATTGCATGTCTTAACTTATCAAGAAATGGATTTTCATCAGCACTGAATGGGACAGAGTTC
ACAACATTACCAGACCTAAAATATCTGGATCTGTCCTTTAACAAGATAGACTTGGCCTATGACTATGCCT
TCAAGGAGTTAAAGAAACTAGAAGTACTAGACCTAAGCTACAACAGCCACTATTTTGAATTGTCTGGTGT
GACACACAATTTGAATTTTCTGAAGAATCTACCAGCTTTAAAAGTACTGAATATGTCAAACAATTACATT
TTTACATTAACCACTAAGCAGATGGTTAGCAAATCTTTGAATGAGCTTCAGTTTCAAAAAAACAAGTTGT
CAACTTTATGGAAAGAGAGGGATGGCTCGTACAACAACCTTTTTAAAGATCTGTCAAATTTGACCTATTT
GGATATTTCATTCAACCAAATTGATAAGATCCCAGAAAGAGTATATGAAAACCTACCACATACCCTTACA
ATATTATGCATCAGTAATAATTTGCTCACCAATTTCTACTGGGATATACTCACTAGATTCCAACATCTCA

ATATATTGGATCTAAGCTATAATTCTTTGAAACATGTATCAGCAAATCTCTCGAACTTTACAAATTCACT
TCAGATTCTTAACTTGAGCCACAATCATATTTCTCAACTCTCTGATGGATTTCTGAGGGATACTTACTCC
CTTCATACACTTGACCTCAGCCACAACCAACTGAAAATCATCAACCAGACAACCTTCCAATGGGGCCCTA
AATACTACTTGAAAACCTTGTCCTTGCTAGGTAATCCATTCCAGTGTACATGTGATGTAATAGAATTTAT
CCTATGGATTGAGAAAAACAACGACATTACGATCCCCAGACTGGCTACTGAGGTGAACTGTAACATGCCA
GCCAATAGGAAAGGCCAACCAGTGATACTATTTGACATTGAGGAATGCATTAATGACAAAATATCCTTCA
TGATTTATTCCCTCTACACTTCCTTGATAATGGTCACCATGGTCATCGGTATGGCAACACATTTGTTTTA
CTGGGATGTCTCCTATATTCTCTACTACCTGAGGGCGAAGCTAAAGGGCTACCATTCCCTGCAGTCAACA
ACCAAAGACAATCTCTATGATGCCTTCATAACCTACGATACTGGAGACCAGTTGGTGTCAGACTGGGTGT
TGAACCACCTGCGTTTCCAGCTGGAAGAGAGGGGTGAGAGACATCTTCCTCTCTGCCTGGAAGAGCGAGA
CTGGCCCCCTGGTGTCCCCCTGATAGAAAACCTCTCCCAGAGCATCAGACAGAGCCGTAAGACTGTGTTT
GTGCTGACCGAGGCCTACGTCAGGTCTGGGAACTTCAGGATGGCTGTGTTCCTGGCTCACCAGAGGCTGC
TGGATGAGAACATGGACGTGATCGTCTTGGTTCTCCTGGAGCCAGTGCTGCAGCACTCCTACTTCCTCCG
TCTGCGGCGGCGTCTCTGTGGGAGGAGTGTCCTGGAGTGGCCCCGGACTGCAGCTGCAGAGCCCTGGTTC
TGGCAGCACCTGAGGAACGCTGTCAGGGTGGACAACCAGGGGATGTACAACAAGATCTACTCCAGGTACG
CAGTCCAACAAGATCTTCATGGTGATGTCTTACATCCCTTGCATTTGTTCTATATTATTGGTTACCCTTG
GGTACATGTTGGAGAGTGGCTGGCAAGAGGGCCTTCCCAGGACCTGCACTGAGCTTTACTCCCATTTCTG
TGCCATGAGGGCTGAAGCCGGTGAACCCAGAGGCAGAGAGTCCAAGATGGACCCGCTTTATGGCAACAAT
CGCAAGCTGTTGGCTAGTTTGGGCCGACTGGCATTCTATGGACTTCTAAAGCACAAGTATACTTTCAGTG
AACAGGACATGAGGGCCTATGGAGTAGACCTTCCATCCACACAAGGTAGCCTCGGAACTGGAATACTAAT
TCGCAAAGAATCTCTGATTTGTGTGGCCTACCGTTTTAGCCACCTTACTGTGCAGGAGTTCTTGGCAGCT
ACATATTACCACATTTCCTCCAAACGGGCTATCTTTGACATGTTCACCGAAAGTGCCATGTCCTGGCCCA
AGATCGGCTTCCAGAACCACTTCAGAAGCGCACTGCAGAGGTCCCAGCAGGCTGAAGATGGTCACTTGGA
TGTGTTTGTACGGTTTCTGGCAGGGTTGCTGTGTCCGGCAGCAGTGAGGCCACTGGCAGGGTTGCTGGCC
CAGGGCAAAGACGATGGGGGCCTCCGGGTTTGGGCGGCAGGGCTTCTCCAGGGCCTGCTGGGCAGCGGGG
GAGCTGTGGTGTCCCTGCGTTCAGTCAATGTGGCCTACTGCCTGCAGGAGTTGCAGCACACAGAGGTGCT
ACGCAGTGTCGAGGAGGATCTGCGCTGTGGCAGCCTGGGAGGGAAGCTAACACGCACACACTGCACTGCG
CTGGCCTACCTGCTGCATGTGTCTCCAGAGTGCAACGAGGAGACCAGTCTGTCGGCCTGTCTCGACTACA
GCACCGTCAAGAGCCTGCTGCCTCAGCTGCTGTACTGCAACCATCTTAGATTGGAAAATAACCATTTTAA
AGATGATGTCATGGAACTACTGGGAAGCTTACTGAGTGCGAAGGACTGTCATCTTCAGAAGATAAGTCTG
GCAGAGAATTCCATCGGTAACAAAGGTGCCAAAGCCCTGAGTCGAGCACTGCTGGTTAACCGGACCCTGA
ACACACTTGATCTCCGTAGCAACAACATTGGATCTAAAGGCACAAAGTTTCTATCAGAAGCCCTCAAGAT
GAATCAGTTCCTGGTGTCCATTAATTTACAGAATAATCTGATTGAGGAGGAAGGAGCTCGTGCTCTGGCT
GATGTGTTGCTGTCCAAATGCAAGCTTGTCTCGCTCAATGTACAGAAGAATTGTATTGGACCGGATGGAG
CCAAGAGACTTGCAGAAGCACTGAAGACAAACCGGACACTTACAGAGCTGATTCTGTGCAGTAACCAACT
GGGGGACAAAGGCACTGCAGCCCTGGCCCAGGCCCTGACAGTCAACCACTCTCTCCTTTCATTACATCTG
CAGAGTAACTCCATCAGTAACAAAGGGATGACCGCATTAACCAAGGCTCTGCGGTTGAATCGTGGACTCA
TCTGTCTGAATCTGCGTGAGAACTCAATTGGAGTGGAGGGGGCTAAGGATATGGCCAGGGCTCTGCAGGA
GAACAGCACTCTCCAAGATCTTGATCTCACAGCGAACCTGCTGCACGATGAAGGGGTGAAGGCCATTGCA
GCAGTTTCTGGATGGAGATGCATGGAAGAGCCGGTGGCTTGTATCGAAGCACAAGTCAGACTATGGAGAG
TGGAAACTGACTGCTGGGAAGTTTTATGGCGATTCTGAGGCAGATAAAGGTCTGCAGACCAGCCAGGACG
CCCGTTTCTATGCCATCTCCAGCCGCTTCGAACCCTTCAGCAACGAGGGCAAGTCCCTGGTGGTCCAGTT
CACTGTTAAGCACGAGCAGAAGATCGACTGCGGGGGTGGATATGTCAAAATCTTTCCAGCTGACGTGGAC
CAGGCTGATATGCATGGGGACTCCCAGTATTACATAATGTTTGGCCCTGACATCTGTGGCTACAGCACCA
AGAAGGTTCACGTCATCTTCAACTACAAAGGCAAGAACCATCTCATCAAGAAAGAAATCAAATGCAAGGA
CGATGAGCTCACACACTTGTACACTCTGATCCTGAACCCGGACCAGACCTACGAAGTGAAGATCAACAAC
GAGAAGGTAGAATCAGGCACCTTGGAGGAGGACTGGGACATGTTGCCCTCAAAGAAAATCAAGGATCCCG
AAGCCAAGAAACCAGAGGACTGGGATGACAGGGCCAAGATTGACGACCCTACTGACACCAAGCCAGAGGA
CTGGGAGAAGCCTGAGAACATCCCTGACCCTGATGCTAAGAAGCCTGACGATTGGGACGAGGACATGGAC
GGAGAGTGGGAGCCCCCTATGATCCCAAACCCAGATTACAAGGGTGAGTGGAAGCCGAAGCAGATTGACA
ACCCTGACTACAAAGGTGTCTGGGTGCATCCTGAGATCGATAACCCTGAGTACACACCTGACGCCTCCAT
CTACAAGTTTGACAACATTGGAGTGCTTGGATTGGACCTGTGGCAGGTGAAGTCTGGAACCATTTTTGAT
AACTTTTTTATCGGAGATGATTTAAAGGAAGCTGAGGAGTTTGGGAAAGAGACGTGGGGAGCTACAAAGG
AACCAGAAAAGAAAATGAAGGACGCCCAGGAGGAGGAGGAGAGGAAGGCAAGAGAAGAAGAAGAGAAGAG
CAAGAAGGACACGGCTGACGATGAAGGGGACGAAGAGGAAGGTGATGAGGATGAACCAGAAGAAGAGGAA
GAGAAAGACAACACAACGGAAGAGGAAGAAGAAGAAACTCTCAAGAAGGACAAGGATGAGAGCAGTAAAC
AGAAGGTGTTGGTGATGGAGTACTGTTCAGGAGGCAGTCTGCTCAGTCTTCTGGAGGAGCCAGAGAATGC
CTTCGGTCTGCAAGAGACAGAGTTCCTAATTGTACTACAGTGCGTGGTTCACGGGATTAACCATCTGCGT
GAGAATGGGGTAGTCCACCGGGACATCAAACCAGGGAACATCATGCGCCAGCTGGGAGACGACGGGAGGT
CTGTCTATAAGCTAACAGACTTCGGTGCGGCCAGAGAGCTAGAGGATGACGAGAAGTTTGTCTCGATCTA
TGGAACAGAGGAATACCTGCACCCGGACATGTACGAGAGGGCAGTGCTGCATAAGCCCCAGCAGAAGACA

```
TATGGTGTGAGTGTGGACCTGTGGAGTATCGGAGTGACCTTTTACCATGCTGTTGCCGGTACCTTACCTT
TCATCCCCTTCGGAGGACCCCGCAGAAACAAACCAACCATGTTTAAAATCACCACAGAGAAACCTGCAGG
AGCCATTGCTGGAGTCCAGAAGGTCGACCACGGGCCTATTGAGTGGAGCTACACACTGCCACAAAGCTGT
CAGCTCTCCAAGAGTCTGAAGGATCTATTGGTGCCAGTGTTAGCGGGTGTACTG

>Salmo salar tlr3-tlr8-nlrc3-calr-ikbke
CCTTTGCTGCCCTACAGAGTCTGACACTGCTGGACGTGTCCAAAAATGACCTGAAGACAGCCAAGTTGGG
CACCCGCCTTCAGCTTCCCAGCCTGGTGACTCTCATCTTGTCTAGCAACAGCATATCAACCATCAAAAAG
GATGACTTCTCTTTTCTCAGAAATTCCTCATCCCTACGAGTCCTTCACCTGTCGGATCTAATAACTCAAG
CAAAGTTTGAGCCTGATTGCTTGAAGCCCATTGCAAGCATATATGAGTTAGTCATGAATGGGAGCAAACT
TGGCCCTTCACTCACATCTAAACTTTGCACAGAGCTTTCTGGGACAGCGATCCGCAGCCTCTCCCTCCAG
AAGACACAGCTAGTTACACTAGACAACACCACCTTCAAAGGCCTGGGGAAGACCCACCTCACTACTTTGG
ATCTATCCCACAACAGCATAGCTAAGATCGGGGACGGTTCTTTCCAGTGGCTGCCCATGCTGGAAGTTCT
TTCTCTAGAGCAGAACAACCTCAAGCACCTGACTAAGAACACTTTCCACGGGCTGGGGAATCTGACACGG
CTCAACCTGAATATGGCGCTGGTGAAGAGTCGCACTTCTTCTTACCCTATTATCGATGACTTCTCCTTCC
AGCCACTAGGAGCACTGGAGAGCCTGAGCATGGAGAATACTGCCTTTCGAAACATCTCAGTGTTCACCTT
TGCGGGTTTGATGAGTCTCCGCCAACTCCACCTGAGCGGGACCAGCTGCATGGCGCTCAGAATCATCACC
AACCAGACCTTTGTGTCCCTCGCAGATTCACCACTTCTTACGCTAAAGCTTACACGCACAGCAATCTCCC
GTCTAGACCCTGGAGCCTTCTCCAGCCTGGGCAATCTCACCACCCTTCTGCTGGGCAACAACTCCATCTC
CCAGACCCTGACGGGGAAAGAGTTCCAGGGTTTGGGCCAGCTACAGGAGATCTACCTCTCTAACGGCAAC
CAGAAGCTCATCCTCAGCCCTATGTCCTTCGTCCATGTGCCCGCTCTCAGGACTCTGATGCTGGGGAGAG
CTCTGACCAGCACCCAGTATATGAATACCTCTCCTTTCAAGCCTCTGTCCAACCTCACCATCCTGGACCT
CAGCAACAACAACATCGCCAACATCAAAATTGATCTTCTGGACGGACTAGAGAATCTGAAGGTGCTGAAG
CTACAGCACAACAACTTGGCCCGGCTGTGGAAGAGTGCCAACCCGGGTGGGCCGGTGCTGTTTCTTAGGG
GGCTCCGCAGCCTTGTTGCCCTGGAGATGGACTTCAACGGTCTGGATGAGATCCCTGAGGAGGCCCTTCA
TGGATTAACCAACCTTCAAGAGCTCAGCCTCAGTGGGAACATCCTGAACCAACTCAAGGACTCAGTCTTC
AACGACTTGGGGTCACTCCGGGTGCTTCGGCTCCAGAAGAACCTAATCACGTCAGTGAGGAAGGAAGTGT
TTGGGCCGGCATTGGCCAACCTCAGCCAGCTGGTCATGGAGAAGAACCCGTTCGACTGCACCTGCGAGAG
CATCCTGTGGTTTGTGGCGTGGCTGAACGGAACAAACGCCAGTGTGCCGGGCATCAGGGATGAGTATGTG
TGCAACACGCCGCAAGCCTACTACAACCGCTCGATCATGGAGTTCGACAGGCTCTCCTGCCTGGACATGA
CACCATTCCAGGCGCTCTACGTGCTCACCAGCACTGCGGTCCTGACCTTAATGGTGACCTCACTCCTAGT
GCGCTTCCAGGGTTGGAGGATTCAGTTCTACTGGAACGTTCTGATCAACCGTACGCTCGGATTGAGCGAT
GCCAGTTCCAGGGAGGGGAGAGAGTTCAACTATGACGCGTTCGTCATTCATGCTGCAAAGGATAAAACCT
GGGTGGAACGAAGCTTGCTCCCCATAGAGGACGAACAGGGGTATAGATTTTATTTGCTGGATCGAGATGC
AGTGCCAGGTGATTTGCGACTGGAGTCCATTGTGGAGAATATGAGAAGGTCCAGGAAAATACTATTTGTG
GTCACCGAAACTCTTCTCGAAGACTCCATGTGTCGACAGTTCATGGCCCACCATGCCCTGCACCAGGTGA
TTGAGGACAGCCGGGACTCGGTGGTGCTGGTCTTCCTGGAGGACGTGCAGGACTACAAGTTGTCTCGCTG
TCTGCTCCTGCGCAGGGGCATGCTTCGTCCACATTGCCTCCTCAACTGGCCACTGCAAAGGGAGAGGGTG
CCCGCCTTCCACCAGAGGCTCCGCATCGCCCTGGGCACCACCAACCGAGTGCAGTAACTTGTGAAGCAAG
AGTGCTTTGACTCTGGTCGAGTGCTGGTCCTGAGTTCAAATAATATCTTCTTCATTTCTCCAAGGCAGTT
TGAGGGCTATGGTGACATTGCATGTCTGAACCTATCAAGAAATGGATTTTCTGCAGCACTGAATGGGACA
GAGTTCACGACGTTACCAGCCCTAACATATCTGGACCTGTCCTTTAACAAGATTGACCTGGCCTATAACA
ATGCATTTAAGGAATTACACAAACTACAAGTATTAGACCTAAGCTACAACAGCCACTATTTTGAAGTGTC
AGGTGTGACACATAATTTAAATTTTTTGACAAATCTACCGGCTTTGAAAGTACTGAATATGTCAAACAAT
AACATTTTTACATTAACAACTAAGCAGATGACAAGCGCATCTTTGAAAGAGCTTCAATTTCAACACAATT
TGTTGGCCACATTATGGAAAGAGAGGGACGGATCATACAACAGCCTTTTTAAAAACCTGGTGAATTTAAC
CTATCTGGATATATCATTCAACAGCATTGAGAAGATTCCATCAACAGTCTATGAAAACTTACCGAATACC
CTTCAGAAACTATGCATTAGTCATAATTTACTCACCCATTTTGATTGGGATAAACTGGCTAGTTTCCAAC
AACTGAATATTCTGGATCTAAGCTATAATTCTTTATATCACATGTCAGCAAATCTCTCAAACTTCACAAA
TGCACTTCAGATGCTTGACTTGAGCTACAATCAGATTGCTCAACTCTCTGATGGATTTCTTAAGGGTGCT
CAAAGCCTTCAGATACTTGACCTCAGCCACAACCAACTGACTATCATCAACGAGACCACCTTCCTATCGG
GCCCTGAAAACTACATGAAAACCTTGTCCTTGCAAGGTAATCCATTCCAGTGTACCTGTGACTTATTAGA
GTTCCTCATGTGGATTAAGGATAACAAAGACATAGAGATCCCCAGACTGGCCACTGAGTTGATCTGCAAC
ATGCCAGCCAAAATGAAAGGCCAACCAGTGATACTGTTTGACAAGAAAGAATGCCTCAATGTCAACAAAG
CCTTCCAGATTTACTCCCTCTCCACTTCCTTGATCATGCTCACCATGGTCATCACCATGGCAGCTCATAT
GTTTTATTGGGATGCTTCCTATATGCTATACTACCTGAGGGTGAAGCTGAAGGGATACCATTCCTTTAGG
TCCGCAACAACAGACAATCTSTATGATGCCTTTGTTACCTACGACACCAGAGACCTGTTGGTGTCAGACT
GGGTGCTGAACCACCTGCGGGTGCAGCTGGAGGAGAGGCAGGAGAGACACCTGCCTCTCTGTCTGGAGGA
GCGAGACTGGATCCCAGGGGTCCCCCTGATAGACAACCTCTCCCACAGCATCCGACAGAGCCGCAAGACT
GTTTTTGTGCTGACCGAGGCATATGTCAGGACCGGGACCTTCAGGATGGCTGTGTACCTGGCCCACCAGA
GGCTGCTGGATGAAAACATGGACGTGATCGTGCTGGTTCTCCTGGAGCCTGTACTGCAGCATTCTCACTT
```

CCTCCGTCTGCGGCGGCGTCTGTGTGGGAAGAGTGTTCTGGAATGGACCCGGACTGCAGCCGCAGAGCCC
TGGTTCTGGCAGTGCCTGAGGAACGCTGTCAGTTTAGACAACCAGGTGATGTACAACCAGATGTACTCCA
GGTACGGAGTCCAACAAGATCTTCATGGTGATGTCTTACATGCCTTGCATTTGCTGGATAGTGGCGGTTA
CCTTTGGGTACCTGTTGGAGAGTGGCTGGCAGGAGGGTCTTCCCAGGACCTGCACTGAGCTCTACTCCCA
CTTCTGCGCTATGAGAGCCGAAGCAGGTGAGCCCAGAGGCAGAGAGCCTATCAAGATGGACCAGCTTAAC
GGGAACAACCGCAAGCTGCTGGGAAGTCTGGGCCGACTGGCCTTCTACGGACTTCTGAAGCACAAATACA
CCTTCAGTGAGCAGGACATGAGGGCCTATGGAGTAGACCTTCCATCCACCCAGGGTGGCCTAGGAACTGG
AGTACTGATCCGCGAGGATTCCCCGAATTGTGTGGCCTACCGCTTCACCCACCTGACTGTGCAGGAGTTC
TTAGCAGCCACATACTACCACATCTGCTCCAAACGTGCCATCTTTGACCTGTTCACCGAGAGCGCCATGT
CCTGGCCCAAGATCGGCTTCCAGAACCACTTCAGGAGCGCCCTGCAGCGGTCCCAGCAGGCAGAGGATGG
TCACCTGGACGTGTTTGTGCGGTTCCTGGCGGGGTTGCTGTGTCCAGCGGCAGTGCGGCCGCTGGCAGGC
CTGCTGGCCCAGGGGAAGGACGATGGGGGCCAAAGGGTTTGGGCGGCTGGGCTGCTGCAGGACCTCCTGG
GCAGCGGTGGTGCTGTGGTGTCACTACGCTCAGTGAACGTGGCCTACTGCCTGCAGGAGCTGCAGCATAC
AGAGGTGCTACGCAGCGTGGAGGAGGACCTGCGCTGTGGTGGCCTGGGAGGGAAGCTGACACGGACACAC
TGCACAGCGCTGGCCTACCTGCTACAGGTGTCTCCAGAGTGCAGCGAGGAGATCAACCTGTCGGCATGTC
TGGACTACTCCACTGTAAAAAGCTTGCTGCCTCAGCTGCTCTACTGCAGCCATCTCAGATTGGAAAATAA
CCATTTTAAAGATGATGTCATGGAACTGCTGGGAAGCTTACTGAGTGCAAAGGACTGCCACCTTCAGAAG
ATAAGTCTGGCAGAGAACGCTATCAGCAACAAAGGGGCCAAGGCCCTGAGTCGAGCACTACTGGTTAACC
GGACGCTAATTACGCTTGATCTCCGTAGCAACAACATTGGATCTAAAGGCGCCAAGTTTCTAGCAGAAGC
CCTCAAAATGAATCAGGTCCTGGTGTCCATCAATTTCCAAAATAATCTCATTGAGGAGGAGGGCGCTCGC
GCCCTGGCTGAGGTGCTTCTGTCCAACCGCAAACTTGTCTCGCTGAATGTACAGAAGAATTGTATTGGAG
CAGATGGAGCCAAGAGGATTGCAGAAGCGCTAAAGACAAACCGGACACTTACAGAACTGATTCTGAGCAG
TAACCAGCTGGGGGACAAAGGCACTGCAGCCCTGGCCCAGGCCCTGACCGTCAACCACAGCCTCCACTCG
CTGCACCTGCAGAGTAACTCCATCAGTAACAAAGGGATGACTGCGTTAACCAAGGCTCTGCGGTTGAACC
GTGGACTCATCTGTCTGAACCTGCGTGAGAACTCGATCGGGGTGGAGGGGGCGAAGGATATGGCCAGGGC
ACTGCAGGAGAACAGCTCTCTCCGACACCTGGATCTTACAGCCAACCTGCTGCACGATGAGGGGGTGAAA
GCCATAGCTACAATTCCTTGATGGAGATGCCTGGAAGACTCGCTGGCTGGACTCTAAGCACAAGGCAGAC
TACGGAGAGTGGAAACTGACAGCTGGTAACTTCTATGGAGATGCTGAGAAGGACAAAGGTCTACAGACCA
GCCAGGACGCCCGTTTCTATGCCGCATCGGCCCGCTTCGAACCCTTCAGCAACGAGGGAAAGACTCTGGT
GATCCAGTTCACTGTGAAACATGAGCAGAAAATTGACTGTGGCGGCGGTTATGTCAAGGTGTTCCCCTCT
ACCCTTGACCAGGCTGACATGCACGGAGACTCCCAGTACTACATCATGTTCGGCCCTGACATCTGTGGCT
ACAGCACCAAGAAGGTCCACGTTATCTTCAACTACAAGGGCAAGAATCACCTCATCAAGAAGGAAGTCAA
GTGCAAGGATGACGAGCTGACCCACCTGTACACGCTGATCCTGAACCCAAACCAGACCTACGAGGTGAAG
ATCGATAACGAGAAGGTTGAGTCAGGCACTCTGGAGGAGGACTGGGACTTCCTGCCGGCTAAGACCATCA
AGGACCCCGAGGCCAAGAAGCCCGACGACTGGGACGACCGCCCCAAGATGGACGATGCTGAAGACGCCAA
GCCAGAGGACTGGGATGTGGCTGAGAACATCCCTGACCCTGATGCCAAGAAGCCTGATGACTGGGATGAG
GACATGGATGGAGAGTGGGAGCCACCTGTGATCACCAACCCAGAGTACAAGGGAGAGTGGAAGCCCAAGC
AGATTGACAACCCTAACTACAAAGGAGCCTGGATCCATCCTGAGATCGATAACCCAGAGTACGCCGCTGA
CTCTACCATCTACAAGTTTGATGACATCAGTGTCCTGGGCCTGGATCTGTGGCAGGTGAAATCTGGCACA
ATCTTTGACAACTTCCTGGTCTCAGATGATGTGAAGGAAGCAGAGAAGTTTGGAGCTGAGACATGGGGTG
TTACTAAGGAGCCAGAGAAGAAAATGAAACAGGAGGAAGATGACAAGAAACGTAAAGAGGAGGATGAGAA
AAACAAAGAACAAGCAACTGAGGCAGAAGAGGAAGGTGAGGAAGAGGAAGGGGAGGATGAAGGGGAGGAG
GAGGAGACGCCAGAGGAAGGAACTGAGGAAGAGGAGGCTCCAGGGAAAGATGAGAGCAATAAACAAAAGG
TGTTGGTGATGGAGTATTGTTCAGGAGGCAGTTTGCTCAGTCTGCTGGAAGAGCCAGAGAACGCCTTCGG
TCTGCCTGAGACAGAGTTTCTCATAGTACTACAGTGTGTGGCGCACGGGATGAACCATCTGCGTGAGAAT
GGGGTGGTGCACCGAGACATTAAGCCTGGGAACATCATGCGTCAGGTGGGAGAGGACGGGAGGTCCGTCT
ATAAGCTAACAGACTTCGGAGCGGCCAGAGAACTAGAGGATGATGAGAAGTTTGTCTCCATCTACGGAAC
AGAGGAATACCTGCACCCAGACATGTATGAGCGTGCAGTACTGCGTAAGCCCCAGCACAAGACCTATGGT
GTGAGTGTTGACCTGTGGAGTATTGGAGTGACGTTTTACCACGCTGCCACTGGTTCTTTACCCTTCACAC
CCTACGGAGGACCCCGCAAGAACAAACCCACCATGTATAAGATCACCAAAGAGAAGCCAGTAGGGGCCAT
TTCTGGAGGTCAGAAGGTCAACGACGGACCTATAGAGTGGAGCTACCACCTACCAAACAGCTGCCTGCTC
TCAAAGGGTCTGAGGGGTCAGCTAGTCCCAGTGTTAGCTGGGATACTG

>Lepisosteus oculatus tlr3-tlr8-nlrc3-calr-ikbke
CTTTTAAATGTCTCCAGAGTTTGAAAAGGCTTGACGTGTCTCGCAATGACCTGACCTCTGCAAAGCTTGG
CACACATCCTCAGCTCCTGAACCTTGAAGAGCTCATACTATCTGGGAACGCAATCTCTGTTCTAAAGGCC
GATGACTTCTCTTACCTCAGCAACGCCTCACTGAAGATCCTGAAACTATCTGCTCTCCCTCTTAAAAAGT
TTGAGCCGGGCTGCTTCCAGTGCATTGGAAGACTCAGTGTCCTTGTTATGGATGGCAGTAAACTGGGTCA
TCCCCTGATGGCCAAGCTGTGCTCTGAATTGTCGGAAACCAAGATTCGTAGCCTTTCGCTGCAGGACACC
TCGCTATTGACCTTGCTGAACACCACTTTTAAAGGACTGCAGTCTACTAACCTTACGTCTATGGACCTCT
CTAAAAACGAAATACCTGCAGTGCAGCGTTCTTCTTTTCAGTGGCTTCACAGTCTAGAAAATCTATCCCT

TGCGGAGAATAACTTTAAACGTTTTACTAAGGACATGTTTATTGGTTTGGGTAGCCTAAAATTTTTAAAT
CTCACCAAGTCCCTTAAAAAGCACAAGTCGTCTTCTTATCCGGTGATTGATGACTTTGCTTTTCAGAATC
TGTTGAACCTGGAGTTTCTAGCTATGGAAAGCAATGCATTCCGAGAAATCACAGGTAACACTTTCACAGG
GTTAAAAAGCCTAAAGTATCTGAGTTTGAGCCACAGTTATTTTAACTTGAAAACTGTGACTAATGTGACA
TTTTCATCTCTGGCCCAGTCTCCCCTCAAGATCCTTGACCTCACGAAAACAGGAATCTCATACCTGGATG
TTGGAGCTTTCTTTTGGCTTCGAAATTTAAGTAAAGTGTACATGGGTCAAAACAAAATTTCACAGGTCTT
AGGAGGAAGGGAATTTGAAGGACTTAACAACATAGAAGAAATTTTCTTGTCTTACAACAAAAAAATCACT
TTGACCCCTTCGTCATTCAACTACACCCGCACTCTGAGAACCCTGATGCTTGGAAGCAATCTGATTGACA
GTCTGGACCTGCATCCTTGTCCATTTCAGCCTTTAGGGAATCTCACTGTATTGGATCTCAGTAATAACAA
TTTAGCTAACATAAATGCTGGTCTGTTTAGTGGCCTTCATGATCTAAAGATTCTGAAGTTACAGCATAAT
AATTTAGCTCGCCTGTGGAAAAAGGCCAATCCTGGGGGACCTGTACTCTTTCTCAAGGACGTGCCTCAGC
TTGAAGTACTTCAGCTGGACTCGAATGGATTTGATGAAATCCCTGTTGATGGCTTCAAAGGATTGTTTCA
CCTTCTGGAGCTCGACCTGGGTTTGAATTACCTGAATGTACTTCCGGACAACGTTTTTAATGACCTCACG
TCCGTGAGAGTGCTGAAGCTGCAGAAGAATTTCATCACGTCTGTGAAAGAGACCGTGTTTGGGCCAGTCT
TCCGCAATCTGAGCGTGCTGCATATAGAGAAGAATCCTTTCGATTGCACTTGCGATAGTGTCCTGTGGTT
TGTCAACTGGCTCAATGTGACCAATGCCAGTGTTCCTCAGTTAAGCTCCCAATACGTTTGCAACACACCT
CCTAAATACTACAACAGCTCACTTGTCCTGTTTGACACATCGCCCTGTCAGGACCAGGCCCCATTCAAAC
CCATGTTCGTTACCACCAGTAGCTTTTTGCTCACATTCATGGCAATGGCCCTTCTCATCCATTTCCAGGG
GTGGAGAATCCAGTTTTTCTGGAATGTCCTGGGTAATCGGATTCTTGGGTACAAGGAAGTTGACTTGGGG
GAAAACAGGTTTGAATATGATGCTTATATAGTCCACGCCCAAGAAGACTGGGAATGGGTGGAAAGGAATC
TTACTCCCTTAGAAGAAAATCCTTTTACATTTTGCTTTGAGGATCGGGACTTTCTGCCTGGGACACCACA
CTTAGAAAATATTGTTGAGACAATTCGCCAGTGCAGAAAAATAGTATTTGTTGTAACCGAAGCTCTTTTA
AAGGACCCTTTATGCAGAAGGTTTACGGTTCATCATGCTTTACATCAGGTAATCGAGGAGAGCCGTGACT
CGATTGTTTTGATCTTTCTGGACGACATCCCGGACCACAAATTAAATCACTGTCTGTATATCCGGCGGGG
CATGCTGAATTCACGCTGCATTTTAAACTGGCCCCTACAGAGGACACGCATTCCAGCATTTCACCAGCAT
CTGAAGGTGGCACTGGGTCTGAGTAACAGAGTGCATTAACTTGTTAAACACGAATGCTTTGTTTATGGTG
AAGTCTTAGATCTTAGTTCAAATAACATCTTTTTCATTTCCCCAGAGCAATTTGAAGGCTTTGAGAAAAT
CTCCTGTCTTAACCTCTCAAAAAATGGTGTCGCGGCTGCTTTGAACGGCACTGAATTCATAAAGCTTCCA
AAACTAAAGTACCTAGACCTGACCTATAACAAGATTGACTTAGCCTATGATTATGCATTTACAGAGTTAA
AGGAGCTGGAAGTGTTGGATCTCAGTTTCAATCCTCACTATTTTGCGGTATCAGGGGTGACACACAATTT
AGCCTTCTTAAAACATCTCCAGTTTCTGAAAGTGCTGAACCTGAGCTGGAATGAAATATTTACGTTAACA
ACAAAAGAGATGATCAGTGAGTCCCTGGAAGAGCTTCAGTTCCAAGGCAACCGATTGGATAAGCTGTGGA
AAAAGAATGATGGTTCCTATATTAGGCTTTTTGAAAATCTTACTAATTTGTCATACCTTGACATATCTTA
TAACAAGCTTAACAAGATACCATCTACTGTCTATGCCAGCTTGCCACAAAATATAAGTAAACTTTGTTTA
AGTCATAATGAACTTACTTGTTTTGATTGGGAAGAGCTTAAACATTTAAGGCAACTGAAAGTTTTGGATC
TACAGCATAACAAGTTGACTAAAGTGTCAGCAGATCTTACAAACTACACAAAAACACTTCAGATTCTTAA
CTTGAGTTACAATAGGATTACTCATCTCTTTGATGGCTTTCTACGGGGTGCAAAGAGCCTTTTAATCCTT
GATTTAAGTCACAATAAACTAAGGCTTATAAACCAGTCAACTTTCCTCTCTGGACCAGACAACTACTTGG
AGGTACTGTCTCTAAAAGGTAACCCCTTCCAATGTACCTGTGAATTGTTAGAATTCATTTTGTGGATTGA
AACAAATGATGTTGATATACCATTGCTGGCCACAGATGTAAGCTGTGCAATGCCAGCAAACAGAAAAGGA
ATTGGAATTATATATTTTGACCTTAAAGAATGTATTGATGACAGCAAAGCAGCAAAATTTTCTTTTCTGT
CTTCCTTTTTTATTTTGTTTACTATGACTGCTGCTCTTACTATGCATTTGTTTTACTGGGATGCTTGGTA
TATTTGTCACTATCTTAAAGCAAAAATGAAGGGTTACCATTACCTGAAATCCACAGAGAGCATCTATGAT
GCTTTCATCACCTACGATACTAAAGACCCAGTTGTTTCAGACTGGGTTCTGAATCATCTCCGAGTCCAGC
TGGAGGAAAACGGAGAAAAACTATTCCCAATTTGTCTGGAGGAACGAGACTGGCACCCCGGCGCACCTGT
CATAGACAACCTGTCTCAGAGCATCAAGCAGAGTCGAAAGACAGTGTTCGTGCTGACTGAAGGGTATGTG
AAGAGTGGGACCTTTAAAATAGCCTTTTACCTCGCACACCAGCGGCTGCTGGATGACAACACGGACGTTA
TCGTGCTGGTTCTGTTAGAGCCAGTGCTGAAGCATTCTCAGTTTTTCCGCTTAAGGAAAAGGTTGTGCAG
GAAGACCATCCTTGAGTGGCCCACAAATCCTCATGCGGAAAAATGGTTCTGGCATTGTCTGAGAAATATC
ATAAGAGTGGAACACCAGGCTGTGTATAATAAGCTCTATACAAGCTATGAACTCCAACAAGATCTTCCTG
GTGATGTCCTACGTGCCCTGCGTTTGTTGGATCATTGCCTCCACCCTTGGACACCTCCTGAGGAATGACA
CGCAGGATGGCTTGCCCAGAACGTGGACCGAGCTCTACTCTCACTTCTTCAAGATGATGGTGGAAGGGGA
TTCCAAGGATAAGGAGCCCTTAAAGATTGAACAAGCCAGCGGGAGCAGTCGGAAGCTGATGGGCAGCCTG
GGGCGATTAGCCTTTTACGGGCTGATCAAGAGGAAGTACAGCTTCTATGAGCAAGACATGAAGGCTTATG
GGATTGACCTGCCTTCCTTACAAGGCAGTCTTTGCACTCGCATCTTAGTAAAGGAAGACTCCCCAGTCTC
CACAGTCTATTACTTTGCCCATCTCACACTCCAGGAGTACCTGGCGGCCACCTACTACTACACGGCCTCG
AAGCGCGCTATATTCGATCTGTTCACGGAGAGTGGGATGTCCTGGCCCAAAATAGGCTTCCAGAACCACT
TTAAAAATGCTCTCCAGAGGTCCCAGCAATCCGAGGATGGACAGCTGGATGTTTTTGTCCGCTTTCTCTC
CGGCCTGCTCTCCCCCCAGGTGATCAAGCCCCTGTCTGGACTGCTCTTGCTGGCCAGGGACGAGCACAAC
GGCTACCGAGGTCCCGCCATCTCCTTCCTGCAGAGCAGCCTCAGCTCCGGCTGCACCATCTCCCTGCGGG
CCGTCAACATGGTGCACTGCCTCCAGGAGCTGCAGCACACGGAGCTGGCCCGGACCGTGGAGGAGTCGCT

CCGCAACGGCAGCCTGGCAGGCAAGCTCAACACCGTCAACTGCTCGGTGCTGGCCTACCTGCTGCAGGTG
TCGGAGGAGTGCGCAGAGGAGACCAACCTGTCCAGCTGCCTCAACTACAGCATCGTCAAGAGCCTGCTGC
CGCAGCTCCTCTACTGCAGCAATCTCAAACTGGAGAATAACCAGTTTAAGGATGATGTCATGGAACTGCT
GGGCAGCTTGCTGAGCGCCAAGGACTGTCACATCCAGAGGATCAGTCTCGCTGAGAACGCCATCAGTAAC
AAAGGAGCCAAGGCCATCAGCCGGTCCCTCATGGTGAACAGGAGCCTGACTGCTCTGGATCTCCGCAGTA
ACAACATCGGGCCCAAAGGAGCCAAAGCCCTGGCAGAAGCGCTCAAGATCAACCAGGGCCTGGTGTTTCT
CAGCCTCCAGAACAACTTAATCGGGGAAGAGGGAGCCAAGGGCATTGCAGAGATCCTGATGACAAATCGC
AAGCTGGTCACCCTTCACCTGCAGAAGAACTCCATCACTGCAGAGGGAGGCAAGCTGATTGCCGAGGCGT
TGAAGAAAAACCGGAGCCTGAAAGAACTGATTCTGTCCAGTAACCAGCTTGGTGACAAGGGGGCCACTGC
GCTGGCCCAGGCGCTGATGGAGAACCACAGCCTTGTCGCTCTTCACCTCCGGAGCAATTCCATTAGTAAC
AAAGGGGTCACAGCGCTGACACGGGCTCTCAAGCACAACAGAGGACTGACTGACCTCAACCTTAGGGAGA
ACTCCATCGGTGTGGAAGGAGCGAAGGAGATTGCCAACGCACTACAAGAGAACAGCACACTGAGAAACCT
GGATCTGACGGCGAATCTCCTGCATGATGAGGGTGCCAAAGCCATTGCAGCAATTTCTCGATGGAGATGC
CTGGAAGACCCACTGGGTGGAGTCCAAGCACAAGTCCGACTATGGAGAGTGGAAACTGACTGCAGGAAAG
TTCTATGGCGACGCAGAGAAGGACAAAGGTCTGCAGACGAGTCAGGATGCCCGCTTCTACGCACTCTCTT
CCCAGTTCAAGGCCTTCAGCAACGAGGGCAAGACCCTGGTGGTCCAGTTCACGGTCAAGCACGAGCAGAA
GATCGACTGCGGCGGCGGATACGTCAAGGTGTTCCCCGCCGACCTGGACCAGGCCGACATGCACGGAGAC
TCGCAGTACTACATCATGTTTGGCCCCGATATCTGTGGTTACAGTACCAAGAAGGTTCACGTCATCTTCA
ACTACAAGGGCAAAAACCACCTCATCAAGAAGGAAATCAAGTGCAAGGATGACGAGCTGACGCACTTGTA
CACCCTGATTCTGCGGCCTGACCAGACCTACGAGGTGAAGATCGACAACGAGAAGGTGGAGTCGGGCACC
CTGGAGGAGGACTGGGACTTCCTTCCCCCCAAGAAGATCAAGGACCCCAACGCCAAGAAGCCCGAGGACT
GGGATGACCGGGCCAAGATCGACGACCCCGAAGATGTCAAGCCTGAGGACTGGGACAAGCCTGAGAACAT
CCCTGACCCCGATGCTAAGAAACCCGACGACTGGGATGAGGAGATGGATGGAGAGTGGGAGCCGCCCATG
ATCCCCAACCCCGAGTACAAGGGAGAGTGGAAACCGAAGCAGATTGACAACCCCAACTACAAAGGCGTCT
GGGTCCACCCCGAAATTGACAACCCCGAGTACACCCCTGATTCCACCATCTACAAGTTTGACAACATCGG
GGTGCTGGGTCTGGACCTGTGGCAGGTGAAATCTGGCACCATCTTCGATAACTTCCTGATCACGGACGAT
GAAAAAGCTGCAGAAGAGTTTGGCAAAGAGACCTGGGGAGTGACCAAGGAGCCCGAGAAGAAAATGAAGG
AGAAGCAGGAAGAGGAGGAGAGAAAGCAGCGTGAGGAGGAGGAGAAGAACAAGAAGGAGCAGGAGACGGA
GGAGGATGACGAGGAGGACGAAGAGGAAGGCGAGGAGGAGGATGACGAGGAGGACAAGAAGGAGGAGGGG
GAGGAAGAGGATTCAACGGAAACCGAAGCTCCAGAACAGCAAGCAGAAGGTGCTGGTGATGGAGTACTGC
TCGGGGGGCAGCCTGCTCAATCTCCTGGAGGAGCCGGAGAATGCCTTCGGGCTCCCGGAGACCGAGTTCC
TCATCGTGCTGCAGTGTGTCGTGTCTGGCATGAACCACCTGCGCCAGAACAGCATGGTCCACCGTGACAT
CAAGCCCGGGAACATCATGCGCCTGCTGGGCGAGGACGGCCTCTCCGTCTACAAGCTCACCGACTTCGGG
GCAGCGCGGGAGCTGGAGGACGACGAGAAATTCGTCTCCATCTACGGCACGGAGGAGTACCTGCACCCCG
ACATGTACGAGCGGGCCGTTCTCAGGAAGCCTCAGCAGAAGTCGTACGGCGTCACCGTGGACCTGTGGAG
CATCGGTGTGACGTTCTACCACGCGGCGACCGGCAGCCTTCCCTTCGTGCCGTACGGAGGACCGCGCAGG
AACAAGCAGATCATGTATAAGATCACCACTCAGAAGCCGCCAGGGGCGATAGCAGGGACTCAGAGGGTGG
AGGATGGGCACATAGAGTGGAGCTACGAGCTTCCCATATCCTGCCAGCTGTCCACGGGGCTGAAGGCCCA
GCTGGTGCCGGTGCTGGCCAACATCCTG

>Balaenoptera acutorostrata scammoni tlr3-tlr8-nlrc3-calr-ikbke
CCTTTAAAAACCTGAAGAATTTAATCAAATTAAACCTAGCTCATAATGGTTTATCATCTACTAAATTAGG
AACTCAGCTGCAACTGGAAAATCTCCAAGAGCTTCTACTATCAAATAATAAAATTTCTGCACTAAGACGT
GAAGAACTTGATTTCCTTGGCAACTCTTCTTTAAAAAGATTAGAGTTGTCATCAAATCAAATAATGGAGT
TCTCTCCAGGGTGTTTTCATGCAGTTGGAAAATTATTTGGCTTCTCTCTGAACAATGCCAAACTGAGCCC
CAGTCTCATAGAAGAGCTCTGCTTGGAATTATCAAACACAAGCATTCAGAATCTGTCCCTGAGCAACAAC
CAGCTGTACACAACCAGCAACAAGACTTTTGTTGGGCTGAAGCAGACAAATCTCACAGTGCTCGATCTTT
CCCATAACAGCTTAAGTGTGATTGGTAATGATTCCTTTGCTTGGCTTCCACATCTAGAATATCTCTTTCT
GGAGTATAACAATATAGAACATCTGTCTTCTCGCTCTTTTTATGGGCTTTCCAATGTGAGATACCTGAAT
TTGAGACGATCTTTTACTAAACAAAGCGTTTCCCTGGCTTCACTTCCCAAGATTGATGATTTTTCCTTTC
AGTGGCTAAAATTTTTGGAGTATCTCAATATGGAAGATAACAACTTTCCAGGCATAAAAAGCAATACTTT
CACGGGATTGACAAAGCTGAGATGTTTAAGTCTATCCAACTCCTTCTCAAGTTTGCGAACTTTAACAAAT
GAAACATTTTTATCACTTGCTGGTTCTCCTCTGCTCATACTCAACCTAACCAAAAATAAAATCTCAAAAA
TAGAGAGTGGTGCTTTTTCTTGGTTGGGCCACCTAACTGTACTTGACCTTGGTCTTAATGAAATTGGGCA
AGAACTCACAGGCCAGGAATGGAGAGGTCTAGAAAATATTGTTGAAATCTACCTTTCCTACAACAAATAC
CTAGAGCTGACTAGCAACTCTTTTGCCTTGGTTCCAAGCCTTCAACGACTGATGCTCCGAAGGGTGGCCC
TTAAAAATGTGGATAGCTCCCCTTCACCTTTTCACCCTCTTCCTAACCTGACCATTCTGGATCTAAGCAA
CAACAACATAGCCAACATAAATGATGAACTGTTGAAGGGTCTTGAGAAACTAGAAATTCTGGATTTGCAG
CATAACAACTTAGCTCGCCTCTGGAAGCATGCAAACCCTGGCGGTCCTGTTCATTTTCTGAAGGGTCTTT
CTCACCTCTACATCCTTAACTTAGAGTCTAATGGCTTTGATGAGATCCCAGCAGAAGTCTTCAAGGACTT
ACGTGAATTGAAGAGCATCGATTTAGGATTGAATAATTTAAATATCCTTCCACCATCTGTCTTTGATAAT

CAAGTGTTGCTAAAGTCATTAAGCCTTCAGAAGAATCTCATAACATCTGTTGAAAAGAACGTTTTTGGGC
CAGCATTCAAGAACCTGAGTAATTTAGATATGCGCTTTAATCCATTTGATTGTACATGTGAAAGCATTGC
CTGGTTTGTTAATTGGATTAATAATACCCATACGAACATCTCTGAACTATCAAGCCGTTACCTCTGCAAC
ACTCCACCTCAATATCGTGGTTTCCCAGTGATGCTTTTTGATATATCACCCTGCAAAGACAGTGCCCCAT
TTGAACTCCTTTTCATGATAAATACCAGTATCCTATTGATTTTCATCTTTATTGTACTTCTCATCCATTT
TGAAGGCTGGAAGATATCTTTTTATTGGAATGTTACAGTGCATCGAGTTCTTGGTTTCAAAGAAATAGAC
AGACAGCCAGAACAGTTTGAATATGCGGCATATATAATTCATGCCTATAAAGATAGGGATTGGGTCTGGG
AACACTTCTCCCCGATGGAAGAAGAAGATCATACACTCAGATTCTGTCTGGAAGAAAGGGATTTTGAGGC
AGGTGTCCTTGAACTTGAAGCAATTGTTAATAGCATCAGAAGGAGCAGAAAAATTATTTTTGTTATAACA
CAGAATCTACTGAAAGATCCATTATGCAAAAGATTCAAGGTGCACCACGCAGTTCAGCAAGCTATTGAAC
AAAATCTGGATTCCATTATATTGATCTTTCTTGAGGATATTCCGGATTATAAACTGAATCATGCGCTCTG
TTTGCGAAGAGGGATGTTTAGATCTCGCTGCATCTTGAACTGGCCGATTCAGAAAGAACGGGTAAATGCC
TTTCATCATAAATTGCAAGTAGCACTTGGTTCCAGAAATTCAGTACATTAATTGATAAAGCCACAATGTT
CCAGTTACGGCAAAGCCTTAGATTTAAGCTTGAACAGTATTTTCTTTATTGGGATAAAGCAATTTGAAGG
TTTTAGGAACATTTCCTGTTTAAATCTGTCTTCAAATGGCAACGGTCAAGCGTTAAATGGAACTGAATTT
TCACTTTTGCGTGGTATCAAGTATTTGGATTTGACAAACAATAGACTAGACTTTGATGACGATGCCGCTT
TCAGCGAATTGCCATTGTTAGAAGTTCTAGATCTGAGCCACAATGCGCACTACTTCCGAATAGCAGGGGT
AACACACCGTCTAGGATTTATTCAAAATTTAACTCAGCTGAAAGTTTTAAACTTGAGCTACAATAGTATT
TTTACTTTAACAGAACCATACCTGAAAAGCATGTCCCTGGAAGAATTAGTTTTCAGTGGAAACCGCCTTG
ACCTTTTGTGGAATGCTCAAGATGTCAGGTACTGGCAAATTTTTAAATATCTCAGCAATCTGACACGGCT
TCATTTAGCCTCTAATAACCTTCAGCATATCCCCAATGAAGCCTTCCTTAACTTGCCCCGGAGTCTCACT
GAACTATATATAAATGATAATAGGTTAAATTTCTTTAACTGGTCATTACTACAGCAGTTTCCTCATCTCT
GCTTGCTTGACTTAAGTGGAAACGAGCTGTCCTTTTTAACTGATAGCCTATCGAAATTCACATCTTCTCT
TGAGACACTGCTACTGGGTCAAAACAAGATTTCCCACCTGCCGTCCGGCTTTCTTTCCGAAGCCAGCAGT
CTGACACACCTCGATTTAAGTGCCAACCAGCTCAAGACGATCAACAAATCCACGTTTGCAACTAAGACCG
CCACCAAGTTAGCCATTTTGGAACTAGGTAGAAACCCTTTTGACTGTACCTGTGACATTGGAGATTTTCG
AGAATGGATGGATGAAAATCTGAACGTCACAATTCCCAGATTGACAGATGTCATTTGCGCCAGTCCTGGG
GATCAACAAGGCAAGAGCATTGTGAGTCTAGAGCTCACAACTTGTGTTTCAGATACCATTGCGGCAGTAT
TCTGTTTCTTCACCTTTTTTGTCACCATCTCAGTTATGCTGGCTGCCCTGGCTCATCATTGGTTTTATTG
GGATGCTTGGTTTCTCTATCATGTGTGCTTAGCTAAGGTAAAAGGCTACAGGTCTCTTTCCACATCCCAG
ACTTTCTACGATGCTTACGTTTCTTATGACATCAAAGATGCCTCTGTCACAGACTGGGTGATTAATGAGC
TGCGCTTCCACCTGGAAGAGAGTGAGGACAAAAATGTGCTCCTCTGTTTAGAGGAAAGGGATTGGGACCC
GGGACTCGCCATCATCGACAACCTCATGCAGAGCATCAACCAAAGCAAGAAAACAATATTTGTTCTAACC
AAAAAGTATGCCAAAAACTGGAACTTTAAAACAGCATTCTACTTGGCCTTGCAGAGGCTAATAGATGAGA
ATATGGATGTGATTGTCTTTATTCTGCTGGAGCCAGTGTTGCAGCACTCGCAGTATTTGAGGCTGCGGCA
GAGGATCTGCAAGAGCTCCATCCTTCAGTGGCCTGACAACCCCAAGGCGGAAGGCTTGTTTTGGCAGAGT
CTGAAAAATGTCGTCTTAACTGCGAATGATTCACGGTATAACAATTTGTATGTCAATTCCGCAGGCTGAC
AGGGCCCTGTATCTGATGTGCGCCGTCCCAGCCTTCTGCCGGCTGGTGGGGTTGGTGCTGGGCCACCTGC
ACCGCAACAGGCCAGGGCCCCACGACGCAGAGCTGTGGCCTCCGAGGACCCTGTGTGAACTCTACTCATG
GTACTTCAGAATGGCCCTCAGCAGGGAGGGGCAGGAGAAGTGCAAGGCGAGCCCCCGTATTGAGCAGCTG
GCCCACGGCTGCCGCAAGATGGTGGGGACACTGGGCCGGCTGGCCTTCCACGGGCTGGTCAGGAAGAAGT
ATGTGTTCTACGAACCAGACCTCAAGGTGTTCGGCGTGGACCTTGCTCTGCTGCAGACCGCCCCGTGCAG
CTGCTTCCTGCAGCGGGAGGAGACCCTGGCCTCCTCGGCAGCCTACTACTTCACCCACCTGTCCCTGCAG
GAGTTTGTGGCAGCTGCCTACTACTACAGCGCGTCCAAGAGGGCCATCTTCGACCTCTTCACCGAGGGCG
GCATGTCCTGGCCCCGGCTTGGCTTCCTCACGCACTTCAGGAGTGCGGCCCAGAGGGCCATGCAGGCCGA
GGATGGGCGGCTCGACGTCTTCCTGCGCTTCCTCTCAGGCCTCTTGTCTCCGAGGGTCAACGCGCTGCTG
GCCGGCTCCCTGCTGACCCAGGGTGAGCACCAGGGCTACCGGGCCCAGGTGGCCGAGCTCCTGCAGGGCT
GCCTGCGCCCCAACATGGTGGTCTGCGCCCGGGCCATCAACGTCCTGCACTGCCTGCGCGAGCTGCAGCA
CACGGAGCTGGCCCACAGCGTGGAGGAGGCCATGGCAAGCGGGGGCCTGGCTGGGCTGACCAGCCCCCCA
CACCGCGCTGCCCTGGCCTACCTCCTGCAGGTGTCCGATGTCTGCGCCCAGGAGGCCAACCTGCCCCTGT
ACCTCAGCCAGGGGGTCCTCCAGAGCCTGCTGCCCCAGCTGCTCTACTGCCGGAGTCTGAGGCTGGACAC
CAACCAGTTCCAGGACCCCATGATGGAGCTGCTGGGCAGTGTGCTGAGTGGGAAGGACTGCCGCATTCAG
AGGATCAGCTTGGCTGAGAACCAGATCAGTAACAAAGGGGCGAAAGCTCTGGCCAGATCCCTCCTCGTCA
ACAGAAGTCTGACCACTCTGGACCTCCACAGTAACTCCATCGGACCTCAAGGGGCCAAGGCACTGGCAGA
TGCTCTGAAGATCAACCGCACTCTGGCCTCTCTGAGCCTCCAGAGCAACAGGATCAGGGATGACGGTGCC
AGGTCCATGGCTGAGGCCTTGGCTGCCAACCGGACCCTCTCCGTGCTGCACCTGCAGAAGAACAGCATCG
GGCCAGTGGGAACCCAGCAGATGGCAGATGCCCTGAAGCAGAACAGGAGTCTGAAGGAGCTCATGTTCTC
CAGTAACAGCATTGGCGATGGAGGTGCCAAGGCCCTGGCGGAGGCCCTGAAGGTGAACCAGGGCCTGAAG
AGCCTGGACCTGCAAAGCAATTCCATCAGTGACCTGGGAGTGGCAGCGCTGATGGAAGCCCTCTGCACCA
ACCAGACGCTCCTCAGCCTCAACCTTCGAGAAAACTCCATCAGCCCGGAGGGAGCCCAGGATCTGGCTCG
CGCTCTCCGCACCAACAGCACCCTGAAGAGCCTGGACCTGACAGCCAACCTGCTCCATGACCAGGGCGCC

CAGGCCATCGCAGCAGTTTCTGGACGGAGACGGGTGGACCGAGCGCTGGATCGAATCCAAACACAAACCG
GATTTTGGTAAATTCGTTCTCAGTTCCGGCAAGTTCTATGGTGACCAGGAGAAAGATAAAGGGCTGCAGA
CAAGCCAGGATGCCCGGTTTTACGCACTGTCTGCCAGATTCGAGCCCTTCAGCAATAAGGGTCATACACT
GGTGGTGCAGTTCACCGTGAAACACGAACAGAACATCGACTGTGGGGGCGGCTACGTGAAGCTATTTCCA
GATGGTCTGGACCAGACAGACATGCATGGAGACTCTGAATACAACATCATGTTTGGCCCGGACATCTGTG
GCCCTGGCACCAAGAAGGTTCATGTCATCTTCAACTACAAGGGCAAGAACGTGCTAATCAACAAGGATAT
CCGTTGCAAGGACGATGAATTCACACACCTGTACACGCTGATTGTGCGGCCTGATAATACCTATGAGGTA
AAGATTGACAACAGCCAGGTGGAGTCAGGCTCCTTGGAGGACGATTGGGATTTCTTGCCTCCCAAGAAGA
TAAAGGATCCTGATGCTGTAAAGCCTGAAGACTGGGACGAGCGAGCCAAGGTTGATGACCCTACAGACTC
CAAGCCTGAGGACTGGGACAAGCCTGAGCACATTCCTGACCCCGATGCTAAGAAGCCCGAGGACTGGGAT
GAAGAGATGGACGGAGAGTGGGAACCACCAGTGATTCAGAACCCAGAGTACAAGGGTGAGTGGAAGCCCC
GGCAGATTGACAACCCAGATTACAAGGGCATTTGGATCCACCCAGAGATCGACAACCCTGAGTATTCCCC
TGATAGCAACATCTACGCCTATGAAAACTTTGCTGTACTGGGCTTGGATCTATGGCAGGTCAAGTCTGGT
ACCATCTTTGACAACTTCCTCATCACCAGCGACGAAGCATATGCTGAGGAGTTTGGCAACGAGACGTGGG
GTGTTACGAAGGCAGCAGAAAAACAAATGAAGGACAAGCAAGACGAAGAGCAGAGACTAAAGGAGGAGGA
GGAGGAGAAGAAGCGCAAGGAAGAGGAGGAGGCAGACAAGGAAGATGAGGAGGACAAGGAGGAGGATGAG
GAGGATGAGGATGAGAAAGAGGAAGAGGAGGAGGAAGATGCTGCCACTGGCCAGGCCAAGGATGAGGGCA
GCCGGCAGAAGGTGCTGGTGATGGAGTACTGCGCCAGCGGGAGCCTGATGAGCGTGCTGGAGGGCCCTGA
GAACGCCTTTGGGCTGCCCGAGGACGAGTTCCTGGTGGTGCTGCGCTGTGTGGTGGCCGGCATGAACCAC
CTGCGGGAGAACGGCATCGTCCACCGAGACATCAAGCCGGGGAACATCATGCGCCTGGTCGGGGAGGAGG
GGCAGAGCATCTACAAGCTGACGGACTTCGGGGCAGCCCGGGAGCTGGATGACGACGAGAAGTTCGTCTC
GGTCTACGGCACTGAGGAGTACCTGCATCCTGCTATGTATGAGCGGGCGGTGCTTCGCAAGCCCCAACAG
AAAGCTTTTGGGGTGGCCGTGGATCTCTGGAGCATTGGGGTGACCCTGTACCATGCGGCCACTGGCAGCC
TGCCCTTCGTCCCCTTTGGAGGGCCCCGGCGCAACAAGGAGATCATGTACCGGATCACCACGGAGAAGCC
AGCCGGGGCCATTGCAGGCACTCAGAGGCTGGAGAACGGGCCCCTGGAGTGGAGCTACACCCTCCCCATC
ACCTGCCAGCTGTCCATGGGGCTGCAGAGCCAGCTGGTGCCCATCCTGGCCAACATCCTG

>Orcinus orca tlr3-tlr8-nlrc3-calr-ikbke
CCTTTAAAAGCCTGAAGAATTTAATCAAATTAGACCTAGCTCATAATGGTTTATCATCTACTAAATTAGG
AACTCAGCTGCAACTGGAAAACCTCCAAGAGCTTCTACTATCAAATAATAAAATTTCTGCACTAAGACGT
GAAGAACTTGATTTCCTTGGCAACTCTTCTTTAAAAAGATTAGAGTTGTCATCAAATCAAATAAAAGAGT
TCTCTCCAGGGTGTTTTCATGCAATTGGAAAATTATTTGGCTTCTCTCTGAACAATGCCAAACTGAGCCC
CAGTCTCATAGAAGAGCTCTGCTTGGAATTATCAAACACAAGCATTCAGAATCTGTCCCTGAGCAACAAC
CAGCTGTACACAACCAGCAACACGACTTTTGCTGGGCTGAAGCAGACAAATCTCACAGTGCTCGACCTTT
CCCATAACAGCCTAAGTGTGATTGGCAATGATTCCTTTGCGTGGCTTCCACATCTAGAATACCTCTTTCT
GGAGTATAATAATATACAACATCTGTCTTCTCGCTCTTTTTATGGGCTTTCCAACGTGAGATACCTGAAT
TTGAGACGATCTTTTACTAAACAAAGCATTTCCCTGGCTTCACTTCCCAAGATTGATGATTTTTCCTTTC
AGTGGCTAAAAGTTTTGGAGTATCTCAATATGGAAGATAACACCTTTCCAGGCATAAAAAGCAATACTTT
CACGGGATTGACAAGGCTGAGATGTTTAAGTCTATCCAACTCCTTCTCAAGTTTGCGAACTTTAACAAAT
GAAACGTTTTCATCACTTGCTGGTTCTCCTCTGCTCATACTCAACCTAACCAAAAATAAAATCTCAAAAA
TAGAAAGTGGTGCTTTTTCTTGGTTGGGCCACCTAAATGTACTTGACCTTGGCCTTAATGAAATTGGGCA
GGAACTCACAGGCCAGGAATGGAGAGGTCTAGAAAATATTGTTGAAATCTACCTTTCCTACAACAAATAC
CTAGAGCTGACTAGCAACTCTTTTGCCTTGGTTCCAAGCCTTCAACGACTGATGCTCCGAAGGGTGGCCC
TTAAAAATGTGAATAGCTCCCCTTCACCTTTTCACCCTCTTCCTAACCTGACCATTCTGGATCTAAGCAA
CAACAACATAGCCAACATAAATGATGAACTGTTGAAGGGTCTTGAGAAACTAGAAATTCTGGATTTGCAG
CATAACAACTTAGCTCGCCTCTGGAAGCATGCAAACCCTGGCGGTCCTATTCATTTTCTGAAGGGTCTTT
CTCACCTCCACATCCTTAACTTAGAGTCTAATGGCTTTGATGAGGTCCCAGCAGAAGTCTTCAAGGACTT
ACGTGAATTGAAGAGCATCGATTTAGGATTGAATAATTTAAATATCCTTCCGCCATCTGTCTTTGATAAT
CAAGTGTCGCTAAAGTCATTAAGCCTTCAGAAGAATCTCATAACATCTGTTGAAAAGAACGTTTTTGGGC
CAGCATTCAAGAACCTGAGTAATTTAGATATGAGCTTTAATCCATTTGATTGTACATGTGAAAGCATTGC
CTGGTTTGTTACTTGGATTAATAATACCCATACCAACATCTCTGAACTATCAAGCCGTTACCTCTGCAAC
ACTCCACCTCAATATCATGGTTTCCCAGTGATGCTTTTTGATATATCACCCTGCAAAGACAGTGCCCCAT
TTGAACTCCTTTTCATGATAAATACCAGTATCCTATTGATTTTTATCCTTATTGTACTCCTCATCCATTT
TGAAGGCTGGAAGATATCTTTTTATTGGAATGTTTCAGTGCATCGAGTTCTTGGTTTCAAAGAAATAGAC
AGACAGCCAGAACAGTTTGAATATGCAGCATATATAATTCATGCCTATAAGGATAGGGATTGGGTCTGGG
AACACTTTTCCCCAATGGAAGAAGAAGATCATACACTCAGATTTTGTCTGGAAGAAAGGGATTTTGACGC
AGGTGTCCTTGAACTTGAAGCAATTGTTAATAGCATCAGAAGGAGCAGAAAAATTATTTTTGTTATAACA
CAGAATCTACTGAAAGATCCATTATGCAAAAGATTCAAGGTGCACCACGCAGTTCAGCAAGCTATTGAAC
AAAATCTGGATTCCATTATATTGATCTTTCTTGAGGATATTCCGGATTATAAACTGAATCATGCGCTCTG
TTTGCGAAGAGGGATGTTTAAATCTCACTGCATCTTGAACTGGCCGGTTCAGAAAGAACGGGTAAATGCC
TTTCATCATAAATTGCAAGTAGCACTTGGTTCCAGAAATTCAGTACATTAATTGATAAAGCCACAATGTT

CCAATTACGGCAAAGCCTTAGATTTAAGCTTGAACAGTATTTTCTTTATTGGGATAAAGCAATTTGAAGG
TTTTAGGAACATTTCCTGTTTAAATCTGTCTTCAAATGGCAACGGTCAAGCGTTAAATGGAACTGAGTTT
TCACTTTTGCGTGGTATCAAGTATTTGGACTTGACAAACAACAGACTAGACTTTGACGATGACGCTGCTT
TCAGCGAATTGCCATTGTTAGAAGTTCTAGATCTGAGCTACAATGCGCACTACTTCCGAATAGCAGGGGT
AACACACCGTCTAGGATTTATTCAAAATTTAACTCAGCTGAAAGTTTTAAACTTGAGCTACAATAGTATT
TTTACTTTAACAGAACCATACCTGAAAAGCATGTCCCTGGAAGAATTAGTTTTCAGTGGAAACCGCCTTG
ACCTTTTGTGGAATGCTCAAGATGTCAGGTACTGGCAAATTTTTAAATATCTCAGCAATCTGACACGGCT
TCATTTAGCCTCTAATAACCTTCAGCGTATCCCCGATGAAGCCTTCCTTAACTTGCCCCGGAGTCTCACC
GAACTATATATAAATGATAATAGGTTAAATTTCTTTAACTGGTCATTACTGCAGCAGTTTCCTCATCTCT
GCTTGCTTGACTTAAGTGGGAACGAGCTGTTCTTTGTAACTGACAGCCTATCGAAATTCACATCTTCTCT
TGAGACACTGCTCCTGGGTCAAAACAGGATTTCCCACCTGCCGTCCGGCTTTCTTTCCGAAGCCAGCAGT
CTGACACACCTCGATTTAAGTGCCAACCAGCTCAAGATGATCAACAAATCCACGTTTGAAACTAAGACCG
CCATCAAGTTAGCCATTTTGGAACTAGGTAGAAACCCTTTTGACTGTACCTGTGACATCGGAGATTTTCG
AGAATGGATGGATGGGAATCTGAACGTCACAATTCCCAGATTGACAGATGTCATTTGCGCCAGTCCTGGG
GATCAACAAGGCAAGAGTATTGTGAGTCTAGAGCTCACAACTTGTGTTTCAGATACCATTGCGGCAATAT
TCTGTTTCTTCACCTTTTTTGTCACCATCTCAGTTATGCTGGCTGCCCTGGCTCATCATTGGTTTTACTG
GGATGCTTGGTTTCTCTATCATGTGTGCTTAGCTAAGGTAAAAGGCTACAGGTCTCTTTCCACATCCCAG
ACTTTCTACGATGCTTATGTTTCTTATGACACCAAAGATGCCTCTGTCACGGACTGGGTGATCAATGAGC
TGCGCTTCCACCTGGAAGAGAGTGAGGACAAAAATGTGCTCCTCTGTTTAGAGGAAAGGGATTGGGACCC
GGGACTCGCCATCATCGACAACCTCATGCAGAGCATCAACCAAAGCAGGAAAACAATATTTGTTCTAACC
AAAAAGTATGCCAAAAACTGGAACTTTAAAACAGCTTTCTACTTGGCCTTGCAGAGGCTAATCGATGAGA
ATATGGATGTGATTGTCTTTATTCTGCTGGAGCCAGTGTTGCAGCATTCGCAGTATTTGAGGCTGCGGCA
GAGGATCTGCAAGAGCTCCATCCTCCAGTGGCCCGACAACCCCAAGGCGGAAGGCTTGTTTTGGCAGAGT
CTGAAAAATGTCGTCTTAACTGCAAATGATTCACGGTATAACAATTTGTATGTCGATTCCGCAGGCTGAC
AGGCCCCTGTATCTGATGTGCGCCGTCCCAGCCTTCTGCCAGCTGGTGGGGTCGGTGCTGGGCCACCTGC
ACCGCAACAGGCCAGGGCTCCAGGATGCAGAGCCGTGGCCTCCAAGGACCCTGTGTGAACTCTACTCATG
GTACTTCAGAATGGCCCTCAACAGGGAGGGGCAGGAGAAGGGCAAGGTGAGCCGCCGCATTGAGCAGCTG
GCCCACGGCTGCCGCAAGATGGTGGGGACACTGGGCCGGCTGGCCTTCCACGGGCTGGTCAGGAAGAAGT
ACGTGTTCTACGAGCCAGACCTCAAGGTGTTCGGCGTGGACCTTGCTCTGCTGCAGACCGCCCCATGCAG
CTGCTTCCTGCAGCGGGAGGAGACCCTGGCCTCCTCAGCAGTCTACTACTTCACCCACCTGTCCCTGCAG
GAGTTTGTGGCAGCTGCCTACTACTACAGTGCGTCCAAGAGGGCCATCTTCGATCTCTTCACCGAGGGTG
GCATGTCCTGGCCCCAGCTTGGCTTCCTCACGCACTTCAGGAGTGCGGCCCAGAGGGCCATGCAGGCCGA
GGATGGGCGGCTCGATGTCTTCCTGCGCTTCCTCTCAGGCCTCTTGTCTCCGAGGGTCAACGCGCTGCTG
GCCGGCTCCCTGCTGACCCAGGGCGAGCACCAGGGCTACCGGGCCCAGGTGGCCGAGCTCCTGAAGGGCT
GCCTGCGCCCCAACACGGTGGTCTGCGCCCGGGCCATCAACGTCCTGCACTGCCTGCGCGAGCTGCAGCA
CACGGAGCTGGCCCACAGCGTGGAAGAGGCCATGGCAAGCGGGGGCCTGGCTGGGCTGACCAGCCCCCAA
CACCGCGCTGCCCTGGCCTACATCCTGCAGGTGTCCGATGTCTGCACCCAGGAGGCCAACCTGCCCCTGT
ACCTCAGCCAGGGGGTCCTCCAGAGCCTGCTGCCCCAGCTACTCTACTGCCGGAGTCTGAGGCTGGACAC
CAACCAGTTCCAGGACCCCATGATGGAACTGCTGGGCAGTGTGCTGAGTGGGAAGGACTGTCGCATTCAG
AGGATCAGCTTGGCTGAGAACCAGATCAGTAACAAAGGGGCGAAAGCTCTGGCCAGATCCCTCCTCGTCA
ACAGAAGTTTGACCACTCTGGACCTCCGCAGTAACTCCATTGGACCTCAAGGGGCCAAGGCACTGGCAGA
TGCTCTGAAGATCAACCGCACTCTGGCCTCTCTGTGCCTCCAGAGCAACAGGATCAGGGATGACGGTGCC
AGGTCCATGGCTGAGGCCTTGGCTGCCAACCGGACCCTCTCCGTGCTGCACCTGCAGAAGAACAGCATCG
GGCCAGCGGGAACCCAGCAGATGGCAGATGCCCTGAAGCAGAACAGGAGTCTGAAGGAGCTCATGTTCTC
CAGTAACAGCATTGGCGATGGAGGTGCCAAGGCCCTGGCGGAGACCCTGAAGGTGAACCAGGGCCTGAAG
AGCCTGGACCTGCAAAGCAATTCCATCAGTGATCTGGGAGTGGCAGCGCTGATGGAAGCCCTCTGCACCA
ACCAGATGCTCCTCAGCCTCAACCTGCGAGAAAACTCCATCAGCCCGGAGGGAGCCCAGGATCTGGCTCG
CACTCTCCGCACCAACAGCACCCTGAAGAGCCTGGACCTGACAGCCAACCTGCTCCACGACCAGGGCGCC
CAGGCCATCGCAGCAGTTTCTGGACGGAGACGGATGGACCGAGCGCTGGCTCGAATCCAAACACAAACCA
GATTTTGGCAAATTCGTTCTCAGTTCCGGCAAGTTCTACGGTGACCAGGAGAAAGATAAAGGGCTGCAGA
CAAGCCAGGATGCCCGGTTTTATGCTCTGTCGGCCAGATTCGAGCCCTTCTCCAATAAGGGTCAAACACT
GGTGGTGCAGTTCACCGTGAAACACGAACAGAACATCGACTGTGGGGGCGGCTACGTGAAGCTATTTCCA
GATGGTCTGGACCAGACAGACATGCATGGAGACTCTGAATACAACATCATGTTTGGCCCGGACATCTGTG
GTCCTGGCACCAAGAAGGTTCATGTCATCTTCAACTACAAGGGCAAGAACGTGCTGATCAACAAGGATAT
CCGTTGCAAGGACGATGAATTCACACACCTGTACACGCTGATTGTGCGGCCTGATAATACCTATGAGGTA
AAGATTGACAACAGCCAGGTGGAGTCAGGCTCTCTGGAGGACGATTGGGATTTCTTGCCTCCCAAGAAGA
TAAAGGATCCTGATGCTGCAAAGCCTGAGGACTGGGACGAGCGAGCCAAGGTTGATGACCCTACGGACTC
CAAGCCTGAGGACTGGGACAAGCCTGAGCACATTCCTGACCCAGATGCTAAGAAGCCCGAGGACTGGGAT
GAAGAGATGGACGGAGAGTGGGAACCACCAGTGATTCAGAACCCAGAGTACAAGGGTGAGTGGAAGCCCC
GACAGATTGACAACCCAGATTACAAGGGCATTTGGATCCACCCAGAGATCGACAACCCTGAGTATTCCCC
TGATAGCAACATCTACGCCTATGAAAACTTTGCTGTACTGGGCTTGGATCTATGGCAGGTCAAGTCTGGC

ACCATCTTTGACAACTTCCTCATCACCAACGACGAAGCATATGCTGAGGAGTTTGGCAACGAGACATGGG
GTGTTACGAAGGCAGCAGAAAAACAAATGAAGGACAAGCAAGACGAAGAGCAGAGGCTAAAGGAGGAGGA
GGAGGAGAAGAAGCGCAAGGAAGAGGAGGAGGCAGACAAGGAAGAGGAGGAGGACAAGGATGAGGATGAG
GAGGATGAGGACGAGAAAGAGGAAGAGGACGAGGAAGACGCTGCCGCTGGCCAGGCCAAGGATGAGGGCA
GCCGGCAGAAGGTGCTGGTGATGGAGTACTGCTCCAGCGGCAGCCTGCTGAGTGTGCTGGAGAGCCCTGA
GAACGCCTTTGGGCTGTCCGAGGACGAGTTCCTGGTGGTGCTGCGCTGTGTGGTGGCCGGCATGAACCAC
CTGCGGGAGAACGGCATCGTCCACCGAGACATCAAGCCCGGGAACATCATGCGCCTGGTCGGGGAGGAGG
GGCAGAGCATCTACAAGCTGACGGACTTTGGGGCAGCCCGGGAGCTGGATGACGACGAGAAGTTCGTCTC
GGTCTACGGCACCGAGGAGTACCTGCACCCTGACATGTATGAGCGGGCGGTGCTTCACAAGCCCCAACAG
AAAGCTTTTGGGGTCGCCGTGGACCTCTGGAGCATTGGGGTGACCCTGTACCATGCGGCCACTGGCAGCC
TGCCCTTCGTCCCCTTTGGAGGGCCCCGACGCAACAAGGAGATCATGTACCGGATCACCACGGAGAAGCC
AGCCGGGGCCATTGCAGGCACTCAGAGGCTGGAGAACGGGCCCCTGGAGTGGAGCTACACACTCCCCATC
ACCTGTCAGCTGTCCATGGGGCTGCAGAGCCAGCTGGTGCCCATCCTGGCCAACATCCTG

>Tursiops truncatus tlr3-tlr8-nlrc3-calr-ikbke
CCTTTAAAAGCCTGAAGAATTTAATCAAATTAGACCTAGCTCATAATGGTTTATCATCTACTAAATTAGG
AACTCAGCTGCAACTGGAAAACCTCCAAGAGCTTCTACTATCAAATAATAAAATTTCTGCACTAAGACGT
GAAGAACTTGATTTCCTTGGCAACTCTTCTTTAAAAAGATTAGAGTTGTCATCAAATCAAATAAAAGAGT
TCTCTCCAGGGTGTTTTCATGCAATTGGAAAATTATTTGGCTTCTCTCTGAACAATGCCAAACTGAGCCC
CAGTCTCATAGAAGAGCTCTGCTTGGAATTATCAAACACAAGCATTCAGAATCTGTCCCTGAGCAACAAC
CAGCTGTACACAACCAGCAACACGACTTTTGCTGGGCTGAAGCAGACAAATCTCACAATGCTCGACCTTT
CCCATAACAGCCTAAGTGTGATTGGCAATGATTCCTTTGCGTGGCTTCCACATCTAGAATACCTCTTTCT
GGAGTATAATAATATACAACATCTGTCTTCTCGCTCTTTTTATGGGCTTTCCAACGTGAGATACCTGAAC
TTGAGACGATCTTTTACTAAACAAAGCATTTCCCTGGCTTCACTTCCCAAGATTGATGATTTTTCCTTTC
AGTGGCTAAAATTTTTGGAGTATCTCAATATGGAAGATAACACCTTTCCAGGCATAAAAAGCAATACTTT
CACGGGATTGACAAGGCTGAGATGTTTAAGTCTATCCAACTCCTTCTCAAGTTTGGGAACTTTAACAAAT
GAAACGTTTTTATCACTTGCTGGTTCTCCTCTGCTCATACTCAACCTAACCAAAAATAAAATCTCAAAAA
TAGAGAGTGGTGCTTTTTCTTGGTTGGGCCACCTAAATGTACTTGACCTTGGCCTTAATGAAATTGGGCA
GGAACTCACAGGCCAGGAATGGAGAGGTCTAGAAAATATTGTTGAAATCTACCTTTCCTACAACAAATAC
CTAGAGCTGACTAGCAACTCTTTTGCCTTGGTTCCAAGCCTTCAACGACTGATGCTCCGAAGGGTGGCCC
TTAAAAATGTGAATAGCTCCCCTTCACCTTTTCACCCTCTTCCTAACCTGACCATTCTGGATCTAAGCAA
CAACAACATAGCCAACATAAATGATGAACTGTTGAAGGGTCTTGAGAAACTAGAAATTCTGGATTTGCAG
CATAACAACTTAGCTCGCCTCTGGAAGCATGCAAACCCTGGCGGTCCTATTCATTTTCTGAAGGGTCTTT
CTCACCTCCACATCCTTAACTTAGAGTCTAATGGCTTTGATGAGGTCCCAGCAGAAGTCTTCAAGGACTT
ACGTGAATTGAAGAGCATCGATTTAGGATGGAATAATTTAAATATCCTTCCGCCATCTGTCTTTGATAAT
CAAGTGTCGCTAAAGTCATTAAGCCTTCAGAAGAATCTCATAACATCTGTTGAAAAGAACGTTTTTGGGC
CAGCATTCAAGAACCTGAGTAATTTAGATATGAGCTTTAATCCATTCGATTGTACATGTGAAAGCATTGC
CTGGTTTGTTACTTGGATTAATAATACCCATACCAACATCTCTGAACTATCAAGCCGTTACCTCTGCAAC
ACTCCACCTCAATATCATGGTTTCCCAGTGATGCTTTTTGATATATCACCCTGCAAAGACAGTGCCCCAT
TTGAACTCCTTTTCATGATAAATACCAGTATCCTATTGATTTTTATCCTTATTGTACTCCTCATCCATTT
TGAAGGCTGGAAGATATCTTTTTATTGGAATGTTTCAGTGCATCGAGTTCTTGGTTTCAAAGAAATAGAC
AGACAGCCAGAGCAGTTTGAATATGCGGCATATATAATTCATGCCTATAAGGATAGGGATTGGGTCTGGG
AACACTTCTCCCCAATGGAAGAAGAAGATCATACACTCAGATTTTGTCTGGAAGAAAGGGATTTTGACGC
AGGTGTCCTTGAACTTGAAGCAATTGTTAATAGCATCAGAAGGAGCAGAAAAATTATTTTTGTTATAACA
CAGAATCTACTGAAAGATCCATTATGCAAAAGATTCAAGGTGCACCACGCAGTCCAGCAAGCTATTGAAC
AAAATCTGGATTCCATTATATTGATCTTTCTTGAGGATATTCCGGATTATAAACTGAATCATGCGCTCTG
TTTGCGAAGAGGGATGTTTAAATCTCACTGCATCTTGAACTGGCCGGTTCAGAAAGAACGGGTAAATGCC
TTTCATCATAAATTGCAAGTAGCACTTGGTTCCAGAAATTCAGTACATTAATTGATAAAGCCACAATGTT
CCAATTACGGCAAAGCCTTAGATTTAAGCTTGAACAGTATTTTCTTTATTGGGATAAAGCAATTTGAAGG
TTTTAGGAACATTTCCTGTTTAAATCTGTCTTCAAATGGCAACGGTCAAGCGTTAAATGGAACTGAGTTT
TCACTTTTGCGTGGTATCAAGTATTTGGACTTGACAAACAACAGACTAGACTTTGACGATGACGCTGCTT
TCAGCGAATTGCCATTGTTAGAAGTTCTAGATCTGAGCTACAATGCGCACTACTTCCGAATAGCAGGGGT
AACACACCGTCTAGGATTTATTCAAAATTTAACTCAGCTGAAAGTTTTAAACTTGAGCTACAATAGTATT
TTTACTTTAACACATCTGAAAAGCATGTCCCTGGAAGAATTAGTTTTCAGTGGAAACCGCCTTGACCTTT
TGTGGAATGCTCAAGATGTCAGGTACTGGCAAATTTTTAAATATCTCAGCAATCTGACACGGCTTCGTTT
AGCCTCTAATAACCTTCAGCGTATCCCCGATGAAGCCTTCCTTAACTTGCCCCGGAGTCTCACCGAACTA
TATATAAATGATAATAGGTTAAATTTCTTTAACTGGTCATTACTACAGCAGTTTCCTCATCTCTGCTTGC
TTGACTTAAGTGGGAACGAGCTGTTCTTTGTAACTGACAGCCTATCGAAATTCACATCTTCTCTTGAGAC
ACTGCTCCTGGGTCAAAACAGGATTTCCCACCTGCCGTCCGGCTTTCTTTCCGAAGCCAGCAGTCTGACA
CACCTCGATTTAAGTGCCAACCAGCTCAAGATGATCAACAAATCCACGTTTGAAACTAAGACCGCCACCA
AGTTAGCCATTTTGGAACTAGGTAGAAACCCTTTTGACTGTACCTGTGACATCGGAGATTTTCGAGAATG

GATGGATGGGAATCTGAACGTCACAATTCCCAGATTGACAGATGTCATTTGCGCCAGTCCTGGGGATCAA
CAAGGCAAGAGTATTGTGAGTCTAGAGCTCACAACTTGTGTTTCAGATACCATTGCGGCAATATTCTGTT
TCTTCACCTTTTTTGTCACCATCTCAGTTATGCTGGCTGCCCTGGCTCATCATTGGTTTTACTGGGATGC
TTGGTTTCTCTATCATGTGTGCTTAGCTAAGGTAAAAGGCTACAGGTCTCTTTCCACATCCCAGACTTTC
TACGATGCTTACGTTTCTTATGACACCAAAGATGCCTCTGTCACGGACTGGGTGATCAATGAGCTGCGCT
TCCACCTGGAAGAGAGTGAGGACAAAAATGTGCTCCTCTGTTTAGAGGAAAGGGATTGGGACCCGGGACT
CGCCATCATCGACAACCTCATGCAGAGCATCAACCAAAGCAGGAAAACAATATTTGTTCTAACCAAAAAG
TATGCCAAAAACTGGAACTTTAAAACAGCTTTCTACTTGGCCTTGCAGAGGCTAATCGATGAGAATATGG
ATGTGATTGTCTTTATTCTGCTGGAGCCAGTGTTGCAGCATTCGCAGTATTTGAGGCTGCGGCAGAGGAT
CTGCAAGAGCTCCATCCTCCAGTGGCCCGACAACCCCAAGGCGGAAGGCTTGTTTTGGCAGAGTCTGAAA
AATGTCGTCTTAACTGCAAATGATTCACGGTATAACAATTTGTATGTCGATTCCGCAGGCTGACAGACCC
CTGTATCTGATGTGCGCCGTCCCAGCCTTCTGCCAGCTGGTGGGGTCGGTGCTGGGCCACCTGCACCGCA
ACAGGCCAGGGCTCCAGGATGCAGAGCCGTGGCCTCCAAGGACCCTGTGTGAACTCTATTCATGGTACTT
CAGAATGGCCCTCAGCAGGGAGGGGCAGGAGAAGGGCAAGGTGAGCCCCCGCATTGAGCAGCTGGCCCAC
GGCTGCCGCAAGATGGTGGGGACACTGGGCCGGCTGGCCTTCCACGGGCTGGTCAGGAAGAAGTACGTGT
TCTACGAGCCAGACCTCAAGGTGTTCGGCGTGGACCTTGCTCTGCTGCAGACCGCCCCATGCAGCTGCTT
CCTGCAGCGGGAGGAGACCCTGGCCTCCTCAGCAGCCTACTACTTCACCCACCTGTCCCTGCAGGAGTTT
GTGGCAGCTGCCTACTACTACAGTGCATCCAAGAGGGCCATCTTCGATCTCTTCACCGAGGGTGGCATGT
CCTGGCCCCAGCTTGGCTTCCTCACGCACTTCAGGAGTGCGGCCCAGAGGGCCATGCAGGCCGAGGATGG
GCGGCTCGACGTCTTCCTGCGCTTCCTCTCAGGCCTCTTGTCTCCGAGGGTCAACGCGCTGCTGGCCGGC
TCCCTGCTGACCCAGGGCGAGCACCAGGGCTACCGGGCCCAGGTGGCCGAGCTCCTGAAGAGCTGCCTGC
GCCCCAACACGGTGGTCTGCGCCCGGGCCATCAACATCCTGCACTGCCTGCGCGAGTTGCAGCACACGGA
GCTGGCCCACAGCGTGGAAGAGGCCATGGCAAGCGGGGGCCTGGCCGGGCTGACCAGCCCCCAACACCGC
GCTGCCCTGGCCTACCTCCTGCAGGTGTCCGATGTCTGCACCCAGGAGGCCAACCTGCCCCTGTACCTCA
GCCAGGGGGTCCTCCAGAGCCTGCTGCCCCAGCTGCTCTACTGCCGGAGTCTGAGGCTGGACACCAACCA
GTTCCAGGACCCCATGATGGAACTGCTGGGCAGTGTGCTGAGTGGGAAGGACTGTCGCATTCAGAGGATC
AGCTTGGCTGAGAACCAGATCAGTAACAAAGGGGCGAAAGCTCTGGCCAGATCCCTCCTCGTCAACAGAA
GTTTGACCACTCTGGACCTCCGCAGTAACTCCATTGGACCTCTAGGGGCCAAGGCACTGGCAGATGCTCT
GAAGATCAACCGCACTCTGGCCTCTCTGTGCCTCCAGAGCAACAAGATCAGGGATGACGGTGCCAGGTCC
ATGGCTGAGGCCTTGGCTGCCAACCGGACCCTCTCCGTGCTACACCTGCAGAAGAACAGCATCGGGCCAG
CGGGAACCCAGCAGATGGCAGATGCCCTGAAGCAGAACAGGAGTCTGAAGGAGCTCATGTTCTCCAGTAA
CAGCATTGGCGATGGAGGTGCCAAGGCCCTGGCGGAGACCCTGAAGGTGAACCAGGGCCTGAAGAGCCTG
GACCTGCAAAGCAATTCCATCAGTGACCTGGGAGTGGCAGCGCTGATGGAAGCCCTCTGCACCAACCAGA
TGCTCCTCAGCCTCAACCTGCGAGAAAACTCCATCAGCCCGGAGGGAGCCCAGGATCTGGCTCGCGCTCT
CCGCACCAACAGCACCCTGAAGAGCCTGGACCTGACAGCCAACCTGCTCCACGACCAGGGCGCCCAGGCC
ATCGCAGCAGTTTCTGGACGGAGACGGATGGACCGAGCGCTGGATCGAATCCAAACACAAACCAGATTTT
GGCAAATTCGTTCTCAGTTCCGGCAAGTTCTACGGTGACCAGGAGAAAGATAAAGGGCTGCAGACAAGCC
AGGATGCCCGGTTTTATGCTCTGTCGGCCAGATTCGAGCCCTTCTCCAATAAGGGTCAAACACTGGTGGT
ACAGTTCACCGTGAAACACGAACAGAATATCGACTGTGGGGGCGGCTACGTGAAGCTATTTCCAGATGGT
CTGGACCAGACAGACATGCATGGAGACTCTGAATACAACATCATGTTTGGCCCGGACATCTGTGGTCCTG
GCACCAAGAAGGTTCATGTCATCTTCAACTACAAGGGCAAGAACGTGCTGATCAACAAGGATATCCGTTG
CAAGGACGATGAATTCACACACCTGTACACACTGATTGTGCGGCCTGATAATACCTATGAGGTAAAGATT
GACAACAGCCAGGTGGAGTCAGGCTCCCTGGAGGACGATTGGGATTTCTTGCCTCCCAAGAAGATAAAGG
ATCCTGATGCTGCAAAGCCTGAAGACTGGGACGAGCGAGCCAAGGTTGATGACCCTACGGACTCCAAGCC
TGAGGACTGGGACAAGCCTGAGCACATTCCTGACCCGGATGCTAAGAAGCCCGAGGACTGGGATGAAGAG
ATGGACGGAGAGTGGGAACCACCAGTGATTCAGAACCCAGAGTACAAGGGTGAGTGGAAGCCCCGACAGA
TTGACAACCCAGATTACAAGGGCATTTGGATCCACCCAGAGATCGACAACCCTGAGTATTCCCCTGATAG
CAACATCTACGCCTATGAAAACTTTGCTGTACTGGGCTTGGATCTATGGCAGGTCAAGTCTGGCACCATC
TTTGACAACTTCCTCATCACCAACGACGAAGCATACGCTGAGGAGTTCGGCAACGAGACATGGGGTGTTA
CGAAGGCAGCAGAAAAACAAATGAAGGACAAGCAAGACGAAGAGCAGAGGCTAAAGGAGGAGGAGGAGGA
GAAGAAGCGCAAGGAAGAGGAGGAGGCAGACAAGGAAGAGGAGGAGGACAAGGATGAGGATGAGGAGGAT
GAGGACGAGAAAGAGGAAGAGGACGAGGAAGACGCTGCCGCTGGCCAGGCCAAGGATGAGGGCAGCCGGC
AGAAGGTGCTGGTGATGGAGTACTGCTCCAGCGGCAGCCTGCTGAGTGTGCTGGAGAGCCCTGAGAACGC
CTTTGGGCTGCCCGAGGACGAGTTCCTGGTGGTGCTGCGCTGTGTGGTGGCCGGCATGAACCACCTGCGG
GAGAACGGCATCGTCCACCGAGACATCAAGCCCGGGAACATCATGCGCCTGGTCGGGGAGGAGGGGCAGA
GCATCTACAAGCTGACGGACTTTGGGGCAGCCCGGGAGCTGGATGACGACGAGAAGTTCGTCTCGGTCTA
CGGCACCGAGGAGTACCTGCACCCTGACATGTATGAGCGGGCGGTGCTTCACAAGCCCCAACAGAAAGCT
TTTGGGGTCGCCGTGGATCTCTGGAGCATTGGGGTGACCCTGTACCATGCGGCCACTGGCAGCCTGCCCT
TCGTCCCCTTTGGAGGGCCCCGACGCAACAAGGAGATCATGTACGGTGGGCCACCGGGCAGCGGGCGAAG
TGGCGGACCCCGGGCCTTCCCTTCTAGTCCCTGCTGTGTCCCCTATTCTG

>Physeter catodon tlr3-tlr8-nlrc3-calr-ikbke
CCTTTAAAAACCTGAAGAATTTAATCAAATTAGACCTAGCTCATAATGGTTTATCATCTACTAAATTAGG
AACTCAGCTGCAACTGGAAAATCTCCAAGAGCTTCTACTATCAAATAATAAAATTTCTGCACTGAGACGT
GAAGAACTTGATTTCCTTGGCAACTCTTCTTTAAAAAGATTAGAGTTGTCATCAAATCAAATAAAAGAGT
TCTCTCCAGGGTGTTTTCATGCAATTGGAAAATTATTTGGCTTCTCTCTGAACAATGCCAAACTGAGCCC
CAGTCTCATAGAAGAGCTCTGCTTGGAATTATCGAACACAAGCCTTCAGAATCTGTCCCTGAGCAACAAC
CAGCTGTACACAACCAGCAACACAACTTTTGTTGGGCTAAAGCAGACAAATCTCACAATGCTCGATCTTT
CCCATAACAGCTTAAGTGTGATTGGTAATGATTCCTTTGCTTGGCTTCCACATCTAGAATATCTCTTTCT
GGAGTATAATAACATAGAACATCTGTCTTCTCGCTCTTTTTATGGGCTTTCCAATGTGAGATACCTGAAT
CTGAGACGATCTTTTACTAAACAAAGCGTTTCCCTGGCTTCACTTCCCAAGATTGATGATTTTTCCTTTC
AGTGGCTAAAATTTTTGGAGTATCTCAATATGGAAGATAACACCTTTCCAGGCCTAAAAAGCAATACTTT
CACGGGATTGACAAGGCTGAGGTGTTTAAGTCTATCCAACTCCTTCTCAAGTTTGCGAACTTTAACAAAT
GAAACGTTTTTATCACTTGCTTGTTCTCCTCTGCTCATACTGAACCTGACCAAAAATAAAATCTCAAAAA
TCGAGAGTGGTGCTTTTTCTTGGCTGGGCCACCTAAATGTACTTGACCTTGGCCTTAATGAAATTGGGCA
AGAACTCACAGGCCAGGAATGGAGAGGTCTAGAAAATATTGTTGAAATCTACCTTTCCTACAACAAATAC
CTAGAGCTGACTAGCAACTCTTTTGCCTTGGTTCCCAGCCTTCAACGACTGATGCTCCGTAGGGTGGCCC
TTAAACATGTGGATAGCTCCCCCTCACCTTTTCACCCTCTTCCTAACCTGACCATTCTGGATCTAAGCAA
CAACAACATAGCCAACATCAATGATGAACTGTTGAAGGGTCTGGAGAAACTAGAAATTCTGGATTTGCAG
CATAACAACTTAGCTCGCCTCTGGAAGCATGCAAACCCTGGTGGTCCTGTTCATTTTCTGAAGGGTCTTT
CTCACCTCTACATCCTTAACTTAGAGTCTAATGGCTTTGATGAGATCCCAGCAGAAGTCTTCAAGGACTT
ACGTGAATTGAAGAGCATCGATTTAGGATTGAATAATTTAAATATCCTTCCACCATCTCTCTTTGATAAT
CAAGTGTTGCTCAAGTCATTAAGCCTTCAGAAGAATCTCATAACGTCTGTTGAAAAGAACGTTTTTGGGC
CAGCCTTCAAGAACCTGAGTGATTTAGATATGGGCTTTAATCCATTTGATTGTACCTGTGAAAGCATTGC
CTGGTTTGTTAATTGGATTAATAACACCCATACCAACATCTCTGAACTATCAAGCCGTTACCTCTGCAAC
ACTCCACCTCAATATCGTGGTTTCCCAGTGATGCTTTTTGATACATCACCCTGCAAAGACAGTGCCCCAT
TTGAACTCCTTTTCATGATAAATACCAGTATCCTACTGATTTTTAACCTTATTGTACTGCTCATCCATTT
TGAAGGCTGGAAGATATCTTTTTATTGGAATGTTTTAGTGCATCGAGTTCTTGGTTTCAAAGAAATAGAC
AGACATCCAGAACAGTTTGAATATGCGGCATATATCATTCATGCCTATAAAGATAGGGATTGGGTCTGGG
AACACTTCTCCCCAATGGAAGAAGAAGATCATACACTCAGGTTTTGTCTGGAAGAAAGGGATTTTAAGGC
AGGTGTCCTTGAACTTGAAGCAATTGTTAATAGCATCAGAAGGAGCAGAAAAATTATTTTTGTTATAACA
CAGAATCTACTGAAAGATCCATTATGCAAAAGATTCAAGGTGCACCACGCAGTTCAGCAAGCTATTGAAC
AAAATCTGGATTCCATTATATTGATCTTTCTTGAGGATATTCCGGATTATAAACTGAATCATGCGCTCTG
TTTGCGAAGAGGGATGTTTAAATCTCACTGCATCTTGAACTGGCCGGTTCAGAAAGAACGGGTAAATGCC
TTTCATCATAAATTGCAAGTAGCACTTGGTTCCAGAAATTCAGTACATTAATTGATAAAGCCACAATGCT
CCAACTACGGCAAAGCCTTAGATTTAAGCTTGAACAGTATTTTCTTTATTGGGATAAAGCAATTTGAAGG
TTTTAGGAACATTTCCTGTTTAAATCTGTCTTCAAATGGCAACGGTCAAGCGTTAAACGGAACTGAATTT
TCACTTTTGCGTGGTATCAAGTATTTGGATTTGACAAACAACAGACTAGACTTTGATGATGATGCAGCTT
TCAGCGAATTGCCACTGTTAGAAGTTCTGGATCTGAGCTACAATGCTCACTACTTCCGCATAGCAGGGGT
AACACACCGTCTAGGATTTATTCAAAATTTAACTCAGCTGAAAGTTTTAAACTTGAGCTACAACAGTATT
TTTACTTTAACAGAACCATACCTGAAAAGCATGTCCCTGGAAGAATTAGTTTTCAGTGGAAACCGCCTTG
ACCTTTTGTGGAATGCTCAAGATGTCAGGTACTGGCAAATTTTTAAATATCTCAGCAATCTGACACGGCT
TCATTTAGCCTCTAATAACCTTCAGCGTATCCCCGATGAAGCCTTCCTTAACTTGCCCCGGAGTCTCACC
GAACTATATATAAATGATAATAGGTTAAATTTCTTTAACTGGTCATTACTACCGCAGTTTCCTCATCTCT
GCTTGCTTGACTTAAGTGGAAACGAGCTGTTCTTTGTAACTGATAGCCAATCGAAAGTCACACCTTCTCT
TGAGACGCTGCTCCTGGGTCAAAACAGGATTTCCCACCTGCCGTCTGGCTTTCTTTCCGAAGCCAGCAGT
CTGACACACCTCGATCTAAGTGCCAACCAGCTCAAGACGATCAACAAATCCACGTTTGAAACTAACACCG
CCACCAAGTTAGCCATTTTGGAACTAGGTAGAAACCCTTTTGACTGTACCTGTGACATTGGAGATTTTCG
AGAATGGATGGATGGCAATCTGAACGTCACAATTCCCAGGTTGACAGATGTCATTTGCAGCAGTCCTGGG
GATCAACAAGGCAAGAGCATTGTGAGTCTAGAGCTCACAACCTGTGTTTCAGATACCATTGCGGCAATAT
TCTGTTTCTTCACCTTTTTTGTCACCGTCTCAGTTATGCTGGCTGCCCTGGCTCATCATTGGTTTTACTG
GGATGCTTGGTTTCTCTATCATGTGTGCTTAGCTAAGGTAAAAGGCTACAGGTCTCTTTCCACATCCCAG
ACTTTCTACGATGCTTACGTTTCTTATGACACCAAAGACGCCTCTGTCACGGACTGGGTGATCAATGAGC
TGCGCTTCCACCTGGAAGAGAGTGAGGACAAAAATGTGCTCCTCTGTTTAGAGGAAAGGGATTGGGACCC
GGGACTCGCCATCATCGACAACCTCATGCAGAGCATCAACCAAAGCAAGAAAACAATATTTGTTCTAACC
AAAAAGTATGCCAAAAACTGGAACTTTAAAACAGCATTCTACTTGGCCTTGCAGAGGCTAATAGATGAGA
ATATGGATGTGATCGTCTTTATTCTGCTGGAGCCAGTGTTGCAGCACTCGCAGTATCTGAGGCTGCGGCA
AAGGATCTGCAAGAGCTCCATCCTCCAGTGGCCTGACAACCCCCAGGCGGAAGGCTTGTTTTGGCAGAGT
CTGAAAAATGTCGTCTTAACTGCAAATGATTCACGGTATAACAACTTGTATGTCAATTCCGCAGGCTGAC
AGGCCCCTGTATCTGATGTGCACCGTCCCAGCCTTCTGCCGGCTGGTGGGGTCGGTGCTGGGCCACCTGC
ACCGCAACAGGCCAGGGCCCCAGGACGCAGAGCTGTGGCCTCCGAGGACCCTGTGTGAACTCTACTCATG
GTACTTCAGAATGGCCCTCAGCAGGGAGGGGCAGGAGAAGGGCAAGGCGAGCCCCTGCATTGAGCAACTG

GCCCACGGCTGCCGCAAGATGGTGGGGACACTGGGCCGGCTGGCCTTCCATGGGCTGGTCAGGAAGAAGT
ACGTGTTCTACGAGCCAGACCTCAAAGTGTTCGGTGTGGACCTTGCTCTGCTGCAGACCGCCCTGTGCAG
CTGCTTCCTGCAGCGGGAGGAGACCCTGGCCTCCTCGGCAGCCTACTACTTCACCCACCTGTCCCTGCAG
GAGTTTCTGGCAGCTGCCTACTACTACAGCACATCCAAGAGGGCCATCTTCGACCTCTTCACCGAGGGTG
GCATGTCCTGGCCCCGGCTTGGCTTCCTCACGCACTTCAGGAGTGCGGCCCAGAGGGCCATGCAGGCCGA
GGATGGGCGGCTCGACGTCTTCCTGCGCTTCCTCTCAGGCCTCTTGTCTCCGAGGGTCAATGCGCTGCTG
GCCGGCTCCCTGCTGACCCAGGGCGAGCACCAGGGCTACCGGGCCCAGGTGGCCGAGCTCCTGAAGGGCT
GCCTGCGCCCCAACATGGTGGTCTGCGCCCGGGCCATCAATGTCCTGCACTGCCTGCGCGAGATGCAGCG
CACGGAGCTGGCCCACAGCGTGGAGGAGGCCATGGCAAGCGGGGGCCTGGCTGGGCTGACCAGCCCCCCA
CACCGCGCTGCCCTGGCCTACCTCCTGCAGGTGTCCGATGTCTGCACCCAGGAGGCCAACCTGTCCCTGT
ACCTCAGCCAGGGGGTCCTCCAGAGCCTGCTGCCCCAGCTGCTCTACTGCCAGAGTCTGAGGCTGGACAC
CAACCAGTTCCAGGACCCCATGATGGAACTGCTGGGCAGTGTGCTGAGCGGGAAGGACTGTCGCATTCAG
AGGATCAGCTTGGCTGAGAACCAGATCAGTAACAAAGGGGCAAAAGCTCTGGCCAGATCCCTCCTCGTCA
ACAGAAGTCTGACCACTCTGGACCTCCGTAGTAACTCCATTGGACCTCAAGGGGCCAAGGCACTGGCAGA
TGCTCTGAAGATCAACCGCACTCTGGCCTCTCTGAGCCTCCAGAGCAACAGGATCAGGGATGACGGTGCC
AGGTTCATGGCTGAGGCCTTGGCTGCCAACCGGACCCTCTCTGTGCTGCACCTGCAGAATAACAGCATTG
GGCCAGTGGGAACCCAGCAGATGGCAGAGGCCCTGAAGCAGAACAGGAGCCTGAAGGAGCTCATGTTCTC
CAGTAACAGCATTGGCGATGGAGGTGCCAAGGCCCTGGCGGAGGCCCTGAAGGTGAACCAGGGCCTGAAG
AGCCTGGAACTGCAAAGCAATTCCATCAGTGACCTGGGAGTGGCAGCGCTGATGGAAGCCCTCTTCACCA
ACCAGATGCTCCTCAGCCTCAACCTGCGAGAAAACTCCATCAGCCCGGAGGGAGCCCAGGATCTGGCTCG
CGCTCTCCGCACCAACAGCACCCTGAAGAGCCTGGACTTGACAGCCAACCTGCTCCACGACCAGGGCGCC
CAGGCCATCGCAGCAGTTTCTGGACGGAGACGGGTGGACCGAGCGCTGGATCGAATCCAAACACAAACCG
GATTTTGGCAAATTCGTTCTCAGTTCCGGCAAGTTCTACGGTGACCAGGAGAAGGATAAAGGGCTGCAGA
CAAGCCAGGATGCCCGGTTTTACGCTCTGTCGGCCAGATTCGAGCCCTTCTCCAATAAGGGTCATACACT
GGTGGTGCAGTTCACCGTGAAACACGAACAGAACATCGACTGTGGGGGCGGCTACGTGAAGCTATTTCCA
GATGGTCTGGACCAGACAGACATGCATGGAGACTCTGAATACAACATCATGTTTGGCCCGGACATCTGTG
GCCCTGGCACCAAGAAGGTTCATGTCATCTTCAACTACAAGGGCAAGAACGTGCTGATCAACAAGGACAT
CCGTTGCAAGGATGATGAATTCACACACCTGTACACGCTGATTGTGCGGCCTGATAATACCTACGAGGTA
AAGATTGACAACAGCCAGGTGGAGTCAGGCTCCTTGGAGGACGATTGGGATTTCTTGCCTCCCAAGAAGA
TAAAGGATCCTGATGCTGCAAAGCCTGAAGACTGGGACGAGCGAGCCAAGGTTGATGACCCTACGGACTC
CAAGCCTGAGGACTGGGACAAGCCTGAGCACATTCCTGACCCCGATGCTAAGAAGCCCGAGGACTGGGAT
GAAGAAATGGACGGAGAGTGGGAACCACCAGTGATTCAGAACCCAGAGTACAAGGGTGAGTGGAAGCCCC
GGCAGATTGACAACCCAGATTACAAGGGCATTTGGATCCACCCAGAAATCGACAACCCTGAGTATTCCCC
TGATAGCAACATCTACGCCTATGAAAACTTTGCTGTACTGGGCTTGGATCTATGGCAGGTCAAGTCTGGC
ACCATCTTTGACAACTTCCTCATCACCGACGATGAAGCATATGCTGAGGAGTTTGGCAACGAGACATGGG
GTGTTACGAAGGCAGCAGAAAAACAAATGAAGGACAAGCAAGACGAAGAGCAGAGGCTAAAGGAGGAGGA
GGAGGAGAAGAAGCGCAAGGAAGAGGAGGAGGCAGACAAGGAAGAGGAGGAGGACAAGGATGAGGATGAG
GAGGAGGAGGAGGAGAAAGAGGAAGAGGAGGAGGAAGATGCTGCCGCTGGCCAGGCCAAGGATGAGGGCA
GCCGGCAGAAGGTGCTGGTGATGGAGTACTGCTCCAGCGGGAGCCTGCTGAGTGTGCTGGAGAGCCCTGA
GAACGCCTTCGGTCTGCCTGAGGACGAGTTCCTGGTGGTGCTGCGCTGTGTGGTGGCCGGCATGAACCAC
CTGCGGGAGAACGGCATCGTCCACCGAGACATCAAGCCGGGGAACATCATGCGCCTGGTCGGGGAGGAGG
GGCAGAGCATCTACAAGCTGACGGACTTCGGGGCAGCCCGGGAGCTGGATGACGACGAGAAGTTCGTCTC
GGTCTACGGCACCGAGGAGTACCTGCACCCTGACATGTATGAGCGGGCGGTGCTTCGCAAGCCCCAACAG
AAAGCTTTTGGGGTGGCCGTGGATCTCTGGAGCATTGGGGTGACCCTGTACCATGCGGCCACTGGCAGCC
TGCCCTTCGTCCCCTTTGGAGGACCCCGGCGCAACAAGGAGATCATGTACCGGATCACCACGGAGAAGCC
AGCCGGGGCCATTGCAGGCACTCAGAGGCTGGAGAACGGGCCCCTGGAGTGGAGCTACACCCTCCCCGTC
ACCTGCCAGCTGTCCATGGGACTGCAGAGCCAGCTCGTGCCCATCCTTGGCAACATCCTG

>Equus caballus tlr3-tlr8-nlrc3-calr-ikbke
CCTTTAAAAATCTGAAGAATTTAATCAAATTAGATCTATCTCATAACGGTTTATCATCTACTAAATTAGG
AACTCAGCTCCAACTGGAAAATCTCCAAGAGCTTCTGTTATCAAATAATAAAATTCAGGCACTGAGACGT
GACGAACTTGATTTCCTTGGCAATTCTTCTTTGAAAAAGTTAGAGTTGTCCTCAAATCAAATTAAAGAGT
TTTCTCCAGGGTGTTTTCAGGCAATTGGAAAATTATTTGGCCTCTCTCTGAACAGTGTCCAACTGGGCCC
CAGTCTCACAGAGAAACTTTGTCTGGAATTATCAAACACCAGCATCCAGAATCTATCTTTGAGTAACACA
CAGCTGTACAGAACGAGCAATACAACTTTCTTTGGGCTAAAGCAGACAAATCTCACCATGCTCGATCTTT
CCCACAACAACTTAACTGTGATTGGTAATGATTCCTTTGCTTGGCTTCCCCATCTGGAATATTTCTTCCT
GGAGTATAATAATATAGAGCATTTGTATGTTCACTCCTTTTATGGGCTTTTCAATGTGAGATACCTGAAT
TTGAGACGATCTTTTACTAAGCAGAGCATTTCTCTTGCTTCGCTTCCCAAGATTGATGATTTTTCCTTTC
AGTGGCTAAAATGTTTGGAGTATCTTAACATGGAAGATAATAATTTTCCAGGCATAAAAAGCAATATGTT
CACAGGATTGGTAAAGCTGAAACACTTAAGTCTGTCCAACTCCTTTTCAAGTTTGCGGACTCTAACAAAT
GAAACATTTCTATCACTCGCTCATTCTCCTCTACTCACACTCAACCTAACCAAAAACAAAATCTCAAAAC

TAGAAAGTGGTGCTTTTTCTTGGTTGGGCCACCTAAAGATACTGGACCTTGGCCTTAATGAAATTGGGCA
AGAACTCACAGGTCAGGAATGGAGAGGTCTAGAAAATATTTTTGAAATCTACCTGTCCTACAACAAATAC
CTACAATTGACTAGCAACTCTTTTGCCTTGGTTCCAAGCCTTCAAAGACTGATGCTCCGAAGGGTGGCCC
TTAAAAATGTGGACAGCTTCCCTCCACCTTTTCGCCCTCTTCATAACTTGACCATCCTGGATCTAAGCAA
CAACAACATAGCCAACATAAATGATGAACTGTTGGAGGGTCTTGAGAAACTAGAAGTTCTGGATTTGCAG
CATAACAATTTAGCACGGCTCTGGAAACACGCAAACCCTGGTGGTCCTGTTCATTTTCTAAAGGGTCTTT
CTCACCTCCACGTCCTTAACTTAGAGTCTAACGGCTTTGATGAGATCCCAGCAGAGGCCTTCAAGAATTT
ATTTGAATTAAAAAGCATTAATCTAGGACTGAATAATTTAAACATACTTCCGCCATCTGTCTTTGACGAT
CAGGTGTCTCTGAAGTCACTGAGCCTTCAGAAGAATCTCATCACATCAGTTGAGAAGAATGTTTTTGGGC
CAGCTTTGAAGAACCTGAGTAGTTTAGATATGAGCTTTAATCCATTTGATTGTACATGTGAAAGCATTGC
CTGGTTTGTTAATTGGATTAATGGTACCCATACCAACATCTCTGAGCTATCAAGTCATTATCTCTGCAAC
ACTCCACCTCAGTATCATGGTTTCCCAGTGATGCTTTTTGATACATCATCCTGCAAAGACAGTGCCCCCT
TTGAACTTCTTTTCATGATAAATACCAGTTTCCTATTGATTTTTATCTTTATTGTACTGCTCATCCATTT
TGAAGGCTGGAGAATATCTTTTTACTGGAACGTTTCAGTGCATCGAGTTCTTGGTTTCAAAGAAATAGAC
AGCCAGCCAGAGCAGTTTGAGTATGCAGCATATATAATTCACGCCTACAAAGATAGGGACTGGGTCTGGG
AACACTTCTCCCCAATGGAAGAACAAGATCAAACTCTCAAGTTTTGTCTGGAAGAAAGGGACTTTCAGGC
AGGTGTCCTTGAACTTGAAGCAATTATTAACAGCATCAAAAGGAGCAGAAAAATTATTTTCATTATAACA
CAGCATCTATTAAAAGATCCATTATGCAAAAGATTCAAGGTGCATCATGCAGTTCAGCAAGCTATTGAAC
AGAACCTGGATTCCATTATATTGATCTTTCTTGAGGAGATTCCAGATTATAAACTGAACCATGCGCTCTG
TTTGCGAAGAGGAATGTTTAAATCGCGCTGTATCTTGAACTGGCCAGTCCAGAAAGAACGGATAAATGCC
TTTCATCATAAACTGCAAGTAGCACTTGGATCCAGAAATTCAGCACATTAATTAATAAAGCCACAGTGTA
CAGCTTATGGCAAAGCCTTAGATTTAAGCTTGAATAGTATTTTCTTTATTGGGCAAAAGCAGTTTAAAGC
TTTTCATGACATTGCCTGCTTAAATCTGTCTTCCAATGGCATTGGTCAACCGTTGCATGGAACTGAATTT
TCAGCTGTACCGCATATCAAATATTTGGATTTGACAAACAATAGAATAGACTTTGATGATGATAATGCTC
TCAGAGAATTGCCTGAGTTAGAAGTTCTAGATTTCAGCTACAATGCACACTATTTCCGAATAGCAGGGGT
AACGCATCGTCTAGGATTTATTCAAAATTTAACACAGCTAAGAGTTTTGAACTTGAGCCACAACAGCATT
TACACTTTAACAGAGTACAATATGAATAGTATGTCTCTGGAAGAATTAGTTTTCAGTGGAAACCGCCTTG
ACCTTTTGTGGAATGCTGAAGACAGAAGGTACAGGAAAATTTTTAAATGTCTCAGGAATCTGACACGGCT
TGATTTATCCTTTAATAACCTCCAGCATATCCCAGATGAAGCATTCCTTAACTTGCCCCAGAATCTCACC
GAACTGTATATAAATGACAATAGATTACATTTCTTTAACTGGACATTACTCCAACATTTTCCTCATCTCC
ACTTGCTTGACCTAAGTAGAAACAAACTCTCCTCTTTAACTAATAACCTATCTAAATTCTCACCTTCTCT
TCGGACACTGCTACTGAGTCAAAACAAGATTTCCCACCTGCCTTCTGGCTTCTTCTCCGAAGCTAGCGGT
CTGATACACCTCGATTTACGTTTCAACAGGCTAAAGATGATCAACAAAACCACGCTTCAAACTAAGACCA
CCATCAATTTAGCCGTTTTGGAACTTGGTGGAAACCCTTTTGACTGTACCTGTGACATTGGAGATTTTCG
GAGATGGATGGATGAAAATCTGAATATCGCAATTCCTAGATTGGCAGATGTCATCTGTGCCAGTCCTGGG
GATCAAAGGGGGAAGAGTATTGTGAGTTTAGAGCTAACGACGTGTGTTTCAGATGCCATTGCCGCAATAT
TATGCTTCTTCACGTTCTTCATCACTGTCACAGTTATGTTGGCTGCCCTAGCTCACCATTGGTTTTACTG
GGATGTTTGGTTTATCTATCACATGTGTTTAGCTAAGATAAAAGGCTACAGGTCTCTTTCCACATCCCAA
ACTTTCTACGATGCTTACGTTTCTTATGACACCAAAGACGCCTCTGTTACGGACTGGGTGATAAATGAGC
TGCGCTTCCACCTAGAAGAGAGTGAAGAAAAAAACGTGCTCCTCTGTTTAGAGGAGAGGGATTGGGACCC
GGGATTAGCCATCATCGATAACCTCATGCAGAGCATAAACCAAAGCAAGAAGACAATATTTGTTTTAACC
AAAAAATATGCAAAGAACTGGAACTTTAAAACAGCATTCTACTTGGCCTTGCAGAGGCTAATGGATGAGA
ATATGGATGTGATTGTATTTATTCTGCTGGAGCCAGTGTTACAGCATTCCCAGTATTTGCGGCTGCGGCA
GAGGATCTGCAAGAGCTCCATCCTCCAGTGGCCTGACAACCCCAAGGCAGAAGGCTTGTTTTGGCAAAGT
CTGAAAAATGTGGTCTTAACTGAAAATAATTCACGGTATAACAATTTGTATGTTGATTCCGCAGGCCGAC
AGGGCCCTCTACCTGATGTGTACCATCCCAGCCTTCTGCCGGCTCACAGGTTTGGCGCTGGGCCACTTAT
GCCGCAACAAGCCAGAGCCCCAGGACACAGAACTGTGGCCTCCAAGGACCCTGTGTGAGCTCTACTCTTG
GTACTTCAGGATGGCTCTTGGTGGGGAGGGGCAGGAGAAGGGTAAGGCGAGCCCTCGCATCGAGCAGCTG
GCCCATGGCGGCCGCAAGATGGTGGGGACACTAGGCCGGCTGGCCTTCCATGGGCTGGTCAAGAAGAAGT
ACGTGTTCTATGAGCCAGACCTGAAGGCGTTTGGCGTGGACCTGGCTCTGCTGCAGAGCGCCCTGTGCAG
CTGCTTCCTGCAGCGGGAGGAGAGCTTGGCCTCCTCGGCAGCCTACTGCTTCACCCACCTGTCCCTGCAG
GAGTTCGTGGCGGCCACATACTACTATAGCGCCTCCAAGAAGGCCATCTTCGACCTCTTCACCGAGGGTG
GCGTGTCCTGGCCTCGGCTCGGCTTCCTCACGCATTTCAGAGGCGCGGCCCAGCGTGCCATGCAGGCTGA
GGACGGACGGCTGGACGTGTTCCTGCGCTTCCTCTCGGGCCTCTTGTCTCCAAGGGTCAATGCCCTGCTG
GCCGGCTCCCTGCTGGCCCAGGGTGAGCACCAGGGCTACCGGGCCCAGGTGGCCGAGCTCCTCCAGGGCT
GCCTGCGCCATGACACAGCGGTCTGCGCCCGGGCGATCAATGTCCTGCACTGCCTGCATGAGCTGCAGCA
CACGGAGCTGGCCCGTGGCGTGGAGGAGGCCCTGGAGAGCGGAGGTCTGGCCAGGCTGACTGGCCCCCAT
CACCGTACCGCCCTGGCCTACCTCTTGCAGGTGTCGGACACTTGCGCCCAGGAGGTCAACCTGTCCCTAC
ACCTCAGCCAAGGCGTCCTGCAAAGTCTGCTGCCCCAGCTGCTCTACTGCCGGAGCCTGAGGCTGGACAC
CAACCAGTTCCAGGACCCCGTGATGGAGCTGCTGGGCAGTGTGCTGAGTGGGAAGGACTGTCGCATTCAG
AGGATCAGTTTGGCTGAGAACCAGATCAGTAACAAGGGGGCCAAGGCTCTGGCCAGATCCCTCCTGGTCA

ACAGAAGTCTGACCACTCTGGACCTCCGCAGTAACTCCATCGGACCTCAAGGGGCCAAGGCGCTGGCAGA
TGCTCTGAAGATTAACCGCACTCTGGCCTCTCTGAGCCTCCAGAGCAACACGATCAGGGATGATGGTGCC
AGGTCCATAGCTGAGGCCTTGGGCACCAACCGGACCCTCTCCGTGCTGCACCTGCAGAAGAACACCATTG
GGCCCGTGGGAACCCAGCGGATGGCAGATGCCCTGAAGCAGAACAAGAGTCTGAAGGAGCTCATGTTCTC
CAGTAACAGCATGGGTGATGGAGGTGCCAAGGCCCTGGCTGAGGCCCTGAAGGTGAACCAGGGCCTGGAG
AACCTGGACCTGCAAAGCAATTCCATCAGTGACGCGGGAGTGGCAGCACTGATGGGGGCCCTCTGCACCA
ACCAGACCCTCCTCAGCCTCAACCTTCGAGAAAACTCCATCAGCCCAGAGGGAGCCCAGGATTTGGCACA
TGCTCTCTGTACCAACAGCACCCTGAAGAACCTGGACCTGACAGCCAACCTCCTTCACGACGAGGGTGCC
CAGGCCATCGCGGCAGTTTCTGGATGGAGACGGGTGGGCCGAGCGCTGGATCGAATCCAAACACAAGTCA
GATTTCGGCAAATTCATCCTCAGTTCCGGCAAGTTTTACGGTGACCAGGAGAAAGATAAAGGGCTGCAGA
CCAGCCAGGATGCCCGCTTTTACGCCCTGTCGGCCAGATTTGAGCCTTTCAGCAACAAGGGCCAGACGCT
GGTGGTGCAGTTCACAGTGAAACACGAGCAGAACATCGACTGTGGGGGCGGCTATGTGAAGCTCTTTCCA
GATAGTTTGGACCAGACCGACATGCATGGAGACTCAGAATACAACATCATGTTTGGCCCGGACATCTGTG
GTCCTGGCACCAAGAAGGTTCATGTCATCTTCAACTACAAGGGCAAGAACGTGCTGATCAACAAGGACAT
CCGTTGCAAGGACGATGAGTTCACACACCTGTACACGCTGATTGTGCGTCCGGATAACACCTATGAGGTG
AAGATTGACAACAGCCAGGTGGAGTCGGGCTCCTTGGAGGATGATTGGGACTTCCTGCCTCCCAAGAAGA
TCAAGGATCCCAATGCTGCAAAGCCCGAAGACTGGGACGAGCGGGCCAAGATCGACGACCCCACAGACTC
CAAGCCTGAGGATTGGGACAAGCCTGAGCACATCCCCGACCCCGATGCTAAGAAGCCCGAGGACTGGGAC
GAAGAGATGGACGGAGAGTGGGAACCACCGGTGATTCAGAACCCAGAGTACAAGGGTGAGTGGAAGCCCC
GGCAGATCGACAACCCCGATTACAAGGGCACGTGGATCCACCCAGAGATCGACAACCCCGAGTACTCCCC
AGATGGCAACATCTACGCCTATGAAAACTTCGCCGTGCTGGGCCTGGATCTCTGGCAGGTCAAGTCTGGC
ACCATCTTTGACAACTTCCTCATCACCAACGACGAGGCGTACGCCGAGGAGTTCGGCAACGAGACGTGGG
GTGTCACAAAGGCGGCGGAAAAGCAGATGAAGGACAAGCAGGACGAGGAGCAGAGGCTAAAGGAGGAGGA
GGAAGACAAGAAGCGCAAGGAGGAGGAGGAGGCCGACAAGGACGACGAGGACGACAAGGAGGAGGACGAG
GAGGATGAGGAGGACGAGGAGGAAGATGCCGCTGGCCAGGCCAAGGATGAGGGCAGCCGGCAGAAGGTGC
TGGTGATGGAGTACTGCTCCAGTGGGAGCCTGCTGAGCGTGCTTGAGAGCCCCGAGAACGCCTTCGGGCT
GCCGGAGGACGAGTTCCTGGTGGTGCTGCGCTGTGTGGTGGCTGGCATGAACCACCTGCGGGAGAACGGC
ATCGTCCACCGTGACATCAAGCCCGGGAACATCATGCGCCTCCTGGGGGAGGAGGGGCAGAGCATCTACA
AGCTGACGGACTTCGGGGCCGCCCGCGAGCTGGACGACGATGAGAAGTTCATCTCGGTCTATGGGACTGA
GGAGTACCTGCACCCTGACATGTATGAGCGGGCAGTGCTTCGCAAGCCCCAGCAGAAGGCGTTCGGGGTG
GCTGTGGATCTCTGGAGCATTGGGGTGACCCTGTACCACGCAGCCACCGGCAGCCTGCCCTTTGTCCCCT
TCGGTGGGCCACGGCGAAACAAAGAGATCATGTACCGGATAACCACAGAGAAGCCAGCCGGGGCCATTGC
AGGCACCCAGAGGCGGGAAAACGGGCCTTTGGAGTGGAGCTACAGCCTCCCCATCACCTGCCGGCTGTCA
TCGGGGCTGCAGAGCCAGCTGGTGCCCATCCTGGCCAACATCCTG

>Canis lupus familiaris tlr3-tlr8-nlrc3-calr-ikbke
CTTTTCGAAGCCTGAAGAATTTAGTCAAATTAGATCTATCTCATAATGGTTTGTCATCTACTAAATTAGG
AAGTCAGCTCCAATTGGAAAATCTCCAAGAGCTTCTGTTATCAAATAATAAAATTAATGTACTGAGGCGT
GAAGAACTTGATTTCCTTGGTAATTCTTCTTTAGAAAAATTGGAGTTGTCATCAAATCCAATTAAAGAGT
TCTCTCCAGGGTGTTTCCATGCAATTGGAAAATTATTTGGCCTCTCTCTGAACAACGTTCAGCTGAACCC
CAGTCTCACAGAGAACCTTTGTTTGGAACTGTCAAATACAAGCATCCAGAATCTATCCTTAAGCAACACC
CAGCTACACAGAACAAGCAATATGACGTTCCTTGGACTAAAGCATACAAATCTCACCATGCTCGATCTTT
CCCACAACAACTTGAATGTGATTGAGAACAATTCCTTTGTCTGGCTTCCACATCTAGAATATTTCTTACT
GGAGTATAATAATATAGAGCATTTGTTTTCCCACTCCTTTTATGGGCTTCTCAATGTAAGATACCTGGAT
TTGAAACGATCTTTTGCTAAACAAAGCACTTCCCTTGCTTCGCATCCCAGGATTGATGATTTTTCCTTTC
AGTGGCTAAAATGTTTGCAGTATCTGAACATGGAAGACAACTACTTCGCAGGCATAAAAAGCAATATGTT
CACAGGATTGATAAAGTTGAAACACTTGAGTCTCTCCAACTCCTTCACAAGTTTGCAAACTTTAACCAAT
GAAACATTTTTGTCACTTGCTCAGTCTCCTTTAATCACACTCAATCTAACCAAAAACAAAATCTCAAAAA
TAGAGAGTGGTGCTTTTTCTTGGCTGGGCCACCTACAGGTACTTGACCTTGGCCTTAATGAAATTGGACA
AGAACTCACAGGCCAAGAGTGGAGAGGTCTAGAAAATATTGTCGAAATCTACCTTTCCTACAACAAATAC
CTACAACTGACGAGCAGCTCTTTTGCCTTGATTCCCAGCCTCCGACGACTGATGCTCCGAAGAACGGCCC
TTAGAAATGTGGACAGCTCCCCTTCACCTTTTCATCCTCTTCGGAACTTGAACATTCTGGATCTAAGCAA
CAACAATATAGCCAACATAAATGATGAGCTGTTGGAGGGTCTTGAGAAATTAGAAATTCTGGATATGCAG
CATAACAACTTAGCAAGGCTATGGAAACATGCAAACCCCGGTGGTCCTGTTCATTTTCTAAAGGGTCTTT
CTCACCTCCACATTCTTAATTTAGAGTCTAATGGCTTTGATGAGATCCCAGCAGAGGTGTTCAAGGGCTT
ATCTGAATTAAAGAGCATTGATTTAGGATTGAATAATTTAAACATATTTCCATCATCTCTCTTTAATGAT
CAGGTGTCTCTGAAGTCATTGAACCTTCAGAAGAATCTCATAACATCAGTTGAGAAGAATGTTTTTGGGC
CAGCCTTCAGGAACCTCAGTAATTTAGATATGAGCTTTAATCCATTTGATTGTACCTGTGAAAGTATTGC
CTGGTTTGTTAATTGGATTAACAGCACCCATACCAACATCTCTGAGTTATCAAGCCATTACCTCTGCAAC
ACTCCACCTCAATATCATGGTTTCCCAGTGATGCTTTTTGATATATCACCCTGCAAAGACAGTGCCCCCT
TTGAAATCTTTTTCATAATAAATACCAGTGTCTTACTGACTTTTATCTTTATTGTGCTGCTGATCCATTT

TGAAGGCTGGAGGATATCTTTTTATTGGAATGTTTCAGTGCACCGAATTCTTGGTTTCAAAGAAATAGAC
AAACAGCCAGAGCAGTTTGAATACGCAGCTTATATAATTCATGCCTATAAAGATAGAGATTGGGTCTGGG
AACACTTCTCTCCAATGGAAGAAAAAGATGAAACTCTCAAATTTTGTCTTGAAGAGAGGGACTTTGAGGC
AGGTGTCCTTGAACTCGAGTCAATCATTAATAGCATCAAAAAGAGTAGAAAAACTATTTTTGTTATAACA
CAGCATCTATTAAAGGATCCATTATGCAAAAGATTCAAAGTGCACCAGGCAGTTCAGCAAGCTATTGAAC
AAAATCTAGAGTCCATTATATTGATCTTTCTTGAGGAGATTCCAGACTATAAACTGAACCATGCACTCTG
TTTGCGAAGAGGGATGTTTAAATCTCACTGCATCTTGAACTGGCCAGTTCAGAAAGAACGGGTAAATGCC
TTTCATCATAAATTGCAAGTAGCACTTGGATCCAGAAATTCAATACATTAATTAATAAAGCCACAATGTA
CAGTTTATGGCAAAGCCTTAGATTTAAGCCTGAACAGTATTTTCTTCATCGGGCGAGAGCAATTTGAAGC
TTTTCATGACATCGCCTGCTTAAATCTGTCTTCGAACGGCAACGGTCAAGTGTTACATGGAAATGAATTT
TCAGCGGTGCCGCATATCAAATATTTGGATTTGACAAACAACAGACTAGACTTTGACGATGACAACGCGC
TCAGTGATCTGCCCGAGTTAGAAGTTCTGGATCTCAGCTACAATGCACACTACTTCCGAATAGCAGGGGT
GACGCACCGCCTAGGATTTATTCAAAACTTAACACAACTGAAAGTCTTAAACTTGAGCCACAACAGCATC
TACACTTTAACGGAGCAAGACCTGAGAAGCGTGTCCCTGGAAGAATTAGTTTTCAGTGGAAACCGCCTTG
ACATTTTGTGGAATGCCGAAGGTGACAAGTACTGGAAAATTTTTACACGTCTCAGGAATCTGACACGGCT
CGATCTATCCTTAAACAACCTCCGGCGCATCCCGAACGAAGCTTTCCTGAACCTGCCCCAGAGTCTCACC
CAACTGTACATAAAAAATAACGCTTTAAATTTCTTTAACTGGACGTTACTCCAGGAGTTTCCACGTCTCC
AGGTGCTGGACTTGAGTGGAAACAGGCTGTCCTCTATAACCAACAGCCTCTCCAAATTCACGTCTTCCCT
GCAGACGCTGCTACTTCACCGAAACAGGATTTCCCACCTGCCCGCCAGCTTTCTTTCCGAAGCCAGCAGT
CTGATTCACCTCGACCTGAGCTCCAACCTGCTGAAGATGATCAACAAATCCACGCTGCAAACTAAGACCA
ACACCAGTTTAGCCATTCTGGAACTAGGTAGAAACCCTTTTGACTGTACTTGCGACATTGGAGATTTTCG
AAGATGGATGGATGAAAATCTGAATGTCACCATCCCTAGGTTGACAGACGTCATCTGCTCTAGTCCCGGG
GATCAAAGGGGGAAGAGCATTGTGAGTCTGGAGCTAACAACTTGTATTTCGGATACCCTCGCGGCGGTGC
TGTGTATCTTCACGTCCTTTATCACCGTCACCGTCATGTTGGCTGCCTTGGGTCACCATTGGTTTTATTG
GGATGTCTGGTTTATCTATCACGTGTGCTTGGCTAAGGTAAAAGGCTACAGGTCTGTTTCCACGTCCCAA
ACTTTCTACGACGCCTATGTTTCTTATGACACCAAAGATGCCTCTGTTACTGACTGGGTGATAAATGAGC
TGCGCTTCCACCTAGAAGAGAGTGAAGGGAAAAATGTGCTCCTCTGTTTAGAGGAGAGAGATTGGGACCC
GGGATTAGCCATCATCGATAACCTCATGCAGAGCATAAACCAAAGCAAGAAAACCATATTCGTCTTAACC
AAAGAATACGCCCAAAACTGGAACTTTAAAACGGCATTCTACTTGGCCTTGCAGCGGCTCATGGATGAGA
ATATGGATGTGATCATATTTATTTTGCTGGAGCCAGTGTTACAGCATTCTCAGTATCTGAGGCTGCGGCA
GAGGATCTGCAAGAGCTCCATCCTTCAATGGCCTGACAACCCCAAGGCGGAAGGCTTGTTTTGGCAAAGT
CTGAAAAACGTGGTCTTAACTGAAAACGATTCACGGTATAACAATTTGTACGTTGATTCCGCAGGCCGAC
AGGGCTCTGTATCTGATGTGCACAGTCCCCGCGTTCTGCCGGCTGGCCGCGTCGGCACTGGCTCACTTGA
GCCGCAGCAAGCTGGGGCCCCAGGATGCAGAGCCATGGGCCCCAAGGACCCTGTGCGAGCTCTACTCCTG
CTACTTCAGGATGGCCCTCGGTGGGGACGGGCAGGAGAAGGGCAAGGCGAGCCCTCGCATCGAGCAGGTG
GCCCACAGCAGCCGCAAGATGGTGGGGACCCTGGGGCGGCTGGCCTTTCACGGGCTGGTCAGGAAGAAGT
ACGTGTTCTATGAGCAGGACCTGAAGGCGTTCGGCGTGGATCTCACTCTGGTGCAGAGCGCCCTGTGTGG
CTGCTTCCTGCAGCGGGAGGAGACCCTGGCCTCGTCCACAGCCTACTGCTTCAGCCACCTGTCCCTGCAG
GAGTACGTGGCAGCTGCGTACTACTACAGTGCGTCCAAGAGGGCCATCTTCGACCTCTTCACCGAGGGTG
GCGTGTCCTGGCCCCGCCTGGGCTTCCTCACACACTTCAGGAGTGCGGCCCAGCGGGCCATGCAGGCTGA
GGACGGGCGGCTGGATGTGTTCCTGAGGTTCCTCTCGGGACTCCTGTCCCCAAGGGTCAATGCCCTGCTG
GCCGGCTCCCTGCTGGCCCAGGGTGAGCACCAGGGCTACCGGGCCCAGGTGGCGGAGCTCCTGCAGGGCT
GCCTACGCCCCAACGTGGCCGTCTGTGCCCGGGCAGTCAACATCCTACACTGCCTGCACGAGCTGCAGTG
CACAGAGTTGGCCCGCAGCGTGGAGGAGGCCATGGAGAGCGGGGGCCTGGCAGGGCTGACCGGCCCCCAG
CACCGCGCGGCCCTGGCCTATCTCCTGCAGGTGTCTGATGCCTGCGCCCAGGAGGCCAACCTGTCCCTGT
GCCTCAGCAGGAGCGTCCTGCAGAGCCTGCTGCCCCAGCTGCTCTACTGCCGGAGCCTCAGGCTGGACAA
CAACCAGTTTCAGGACCCCGTGATGGAGCTGCTGGGCAGTGTGCTGAGTGGGAAGGACTGTCGCATTCAG
AGGATCAGCTTGGCTGAGAACCAGATCAGTAATAAAGGTGCCAAAGCTCTGGCCAGATCCCTCCTGGTCA
ACAGAAGTCTGACTGCTCTGGACCTCCGCAGCAACTCCATTGGACCTCAAGGGGCCAAGGCTCTGGCAGA
TGCTCTGAAGATTAACCGCACTCTGGCCTTTCTGAGCCTCCAGAGCAACGAGATCAGGGATAATGGTGCC
AGGTCCATGGCTGAGGCGTTGGCTACCAACCGGACCCTCTCTGTGCTGCACCTACAGAAGAATACCGTCG
GGCCTGTGGGAGCCCAGCTGATGGCGGAGACCCTGAAACAGAACAGGAGTCTGAAGGAGCTCATATTTTC
CAGTAACAGCATCGGTGACGGAGGTGCTGAGGCCCTGGCTAAGGCCCTGAGGGTGAACCAGGGCCTGGAG
AATCTGGACCTGCAGAGCAATTCCATCAGTGACACGGGAGTGGCAGCCCTGATGGGGGCCCTCTGTGCCA
ACCAGGCCCTCACCAGCCTCAATCTTCGAGAAAACTCTATCAGCCCGGAGGGAGCCCGGGAGCTGGCACG
AGCTCTCCGCAGCAACAGCACCCTAAAGAACCTGGACCTGACAGCCAACCTCCTCCATGACCAGGGCGCC
CAGGCCATCGCGGCAGTTTCTGGACGGAGACGGGTGGACCGACCGCTGGATCGAATCCAAACACAAGTCA
GATTTTGGTAAATTTGTTCTCAGTTCCGGCAAGTTCTACAATGACCAGGAGAAGGATAAAGGGCTGCAGA
CAAGCCAGGATGCCCGCTTTTACGCTTTGTCGGCCAGATTTGAGCCCTTCAGCAACAAAGGCCAGACACT
GGTGGTGCAGTTCACCGTAAAACATGAGCAAAACATCGACTGTGGGGGCGGCTACGTGAAGCTATTTCCA
GATGGTTTGGACCAGACGGACATGCACGGAGACTCTGAGTACAACATCATGTTTGGCCCGGATATCTGTG

GCCCCGGCACCAAGAAGGTTCATGTCATCTTCAACTACAAGGGCAAGAACGTCCTGATCAACAAGGACAT
TCGTTGCAAGGATGATGAATTCACACACCTGTATACGCTGATTGTGCGGCCGGATAACACCTATGAGGTG
AAGATTGACAACAGCCAGGTGGAGTCAGGCTCCTTGGAGGATGATTGGGACTTCCTGCCTCCCAAGAAGA
TAAAGGATCCTGATGCTTCAAAGCCTGAAGACTGGGACGAGCGGGCCAAGATTGATGACCCCACAGACTC
CAAGCCTGAGGACTGGGACAAGCCTGAGCACATTCCTGACCCTGATGCTAAAAAGCCAGAGGACTGGGAT
GAAGAGATGGATGGAGAGTGGGAACCACCTGTAATTCAGAACCCTGAGTACAAGGGCGAGTGGAAGCCCC
GGCAGATTGACAACCCAGATTACAAGGGCACTTGGATCCACCCAGAGATCGACAACCCTGAGTACTCTCC
TGATAGCAACATCTATGCCTATGAAAACTTTGCTGTTCTGGGCTTAGATCTCTGGCAGGTCAAGTCTGGC
ACTATCTTTGACAACTTCCTCATCACCAACGACGAGGCGTATGCAGAGGAGTTTGGAAATGAGACCTGGG
GTGTCACGAAGGCGGCAGAGAAGCAGATGAAGGACAAGCAGGATGAGGAGCAGAGGCTAAAGGAGGAGGA
GGAGGACAAGAAGCGCAAGGAAGAAGAGGAGGCAGACAAGGAAGATGAGGAAGACAAGGACGAGGATGAG
GAGGACGAGGATGACAAGGAGGAAGAGGAAGAGGATGATGCTGCCGCTGGCCAGGCCAAGGACGAGGGCA
GCCGGCAGAAGGTGCTGGTGATGGAGTACTGCTCTAGCGGGAGCCTGCTCAGCGTGCTCGAGAGCCCTGA
GAATGCCTTCGGGCTGCCCGAGGACGAGTTTCTGGTGGTGCTGCGCTGTGTGGTGGCTGGCATGAACCAC
CTGCGGGAGAATGGCATCGTCCACCGCGACATCAAGCCTGGAAACATCATGCGCCTCATGGGGGAGGAAG
GGCAGAGCATCTATAAGCTGACGGACTTTGGGGCCGCCCGGGAGCTAGATGATGATGAGAAGTTTGTCTC
CGTCTATGGCACTGAGGAGTACCTGCACCCCGACATGTATGAGCGGGCAGTGCTTCGCAAGCCCCAGCAG
AAGACATTCGGGGTAACCGTGGATCTCTGGAGCATTGGGGTAACCCTGTACCACGCAGCCACTGGCAGCC
TGCCCTTCGTCCCCTTTGGTGGGCCACGGCGCAACAAGGAGATGATGTACCGGATCACCACCGAGAAGCC
GGCCGGGGCCATCGCGGGCACCCAGCGGCGGGAGAACGGGCCCCTGGAGTGGAGCTACGGCCTCCCCATC
ACCTGCCGGCTGTCCCCGGGGCTGCAGAGCCAGCTGGTGCCCATCCTGGCCAACATCCTG

>Homo sapiens tlr3-tlr8-nlrc3-calr-ikbke
CCTTTGTCAAGCAGAAGAATTTAATCACATTAGATCTGTCTCATAATGGCTTGTCATCTACAAAATTAGG
AACTCAGGTTCAGCTGGAAAATCTCCAAGAGCTTCTATTATCAAACAATAAAATTCAAGCGCTAAAAAGT
GAAGAACTGGATATCTTTGCCAATTCATCTTTAAAAAAATTAGAGTTGTCATCGAATCAAATTAAAGAGT
TTTCTCCAGGGTGTTTTCACGCAATTGGAAGATTATTTGGCCTCTTTCTGAACAATGTCCAGCTGGGTCC
CAGCCTTACAGAGAAGCTATGTTTGGAATTAGCAAACACAAGCATTCGGAATCTGTCTCTGAGTAACAGC
CAGCTGTCCACCACCAGCAATACAACTTTCTTGGGACTAAAGTGGACAAATCTCACTATGCTCGATCTTT
CCTACAACAACTTAAATGTGGTTGGTAACGATTCCTTTGCTTGGCTTCCACAACTAGAATATTTCTTCCT
AGAGTATAATAATATACAGCATTTGTTTTCTCACTCTTTGCACGGGCTTTTCAATGTGAGGTACCTGAAT
TTGAAACGGTCTTTTACTAAACAAAGTATTTCCCTTGCCTCACTCCCCAAGATTGATGATTTTTCTTTTC
AGTGGCTAAAATGTTTGGAGCACCTTAACATGGAAGATAATGATATTCCAGGCATAAAAAGCAATATGTT
CACAGGATTGATAAACCTGAAATACTTAAGTCTATCCAACTCCTTTACAAGTTTGCGAACTTTGACAAAT
GAAACATTTGTATCACTTGCTCATTCTCCCTTACACATACTCAACCTAACCAAGAATAAAATCTCAAAAA
TAGAGAGTGATGCTTTCTCTTGGTTGGGCCACCTAGAAGTACTTGACCTGGGCCTTAATGAAATTGGGCA
AGAACTCACAGGCCAGGAATGGAGAGGTCTAGAAAATATTTTCGAAATCTATCTTTCCTACAACAAGTAC
CTGCAGCTGACTAGGAACTCCTTTGCCTTGGTCCCAAGCCTTCAACGACTGATGCTCCGAAGGGTGGCCC
TTAAAAATGTGGATAGCTCTCCTTCACCATTCCAGCCTCTTCGTAACTTGACCATTCTGGATCTAAGCAA
CAACAACATAGCCAACATAAATGATGACATGTTGGAGGGTCTTGAGAAACTAGAAATTCTCGATTTGCAG
CATAACAACTTAGCACGGCTCTGGAAACACGCAAACCCTGGTGGTCCCATTTATTTCCTAAAGGGTCTGT
CTCACCTCCACATCCTTAACTTGGAGTCCAACGGCTTTGACGAGATCCCAGTTGAGGTCTTCAAGGATTT
ATTTGAACTAAAGATCATCGATTTAGGATTGAATAATTTAAACACACTTCCAGCATCTGTCTTTAATAAT
CAGGTGTCTCTAAAGTCATTGAACCTTCAGAAGAATCTCATAACATCCGTTGAGAAGAAGGTTTTCGGGC
CAGCTTTCAGGAACCTGACTGAGTTAGATATGCGCTTTAATCCCTTTGATTGCACGTGTGAAAGTATTGC
CTGGTTTGTTAATTGGATTAACGAGACCCATACCAACATCCCTGAGCTGTCAAGCCACTACCTTTGCAAC
ACTCCACCTCACTATCATGGGTTCCCAGTGAGACTTTTTGATACATCATCTTGCAAAGACAGTGCCCCCT
TTGAACTCTTTTTCATGATCAATACCAGTATCCTGTTGATTTTTATCTTTATTGTACTTCTCATCCACTT
TGAGGGCTGGAGGATATCTTTTTATTGGAATGTTTCAGTACATCGAGTTCTTGGTTTCAAAGAAATAGAC
AGACAGACAGAACAGTTTGAATATGCAGCATATATAATTCATGCCTATAAAGATAAGGATTGGGTCTGGG
AACATTTCTCTTCAATGGAAAAGGAAGACCAATCTCTCAAATTTTGTCTGGAAGAAAGGGACTTTGAGGC
GGGTGTTTTTGAACTAGAAGCAATTGTTAACAGCATCAAAAGAAGCAGAAAAATTATTTTTGTTATAACA
CACCATCTATTAAAAGACCCATTATGCAAAAGATTCAAGGTACATCATGCAGTTCAACAAGCTATTGAAC
AAAATCTGGATTCCATTATATTGGTTTTCCTTGAGGAGATTCCAGATTATAAACTGAACCATGCACTCTG
TTTGCGAAGAGGAATGTTTAAATCTCACTGCATCTTGAACTGGCCAGTTCAGAAAGAACGGATAGGTGCC
TTTCGTCATAAATTGCAAGTAGCACTTGGATCCAAAAACTCTGTACATTAATTAATAAAGCCACAATGTG
CTGCTTATGGAAAAGCCTTAGATTTAAGCCTCAACAGTATTTTCTTCATTGGGCCAAACCAATTTGAAAA
TCTTCCTGACATTGCCTGTTTAAATCTGTCTGCAAATAGCAATGCTCAAGTGTTAAGTGGAACTGAATTT
TCAGCCATTCCTCATGTCAAATATTTGGATTTGACAAACAATAGACTAGACTTTGATAATGCTAGTGCTC
TTACTGAATTGTCCGACTTGGAAGTTCTAGATCTCAGCTATAATTCACACTATTTCAGAATAGCAGGCGT
AACACATCATCTAGAATTTATTCAAAATTTCACAATCTAAAAGTTTTAAACTTGAGCCACAACAACATT

TATACTTTAACAGATAAGTATAACCTGGAAAGCAAGTCCCTGGTAGAATTAGTTTTCAGTGGCAATCGCC
TTGACATTTTGTGGAATGATGATGACAACAGGTATATCTCCATTTTCAAAGGTCTCAAGAATCTGACACG
TCTGGATTTATCCCTTAATAGGCTGAAGCACATCCCAAATGAAGCATTCCTTAATTTGCCAGCGAGTCTC
ACTGAACTACATATAAATGATAATATGTTAAAGTTTTTTAACTGGACATTACTCCAGCAGTTTCCTCGTC
TCGAGTTGCTTGACTTACGTGGAAACAAACTACTCTTTTTAACTGATAGCCTATCTGACTTTACATCTTC
CCTTCGGACACTGCTGCTGAGTCATAACAGGATTTCCCACCTACCCTCTGGCTTTCTTTCTGAAGTCAGT
AGTCTGAAGCACCTCGATTTAAGTTCCAATCTGCTAAAAACAATCAACAAATCCGCACTTGAAACTAAGA
CCACCACCAAATTATCTATGTTGGAACTACACGGAAACCCCTTTGAATGCACCTGTGACATTGGAGATTT
CCGAAGATGGATGGATGAACATCTGAATGTCAAAATTCCCAGACTGGTAGATGTCATTTGTGCCAGTCCT
GGGGATCAAAGAGGGAAGAGTATTGTGAGTCTGGAGCTAACAACTTGTGTTTCAGATGTCACTGCAGTGA
TATTATTTTTCTTCACGTTCTTTATCACCACCATGGTTATGTTGGCTGCCCTGGCTCACCATTTGTTTTA
CTGGGATGTTTGGTTTATATATAATGTGTGTTTAGCTAAGGTAAAAGGCTACAGGTCTCTTTCCACATCC
CAAACTTTCTATGATGCTTACATTTCTTATGACACCAAAGATGCCTCTGTTACTGACTGGGTGATAAATG
AGCTGCGCTACCACCTTGAAGAGAGCCGAGACAAAAACGTTCTCCTTTGTCTAGAGGAGAGGGATTGGGA
CCCGGGATTGGCCATCATCGACAACCTCATGCAGAGCATCAACCAAAGCAAGAAAACAGTATTTGTTTTA
ACCAAAAAATATGCAAAAAGCTGGAACTTTAAAACAGCTTTTTACTTGGCTTTGCAGAGGCTAATGGATG
AGAACATGGATGTGATTATATTTATCCTGCTGGAGCCAGTGTTACAGCATTCTCAGTATTTGAGGCTACG
GCAGCGGATCTGTAAGAGCTCCATCCTCCAGTGGCCTGACAACCCGAAGGCAGAAGGCTTGTTTTGGCAA
ACTCTGAGAAATGTGGTCTTGACTGAAAATGATTCACGGTATAACAATATGTATGTCGATTCCGCAGGCT
GACAGGGCCCTGTACCTGATGTGCACCGTCCCAGCCTTCTGCAGGCTCACGGGGATGGCGCTAGGCCACC
TGTGGCGCAGCAGGACGGGGCCCCAGGATGCAGAGCTGTGGCCCCCGAGGACCCTGTGCGAGCTCTACTC
ATGGTACTTTAGGATGGCCCTCAGCGGGGAGGGGCAGGAGAAGGGCAAGGCAAGCCCTCGCATCGAGCAG
GTGGCCCATGGTGGCCGCAAGATGGTGGGGACATTGGGCCGTCTGGCCTTCCATGGGCTGCTCAAGAAGA
AATACGTGTTTTACGAGCAAGACATGAAGGCGTTTGGTGTAGACCTCGCTCTGCTGCAGGGCGCCCCGTG
CAGCTGCTTCCTGCAGAGAGAGGAGACGTTGGCATCGTCAGTGGCCTACTGCTTCACCCACCTGTCCCTG
CAGGAGTTTGTGGCAGCCGCGTATTACTATGGCGCATCCAGGAGGGCCATCTTCGACCTCTTCACTGAGA
GCGGCGTATCCTGGCCCAGGCTGGGCTTCCTCACGCATTTCAGGAGCGCAGCCCAGCGGGCCATGCAGGC
AGAGGACGGGAGGCTGGACGTGTTCCTGCGCTTCCTCTCCGGCCTCTTGTCTCCGAGGGTCAATGCCCTC
CTGGCCGGCTCCCTGCTGGCCCAAGGCGAGCACCAGGCCTACCGGACCCAGGTGGCTGAGCTCCTGCAGG
GCTGCCTGCGCCCCGATGCCGCAGTCTGTGCACGGGCCATCAACGTGTTGCACTGCCTGCATGAGCTGCA
GCACACCGAGCTGGCCCGCAGCGTGGAGGAGGCCATGGAGAGCGGGGCCCTGGCCAGGCTGACTGGTCCC
GCGCACCGCGCTGCCCTGGCCTACCTCCTGCAGGTGTCCGACGCCTGTGCCCAGGAGGCCAACCTGTCCC
TGAGCCTCAGCCAGGGCGTCCTTCAGAGCCTGCTGCCCCAGCTGCTCTACTGCCGGAAGCTCAGGCTGGA
CACCAACCAGTTCCAGGACCCCGTGATGGAGCTGCTGGGCAGCGTGCTGAGTGGGAAGGACTGTCGCATT
CAGAAGATCAGCTTGGCGGAGAACCAGATCAGTAACAAAGGGGCCAAAGCTCTGGCCAGATCCCTCTTGG
TCAACAGAAGTCTGACCTCTCTGGACCTCCGCGGTAACTCCATTGGACCACAAGGGGCCAAGGCGCTGGC
AGACGCTTTGAAGATCAACCGCACCCTGACCTCCCTGAGCCTCCAGGGCAACACCGTTAGGGATGATGGT
GCCAGGTCCATGGCTGAGGCCTTGGCCTCCAACCGGACCCTCTCCATGCTGCACCTGCAGAAGAACAGCA
TCGGGCCCATGGGAGCCCAGCGGATGGCAGATGCCTTGAAGCAGAACAGGAGTCTGAAAGAGCTCATGTT
CTCCAGTAATAGTATTGGTGATGGAGGTGCCAAGGCCCTGGCTGAGGCCCTGAAGGTGAACCAGGGCCTG
GAGAGCCTGGACCTGCAGAGCAATTCCATCAGTGACGCAGGAGTGGCAGCACTGATGGGGGCCCTCTGCA
CCAACCAGACCCTCCTCAGCCTCAGCCTTCGAGAAAACTCCATCAGTCCCGAGGGAGCCCAGGCCATCGC
TCATGCCCTCTGCGCCAACAGCACCCTGAAGAACCTGGACCTGACAGCCAACCTCCTCCACGACCAGGGT
GCCCGGGCCATCGCAGCAGTTTCTGGACGGAGACGGGTGGACTTCCCGCTGGATCGAATCCAAACACAAG
TCAGATTTTGGCAAATTCGTTCTCAGTTCCGGCAAGTTCTACGGTGACGAGGAGAAAGATAAAGGTTTGC
AGACAAGCCAGGATGCACGCTTTTATGCTCTGTCGGCCAGTTTCGAGCCTTTCAGCAACAAAGGCCAGAC
GCTGGTGGTGCAGTTCACGGTGAAACATGAGCAGAACATCGACTGTGGGGGCGGCTATGTGAAGCTGTTT
CCTAATAGTTTGGACCAGACAGACATGCACGGAGACTCAGAATACAACATCATGTTTGGTCCCGACATCT
GTGGCCCTGGCACCAAGAAGGTTCATGTCATCTTCAACTACAAGGGCAAGAACGTGCTGATCAACAAGGA
CATCCGTTGCAAGGATGATGAGTTTACACACCTGTACACACTGATTGTGCGGCCAGACAACACCTATGAG
GTGAAGATTGACAACAGCCAGGTGGAGTCCGGCTCCTTGGAAGACGATTGGGACTTCCTGCCACCCAAGA
AGATAAAGGATCCTGATGCTTCAAAACCGGAAGACTGGGATGAGCGGGCCAAGATCGATGATCCCACAGA
CTCCAAGCCTGAGGACTGGGACAAGCCCGAGCATATCCCTGACCCTGATGCTAAGAAGCCCGAGGACTGG
GATGAAGAGATGGACGGAGAGTGGGAACCCCCAGTGATTCAGAACCCTGAGTACAAGGGTGAGTGGAAGC
CCCGGCAGATCGACAACCCAGATTACAAGGGCACTTGGATCCACCCAGAAATTGACAACCCCGAGTATTC
TCCCGATCCCAGTATCTATGCCTATGATAACTTTGGCGTGCTGGGCCTGGACCTCTGGCAGGTCAAGTCT
GGCACCATCTTTGACAACTTCCTCATCACCAACGATGAGGCATACGCTGAGGAGTTTGGCAACGAGACGT
GGGGCGTAACAAAGGCAGCAGAGAAACAAATGAAGGACAAACAGGACGAGGAGCAGAGGCTTAAGGAGGA
GGAAGAAGACAAGAAACGCAAAGAGGAGGAGGAGGCAGAGGACAAGGAGGATGATGAGGACAAAGATGAG
GATGAGGAGGATGAGGAGGACAAGGAGGAAGATGAGGAGGAAGATGTCCCCGGCCAGGCCAAGGACGACG
GAAGCCGGCAGAAGGTACTGGTGATGGAGTACTGCTCCAGTGGGAGCCTGCTGAGTGTGCTGGAGAGCCC

TGAGAATGCCTTTGGGCTGCCTGAGGATGAGTTCCTGGTGGTGCTGCGCTGTGTGGTGGCCGGCATGAAC
CACCTGCGGGAGAACGGCATTGTGCATCGCGACATCAAGCCGGGGAACATCATGCGCCTCGTAGGGGAGG
AGGGGCAGAGCATCTACAAGCTGACAGACTTCGGCGCTGCCCGGGAGCTGGATGATGATGAGAAGTTCGT
CTCGGTCTATGGGACTGAGGAGTACCTGCATCCCGACATGTATGAGCGGGCGGTGCTTCGAAAGCCCCAG
CAAAAAGCGTTCGGGGTGACTGTGGATCTCTGGAGCATTGGAGTGACCTTGTACCATGCAGCCACTGGCA
GCCTGCCCTTCATCCCCTTTGGTGGGCCACGGCGGAACAAGGAGATCATGTACCGGATCACCACGGAGAA
GCCGGCTGGGGCCATTGCAGGTGCCCAGAGGCGGGAGAACGGGCCCCTGGAGTGGAGCTACACCCTCCCC
ATCACCTGCCAGCTGTCACTGGGGCTGCAGAGCCAGCTGGTGCCCATCCTGGCCAACATCCTG

>Trichechus manatus latirostris tlr3-tlr8-nlrc3-calr-ikbke
CCTTTAAAAACCTACAGAACTTAATCAAATTAGATCTAGCTCATAATGGTTTATCATCTACAAAATTAGG
AACTGGACTCCAGCTGGAAAATCTCCAAGAGCTTTTACTGTCAAATAATAAAATTCATGCCTTAAGACGT
GAAGAACTTGATTTCCTTGGCAATTCTTCTTTAAAAAAATTAGACTTGTCATCAAATCAAATTAAAGAGT
TCTCTCCAGGGTGTTTTCATGCAATCGGAAAATTATACGGCCTTTTTCTGAGCAACGTCCAGCTGGGCCC
CGGTCTCACGGAGAAGCTTTGTTTGGAATTATCAAACTCCAGCATTCAGAGCCTATATCTGAGCAACATC
CAGCTCTACAGAACGAGCAATATGACATTTTTTGGACTGAAGCAGACAAATCTCACCAAGCTCGATCTTT
CCCATAACAGTTTAAGTATGATCGCTAACGATTCCTTTGCCTGGCTTCCACGTTTGGAATATTTCACCCT
GGAGTATAATAATATAGAGCATTTGTCTTCTCGCTGCTTTTATGGGCTGTTCAGTGTGAGATACCTGAAT
TTAAAACGATCTTTTACGAAACAAAGTAATTCCCTTGCTTCACTTCCCAAGATTGATGACTTTTCCTTTC
AGTGGCTAAAATGTTTGGAGTACCTTAACATGGAAGATAACGATTTTCCAGGCATAAAAAGCAATACGTT
CACAGGATTGATACGTCTGCAATACTTAAGTCTCTCCAGCTCCTTTACGAGTTTGCGAACTTTAACAAAT
GAAACATTTTTATCACTTGCGCGTTCTCCTTTACTCGTACTCAACCTAACCAGAAATAAAATCTCAAAAA
TAGACAGCGGTGCTTTCTCTTGGTTGCACCACCTGGAGGTACTGGACCTTGGCCTGAATGAAATTGGGCA
AGAACTCACAGGCCAGGAATGGAAAGGTCTAGAAAATATTGTTAAAATCTACCTTTCCTATAACAAAGAC
CTACAATTGACTAGCAATTCCTTTGCCTTGGTTCCAAGCCTTCAAGGACTGATGCTCCGAAGAGTGGCCT
GTAAAAGTGTGGATATCTCCCCGTCACCTTTCTGCCCTCTTCGTAACTTGACCGTTCTGGATCTCAGTAA
CAACAATATAGCCAACATAAAGGAGGACACGTTCGAGGGACTTGACAAACTAGAAATTCTGGATTTGCAG
CACAACAACTTAGCACGGCTCTGGAAGCACGCCAACCCTGGTGGTCCTGTTCATTTCTTAAAGGGTCTTT
CTCACCTCCACATACTTAACTTAGAGTCTAACGGCTTTGACGAGATCCCAGAGGAGGTCTTCAAGGACTT
GTCTGCATTAAAGAGCATTGATTTAGGATTGAATAATTTAAACATACTTCCACCATCTCTGTTTGATAGT
CAGACCTCTCTGAAGTCATTGAACCTTCAGAAGAATCTTATAACATCAGTTGAGAAGGATGTTTTCGGAC
CACCTTTCAAGAATTTGACTAATTTAGATATGCGCTTTAATCCGTTTGACTGTACCTGTGAAAGTATTGC
CTGGTTTGTTAACTGGATTAACGGAACCCGTGCCAACATCTCCGGTCTACCAGACGATTATTTCTGCAAC
ACTCCACCCCAGTATCATGGTTTGTCAGTGACATTCTTTGACATATCAGCCTGCAAAGACAGTGCCCCTT
TTGAACTCCTGTTCGTAATCAACACGAGTACCCTGTTGTTTTTTATCTTCATTGTACTGCTCGTCCACTT
TGAAGGCTGGAGGATATCGTTTTATTGGAATGTTTCAGTACATCGAATTCTTGGTTTCAAAGAGATAGAC
AGACAGCCAGAGCAGTTTGAATTTGCAGCATATGTAATTCATGCCCATAAAGATAGAGATTGGGTCTGGC
AAAACTTCTCCCTCATGGAAGAAAAAGATCAATCTCTCAAATTTTGTCTGGAAGAAAGGGATTTTGAGGC
AGGTACCCTTGAACTTGAAGCAATTGTCAACAGCATCAAAAGAAGCAGAAAAATTATTTTTGTTATAACA
CAGCATCTATTGAAAGATCCATTATGCAAAAGATTCAAAGTGCACCACGCAGTTCAGCAAGCTATTGAAC
AAAACCTGGATTCCATTATATTGATCTTTCTTGAGGAGATTCCAGATTATAAACTGAACCATGCACTTTG
TTTGCGAAGAGGAATGTTTAAATCCCGATGCATCTTGAACTGGCCAGTTCAGAAAGAACGGCTAAGTGCG
TATCGTCATAAATTGCAAGTAGCACTTGGATCCAGAAATTCAGTACATTAATTAGTAAAGCCACAATGTA
GCATTTACGGCAAAGCCTTAGATTTAAGCTTGAACAGTATTTTCTTCATTGGGCAAAAGCAATTTGAAGC
TTTTGGTGACATCGCCTGTTTAAATCTGTCTGCAAACGGCAATGGCCAAGTGTTACATGGAACTGAGTTT
TCGGCTGTGCCTCATGTCAAATACTTGGATTTGACAAACAACAGACTAGACTTTGATGATGACAAAGCTC
TCACTGAACTGGTTGAATTAGAAGTTCTAGATCTTAGCTATAACGCGCACTATTTCAGAATAGCTGGGGT
AACGCACCGTCTAGGATTCATCAAAAATTTAACACAGCTAAAAGTTTTAAACTTGAGCCACAACAGCATT
TACACTTTAACAGAGTATAATCTGACAAGCATGTCCCTGGAAGAATTAGTCTTCAGTGGAAACCGCCTTG
ATCTTTTGTGGAATGACGAGGACGACAGGTACTGGCATTTTTTCACATTTCTCAGGAATCTGACACGGCT
TGATCTATCCTTTAATAACCTTCGGCGCATCCCGAATGAAGCCTTCTCTAACTTGCCCCAGAGCCTCACA
GAACTATACATAAACAATAATATGTTACATTTCTTTAACTGGACACTACTCCAGCAGTTTCCTCACCTCC
GCTTGCTTGACTTAAGCAGGAACAAGCTGTCCACCTTAACTAACAGCCTGTCTACATTTACCTCTTCTCT
TCAGACCCTGCTGCTGAGGCAAAACAGGATCTCCCACCTGCCCTCTGGCTTCTTTTCTGAAGCCAGAGGT
CTGGTCCACCTCGATTTAAGTTCCAACCTGATAAAGATGATCAACAAATCCACGCTTCAAACGAAGACAG
CCACCAACCTCGCTGTTTTAGAACTAGGAGGAAACCCTTTTGACTGCACATGTGACATTGGAGATTTTCG
AAGATGGATGGATGAAAATCTGAATGTCTCAATTCCTAGACTGACAGATGTCATTTGTGCCAGTCCTGGA
GATCAAAGAGGGAAGTGTATTGTGAGTCTAGAGCTAACAACTTGCGTTTCAGATGCCATCGCGGCAATAT
TCTTCTTCTTCACCTTCTTTGTTACCACCATGGTTATGTTGACGGCCGTGGCCCACCATCTGTTTTACTG
GGATGTTTGGTTTATCTACCACGTGTGCTTAGCGAAGATAAAAGGGTACAGGTCTCTTTCCACATCTCAA
ACTTTCTATGATGCCTACATTTCTTATGACACCAAAGATGCCTCTGTCACCGACTGGGTGCTAAATGAGC

TGCGCTACCACCTCGAGGAGAGTGAAGACAAAAATGTTCTCCTGTGTTTGGAGGAGAGGGATTGGGACCC
GGGATTAGCCATCATCGATAACCTCATGCAGAGCATCAACCAAAGCAAAAAAACCATATTTGTTTTAACC
AAAAAATATGCAAAGAACTGGAACTTTAAGACAGCTTTCTACTTGGCCCTGCAGAGGCTAATGGAGGAGA
ACATGGACGTGATCATTTTCATTCTGCTGGAGCCAGTGCTACAGCATTCTCAGTATCTGAGGCTGCGGCA
GCGGATCTGCAAGAGCTCCATCCTCCAGTGGCCTGACAACCCCAAGGCAGAAGGCCTGTTCTGGCAAAGT
CTGAAAAACGTGGTCTTGACTGAAAATGATTCACGGTATAACAATTTGTACGTTGATTCCGCAGGCCGAC
AGGGCTCTCTACCTGATGTGCACCGTCCCTGCCTTCTGCCGGCTTTCAGGGTCAGCGCTGGGCCACTTGT
ACCGCCACAGGCGAGGGCCCCTGAATGCCGAGCTGTCACCCCCAAGAACCCTGAGCGAGATCTACTCCTG
GTACTTCAGGATGGCACTCAGCAGGGAGGGGCAGGACAAGGGCAAGGTGAGCCCACGCATCGAGCAGGTG
GCCCACGGGGGCCGCAGGATGGCGGGGACGCTGGGCCGGCTGGCCTTCCAAGGGCTGGTCAGGAAGAAGT
ACGTGTTTTACGAGCAGGACCTGAGGGCCTTCGGGGTGGACCTGGCCCTGCTGCAGAGTGCCCTGAGTGG
CTGCTTCCTGCAGCGGGAGGAGACACTGGGCTCTGCGGCCGCCTACTGCTTCTCCCACCTGTCCCTGCAG
GAATTTGTGGCAGCTGCCTATTACTACGGTGCGTCCAAGAGGGCCATCTTCGATCTCTTCACAGAGAGCG
GCGTGTCCTGGCCCCGGCTGGGCTTCCTCACCCACTTCCGGAGTGCGGTGCAGAGGGCCATGCAGGCCGA
GGACGGCAGGCTGGACGTGTTCCTGCGCTTCCTCTCGGGCCTCCTGTCCCCCAGGGTCAACACCCTGCTG
ATGGGCTCCCTGCTGGCCCCGGGCGAGCACCAGGGCTACCGGGAGCAGGCGGCCAGGCTCCTGCAGGGCT
GCCTGGCCCCCGAGGCTGCCGTCAGCGCCCGGGCTGTCAATGTCCTGCGCTGCCTGCACGAGCTGCAGCA
CACAGAGCTGGCCAGCAGCGTGCAGGAGGCCATGGAGCGCGAGGGCCTGGCCAGGCTGACCAGCCCCCCA
CACCCCGCTGCCCTGGCCTACCTGCTGCAGGTGTCGGATGCCTATGCCCAGGAGGCCAACCTGTCCCTGT
GCCTCAGCCAGGCCATCCTCCAGAGCCTGCTGCCCCAGCTGCTCTACTGCCAGAGCCTCAGGCTGGACAC
CAACCAGTTCCAGGACCCCGTGATGGAGTTGCTGGGCAGTGTGCTGAGCGGGAAGGACTGTCGCATCCAG
AAGATCAGCTTGGCTGAGAACCAGATTAGCAACAAAGGAGCCAAAGCTCTGGCCAGATCCCTCCTGGTCA
ACAGAAGTCTGACCACTCTGGACCTCCGCGGTAACTCCATTGGGCCTCAAGGGGCCAAGGCCCTGGCAGA
TGCTCTGAAGATTAACCGGACTCTGGCTTCTCTGAGCCTCCAGAACAACATGATCAAGGACGATGGTGCT
GGGTCCATAGCTGAGGCCTTGGCCTCCAGCCGGACCCTCTGCATGCTACACTTGCAGAAGAACTCCATTG
GGCCCGTGGGAGCCCAGCGGATGGCAGACGCCCTGAAGCAGAACAGGAGTCTGAAGGAGCTCATGTTCTC
CAGTAACAGCATAGGTGATGGAGGCGCCAAGGCCCTGGCTGAGGCCCTGAAGGTGAATCAGGGCCTGGAG
AGCCTGGACCTGCAGAGCAATTCCATCAGCAACGCTGGGGTGGCTGCACTGATGGGGGCCCTCTACACCA
ACCAGACCCTCCTCAGCCTCAACCTCCGAGAAAACTCCATCAATCCAGAGGGAGCCCAGGCTCTTGCCCG
CGCCCTCTGCACCAACAGCACGCTGAAGAACCTGGACCTGACAGCCAACCTCCTCCGCGACCAGGGTGCC
CAGGCCATCGCCGGAGTTCGAAGATGGAGATGGATGGAAAAACCGATGGGTGGAATCTAAACATAGTCCG
GATTATGGTAAATTTCAGCTCACAGCAGGAAAATTTTATGCAGACAAAGAAAAAGCTAAAGGTCTCCAGA
CCAGCGAAGATGCCAAGTTGTACGCCCTCTCAACCAGATTTAAGCCATTCAGCAATGAAAATGAAAGCGT
GGTGGTTCAGTTTTCAGTAAAACATGAGCAAGGCATCAATTGTGGTGGTGGATATGTGAAACTCTTCCCT
GACACTCTGAACCAAGAGGATATGCATTCAGAGTCTGAGTATTATATCATGTTTGGCCCTGACATCTGTG
GCTTTGGCAACAATAAGGTACAGGTCATCCTTCATTACCAAGGGAAATACCATGGGAACAATAAGACCAT
CGAATGTCGGATCAATAAAGACACTCACCTATACACTCTAGTCATTCGTCCCAACGCTACTTATGAGGTC
AAAATTGTTAACCAGCAAATGGCAGCCGTGGATCTGGAGGACGACTGGGACTTCTTGCCGCCCAAGAAGA
TAAAAGACCCCTATGCCCAGAAACCAAGGAAATGGGATGAGCATCTGCAAATAGAGGATCCTGAAGATGA
GAAACCTGAGGATTGGGATGACTTTGAGTACATCCCAGACCCAGAGGCCAAGAAGCCGGATGACTGGAAT
GAGGCAATGGATGGGAAGTGGGAAGAGCCCCTGATTCCAAATCCCAAGTATAAGGGGAGGAGTCTCTCTT
TCCAATAGGGACAATGGAAGCCTCGGATCATTGACAATCCTAATTACCAAGGGGAGTGGATTCATCCAGA
AATAGACAACCCTAAATATAAGCCTGACCCCACCATTTGTCACGATTATAACATCAGTGTTCTTGGCCTG
GACCTTTGGCAGGTAAAATCAGGCAGCATCTTTGACAATTTCCTTATTACAAATGATAAAGAGTTTGCTG
AAGAAGTTGGAAACAAGACCTGGGGAATAAGAAAAGACTTAGAGAAGCAATGGAGAGAACTGTATGAAGA
AATGGAGAAAAGAAAACAGGAAGACGAGGCTGAGAAAAAAAAGGAAATGGAGGAGGAAGGGGAAGATGAG
ATCTGGGGAATCGAAGAACAGGGAGTGAAGAGGATACTGCTGAAGAGCTGGGAAATGACAGGCCGATGAG
GGCAGCCGGCAGAAGGTGCTGGTGATGGAGTACTGCTCCAGCGGGAGCCTGCTGAGCGTGCTTGAGAGCC
CTGAGAACGCCTTTGGGCTGCCCGAGGACGAGTTCCTGGTGGTGCTGCGCTGTGTGGTGGCCGGCATGAA
CCACCTGAGGGAGAATGGCATTGTCCACCGCGACATCAAGCCTGGAAATATCATGCGCCTCATGGGGGAG
GAGGGCCAGAGCATCTACAAGCTAACGGACTTCGGGGCTGCCCGGGAGCTGGATGACGATGAGAAGTTCG
TCTCAGTCTACGGGACTGAGGAGTACCTGCACCCCGACATGTATGAGCGGGCGGTACTTCGCAAGCCCCA
GCAGAAGGCGTTTGGGGTGACTGTGGATCTCTGGAGCATTGGGGTGACCCTGTACCATGCAGCCACCGGC
AGCCTGCCCTTCACCCCCTTTGGTGGGCCGCGACGTAACAAGGAGATCATGTACCGGATCACCACGGAGA
AGCCAGCTGGGGCCATTGCAGGAACTCAGCGGCGGGAAAACGGGCCCCTGGAGTGGAGCTACACCCTCCC
CATCACCTGCCAGCTGTCGATGGGGCTGCAGAGCCAGCTGGTGCCCATCCTGGCCAACATCCTG

>Mus musculus tlr3-tlr8-nlrc3-calr-ikbke
CTTTCAAAAACCAGAAGAATCTAATCAAATTAGATTTGTCTCATAATGGTTTATCATCTACAAAGTTGGG
AACGGGGGTCCAACTGGAGAACCTCCAAGAACTGCTCTTAGCAAAAAATAAAATCCTTGCGTTGCGAAGT
GAAGAACTTGAGTTTCTTGGCAATTCTTCTTTACGAAAGTTGGACTTGTCATCAAATCCACTTAAAGAGT

TCTCCCCGGGGTGTTTCCAGACAATTGGCAAGTTATTCGCCCTCCTCTTGAACAACGCCCAACTGAACCC
CCACCTCACAGAGAAGCTTTGCTGGGAACTTTCAAACACAAGCATCCAGAATCTCTCTCTGGCTAACAAC
CAGCTGCTGGCCACCAGCGAGAGCACTTTCTCTGGGCTGAAGTGGACAAATCTCACCCAGCTCGATCTTT
CCTACAACAACCTCCATGATGTCGGCAACGGTTCCTTCTCCTATCTCCCAAGCCTGAGGTATCTGTCTCT
GGAGTACAACAATATACAGCGTCTGTCCCCTCGCTCTTTTTATGGACTCTCCAACCTGAGGTACCTGAGT
TTGAAGCGAGCATTTACTAAGCAAAGTGTTTCACTTGCTTCACATCCCAACATTGACGATTTTTCCTTTC
AATGGTTAAAATATTTGGAATATCTCAACATGGATGACAATAATATTCCAAGTACCAAAAGCAATACCTT
CACGGGATTGGTGAGTCTGAAGTACCTAAGTCTTTCCAAAACTTTCACAAGTTTGCAAACTTTAACAAAT
GAAACATTTGTGTCACTTGCTCATTCTCCCTTGCTCACTCTCAACTTAACGAAAAATCACATCTCAAAAA
TAGCAAATGGTACTTTCTCTTGGTTAGGCCAACTCAGGATACTTGATCTCGGCCTTAATGAAATTGAACA
AAAACTCAGCGGCCAGGAATGGAGAGGTCTGAGAAATATATTTGAGATCTACCTATCCTATAACAAATAC
CTCCAACTGTCTACCAGTTCCTTTGCATTGGTCCCCAGCCTTCAAAGACTGATGCTCAGGAGGGTGGCCC
TTAAAAATGTGGATATCTCCCCTTCACCTTTCCGCCCTCTTCGTAACTTGACCATTCTGGACTTAAGCAA
CAACAACATAGCCAACATAAATGAGGACTTGCTGGAGGGTCTTGAGAATCTAGAAATCCTGGATTTTCAG
CACAATAACTTAGCCAGGCTCTGGAAACGCGCAAACCCCGGTGGTCCCGTTAATTTCCTGAAGGGGCTGT
CTCACCTCCACATCTTGAATTTAGAGTCCAACGGCTTAGATGAAATCCCAGTCGGGGTTTTCAAGAACTT
ATTCGAACTAAAGAGCATCAATCTAGGACTGAATAACTTAAACAAACTTGAACCATTCATTTTTGATGAC
CAGACATCTCTAAGGTCACTGAACCTCCAGAAGAACCTCATAACATCTGTTGAGAAGGATGTTTTCGGGC
CGCCTTTTCAAAACCTGAACAGTTTAGATATGCGCTTCAATCCGTTCGACTGCACGTGTGAAAGTATTTC
CTGGTTTGTTAACTGGATCAACCAGACCCACACTAATATCTCTGAGCTGTCCACTCACTACCTCTGTAAC
ACTCCACATCATTATTATGGCTTCCCCCTGAAGCTTTTCGATACATCATCCTGTAAAGACAGCGCCCCCT
TTGAACTCCTCTTCATAATCAGCACCAGTATGCTCCTGGTTTTTATACTTGTGGTACTGCTCATTCACAT
CGAGGGCTGGAGGATCTCTTTTTACTGGAATGTTTCAGTGCATCGGATTCTTGGTTTCAAGGAAATAGAC
ACACAGGCTGAGCAGTTTGAATATACAGCCTACATAATTCATGCCCATAAAGACAGAGACTGGGTCTGGG
AACATTTCTCCCCAATGGAAGAACAAGACCAATCTCTCAAATTTTGCCTAGAAGAAAGGGACTTTGAAGC
AGGCGTCCTTGGACTTGAAGCAATTGTTAATAGCATCAAAAGAAGCCGAAAAATCATTTTCGTTATCACA
CACCATTTATTAAAAGACCCTCTGTGCAGAAGATTCAAGGTACATCACGCAGTTCAGCAAGCTATTGAGC
AAAATCTGGATTCAATTATACTGATTTTTCTCCAGAATATTCCAGATTATAAACTAAACCATGCACTCTG
TTTGCGAAGAGGAATGTTTAAATCTCATTGCATCTTGAACTGGCCAGTTCAGAAAGAACGGATAAATGCC
TTTCATCATAAATTGCAAGTAGCACTTGGATCTCGGAATTCAGCACATTAATTAATAAAGCCACAGTGTA
CTGCTTATGGCAAGGCCTTGGATTTAAGTTTGAACAATATTTTCATTATTGGGAAAAGCCAATTTGAAGG
TTTTCAGGATATCGCCTGCTTAAATCTGTCCTTCAATGCCAATACTCAAGTGTTTAATGGCACAGAATTC
TCCTCCATGCCCCACATTAAATATTTGGATTTAACCAACAACAGACTAGACTTTGATGATAACAATGCTT
TCAGTGATCTTCACGATCTAGAAGTGCTGGACCTGAGCCACAATGCACACTATTTCAGTATAGCAGGGGT
AACGCACCGTCTAGGATTTATCCAGAACTTAATAAACCTCAGGGTGTTAAACCTGAGCCACAATGGCATT
TACACCCTCACAGAGGAAAGTGAGCTGAAAAGCATCTCACTGAAAGAATTGGTTTTCAGTGGAAATCGTC
TTGACCGTTTGTGGAATGCAAATGATGGCAAATACTGGTCCATTTTTAAAAGTCTCCAGAATTTGATACG
CCTGGACTTATCATACAATAACCTTCAACAAATCCCAAATGGAGCATTCCTCAATTTGCCTCAGAGCCTC
CAAGAGTTACTTATCAGTGGTAACAAATTACGTTTCTTTAATTGGACATTACTCCAGTATTTTCCTCACC
TTCACTTGCTGGATTTATCGAGAAATGAGCTGTATTTTCTACCCAATTGCCTATCTAAGTTTGCACATTC
CCTGGAGACACTGCTACTGAGCCATAATCATTTCTCTCACCTACCCTCTGGCTTCCTCTCCGAAGCCAGG
AATCTGGTGCACCTGGATCTAAGTTTCAACACAATAAAGATGATCAATAAATCCTCCCTGCAAACCAAGA
TGAAAACGAACTTGTCTATTCTGGAGCTACATGGGAACTATTTTGACTGCACGTGTGACATAAGTGATTT
TCGAAGCTGGCTAGATGAAAATCTGAATATCACAATTCCTAAATTGGTAAATGTTATATGTTCCAATCCT
GGGGATCAAAAATCAAAGAGTATCATGAGCCTAGATCTCACGACTTGTGTATCGGATACCACTGCAGCTG
TCCTGTTTTTCCTCACATTCCTTACCACCTCCATGGTTATGTTGGCTGCTCTGGTTCACCACCTGTTTTA
CTGGGATGTTTGGTTTATCTATCACATGTGCTCTGCTAAGTTAAAAGGCTACAGGACTTCATCCACATCC
CAAACTTTCTATGATGCTTATATTTCTTATGACACCAAAGATGCATCTGTTACTGACTGGGTAATCAATG
AACTGCGCTACCACCTTGAAGAGAGTGAAGACAAAAGTGTCCTCCTTTGTTTAGAGGAGAGGGATTGGGA
TCCAGGATTACCCATCATTGATAACCTCATGCAGAGCATAAACCAGAGCAAGAAAACAATCTTTGTTTTA
ACCAAGAAATATGCCAAGAGCTGGAACTTTAAAACAGCTTTCTACTTGGCCTTGCAGAGGCTAATGGATG
AGAACATGGATGTGATTATTTTCATCCTCCTGGAACCAGTGTTACAGTACTCACAGTACCTGAGGCTTCG
GCAGAGGATCTGTAAGAGCTCCATCCTCCAGTGGCCCAACAATCCCAAAGCAGAAAACTTGTTTTGGCAA
AGTCTGAAAAATGTGGTCTTGACTGAAAATGATTCACGGTATGACGATTTGTACATTGATTCCGCAGGCC
AACAGGGCTCTGTATCTGATGTGCACTGTACCAGCCTTTTGTAGGCTCACGGGGCTGGCTCTGGGTCACT
TGTATCGCACCAGGCTGGCCGTCCAAGACATAGAGCTGCCATTGCCTCAGACCCTGTGTGAGCTCTACTC
TTGGTACTTTAGGATGGCTCTTGGTGGGGAGGGCCAGGATAAGGAAAAGGTAAGTCCTAGGATCAAGCAG
GTGACCCAGGGAGCTCGCAAAATGGTGGGGACATTGGGCCGCCTGGCCTTCCATGGGCTGGTCAAGAAGA
AATACGTGTTTTATGAACAAGACATGAAGGCATTTGGAGTGGACCTCGCTCTGTTGCAGAACACTCTGTG
CAGCTGTCTCCTGCAGCGGGAAGAGACCCTGGCCTCCTCTGTAGCTTACTGCTTCATTCACCTGTCTCTG
CAAGAATTTGTGGCAGCTACATATTACTATAGTGCATCCAAGAGGGCCATCTTTGACCTCTTCACCGAGA

GTGGCATGTCCTGGCCCAGACTGGGTTTCCTCGCCCATTTCAGGTGTGCAGCCCAGCGGGCCACACAAGC
TAAGGATGGAAGGCTGGATGTGTTTCTGCGCTTCCTCTCTGGCCTCTTGTCCCCAAGGGTCAATACTCTG
CTGGCCGGCTCCCTGTTGTCCCAAGGCGAGCATCAGAGCTACCGGGACCAGGTGGCTGAGGTCCTACAAG
GCTTCCTTCATCCTGACGCTGCAGTCTGTGCACGTGCCATCAATGTCTTGTACTGCCTAAGTGAGCTGCG
GCACACAGAACTGGCCTGCAGTGTGGAGGAGGCCATGCGGAGTGGGACCTTGGCTGGGATGACCAGCCCC
TCACACCGCACTGCTCTGGCCTACCTCCTGCAGATGTCTGACATCTGCTCCCCAGAGGCTGACTTCTCCC
TGTGTCTCAGCCAGCATGTCCTCCAGAGCCTGCTGCCCCAGCTGCTCTATTGTCAAAGCCTCAGGCTGGA
CAACAACCAGTTCCAGGACCCTGTGATGGAGTTGCTGGGCAGCGTGCTGAGTGGGAAGGACTGTCGCATT
CGAAAGATCAGCCTGGCTGAGAATCAGATTGGTAACAAAGGAGCCAAAGCCCTGGCCAGATCCCTCCTGG
TTAACAGAAGCCTCATCACACTGGACCTCCGGAGTAACAGCATTGGACCACCGGGGGCTAAGGCTTTGGC
CGATGCTCTGAAGATAAACCGAACGCTAACTTCTCTAAGCCTCCAAAGCAACGTGATCAAGGATGACGGT
GTCATGTGCGTGGCTGAGGCCCTGGTCTCCAACCAGACCATCTCCATGCTACAGCTACAGAAGAACTTAA
TTGGGCTCATAGGAGCCCAGCAGATGGCAGATGCCCTGAAGCAGAACAGGAGCCTGAAAGCACTCATGTT
TTCCAGTAATACCATTGGCGACAGAGGTGCCATAGCCCTGGCTGAGGCCCTGAAGGTGAACCAGATCCTG
GAGAACTTAGACCTACAGAGCAATTCCATCAGTGACATGGGAGTGACGGTGCTGATGCGAGCCCTCTGCA
GTAACCAGACACTCTCCAGTCTCAACCTGCGAGAAAACTCCATCAGCCCAGAGGGAGCCCAGGCCCTCAC
TCAAGCTCTCTGCAGGAACAACACTCTGAAGCACTTGGACCTGACAGCTAATCTCCTCCATGACCGAGGT
GCCCAGGCCATTGCAGCAGTTCTTGGACGGAGATGCCTGGACCAACCGCTGGGTCGAATCCAAACATAAG
TCCGATTTTGGCAAATTTGTCCTCAGTTCTGGCAAATTTTACGGGGACCTGGAGAAGGATAAAGGGCTGC
AGACAAGCCAAGATGCCCGATTTTACGCACTGTCCGCCAAATTCGAACCCTTCAGCAATAAGGGCCAGAC
ACTGGTGGTACAGTTCACGGTGAAGCATGAGCAGAATATCGACTGTGGGGGCGGCTACGTGAAGCTGTTT
CCGAGTGGTTTGGACCAGAAGGACATGCATGGAGACTCAGAATATAACATCATGTTTGGTCCGGACATCT
GCGGTCCTGGCACCAAGAAGGTTCATGTCATCTTTAACTACAAGGGCAAGAATGTGCTGATCAACAAGGA
TATCCGGTGTAAGGATGATGAATTCACACACCTATACACACTGATTGTGCGGCCAGACAACACCTATGAG
GTGAAAATTGACAACAGCCAGGTGGAGTCAGGCTCCTTGGAGGATGATTGGGACTTTCTGCCACCCAAGA
AGATAAAGGACCCTGATGCTGCCAAGCCGGAAGACTGGGATGAACGAGCCAAGATCGATGACCCCACAGA
TTCCAAGCCTGAGGACTGGGACAAGCCAGAGCACATCCCTGACCCTGATGCTAAGAAGCCTGAGGACTGG
GATGAAGAGATGGATGGAGAGTGGGAACCACCAGTGATTCAAAATCCTGAATACAAGGGCGAGTGGAAAC
CACGTCAAATTGACAACCCAGATTACAAGGGTACCTGGATACACCCAGAAATTGACAACCCTGAATACTC
CCCCGATGCAAATATCTATGCCTATGATAGTTTTGCTGTACTGGGCCTAGATCTCTGGCAGGTCAAGTCC
GGGACAATCTTTGACAATTTCCTCATCACCAATGATGAGGCCTATGCAGAGGAGTTTGGCAATGAGACGT
GGGGTGTTACCAAGGCTGCAGAGAAGCAGATGAAGGACAAGCAGGATGAGGAGCAGAGGCTTAAGGAAGA
AGAAGAGGACAAGAAGCGTAAAGAGGAAGAAGAAGCTGAGGATAAAGAGGATGATGATGACAGAGATGAA
GATGAGGACGAAGAAGATGAGAAGGAGGAAGATGAGGAAGAATCCCCTGGCCAAGCCAAGGATGAAGGCA
GCCGGCAGAAGGTGCTAATCATGGAGTACTGCTCCAGTGGGAGCCTGCTGAGCGTGCTGGAAGACCCTGA
GAACACGTTCGGGCTTTCTGAAGAGGAGTTCCTAGTGGTGCTGCGCTGTGTGGTGGCTGGCATGAACCAC
CTGCGGGAGAATGGCATTGTCCATCGGGACATCAAACCTGGGAACATCATGCGCCTGGTGGGCGAGGAGG
GGCAGAGCATCTATAAGCTGTCTGACTTCGGGGCTGCCCGCAAGCTGGACGATGATGAGAAGTTTGTTTC
TGTCTATGGTACAGAGGAATACCTGCACCCTGACATGTATGAGCGTGCAGTGCTGCGCAAACCCCAGCAA
AAGGCATTTGGTGTGACTGTGGATCTCTGGAGTATTGGGGTGACCCTGTACCACGCAGCCACAGGCAGTC
TGCCCTTCATCCCCTTCGGTGGGCCCCGGCGCAACAAAGAGATCATGTACAGAATCACCACAGAGAAGCC
AGCCGGGGCCATTTCAGGGACTCAGAAGCAGGAAAATGGTCCCTTGGAGTGGAGCTACAGCCTCCCCATC
ACCTGTAGACTGTCCATGGGACTGCAGAACCAGCTGGTGCCCATCCTGGCCAACATCCTG

>Ornithorhynchus anatinus tlr3-tlr8-nlrc3-calr-ikbke
CATTTATCCCCCACCAGAACTTGAATATACTGGACCTCTCCCACAACCACCTGTCATCTGCGAAATTAGG
AACCCGCACCCAGCTGAGAAACCTCCAACAGCTTCTGCTGTCCAGCAACAAAATCGACATTCTGAAGGAG
GACGACCTGGAGTTCCTTGCCAATTCTTCCTTAAGCAAGCTGGACCTTTCCTCGAATCAGCTGAAAGTGT
TTTCTCCTGGCTGCTTCCGAGCCATAGGGCAACTGACCAGCCTGTGGCTGAACAACATGCCACTGGGGCC
CAAACTCACAGAGCAGTTGTGCGCCGAGCTGTCCGGGACGCACCTCCAGAGCCTTGCCCTAAGCAACACG
GAACTCCAGCGCACCACCAACACCACCTTCCAGGGCCTGGCCTCCACCAACCTCACCGCCCTCGACCTGT
CCCTCAACAGCCTGACTGTCATCGCTCCCAACTCCTTCGCCTGGCTCCCACACCTGGAGACCCTCAAGCT
GGAGAGCAACAGTCTGGTCCGCCTGCAGGCGTTCTCGTTCTCTGGCCTGTCCGGCCTCCGGCACCTGAGT
CTCAGAGCCGCCTTTGGGTGGTCCCACTGGCAGGGGAAGCCTGGCCCGGTGGTCGAGGATGGCGCCTTCG
CAGGGCTGCGATGCCTGGAGGTCCTGTCGCTGGAGCAGAACAACCTGCGCGAGGTGACGCCCGGCACCTT
CTTGGGTTTGGTCAGCCTGAAGCACCTCAGCCTATCCGGCTGCCATCAGGGCCTGCAGACATTGACCAAC
CGGACCTTCGCCTCTCTGGCCGCCTCGCCCCTGCTCCTGCTGAACCTCTCGCGGGCCAAGGTCGAGACCG
TTGAGGCCGGCGCCTTCTCCCAGCTGGGGCGGCTGCGGGTGCTGGACCTGGGTGTGAATGAGCTGCGGCA
ATGGCTCTCAGGCTATGAGTGGGTTGGTCTGGCCCAGCTGGAGGAGCTCTACCTGTCCTACAACCCACAG
GTGGGGATGAGCAGCTCGGCGTTCGCCTTGGTGCCCGGCCTACGGCGGCTCTTATTACGCCGGGTGGGCT
GCGGCAGCCTGGCTCTGGCCACGTCACCCTTCCGCCTACTGCGGAACCTGACCGTGCTGGACCTGAGCAA

CAACAACATCGCCGGCGTCAGCGCCGACCTGCTGGAGGGGCTGGACCGCCTGGAGGTCCTGGACCTGCAG
CACAACAACCTGGCTCGCTTGTGGAAGGCAGTCAATCCCGGCGGGCCCGTCCTCTTCCTGCGGGGCCTGT
CCAGCCTGCGCGCCCTTCAGCTGGAGTCCAACGGGCTGGACGAGGTGCCCGTCGGGGCCTTCCGTGGTCT
GGTGGCCCTCCGGACCCTGGACCTGGGCCTCAACAACCTGAACCTGCTGCCAGCCGGGCTGTTCGCTGAC
CTGGCCAGCTTGCGCTCGCTCAACTTGCAAAAGAACCTGCTCACGACGGTGCCCAGAGAGGTGTTTGGAC
CAGTCTTCCGCAACTTGAGCACTTTGGACCTGCGCTTCAACCCGTTCGACTGCACGTGCGCCAGCATCGC
CTGGTTCGTGGCCTGGTTGAACAGCACGAGAGCCACCATCCCCGAGCTGCACACCCAATACCTCTGCAAC
ACGCCGCCGCAGGCCCACGGCTCGCTCATGGTGCACTTCGACGACGCGCCCTGCAAGGACAGTGCCCCCT
TTGACATGTTCTTCATGGTTAGTGCCAGCGCTCTGTTCCTCTTCACCTCTGGCGTCCTGCTCGCTCGCTT
CCAGGGCTGGCGGCTCACGTTCTACTGGCACGTGGTTGGGGAGCGGGTCCTGGGCAGGTGGAGGGCCGGC
TGCGCCCCTGGCCAACCTGACTTTGCCGCCTACGTGATCCACGCCCAGCAGGACTCCCGCTGGGTCTGGA
AGAATTTTGCCTCTCTGGAGGAGCAGGATCCAGCCCTGCGCTTCTGCCTGGAGGAGCGGGACTTGGAGGC
CGGCCGGCCCGAACTCGAGGCCATCGTCAGAGGCATGAGGCGGAGCCGGAAGATCATCTTCGTGGTGACG
CAGCACCTCCTGGAGGATCCCCTGTGCAGAAGCTTCAAGGTGCACCACGCGGTCCATCAAGCCATCGAGC
AGAACCTGGACTCCATCGTCCTGGTCTTCCTGCAGGACATCCCAGACTACCGGCTGAACCACGCCCTGGG
CCTGCGCAGAGGCATGTTCCGCTCCCACTGTGTCCTGCACTGGCCGGCCTACCGGGAGCGCCTCTGTGCC
TTCCACCAGAAGCTCAAGGCTGCGCTTGGGGCCCCCAACACGATGCCCTGATTAATAAAACCGCAGTGCA
GTTCTTACGGTAAAGCCTTGGATCTTAGCTTGAACAACATTTTTTTTATCGGGCAAAAACAGTTTAAAGG
CTTTAATGAAATAGCCTGCTTAAATTTGTCTTCAAACGGCATCGGACAAGTTTTACACGGCACCGAGTTT
TCATCGTTACCTCACCTCAAATATTTGGATTTGTCATATAATAAGTTGGACTTTGATGACGATAATGCAT
TCCAAGAAGTGCCAGGCCTAGAGGTATTAGATCTCAGCTATAACATACACTATTTCAGGGAAGCAGGAAT
CACGCATCGCCTGGGATTTATTCAAAATCTAACGCAGCTGAAGGTATTAAACCTAAGCCACAATGCCATT
TTTACCCTCACCGAGCATAAGCTAATCAGCAGGTCCCTGGAAGAATTGGTTTTCAAAGGAAACCGTCTCG
ATATCTTGTGGAACCTTGAAGATGACAGGTACATTCAGATTTTCAGTAACCTCTGCAACCTAACACGACT
TGATCTCTCCAGCAACATGCTTCATGAGATCCCAGAAGAGGCATTCTTCAACCTCCCAGAGACTTTAACT
GAGCTATATCTAAGTAACAATCGATTAACGTTCTTCGATTGGACAGTGCTACGGCAACTCCCCTCTCTCC
AACTGCTGGATGTAGGTGGAAATGCGCTCACCAGTTTAACCAGTAACGTATTTGCGTTCACACCTTCACT
TCAGACGCTGGTACTGAGGAGAAACAGGATTTCCCAGCTCGCAGAGGGATTTCTCACGGAAGGGTTGAGC
CTTTTGCACCTGGACCTAAGCTTCAATCACCTGAAAATCATCAACCAGTCGACATTCCAAACCAAAGTAA
CGAATCACTTGACAATTTTGGACTTACGAGGAAATCCCTTCGACTGTAGCTGCAAAATGGGTGACATCCG
GAAGTGGATGGACGAAAACGAGAACGTCAGCATTCCCGGACTGGCGTCCGAAGTTGTGTGTGCCACACCT
GGAGACCAAACAGGGAGAAGCATCATAAGCTTAGACCTAACTACCTGTGTTTCAGATACCATTGCGGTCG
TTCTCTTTTGCCTCTCATTCTTGATCACAATTCATCTTATGCTGATAACTGTGGCTCATCACTTGTTCCA
TTGGGACGTGTGGTATATTTATCATTGGTGCAGAGCCAAGTTTAGAGGTTACAGATCTATCGCCACGTCA
AAAACCGTCTACGATGCGTATATTGCTTATGATACGACAGACCCTTTGGTGACCGACTGGGTAATAAATG
AGTTACGAGTTCACTTGGAAGAAAGTGAAGACAAGAAGGTTCTCCTTTGCCTAGAAGAAAGGGATTGGGA
CCCTGGGATAGCTATTATCGATAACCTCATTCAGAGCGTAAGTCAGAGCAATAAGACGGTATTTGTGCTG
ACGAAAAAGTATGCAAGAAACTGGAACTTTAAAACTGCTTTCTACTTAGCCTTGCAGAGGCTAATGGATG
AGAATGTGGATGTGATTATTTTCATTTTGCTGGAGCCTGTGTTGCAGAACTCTCAATATATGAGACTGAG
GAGGAGGATTTGTAAAAGCTCGATTCTCAACTGGCCCAATAACCCCAAGGCTGAAGGCTTCTTCTGGCAA
AGTCTGAAAAATGTGGTGTTGACAGAAAATGATACCAGGTATAACAGTCTGTATGCAAATTTCCGGGGCC
AATCCGGCCCTGTACTTACTGTGCACGGTCCCCTCCTTCTGCCGGCTCTGCGGCGTGGTCCTGGGCTGCC
TGCTCAAGGGCCGGCCCGACCGCCAGCTCCCCGAGGCCTCGTTCCCCAAGTCCCTGTCGGAGCTGTACGC
CTCCTACTTCAAGATGGCGCTGTGCGGGGACTGGCCGGACCGGCCGAAGGAGAGCCTCCGGACGGAGCAG
GCCGCCAGCGGCAAGAAACTGCTGGGGGCCCTGGGCCGGCTGGCCTTCTTCGGCTTGGTCAAGAAGAGAT
ACGTCTTCTCTGAGCCGGACCTCAAGGCCCACGGCGTGGAGCTCTCGCTGCTGCAGGCTAACCCCCGCCA
CTGCCTCTTCCTCAAGGAGGAGGCCCAGGGCTCCGTCCACTACTACTTCGCCCACCTGTCTCTGCAGGAG
TTCGCGGCCGCCGTTTTCTACTATTGCGCGGCCAAGCGGGCTCTCTTCGACCTCTTCTCCGATAACGGCA
TGTCCTGGTTCAAACTGGGCTTCCTCACCCACTACAGGAACGCCCTTCAGAGAGCGCTGCAGGCGGAGGA
CGGGCGGCTGGACGTTTTCCTGCGCTTCCTGTCCGGCCTGCTCTCCCCGCGGGTCAACCGGCTCCTGGCC
GGCTGGCTGCTGGTCAAGGACGAGCACGACGGTCACCGGGGCCCCGTCAGGGACCTCCTCCAGGACTGCC
TCCGCCCCATGGCCAACGTCTCCTCCCGCATGGTCAACATCGTGCGCTGCCTGCAGGAGCTCCAGTGCCC
GGAGCTGGCCCAGAGCGTGGAGGAGGCCATGGCGAGCGAGAGCCTGGCCGGGAAGCTGACCCCTCTCCAC
CGCTCGGCCCTGGCCTACGTGCTGCAGGTTTCGGAGGCGTGCGCCGACGAGACCAACCTCTCCAGGTGCC
TCAGCCACGGCGATCTCGAGAGCCTCCTGGCTCAGATTCTCTACTGCCGGAACCTCAGGATGGACAGCAA
CCAGTTCCAGGACAGTGTGATGGAACTCCTGGCGAGCGTGCTCAGTGGGAAGGACTGTCTGATTGAGAAG
CTCAGCCTGGCAGAGAACGAGATTAGCAACAAAGGGGCCAAAGCTCTGGCCAGATCTCTCATGGTCAACA
GAAGTCTGACTACACTAGACCTCCGTGGTAACTCCATCGGGCCTCAAGGAGCCAAAGCCCTGGCGGATGC
TCTGAAGATTAACCGCGTCCTGATGTCTCTGAACCTCCAGAACAACAAGATAAAGGATGACGGCGCCAAG
GCCTTAGCTCATGTCCTTTCTACTAATAACACGCTCTCCGTTCTCCACTTGCAGAAGAACTCCATCGGGC
CCATAGGAGCCAAGACGTTAGCCAGTGCCCTGAAGCAGAACCGGACTCTGAAAAAGCTCATGTTCTCCGG

CAACGGATGTGGAAATGAGGGGTCGGAAGCCCTGGCGGAGGCCCTGAAACTGAACCAAGGCCTCATCACC
CTGGACCTGCAGAGCAACTCCATCAGTAACGCTGGGATCCTGGCCCTGACCCAAGCCCTCTGCTCTAACA
AGACTCTGATCAACCTGAACCTCCGGGAAAACTCCATCGGTTCAGAGGGGGCACGTGAGATCGCCAGCGC
ACTCCGCGCCAACCGGACCCTGAAGGACCTGGACTTGGCAGCAAACCTTCTCCGCGAGGAAGGAGCCCAA
GCTCTTGCCGCAATTCCTCGATGGAGATGCTTGGGGAGAACGCTGGGTAGAATCCAAGCACAAGTCTGAC
TTCGGCAAGTTTAAACTGACGGCTGGCAAATTCTACGGCGATGCTGAGAAGGACAAAGGACTGCAAACGA
GTCAGGACGCCCGGTTCTACGCCTTGTCTTCCCGTTTTGATTCCTTCAGTAACAAGGACCAGACACTGGT
GGTGCAGTTCACGGTGAAACACGAGCAGAACATTGACTGCGGCGGTGGTTATGTGAAGCTGTTCCCCTCT
GGCCTGAACCAGGCTGACATGCATGGGGACTCCGAGTACAACATCATGTTTGGCCCAGACATCTGTGGCC
CTGGCACCAAGAAAGTCCACGTCATCTTCAACTATAAGGGCAAGAACGTGCTGATCAATAAAGACATCCG
TTGTAAGGATGATGAGTTTACCCACTTGTACACACTGATCGTGCGGCCGGACAACACCTATGAGGTGAAG
ATTGACAATAGCAAAGTGGAGTCAGGGACGCTGGAGAGTGACTGGGACTTCCTGCCCCCGAAAAAGATCA
AGGACCCTGAGGCCAAGAAGCCAGAGGACTGGGATGAGCGGGCCAAGATTGATGACCCTACAGACACTAA
ACCAGAGGACTGGGAGAAGCCAGAACACATTCCTGACCCCGATGCCAAGAAGCCAGAGGACTGGGATGAG
GAGATGGATGGGGAGTGGGAACCACCTGTCATCCAGAACCCAGAATACAAGGGTGAGTGGAAACCTCGAC
AGATTGATAATCCTGACTACAAGGGCAAGTGGGTCCACCCTGAAATTGACAACCCCGAGTACACCCCTGA
CTCCAACCTGTATGCTTACGACAGCTTTGGTGTTCTGGGCCTGGATCTTTGGCAGGTCAAGTCTGGAACC
ATCTTTGACAACTTCCTCATCACTAACGATGAGAAATACGCAGAGGAGTTTGGGAACGAAACCTGGGGTG
CCACCAAGGAGGCAGAGAAGAAGATGAAGGAGCAGCAGGATGAGGAACAGCGGAAGAAACAAGAGGAAGA
AGACAAGAAGCGGAAAGAGGAGGAAGGGGAGGATGAGGTGGATGGGGATGATAATGAGGAGGAGGATGAA
GATGAAGAGGAGGAGCCTGAGAAAGAGGAGGAGGAGGAAGAGTCAGAGGGGCAGCCCAAGGATGACAACA
GCAAGCAGAAGGTGCTGGTGATGGAGTACTGTGCCAGAGGGAGCCTGCTGACCGTGCTGGAGGAGCCCGA
GAATGCCTTCGGCCTGCCCGAGGAAGAATTCCTCGTGGTGCTGCAATGTGTGGTTGCTGGCATCAACCAC
CTCCGAGAGAATGGCATCGTGCACAGGGACATCAAGCCAGGAAACATCATGCGTCTGGTGGGCGAAGCCG
GGCAGAGCATATACAAGCTGACGGACTTCGGCGCGGCCCGGGAACTGGCTGACGACGAGAAGTTCGTGTC
GGTCTACGGCACGGAAGAGTATCTCCACCCAGATGTATATGAGAGAGCCGTGCTGAAGAAGCCCCAGCAA
AAGGCTTTTGGGGTGACGGTGGACCTTTGGAGCATCGGAGTCACCTTCTACCATGCGGCCACAGGCAGCC
TCCCCTTTGTGCCCTTCGGTGGGCCCCGGCGGAACAAGGAGATCATGTTCCGCATGACCTCGGAGAAGCC
GGCCGGGGCCATCGCGGGCGTCCAGAAGCGGGAGAGCGGACCGCTGGAGTGGAGCTACGACCTCCCCACC
ACCTGCCAGCTGTCTGGAGGGCTGCGGGCCCAGCTGGTGCCTATCCTGGCCAACGTCCTG

>Chelonia mydas tlr3-tlr8-nlrc3-calr-ikbke
CTTTCAAAAACCTGAAGAATTTGAACATACTGGATGTATCTCATAATCGTTTGCTTTCTACAAAATTAGG
ATCCCAGCCACAGTTGGAGAGCCTTCGTGAGCTTGTGCTGTCAGAGAATAAAATCACTGAGTTAAAAAAG
GAAGAACTAGATTTTCTTAGCAACGCTTCCTTAAATAGGCTTGACTTATCATCAAACCCACTGAAAGAGT
TTCACACAGGCTGTTTACATGCAATTGGAAATCTGTATGGCCTTGTGCTGAACAATGTAGAGCTTGGTGA
AAATCTCACAGAGAAACTTTGCTTGGAATTATCAGGCACAAGAATTCAGAATCTGTCACTGAGTCAGATC
CAGCTTTCTTTTATTCACAAGTCAACCTTCCACGGACTTCAAACAACAAACCTCACAGCTTTAAACCTTT
CCAAAAATTATTTGAACATGATAGAAAACGACTCTCTTACCTGGCTTTCAAGCTTAGAGAATTTAAACCT
AGAGGATAATAGGATTTATCACTTATTTTCTCATTCATTGTATGGATTATCCAATGTCAAATATTTGAAT
CTGAGAAGATCACGTGTCAGAAAAATTGATGATTTCTCCTTTCGTTGGCTAAGCCATTTAGAGTATCTTC
TTATAGATAGTAATAGTTTTCAAGAAATTACTCCTAATATGTTCACAGGCTTGGACAATCTGAAATATTT
GAGTCTATGTAACTGGACCAATGGCTTACAAATAATAACTAATAAAACATTTTCATCACTTGCTAATTCT
ACTCTGCAGTTTCTTAATCTTACAAAAAGTAGAATCACAAAAATAGAAGGTGGAGCATTTTCTTGGTTGG
GACACCTAAAAATTCTTGATTTGGGACTCAATGAAATAAACCAGGTGCTCACAGGTCATGAGTTTAAAGG
TCTGCAAAATATTGAGGATATCTACCTTTCCTACAACAAAGAGTTGACTTTGACAAGCGAATCATTTGCT
TTTGTTCCAAGCCTTAGAAAACTGATGCTACGGAAGGTAGCTTGTAGCAATCTGGACCTCTCTCCTTCAC
CTTTTCACCCTCTACGGAATCTAACCATCCTGGATATCAGCAACAACAATCTAGCTAACGTAAAAGATGA
TTTGTTTGATGGACTTCACAAACTTGAAATTCTGGATTTGCAACATAATAATTTGGCTCGACTTTGGAAA
CATGCCAATCCAGGTGGCCCTGTCCTTTTTTTAAAAGATCTTCTTAACCTGCATATACTTAATTTGAAGT
CTAATGGCTTGGATGAGATTCCAGTACAAGTTTTCAAGGGCTTGTTTCAATTAAGAAGCCTGGATTTAGG
ATCAAATAATTTGAACTTGCTTCCAGCATCTTTGTTTGATGACCAGATATCTCTGAATTCATTAATCCTT
CAGAAAAATCTTATAACATCTGTTGAAGAAAAAGTGTTTGGTTCAGCTTTCAAGAATCTGAAAGAACTAG
AGATGGATTCCAATCCATTTGACTGCACCTGTGAAAGTATCTCCTGGTTTGTTAATTGGCTTAATGTGAC
CCAAACAAACATACCTGGATTAGATACTCATTACCTTTGCAATACCCCACCTAAATATCATAGTACTCTG
GTGATGTATTTTGACATTTCACCCTGCAAAGATAGTGCCCCTTTTCAATTACTGTATATAATAAGCACCA
CTGTGATAATGCTCTTCATCTTTATTGTCATTCTTATCCATTTTGAAGGCTGGAGGATAGCATTTACTGG
AATGTGTCAGTAAATCGAGTACTTGGTTTTACAGAAATAGACAGGCAACAGGAAGAGTTTGATTATGATG
CCTACATTATTCATGCAAGAAGGGACAGGAATTGGGTGTCCAAGAACTTCATTCCTCTGGAAGAAAATGA
TCAGTCTCAAATTAGGTTTTGTTTAGAGGAACGAGACTTTGAAGCAGGCATATCTGAATTTGAAGCCACT
ATTAATAGTATAAAAAGAAGTCGGAAGATTATCTTTGTTGTCACTGACCATCTCTTAAAAGATCCCTGGT

GCAAAAAGTTCAAGGTGTACCACGCTGTTCAGCAAGCTATTGAGCAAAGTCGGGATTCCATCATACTGAT
CTTTCTTCATGACATTCCAGATTACAAACTGAATCATGCACTTTGCTTGAGAAGAGGAATGTTCAAATCT
CGTTGCATCTTGGATTGGCCAGCTCAGAAAGAACGGGTCAATGCATTTCATCAGCAATTAAAAATGGCAC
TTAAATCTAGCAGCAACATACATTGATTCCTTCACCAGCTTTGTACCTCATATGGCACAGCCTTGGATTT
GAGTTTAAACAATATCTTCTTCATTAACCCAAATCAGTTTAAAGGCTTTGGGGATATAGCTTGTTTGAAT
TTGTCTTCAAATGCCCTTGGCCAGGCTTTCAATGGCACTGAATTAATCTATTTATCTAATCTCAAATATT
TAGATCTCTCATTTAATAAACTGGATTTGACCAGTTCCTCTGCATTTAAAGAACTACCTAACCTAGAGGT
ATTAGATCTTAGCTATAACAAGCACTATTTTCTAGTGACAGGTTTTGCACATCAGCTCCTATTTACTGAA
AACCTTCCTAACTTAAAAATTTTAAATTTAAGCTGGAATGACATTTCTGCCCTAACAAAATTTGAACTAA
GGAGTGACTCCCTTCAAAAACTAGACTTCAAAGGAAACCGTCTTGATATCTTATGGAAAAATGGAGAAAT
GAATCACATACAATTGTTTAAGCATCTAAAAAAGCTGACACATCTAGATATCTCATACAACAGACTTCGA
AATATCCCCACTAGGGCTTTCCAAAATCTGCCTCAGAGCTTAACTAAATTGTATATAAATAACAACAAAA
TACATACCCTCAGCTGGGAAAATCTTAGATACTTTAAGTCTCTGAAGTTGCTTGACTTAAGTCAGAACAA
ACTGAAGGCTGTTGATATCCAGTACAACTACACACAGTCCCTCCAGACTCTGCTGCTGAGGGAGAACAAG
ATTTCCAGGATTGTTGTTGGGTTGCCTGAGAGAGTAAGCAGCCTCCTGTACCTGGATTTGAGTTATAACG
AACTGCAAGTCTTAAATCAGTCAACTTTCTTATCAGGACTTATACAACATTTGAAGGTTTTAAAATTAAA
AGGGAATCCGTTTGACTGCACTTGCAAAAACAGCAACTTCATAAGATGGATACAGAAAACCAAAACTCCG
ATCTCACAAGTAGCCAGAAATGTCATTTGCATGAACCCTGAGGACCAAAGGCAGCATAGCGTTCTCTTAA
TTGACCTGCATGCTTGCATTCTGGATAGCGTTGCAAAAATATTATTTTATATTTCTTTCTCCACTATTAT
TAGCATTATGATGGTAGCAGTTACTAAACATTTATTTTATTGGGATGTCTGGTATACTTATCATTCTTGT
ATGGCAAAAATAAAAGGATACAAATCTATAACCACAGACAAAGCTCTCTATGATGCTTACATCGCCTATG
ATACTCAGGATGCAACAGTAACTGACTGGGTAATAAATGAACTACGATTTCGTCTAGAGGAAAACGGAGA
AAAGCACGTTCTACTTTGTTTGGAGGAAAGGGACTGGGAGCCGGGAAAGGCTGTCATTGACAACCTTGCA
CAGAGCATCCATCACAGCAGAAAGACCGTCTTTGTTCTAACCGAAAGATATGTGAAAAATGGGAACTTCA
AAACCGCTTTTTATATCGCTCTGCAGAGGCTAATGGATGAGAATACAGATGTGATTGTGTTCATTCTGCT
GGAGCCGGTGCTACAGCATTCCCAGTACCTGAGGCTGAGGAGGAGGATCTGCAAGAGCTCTGTTCTTGAC
TGGCCTAAGAATCCACACGCTGAAGGCCTTTTCTGGCAAAGACTAAAAAGTGCAGTGCTAACGGATAACA
GCATGCGAGATGATGGGGTGTACCAAGGCTAACAGATCTTTGTACACAATGTGTACGGTTCCTGCCTTTT
GCTGGATATCTGGCTCCTCAATGGGCTATTTCCTAAAGAACAGCACTGATCAATCCCAAGAAATGACAAC
TGTTCCAAAGACTTTATCAGAAATCTACTCCTATTACTTTAAAATGGCACTGAGTAGTGACTGGCAGGAG
AAACAGAAGGAGACTCCCAGAATAGAACAAGCTATAAACAACAGCAAGAAAATTATTGGTAACCTGGGTC
GGCTGGCATTTTATGGTCTGCTCAAGAGAAAGTACGTATTTTACGAACAAGACTTGAAAGCCTATGGCAT
AGACCTCTCATCACTACAAGGCAGCTTGTGCAGTAGGATGTTACTCAAGGAAGAGATGCAGTTCTCCACT
GTCTACTATTTCTCCCATTTAACCCTCCAGGAGTTTTTGGCAGCCACTTATTACTATGCTTCAGCAAAGC
GAGCAATATTTGATCTCTTTACTGAGAGCGGGATGTCCTGGCCTAAGTTTGGTTTCCTCAATCACTTTAA
GAACGCTGTCCAGAGATCACTGCAGTCAGAGGATGGGCAGCTGGATATCTTTGTGCGCTTTCTCTCTGGC
CTTCTCTCCCCACAGGTGAACAAATTGCTGTCTGGTTGGCTCTTGGTGAAGGATGAGCATAACAGCTTCC
GAAGCCACATGATCAGCTTTCTCCAGAGCTGCCTGAACACTGACTATGTCATCTCCTCCAGGACCGTGAA
CATCATGCAGTGCTTGTATGAAGTGCAGCACATGGAGCTGGCCAAGACAGTGGAGGAAGCTATGAAAAAC
GAGAGCTTGGCTGGGATGTTAACACCAGTGAATTGCTCTGCCCTGGCTTATCTCCTGCAGGTCTCTGAGG
TGTGCATGGAGGAGACAAACCTCTCCAACTGCCTCACCTACAATATCTGCAAAAGTCTGCTTTCCCAGCT
CCTCTTCTGCAGTAATCTCCGGCTGGACAACAACAAGTTTAAGGACAACGTGATGGAGCTGTTGGGGAGC
GTGCTGAGCGTGAAGGATTGTCAGATTCAAAAGATGAGCTTGGCAGAAAACCAGATCAGTAACAAAGGGG
CCAAAGCCCTTGCCAGATCTCTGATGGTCAACAGGAGCCTGACGGTACTTGACCTTCGCAGTAACGCCAT
CGGACCCACAGGGGCAAAAGCACTGGCTGATGCACTGAAAATAAACCAAGTCCTACTCTCCCTGAACCTC
CAGAATAACGTGATCAAGGAAGATGGTGCTAAATTCCTGGCTGAGGCCCTTTTAACCAACCACAAGCTGA
CAACTTTGCACCTTCAGAAGAACTCCATTGGGCCCCAAGGTGCAAAGAAAATAGCAGAAGCACTGAAGCA
GAACGGGAATCTCAGGGAACTGATCCTCTCCAGTAACTGTGTGGGGGACACTGGCTCTGTAGCCTTGGCC
GAAGCTCTGAGAGTGAATCACAGTCTCACAACCCTGGACCTCCAGAGCAATTCGATCAGTAACGCGGGGG
TCACGGCACTGATAGGAGCGCTGTGCTCCAACCGGGGCCTCATCAGCCTCAACCTTCGGGAGAATTCCAT
TGGCCAAGAGGGGGCGCCGGAGATAGCCAACGCTCTGCGCGCTAACCGCACGCTGAGGAACCTAGACCTA
GCGGCGAATCTGTTGTATGATGAAGGTGTCAAGGCTATTGCAGAAGCGCTTAGACCTGGAAAGGGCTAAG
ATGGATGCCTGGACGAGCCGCTGGGTGGAGTCGAAGCACAAGTCGGACTATGGGAAGTTCAAACTCACGG
CGGGCAAGTTCTATGGGGACGCCGAGAAGGATAAAGGCCTCCAGACGAGCCAGGACGCCCGTTTCTACGC
CCTGTCTGCCCGCTTTGACCCCTTCAGCAACAAGGACAAGACCTTGGTGGTGCAGTTCACGGTGAAACAC
GAGCAGAATATTGACTGTGGTGGTGGTTATGTCAAGCTCTTCCCCTCCAGCCTGAGCCAGGAGGACATGC
ATGGGGACTCGGAGTACAACATCATGTTTGGCCCTGACATCTGTGGCCCAGGCACCAAAAAAGTGCACGT
GATCTTCAACTACAAAGGGAAGAACGTCCTGATCAACAAAGACATCCGTTGCAAGGACGACGAGTTCACC
CACCTCTACACCCTCATTGTGCGGCCTGACAACACCTACGAGGTGAAGATTGACAACAACAAGGTGGAGT
CGGGCAGCCTGGAGGAAGACTGGGACTTCTTGCCTCCCAAGAAGATCAAGGACCCTGAGGCCAAGAAACC
AGAGGACTGGGACGATAGGGCCAAGATAGACGACCCCGAGGACACTAAGCCAGAGGATTGGGATAAGCCG

GAGCACATTCCTGACCCCGATGCCAAGAAGCCGGAGGACTGGGATGAGGAGATGGACGGGGAGTGGGAGC
CTCCAGTGATCCAGAACCCAGAGTACAAGGGCGAGTGGAAACCCCGCCAGATAGACAACCCTGACTACAA
GGGGAAGTGGGTGCACCCTGAGATCGACAACCCAGACTACACCCCTGATCCCAGCCTGTACTCCTACGAC
AGCTTCGGCGTCATCGGCTTGGTCCTGTGGCAGGTGAAATCCGGCACCATCTTCGACAACTTCCTGATCA
CGGATGACGAGAAGTTCGCAGAGGAGTCAGGCAATGAGACCTGGGGGGCCACTAAGGATGCTGAGAAGAA
AATGAAGGAGCAGCAGGACGAGGAGCAGAGGAAGAAACAAGAGGAGGAGGACAAGAAGCGGAAAGAGGAG
GAAGGGGACGATGAGCCCGAAGGGGACGATAATGACGAAGAGGAGGAGGAGGACGATGATGAGCCAGAGA
AGGAGGAAGAGGAGGAAACGGAGGCACCGCTGAAGGACGAAAACAGCAAGCAGAAGGTGCTGGTGATGGA
GTACTGCTCATGTGGGAGCTTGCTGAGCGTATTAGAAGATCCAGAGAACTCCTTTGGCTTGTCTGAGTCA
GAGTTCCTCATCGTGTTGCATTGTGTTGTGGCCGGCATGAACCACCTTCGTGAGAACAGCATTGTCCATA
GGGACATCAAACCAGGAAATATCATGCGGCTGGTGGGGGAAGACGGGCAGAGCATCTATAAGCTCACGGA
TTTTGGGGCCGCTCGAGAGCTGGACGATGACGAGAAATTTGTGTCTGTCTACGGGACAGAGGAATATCTT
CACCCGGACATGTACGAGCGAGCAGTTCTGAGAAAACCCCAGCAAAAGGCTTACGGGGTGACTGTAGATC
TGTGGAGCATTGGGGTAACCTTTTACCATGCTGCTACAGGCAGTCTCCCATTTATCCCCTTCGGTGGACC
TCGCAGGAATAAAGAAATCATGTATAAAATAACGACGGAGAAACCTGCTGGAGCTATCGCAGGCGTCCAG
AGGCAGGAGAACGGGCCCATTGAGTGGAGCTACGAGCTGCCCATCACCTGCCAGCTGTCTGTGGGGCTGA
AGAGCCAGCTCATCCCCATCCTGGCTAACATTCTG

>Latimeria chalumnae tlr3-tlr8-nlrc3-calr-ikbke
CCCCTCCATTGTTTCAGAGATTAAGTGTGTTAGATATTTCCCGCAATACACTGACATCTGCAAAGCTAGG
AAGTGAACAGCAACTACAGAATCTTTGGAGCCTTGTGCTATCTGCCAACAAAATCAAAGTATTAAAAAAG
GATGACTTATCGTTCCTGAGTAACACTTTCTTAAAGCAACTTGATCTTTCCTTAAACCCACTGAATCAGT
TTGAACCAGGATGTCTGCATGGAGTGGGAACTTTAGGAGAACTAATTATGGACAACAGTTCTCTAAGCGC
TAACCTCACAGAGAGACTCTGCTTAGAATTATCAGGAACAGAAATCAAGAGCCTATCCCTAAGCAATAAT
AGGCTTTCAAAAATACTCAACATTACCTTCAGAGGATTGCAAGAAACCAACCTAACAACTCTTATTCTTT
CGCAAAATGGATTGTCAGAAATAGAGAATGGATCATTTACCTGGCTTCATAATTTGACCCACTTAATTCT
GGAAGATAACAGAATTGCACACGTAAAAACTGGGACATTTTTTGGCTTGTCAAATTTAAGATACTTAAGT
CTGAAAAAATCCCTGAGGGCAAAACTTGGTTCCAAATATGAAAAGGCACATATAGATGATTTATCTTTTC
AGTGGCTTAATCTCTTAGAGCATCTCAACATGGAGGAAAATATTTTTTCAGCGATTACAACTAATACATT
TATAGGCTTAAAAAGTCTGATGGATCTTAGTCTATGCCACAGCTCCAGTGACTTGAAAGTTATTACCAAT
GCAACCTTTTCATCTTTAGCTATGTCACCTCTTGCTTTTCTTAACTTATCAAGTACTGGAATCTCAAAAT
TAGAGAGTGGAGCCTTTAACTGGTTTAAAGTGCTGAAGAAACTGTACATAGGCTTTAATGAAATTAGCCA
AACCCTATCTGGGTATGAGTTTAAAGGCTTAGACCATATTGAGGAGATATACCTTTCCAACAACAAAAGG
CTGACCTTGACTTCATTTTCTTTTCACTATGTCCCAACTCTTACAATACTAATGCTTGGAAAAACACAAG
TTGCAAACCTGAACATCAATCCTTCACCTTTTCAGCCTCTCCAGAACCTCCGAATACTTGATCTTAGCAA
CAACAACCTTGCTAAAATAAATGATGATTTATTCTCTGCACTTAAGAATCTTGAAATTTTAAAGATGCAG
CATAATAACCTCGCTCGCCTTTGGAAGCATGTGAATCCTGGAGGCCCAGTACTATTTCTGAGAGGCCTGC
AGAACCTGCAGGTACTTCGGTTAGATTTTAATGGTTTTGATGAGATTCCAGTGAATGGGTTTAAAGGCTT
GTCTCAACTAATGGATTTGAATTTAGGCTCAAATGACTTGAATGTTCTTCCAAAAGGTGTTTTCAGTGAT
CTGACATCACTCAGGACACTGGAGCTTCAGAAAAATCTTATAACCTTTATTGATGAAGAGGTGTTTCATC
CAGTTTTTGTGAACTTGGCAGCCCTGTATATGGGGTTCAATCCATTTGACTGTACTTGTGAAAGCATTTC
TTGGTTTGTTAGTTGGCTCAATGTGACTAACACAAGCATTCCTGGGTTGGAATTCCAATATATTTGTAAC
ACCCCACAAAAATTTTACAATACTTCAGTGATGCTCTTTGACACTTCTCCCTGTGCACCTTTTATAATTT
TGTTCAAAATAAGTACAAGCTTCATCCTTATTTTCTTGTTTGCAGTGGTTTTTATTCAGTTTCAAGGCTG
GAGGATAGAATTTTACTGGAATATTGCAGTGAACAGAGTTCTAGGATATAAAGAGATAGATAGACTTGAA
GTCCAATATCAGTATGATGCTTACATTATTCATGCAGAGAAAGACATTGGCTGGGTGAACAGAAATATAG
TACCTCTTGAAGAAAATGACCAAACCACCTTCCACTTTTGCCTTGAAGAACGTGACTTTGAAGCTGGCGC
CTGTAAGTTAGAAGCCATTGTCAATGGCATAAGGAGAAGCAAGAAAATAGTATTTGTTGTAACTCGAAAG
CTTTTGGAGGACCCCTGGTGTAAAAGGTCAAGGTTCAAAGTGCATCATGCTGTACAGCAAGCAATTGAAC
AGAGCCATGATTCCATCATCCTGGTTTTTCTTGAGGATATCCCAGATTATAAACTAAACCACGCCCTCTG
TCTTCGAAGAGGAATGTTCAAATCACGCTGCATTCTGTCTTGGCCAGCACAGCGAGAGAGGGTGAATGCT
TTTCATCAGAAATTTAAAGTGGCACTTGGATCAAGTAACCGGGAACATTAATCATTTTGGCCTCAGTGCA
GTTCATATGGGAAGACACTGGACTTAAGTAAAAACAGCATTTTTTTTATCAACCCAGAACAATTTAAAGG
CTTTGATGACATAGCATGTTTGAATCTGTCTAAAAATGCAATTGGAGATGCTTTAAATGGTACAGAGTTT
GTTCACTTATCAAATCTGAAATACTTAGACATTACAAACAATAAGATTGACTTAGCTTATGATTATGCAT
TTGTGGAATTACAAAAGTTAGAGGTGCTGGATCTAAGTTATAACTACCATTATTTTGATGTAGAAAGTGT
AACACACAAACTGGGATTCATCCAGCATCTTCCAGTCCTAAAAGTGTTAAATTTAAGTTACAATGAGGTA
TTTACATTAACAGAGTCTCAAATTATCAGTAAATCTTTAAAAGAATTTGTATTTAAAAATAGGCTTGATA
TCCTGTGGAAAGACACAGATTCTAGGTACATACATATTTTCAAAAATCTAATCAATTTGACACACCTTGA
CATAAGACAAAACAGGCTTAAAAACTTTCCACAAGAAGCATTTTTGAACCTGCCAAAAAGCCTAGTGAAG
TTGTATATAAGTAATAATTATTTGAAGAATTTTGATTGGCAGAACCTTCGGCACTTCCCCCATCTTGAGT

TGCTTGATCTAGGCTCAAACAGACTGACTGTTATGACTAATAATCTCCACAACCTAACAAAGTCTCTTCA
TACACTAGTGCTAAGGAGAAACAAGATTTCTCATCTTCCTGCTGGTTTTCTTGACAAAGACACTAGTCTT
AAGTACCTTGATTTAAGCTTCAACAAGTTGTTCGTCATCAACCAGTCAACATTCCTGTCAGGGGCAGCAA
ATTACTTGGAGGTGCTGGTTCTCAAGGGAAATCCTTTCCAGTGCACTTGTGAAATTACTGAATTTATAAT
GTGGATACATTCAAACAATGTCATTATCCCCCAACTAGCCACAGATGTCACCTGTGCTACCCCAACAAAT
CATAAGGGCCAAGGCATCATTTATTTTGATCTTCATGCTTGTGCACTGGATAACCTTTCGGCATTATTTT
GTCTCATGTCACTCTTCATTATTATAATCACAATGGTTGTGGTAGTCACAAAGAATTTATTTTACTGGGA
TTATCACTTCTGTGTTGCTCGAATGAAGAAGCCTATAGTTTCAGGAAACAGCATCTATGATGCCTATGTT
GCTTACGACACTAAAGATCCAACTGTCACTGACTGGGTTGTAAATGAACTATGTGTTCATTTGGAGAATG
AAAAAAGAAGCTTTCTTCTGTGCTTAGAAGAAAGAGACTGGGAGCCTGGAATAGCCATTGTGAACAATCT
TTCCCGGAGCATCACTGAAAGCAGGAAAACTGTGTTTGTGCTAACAGACAAATATGTAAAAAGTGGAACT
TTCAGAACAGCATTCTATTATTATTATATGACTCATCAAAGATTAATGGATGAAAACATGGATGTGATAG
TGCTGATTGTGCTAGAACCAGTTCTCCAGCATTCCCAGTATTTGCGGCTCAGGCGAAGGTTATGTGGAAG
TTCTATCCTTGACTGGCCAATAAATCCAGACGCTGAAAGATTTTTCTGGCAATCCCTGAGGAATGTGATA
GCAACAGACAACACAGTTAGATATAACAAAATGTACAAAGAGTCCTAAGGGTAACAAATCCTTCCACATA
ATGTGCACTGTTCCATCGTTTTGTTGGATAGCTGGGCTTTCAATAGGGCATCTTCTTAAAAACAGTTACA
ACTCTCAAGAAGGTGTTACAGTCCCCAAAACTTTGTGTGAACTTTACTCTTACTTTTTCAAATTGGCTTT
AAATGGTGACTGGACAGACAGGGAGAAAGAAACCTTAAAGATAGACCAGACTATCAACAGCAGTAAAAAG
CTGGCTGGAAATCTTGGGAAACTAGCTTTCTATGGTCTAATCAAACGGAAGTATGTCTTTTATGAGCAGG
ACATGAAAACCTATGGAATAGATCTCACGTCACTACAAGGAAACTTGTGCAATAATATCCTACTTAAGGA
TGACTCTCAAACGTTCACTGTCTACTATTTCACTCACTTGACACTTCAGGAATACATGGCAGCCACATAC
TATTACACAGCAGCCAAAAGAGCTATCTTTGATCTCTTTACTGAGAGTGGGATGTCCTGGCCCAAGCTGG
GCTTTCATAACCACTTCAAAAATGCCATCCAGAGATCTCTGCAGTCAGAAGATGGTCAGCTGGATATCTT
CGTGCGCTTTCTTGCTGGTCTTCTTTCCCCGCAAGTCAACAAGCTGCTTTCTGGCTTGCTCTTGACCAAA
GAGGAGCACAATGGCTACAGGGAGCATGTAATTAACCTTCTTCAGGCATGCTTGAACACAAACTACAACA
TATCCTCCAGGACAGTGAATGTCATGCATTGCTTGCATGAACTCCAGCACACAGAAATAGCGCGGACTGT
AGAGGAGGCCATGAGGACCGAAAGCCTTGCTGGAAAGCTGACACCTGTGAATTGCTCCGCCTTAGCTTAC
CTTCTTCAGGTCTCTGAGACCTGCATGGAAGAGACCAACCTCTCAAACTGCCTGAATGATAGTGTCCTCA
AGAGTTTACTGTCTCAGCTTCTGTACTGTCATAATCTCAGGTATGTTGCAGATGAGCATTTCAAAGTGCT
TTATGAAGCAAAAGGCAAAGTGTTAGAGTACAAGGCTTGCAAGCTCCAAAAAACAAATTTGATTTACTGC
CATGTCTCAAACACTTTTTTGTTGTTGACACTTCTTGTCAATAAAAGCCTCTCCTGTCTGAACCTGCGTA
ACAATGGTATTGGACCCAGGGGTGCAAAAGCATTGTCTGATGCTCTGAAGATGAACCAAGCCCTAGTATC
ACTAAACCTCCAGAATAATCAAATAGGAGAAGAAGGAGCAAAATGTGTAGCTGATTTCTTGCAAGTCAAC
CGCAGACTAACCACCTTGCACCTCCAGAAGAATTCAATAGGCCCTGAAGGAGCGAAACGCATGGCAGAAG
CACTGAAAAACAATTGCAGCCTCAAGGAATTTATACTCTCCAGTAACTGTGTTGGAGACAAAGGAGCAGC
AGCATTAGCCAAAGCATTGAAAGTCAACAAGAGTCTTACAACCCTTGATCTCCGGAGTAATTCCATAAGC
AACACAGGTGTTACAGCATTGACGGAGGCGCTTAAATGCAACCAAGGACTAATTGACCTTAATCTTCGAG
AAAATTCTATTGGCATTGACGGTGCAAAGGAAATAGCTAATGCACTACGTGAAAACTGTACCCTGAAAAA
TCTAGATTTGACAGCTAATCTTCTGCATGATGAAGGAGCAAAAGCCATTGCACCGGTTGGAGGATGGAGC
TGGCTGGGAGAAGAGGTGGATTGAATCAAAGCACAAATCTGACTATGGAAAATTCAAACTGACTGCTGGG
AAATTTTATGGAGATGCAGAAAAAGACAAAGGTCTCCAGACCAGTCAGGATGCCAGGTTTTACGCCGTCT
CCTCACGCTTCGAGCCCTTCAGTAATGAGGGGAAAACGCTGGTCATTCAGTTTACGGTGAAGCATGAACA
AAAGATCGACTGCGGTGGGGGGTACGTCAAGATCTTCCCTTCCGACTTGGACCAGGAGAACATGCACGGG
GATTCTCAGTATTATATTATGTTTGGGCCTGATATTTGTGGTTACAGCACCAAGAAAGTTCATGTAATTT
TCAACTACAAAGGGAAAAACCACTTAATCAAGAAGGAGATTAAATGCAAGGACGATCAGTTAACTCACTT
GTATACTTTGATCCTGCGTCCGGATCAAACGTACGAGGTTAAAATTGACAATGAAAAAGTGGAATCTGGC
AGCTTGGAGGAAGACTGGGATTTCCTACCACCAAAGAAGATTAAAGATCCTGAAGCCAAGAAACCTGAAG
ATTGGGATGATAGAGCCAAAATTGATGATCCTGAGGATGTGAAGCCAGAGGATTGGGAGAAGCCGGAAAA
CATCCCTGACCCGGATGCCAAGAAACCAGAGGACTGGGATGATGAGATGGATGGAGAATGGGAGCCTCCA
ATGATTCAGAATCCAGAATACAAGGGTGAGTGGAAACCCAAGCAGATTGACAATCCAAACTACAAGGGGG
TCTGGGTTCACCCCGAGATCGACAATCCGGAATACACCCCCGACCCTGTGATCTACAAATTTGACAACAT
TGGAGTACTAGGACTTGATCTCTGGCAGGTCAAATCTGGCACCATCTTTGACAACTTCCTAATCACAGAC
GACGAGACGTTTGCTGAGGAAGTTGGGAAAGAGACCTGGGGAGCCACCAAGGAGCCAGAACAGAAAATGA
AGGAGGAACAAGATGAAGAAGAGCAGAAGAAGCATGAGGAAGAGGAGAAGAAAAAGGAGGAAGAGGAGGA
TGAAGATGACGATGAAGGAAAGGAGGAGGAAGAGGAGGAGGATTCGGAAAAAGAAGCTCCGACTAAAGAT
GAATTATAAAACTTTGGGGGGGGGGGGATTGCAAATCTAAACTTGTAAACACTAAACAGAAGGTGTTGGT
AATGGAATACTGTGCGGGAGGAAGCCTGTTGAACGTTCTTGAAGAACCGGAGAATGCTTTTGGCCTCTCG
GAGTCCGAATTCTTAATAGTATTAGAAAGTGTCGTTGCTGGAATGAACCACCTTCGGGAGAACGGGATAG
TCCACCGAGATATTAAACCTGGGAACATCATGCGTCTGGTGGGGGAAGAAGGACAGTCTATTTATAAACT
CACAGATTTTGGAGCAGCACGAGAGCTGGACGATGATGAGAAATTCATTTCTCTCTACGGGACCGAAGAG
TACTTGCACCCCGATATGTACGAGCGGGCTGTTCTTCGGAAACCCCAGCAGAAGACCTATGGTGTGACGG

TAGACCTCTGGAGTATCGGGGTGACCTTTTATCATGCAGCTACAGGCAATCTCCCCTTCATCCCCTATGG
AGGACCTCGTAGAAACAAAGAAGTCATGTATAAAATAACAACTGAAAAACCATCTGGGGCCATCACAGGA
GTCCAGAAGCGGGAAAATGGTCCAATCGATTGGAGCTTTGAACTGCCAATCACCTGTCAGCTTTCTGGAG
GTCTGAAGGCCCAGCTGGCGTCCATTTTGGCTAATATCCTG

>Astyanax mexicanus tlr3-tlr8-nlrc3-calr-ikbke
CCTTCTCCGTTTTAAAGAGTTTGACGTGGTTAGACGTGTCTCGCAACAAGCTGATCACTGCTGGCCTGGG
CACCAAACCTCAGCTGCCCAGCCTGGTGACCCTCATCCTCTCCGGGAATGACTTCACAACTCTTAAGACG
GATGATTTCTCTTTCCTTAGCAGCTCCCCTTCGTTCCGTGTGCTGGGACTTTCCTCCCTGCCTCTAAAAA
AAGTGGAACATGGCTGCTTCAAACCCGTTTCTGCACTCACAGACCTGGTGTTGGATGGTTGCAACCTCAA
CACTCTCCTCACCAAAGTCTGTGATGAGTTGTCGGACACTTCTGTAAGAAATCTCACCCTCCGCAGCACT
CTGCAAACCGTGCTCAAGAACATCACCTTCAAAGGCCTGCTCAAGACTAACCTCACCATGCTGGACCTCT
CCAGCAACAAAATATCTAAAATCGATGACGGCACTTTCCAGTGGCTTCCCTTGCTGGAGTCCCTCTCTTT
AGAGAACAACAATATTAAACACCTGACCAAAGATACCTTTATGGGTCTAAAAAGTGTAACGCAACTCAAC
TTGCAAAAGGCACTGGTTAAAACCAAGGCCGCCTATCCGATAATAGATGACTTCTCCTTTCAGCCATTGG
TCAAGCTAGAGCATCTGTTTATGAGGGACACTTCCTTTAGGGACATTACTGAGAATATGTTTGCTGGACT
ACCATCTCTTAGGACACTAGACCTGAGTTGGAGCAAAGCAGGGCTTAAGACTATCAGCAATACCACATTC
GCCTCCTTGCAAGGGTCTCCACTCCAGGTGCTCAATCTTACAAGAATGGCCCTCACGAAACTTGACCCCG
GCGCTTTCTCATGCCTGGGAAACCTCACCACTCTTGTGCTCAAGTACAACTTCATCTCCCAGGATCTAAC
AGGAGAAGAGTTCCGCGGGCTCAACAGTATAAGGGAAATCTATTTGTCTCTCAACCAGCAGAAGATCAGC
CTAACCCACAGATCCTTCATCCATGTGCCCACACTGAGGGTTCTAATGTTGGGTCGTGCCTTGACTGGCA
CTCTAGACATGGAACCCTCTCCCTTCAGACCACTAACCAACCTCACAGTGCTGGATCTCAGCAATAATAA
CATTGCAAACTTCAACAGTGGCTTGCTGGAGGGACTGTATCATCTCAAGGTGCTGAAAATGCAGCACAAC
AACTTGGCTAGGTTGTGGAAGGATGCTAACCCAGGGGGTCCTGTTCTGTTCTTGAAGGACACTCAGAACC
TCTCTGTTCTGGAGTTGGATTATAACGGTTTAGATGAGATTCCACTCAAAGCCTTGCGAGGCCTTTTCCA
TTTAAATGAGCTCAGCCTCAGTGGGAATCTGTTGAACTACATGCATGGGTCCATATTTAATGACTTGCAG
GCGCTGCGCTACTTGCGAATGGAGAAGAACCTTCTGACGTCAGTGCTGATGGGGACGTTCAGCGTCCCGC
TGTCCAACTTGTCTGAGCTTCACATGGAACACAACCCCTTTGACTGCACCTGTGAAAGCATCCAGTGGTT
TTCTGACTGGCTCAATTCCACCAACACCAGCGTCCCTGAGCGCACCAGCAGCTACATCTGCAACACCCCA
GCAGCGTACTTTAACCGCTCTGTGCTAGACTTTCAGACAAACTCCTGTAAGGACTTGCTTCTCTTCCAGG
GTCTCTATACTTTCAGTAGCACCATGGTGCTTGGTCTAATGGCTATAGCCTTTCTGGTGCACTTCCAGGG
ATGGAGGATTCAGTTCTTCTGGAACATTGCAGTTAATCGTACCCTTGGTATGAAAGGCTCCAGATATGAA
CAAATGGTTGAGGACAGATACGACCACGATGCCTATGTTATCCACGCACCTGACGATAAACCTTGGGTGG
AGCGAAGCTTGCTCCCACTGGAAAATGAAAATTTTAGTTTTTTCCTGGAGGATCGAGATGCAGTGCCAGG
CCAGTCCACTTTGGAGTCTATTGTTGAGAACATAAGAAGGTCCAGGAAAATCATTTTCGTCGTCACAGAG
GCTCTTCTGAACGATCCCTGGTGCAGAAGGTTTAAAGCGCACCATGCACACCACCACGTAGTGGAAGACA
ACCGAGACTCCTTGGTTCTGGTTTTCCTGCAGGACGTCGACGATTACCGTCTGAGCCGCTCACTCCTGAT
ACGCCAAGGCATGCTGAAATCACGCTGCATCGTCCACTGGCCTCTGCAGAAAGAACGCATTGCTGCGTTC
CACCAGAAACTACAGATAGCGTTAAACTCCAGCAACAGGCAGAACTGACTGGTCAAGCCAGAGTGCTTCA
ATTCTGGCCGTGTGCTGGATTTGAGCCGAAACAACATCTTCTTCATTTCTCCCAAGCAGTTTGAAGGACA
TGGTAATATTTCCTGCCTCAATCTGTCCAGAAATGGCTTTGCGGCAGCCCCCAACGGGACTGAATTCACA
GCTTTGCCAAAACTGAAATACCTAGATTTGTCATTTAACAAGATTGACCTTGCCTATGACAATGCCTTTA
AAGAATTGAAGATGCTGGAGGTGCTTGATCTCAGCTTCAACGCACATTACTTCACTGTGCCTGGAGTTAC
TCATAATTTAAACTTTTTGAAGCAGCTACCCAACCTGAGGGTCCTAAATATGAGCTCTAACAGCATTAAT
ACCTTAACGACCAAGGAGATGCACAGCAATTCTCTCAGCGAGCTTCAGTTTCAGCACAACAACCTGGGAA
AGTTATGGAGAGAAAACGACAAAACGTATGACAATCTTTTCACGCATTTGAAAAACCTGACACATCTTGA
CATTTCGTACAACAACCTCAACAAGATCCCAATCAGGGTTTACGATTGCTTGCCGGGGAAGCTAAAGAAA
TTCAGATTGTCTCACAACGGACTGGAAGGTTTGGAATGGACTTTGTTGAAAAGATTTACCCAACTAGAGG
AGCTTATCCTCAGCTTCAACAGCTTAGTGAACATCTCTCAAAACATAAGTCAGAGTGTTCCATACCTGCG
TTTCCTCGACCTGAGCAAAAACAAGATCTCTCAACTGACTGATGACTTTCTCATAGGCGCTGTGAATCTC
CAGTGGCTTGACCTCAGTAACAACAGGCTGGCCATCATCAACCAGACCACGTTTCCTTCAAAGGAATACA
GCCACCTGAGCAGCTTGTGGCTTCATGGAAACCCTTTTCGCTGCACCTGCGACATCTTGAGCTTCATCCT
TTGGATGGACGGCACCAATGTAAAGATCCCCAGACTTGCGACCTCGGTAATGTGCAATATGCCTCAATCA
GTCAAAGGAAAACCGGTGGTAAATTTTGACATCAGGGACTGCATAGATGACCAGGTAGCCTTCCTTATCT
ACTTCTTCACCACTTTCTTCATTATTTGCATTACCTTCACCACCACTGTGATGCACCTGTTCTACTGGGA
CGTCTCCTACTTGTTCTACTACATGAAAGCCAAATACAAAGGATACGAGTACCTGAGTTCCACGGACAAC
GTCTACGACGCATTCGTGACCTACGACACCAAGGACCCGCAAGTATCCGACTGGGTCCTGAACCATCTGC
GGGTTCAGCTTGAGGAACAAGGGGACCGGTACCTTCCTGTGTGCCTGGAGGAGCGGGACTGGGTTCCAGG
AAGCCCCCTGTTAGAAAGCATGACCCAGAGCATCCAGCAGAGCCGCAAGACGGTCTTCGTCCTCACGCAG
AGCTTCGTCAACAGTGGCTCCTTCAAGATGGCTGTGTACCTGGCGCACCAGCGGCTGCTGGAGGAGAGCA
AGGACGTCATCGTCCTGCTCATGCTGGAACCCGTTCTGCAGAGCTCCCACTTCCTGCGACTGCGGCAGCG

ACTGTGCAGTCAGAGCGTCCTCGAGTGGCCCCGGGCTCCGGCTGCAGAACCGTGGTTCTGGCAGTGCCTG
AGGAACGCCATCAGGGTGGAGAACTGTGACATGTACAGCAAGATCTACTCCAGATACGAGTTTGCAGAAA
CCTTTGCGTATCCTGTGCTCTGTGCCTGGCATCTGTCGCATTGTCACAAGGACACTGTCCCGCCTTGTAG
GCTCAGAGTCCGAGGATTTGCTTCTTCCACGAACCTTGACGGAGATATACACTCACTATTGTTGGCCACA
CCTTTCCTCTTCAGACCCTTCCACTGGAAGCATCCGGAAATCACTAACTTCCCTGGGACGCCTGGCCTTC
TACAGCCTCCTACGACGGCGTCACACATTCTGCGAAGCTGAGTTACGGACTTACGGTGTCGATGTGCCGC
CACCACGAGGAACTTTGGGACACAGAGTCCTCAAGCGTGAGCAGAGTTGGTCATCAGAAAGCATCTCATG
GCGCTTCTTGCATACGTCTGTGCAGGAGTTTCTTGGTGCGGTTTTCTACTACATGTCCTCTCGGCGGGGG
ATGTTTGATTTGTTCTCTGAAAGCGGAGTCTCTTGGCCGCGCATCGGCTTCCACAGCCATTACCGTGCCG
CCCTTCAGAAAAGCAGCACTGCCACCAGTGGTAACCTGGACCTTTTCATGTGCTTCCTATCTGGAGTGTT
GTCCTCGGCAACAGGAGCCCTACTGGGCAGTGCTTTGGGCGTAGGCCGGGAGGAGCAAGCGAACCAGCGC
ACCATGGCCATGACCCTGCTTCAGAACACGGTGGCAGGGTCAGGGAGCGAGCCGGTTAGTATGCGCAGTG
TTTCCACAGTGGCCTGTCTGGCAGAGCTTCAGCAGGGGGAGTGGCTTCGGTCTGTGGAGGAGGACCTGAT
TGGCTGTAGGTTGCGGGGAAAGTTGAAGGGAGGGGTTTGTGCAGTGCTGGCGTACCTGCTGCAGGTGTCG
GACGCCTGTGCCGAAGAGACGCACCTCTCAAACTGCTTAGACTCTGCCTCCCTCAAGAGACTTCTTCCAC
AGCTTCTGTACTGCAGCAAGTTACGCATGGAAAACAACGGATTTAAGGATGATGCAATGGAGCTTCTGGG
GAGCTTGCTGAGTGCCAAAGACTGTCACATTCAGTTCTTAAGTTTGGCAGACAGCTCCATTAGCAGTAAA
GGGATCAAACCACTGAGTCGAGCCCTTCTGGTCAATCGAACACTCACTATACTGGATCTTCATGGCAACA
ACATCGGCACCAAAGGAGCAAAGACACTGGCCGAGGCTCTGAGAATGAACCAGATCATTGTGTCGATTAA
TCTTCAGAGTAACAGTATTGAGGATGAGGGAGCTCGAGCTCTGGCAGAGGTGCTGCAGTCCAACCGGAAA
CTCACCTCACTGAATGTTCAAAAGAACGGTATCGGCCCAGACGGAGTGAAGAGAATTGCAGAGTCCCTAA
AAAAGAACCAGATTCTACAAGACCTCAATGTATCAAGCAATCACTTGGGAGACCTTGGCACAGTGGCTTT
GGCACAAGCTCTTGTGGTCAACCATACACTCTGCACTCTCAGTCTCCAGAGTAACTCCGTCAGTGACAAG
GGCATGAAGGCTCTCACTCTGGCCCTGCGCTCCAACAGAGGCCTGACCACACTGAACTTAAGGGAGAACT
CTATAGGAGTTGAAGGTGCCAAGGCCATCGCTCGAGCTTTACAGGAGAACAGCACACTCAGAGAACTGGA
TCTCACTGCTAATCTTCTCCATGACGAAGGAGTGACGGCCATCGCTAAAGTTTTATAAAGAACAGACAAA
TTCTCTTTCAGATGAGTGGAAGAGCCGGTGGGTGAACTCTGAGCACAAGTCTGACTATGGACAGTTTAAA
CTTACATCTGGAAACTTCTACGGGGATGTGGAGAAAGATAAAGGTCTGCAAACCAGCCAGGATGCACGGT
TTTATGCAGCATCCGCCCGCTTTGAGCCCTTCAGCAACGAGGGCAAATCCTTGGTGGTCCAGTTCTCTGT
TAAACACGAGCAGAAGATCGACTGCGGAGGTGGCTACGTCAAAATCTTCCCTTCTGACCTCGACCAGACC
AACATGCATGGAGACTCGCAGTACTACATCATGTTCGTAACAGGGCCTGATATCTGTGGCTACAGCACCA
AGAAAGTTCACGTCATCTTCAACTACAAGGGCAAGAACCACCTTATCAAGAAGGAGATCAAGTGTAAAGA
TGATGAGCTGACTCACCTGTACACACTAATCCTGAATCCGGATCAGACCTATGAGGTGAAGATTGATAAT
GAGAAGGTGGAGTCTGGCTCTCTGGAGGAAGACTGGGACTTCTTGCCTGCAAAGAAGATCAAAGACCCAG
AGGCCAAGAAACCCGAGGACTGGGACGATCGTGCCAAAATCGACGATGAGACTGATACCAAACCTGAGGA
CTGGGACAAGCCTGAGAACATTCCAGATCCAGATGCCAAGAAACCTGAAGACTGGGACGAGGACATGGAT
GGAGAATGGGAGCCACCCATGATCCCTAACCCAGAGTACAAGGGAGAGTGGAAACCCAAGCAGATTGATA
ACCCCAACTACAAAGGAGCCTGGGTGCACCCTGAGATCGACAACCCTGAATACAGCCCAGACGCTGAGAT
TTACAAGTTCGAAAAGATTGGAGTCCTTGGGTTGGATCTCTGGCAGGTCAAGGCTGGCACCATTTTCGAC
AACTTCCTGATCACAGATGACGTACAGACAGCAGATGAATTTGCAAAAGAAACCTGGGGAGTTACTAAGG
GCCCAGAAAAGAAAATGAAGGAAGAGCAGGACGAGAAGAAGCGAAAGGAAGAAGAGGAAAAGAATAAGGA
ACAGGATACTGCACCAGGGGAAGAGGAGGGAGATGAGGAGGATGGCGACGACGATGATGATGAGGAAGAG
GAGGAGACGGAAGAGCCTCCAACTGATGACGAGGACAGCGACGCTGCACCTAAAGATGAGAACCCCAAGC
AGAAGGTTCTGGTGATGGAATTCTGTTCAGGGGGAAGTTTGCTGAATGTGCTGGAGGAGCCAAAGAATGC
CTTTGGTCTGTGTGAATCAGAATTCCTTATAGTGCTACGGTGTGTGGTTCATGGCATAAAACATTTACGT
GATAACGGGGTGGTCCACCGAGACATCAAGCCAGGAAACATCATGAGACAAGTGGGCGAGGACGGACGCT
CCATCTACAAGCTCACAGACTTCGGTGCTGCTCGAGAACTGGAGGACGACGAGACGTTTATGTCCATATA
CGGCACAGAAGAGTACCTGGTGAGACTGGCATTGGATATAACTACTGTATACAGCTCTGGAAAAAAAATA
ACTTTTGGTGGAGTAATTGCCTCTATTGTCCATAAAGAGACTGCACAAGTGGGTTTGGGAGGGCAAAGTA
TGGGGTTCGCAAAATTTTCTGGGGCCTATAAGGCTAAAGATTTAAGGCATAAGATCACGACTGAAAAGCC
TGATAAGGTGATAGCAGGAGTGCAGAAGGTGGAGAATGGAGAGATTGAATGGAGCTACCAGTTGCCTGAC
TTCTGCCAGCTTTCAGAGGGTTTGAAAATGCAGCTGGTGCCAGTTCTGGCCAACATCCTG

>Danio rerio tlr3-tlr8-nlrc3-calr-ikbke
CATTCTCTCTCTTAAAGAATTTAACATGGTTGGATGTATCTCGAAACAAACTGACATCTGCTAAACTGGG
AACAGAACCTCAGCTGCCAAACCTGGTGACCCTGGTTCTCTCTGGAAATAACATTAACATACTGCAAAAG
AATGACTTCTCATTTCTCAGTAATTCTTCTTCATTTCGGGTTCTGATTCTCTCATCTCTGATTCTTAAAA
AGGTGGAGAATGGGTGTTTCAAGGCTATCGATACACTGTCTGACTTGGTGTTGGATAGCAGCAAGCTCAC
CTCTCAGTTTACCACCAGTCTTTTTGAAGAACTTGCTGACACAGCCTTGCGAAACCTTTCTCTTAAGAGC
ACTGAACAGGTGACACTGTCAAACACAACTTTCCAAGGTCTAGAGAAGACCAAGATCAAAGTTCTGGACC
TTAGTGAAAACAGAATTTCAAAGATTGTAGATGGTGCCTTTCAGTGGTTGCCCCAACTGGAATTTTTGTC

TCTGGAGCATAACACAATTAGACACCTAACTAATGACTCATTCAGTGGACTAGGAAACCTAAGGCAGCTC
AACCTGCGGAAAGCCCTAATTAAGAGTCATGCATCTTTACCTGTTATTGATGACTTTTCCTTCCAACATC
TAGTCCAGTTGGAATATTTGTGTATGGCAAATACTGCCTTCCGAGAGTTAACTGAACAAATCTTTTTTGG
CCTTCGGAACCTGAAGACATTGGATTTGAGTTGGAGCATCACAGGGATAAAGACAGTCACAAATAAAACC
TTTGCTGGTCTGCAAGAATCTCCACTCCTTCAGACTCTTAATCTTACAGGCATGGGCATCAACAAGTTGG
AGCCTGGTGCCTTCTCAAGTTTAGGAAATCTCAGCAACCTACTAATGAGTCGCAATTTTATCAATCAGCA
GTTACAAGGGAATGAGTTCAAGGGCCTCTCTAACATTAAAGAGATTGACATGTCTATAAACCAGCAAAGT
ATTTCCCTCACCAATGCATCATTCGTTCATGTCTCTACATTGAGGACCCTAAAGCTAGGTCGTGCTTTAA
AAGGGACTCTAGATATGGAACCATCTCCTTTCAGGCCACTTGTCAACCTCACAATCCTAGATCTCAGTAA
TAATAATATTGCAAACATAAATGCTGACTTGTTGGAAGGTCTGTATAATCTAAAAGTGGTGAAAATGCAG
CACAACAACTTAGCTAGATTGTGGAAAATGGCAAACCCTGGTGGTCCGGTGCTGTTTCTGAAGGATGCTA
CAAATCTGTCGTATCTGAATCTGGATTACAATGGCATCGATGAGATTCCACCTAATGCTTTTCGTGGCTT
CTCTGAGTTGCATGAACTAAGTCTTCGTGGCAATCTCTTGGATCAGTTGCATGCCTCTGTTTTTGATGAC
CTAAACTCTTTAAAATATTTGCATCTTCAAAAGAACCTTATAACATCTGTCCAAAGGGCTACGTTTGGTG
TGCCACTGTCCAAACTGAAAGAACTTAACATGGACCACAACCCATTTGACTGCACCTGTGAGAGCATCCT
GTGGTTCTCTGAATGGCTGAACTCCACCAACACCAGCGTTCCAGGATTTCCTCAAAGTTATATCTGCAAT
ACCCCCAATGCCTACTTTAATCACTCTGTAATGAACTTTGATCCTTTGTCCTGCAAGGACATGACACCTT
TTAAGGCGCTGTACATTTTGAGTAGTACGGCAGTGTTAATGTTGTTGTTTATATCCTTCCTGGTACACTT
CCAGGGATGGAGAATTCAGTTCTACTGGAATATAATGGTAAACCGTATGTTAGGGTCGCTGAAGGATGCA
AAAGTTACTGAAGGTAGATTTCAATTTGATGCCTATATCATCCACGCAGGTGAAGATAAATCATGGGTGG
AACGAAGCTTGCTCTCTCTGGAGGACAAAGATTTAAATTTTTTCTATGAACAGAGAGATTCTACACCTGG
ACATTCTCGTCTTGAGACAATTGTTGATAACATGGTATACTCCAGGAAGATCATTTTTGTTATAACAGAA
ATGCTACTAAAGGATCCATGGTGTAGGCAATTCAAAGCCCATCATGCTCTTCATCATGTAATGGAGGATA
ACCGCGACTCACTGATCCTGATTTTCTTGGAAGATGTAACTGATTACAACCTGAATCGCTCTCTTCATCT
TCGTCGTGGCATGTTGAAGCCTAAATGTGTCCTCTACTGGCCTTTACACAAGGAACGCATCCCAGCATTT
CATCAGAAACTCCGCTCAGCATTAGCCTCTACAAACAAGGTCAACTAGCTCGTAAAGTTAGAATGCTACA
ACACCGGCCGTGTGCTGGATCTCAGTCGAAATAACCTGTTTTTAATTTCTCCTGAACAATTTGACACATA
CGGGAACATCTCCTGCCTCAACCTCTCCAGGAATGGCTTTTCCACGGCTCCCAATGGATCTGAATTTACA
TCCCTTCCCAATCTCAAATATCTGGATTTATCCTTCAACAAAGTCGATCTTGCCTATGATAACGCCTTCC
GAGAGCTACAGAGTCTTGAAGTGCTCGATATCAGTTACAATTCGCATTATTTCACTGTGGCCGGTGTAAC
CCACAACATGATGTTTCTTCAATATCTGCCCTCACTGAAGGTGTTGAACATGAGCTTTAATAGCATCAAC
ACGTTAACAACCAAAACCATGTCAAGCAAGTCTCTTCGAGAACTGCAATTTCGGGGCAATAAGCTCGGAA
GGATGTGGAGGGACAAAGACAATACCTATGTGAAGATTTTTAAAAACTTCACCAACCTCACCCACCTTGA
CATATCCAACAACAGCATTGGGAAAATTCCCTATACAGTTTATACGCAACTTCCCATTACTATTCAACGC
TTGCAAATGAGCCACAATCAACTTGCAAACATTAACTGGACAATGTTGAGGCGTTTCCAGAATCTCAGAG
AACTCATTTTACATGACAACAACATTATTGAAATCGCTAGCAATTTGTCAGTCGATGTTCCCTCCTTAGA
GTTGCTCAATCTCCAGCGTAATCGAATCTCCAAGCTTGCCATTGGATTTCTGCAGGGGGTGGTGAACCTT
AAGGAACTTGATCTCAGTTATAATTATCTTATAACTGTTAACCAGTCTACATTTCCAACTGAGAGTGATC
TGAAGATGCTATGGCTTCACGGAAACCCATTCCACTGCACGTGCAACCTGCTTGAATTTGTTCTGTGGAT
TCTGGATACTAATGTAAAGATCCCTCGGTTGGTGACCGGTGTGACTTGTACCATGCCGGAGGAAAGGAAA
GGTCTGGCTGTGATTAAGTTTGACATTCAAGAATGCATTGATGATCAGCTAGCCTTTGTGGCGTACTTCA
TTTCCGCTGCGTGCATCATATGCACTACTTTCGCAGCCATCACTATGCACTTGTTCTACTGGGACGTCTC
CTACTTATACTACTATCTGAAAGCCAGATTCACTGGATATCAGCAGTTGAGCTCAGAAAGTTGCATCTAT
GACGCCTTCATCACCTACGATACCAAGGACCCGCAGGTGTCAGACTGGGTGCTGAACCACCTGCGTGTGC
AGCTGGAGGAGCGAAGCGAACTCTTCCTCCCGATCTGCCTGGAGGAGCGAGACTGGATCCCTGGCTCCCC
GGTGTTAGACAGCCTGACCCAGAGCATCCAGAACAGCCGAAAAACGGTCTTCGTGCTGACCGAGGGCTAC
GTGAACAGCGGCTCCTTCAAGCTGGCGGTGTTTCTGGCCCATCAGCGGCTCCTGGAGGAGAATGAGGACG
TGATTGTTCTGCTGTTGTTGGAGCCGGTGCTGCAATACTCTCATTTTGTCCGTCTGCGGAGGCGGTTGTG
CGCTCGCAGCATCCTGGAGTGGCCACACTCATCCTCCGCCGAGGCCTGGTTCTGGCAGTCACTCAGAAAC
GCCATACGAGTCGACAACCAGGCCCTGTACAGCGAGCTCTACAGCAGATACGAATTCCCAGAAACTATTG
CTCATTTTGTCTTCAGTCCCTGCGATCTGCCATGTAATCGTAACGACGCTGTCGCGCCTTATAAAGATAG
AATCAGAAGCCACGCCCCTTCCCCGAACCCTAACAGAAGTCTACGCCCACTACTGCTGGCCACACCTCTC
CAAATCAGACACAGCGAGCGGCATCAGGAAATCATTGAACACTTTGGGTCGTCTTGCTTTTTATAGCCTC
TTACGGCGGCGATATACATTTGTCGAGAGCGAATTGCGAACTTACGGTGTTGACGTACCGCCTCAGGTCG
GCACTCTCAGCTACCGCATTCTCCAGCGCAAGCAGAGCTGGTCATCAGATAACAAGGCGTGGCAGTTCAC
CCACACATCCGTGCAGGAGTTCCTGGGCGCTCTCTATTACTACGTGTCTTCAAGGCGCGCCGTGTTTGAT
TTGTTTTCCGAAAGTGGCGTTTCCTGGCCCCGCATTGGCTTTCACAGTCACTACCGTGCAGCGCTGCAGA
AAACCAACCAATCAGACGCCACATTACAAGGCAGCCTGGACCTCTTCATGCGCTTCCTCTCAGGTCTACT
GTCGCCCACAGCTGGAGTTTTGCTTGGCGGTCCGCTGGGTGTTTCCCGCGAGGAACAGGTGACCCAGCGT
ACCACTGCTATGACTTTGCTTCAGAATGCAGTGGCGGCAGCAGGAGGCGATGCTGTAAGCATGCGCAGCG
TTAATATGGTGACGTGTCTGGCGGAGCTGCAGCAAGGGGAGTGGCTGAGATCCATAGAGGAGGATCTGAT

CAGCTGTGGGCTGCGGGGGAAGCTGAAGGGCGGAGTCTGTGCTGTTCTCGCCTATTTGTTGCTGGTTTCA
GACTCGTGTACTGAAGAGACGCAGCTGTCCAACTGCCTGGACTCCTCCTCCCTCAAGAGGCTCCTCCCAC
AGTTGCTGTACTGCAGTAAGCTGAGAATGGAAAACAATGAATTTAAGGAGGGTGCCATGGAGCTGTTGGG
AAGTCTGCTGAGTGCTAAAGAGTGCCATATTCAGATGTTAAGTCTGGCTGATAACTCAATAAGCAGTAAA
GGAGTTAAACCACTCAGTCGGGCTCTCCTCGTCAACCGAACCCTCACCACACTTGACCTCCGTGGCAACA
ATATTGGTGCCAAGGGAGCAAAGACGTTGTGTGAGGCCCTTAAATTGAACCAGTTCCTTGTGTCAGTCAA
TTTGCAGAATAACCACATTGAGGATGAAGGTGCTCGTTCTCTCTCTGAAGTCCTTCAATCAAACCGAAAG
CTCACCACACTCAACGTTCAGAAAAACGGCATCGGACCAGAAGGCATGAAAAAAATCGCAGAAGGCCTCA
TAAAGAATCAAACTCTACAGGATTTCAATGTATCCAGCAATCATCTGGGTGATTTGGGAACAGTGGCTCT
GGCTCAGGCTCTTATGGTCAATCACGTTCTACACACACTCAGTCTACAGAGTAACTCAGTGAGCGACAGA
GGCATCAAAGCTCTCTCACATGCTCTGCAGTCCAACCGAGGACTCTGCTGTCTGAATTTAAGGGAGAACT
CAATAGGGGTTGCTGGTGCCAAAGACATCGCCAAAGCCTTAAAAGTCAACACTTGCCTACGAGAACTAGA
TCTGACTGCTAATCTCCTTCATGATGAGGGAGTGACGGCCATCGCTGCAGTTTCTGGACGGAGATGCCTG
GAGGAGTCGATGGGTTAATTCGGAGCACAAATCTGACTACGGGCAGTTTAAACTGACCGCTGGAAACTTC
TACGGAGACGCTGAGAAAGATAAGGGCCTGCAGACCAGTCAAGATGCTCGTTTCTACGCCACCTCTGTCC
GCTTCGAGCCCTTTAGCAATGAGGGCAAAACACTGGTGATCCAGTTTACGGTTAAACACGAGCAGAAGAT
CGACTGTGGAGGCGGATACGTTAAAGTCTTCCCAGCTGATCTCAACCAGGCGGACATGCACGGCGACTCC
CAGTATTACATCATGTTTGGGCCTGATATCTGTGGCTACAGCACTAAAAAGGTTCACGTCATTTTTAACT
ATAAGGGACAGAACCACCTCATCAAGAAAGATATCAAATGCAAGGATGATGAACTGACTCACCTGTACAC
ATTGATCTTGAATCCGGATCAGACATATGAGGTTAAGATTGATAATGAGAAGGTGGAGTCCGGCTCTCTG
GAGGAGGACTGGGATTTCTTGCCCCCGAAGAAGATCAAGGACCCCGAAGCTAAAAAACCCGAGGACTGGG
ACGACCGGGCCAAGATTGATGACGAGACTGACACCAAACCTGAGGACTGGGATAAGCCCGAGAATATCCC
CGATCCTGATGCTAAGAAGCCAGAAGACTGGGATGAGGACATGGATGGAGAGTGGGAGCCTGCCATGATC
CCTAACCCCGAGTACAAGGGTGAGTGGAAACCCAAACAGATTGACAACCCGAACTACAAGGGCGCCTGGG
TGCATCCTGAAATCGATAACCCAGAACACGTTCCTGATGACCAGATCTACAAATTCGACAATATTGGAGT
CCTTGGGCTGGATCTCTGGCAGGTGAAGTCTGGCACCATTTTTGACAACTTCTTCATCTCAGATGATGTG
AAAGAAGCTGAGGAATTTGGAAATGAAACGTGGGGCGCTACAAAGGGCCCCGAGAAGAAAATGAAGGAGG
AACAGGAAGAGAAGAAACGCAAAGAGGAAGAGGAAAAGAACAAGGAGCAGAACACCGAAGCAGCAGATGA
GGAAGAGGAAGACGAAGGAGAGGAGGAAGAGGAAGAAGACGAGACTGAAGAGCCTCAGGAGGACGAAGGA
GACGAGGATGTGCTTCAGAAAGACGAGAACCCAAAGCAGAAGATTCTTGTCATGGAGTATTGCTCAGGTG
GAAGTTTACTGAACATGCTGGAGCAGCCTGAGCATGCGTTTGGACTTCCTGAGTCCGAGTTCCTCATTGT
ATTGCATTGTGTGGCTCATGGAATGAACCACCTCCGGGAAAATAGTGTGGTGCATAGGGACATCAAACCT
GGGAATATCATGAGGCAAGTGGGTGAGGACGGGCGCTCCGTGTACAAGCTAGCAGACTTTGGTGCCGCTC
GTGAGTTGGAAGATGATGAGAAGTTTGTGTCCATATATGGCACAGAGGAATATCTGCATCCGCACATGTA
CGAGCGAGCTGTGCTGCGTAGTCCCCAGCAGAAAGCATATGGCGTGTCTGTGGATCTGTGGAGCATTGGT
GTCACCATTTACCACATGGCTACTGGTAGTCTTCCATTTAGACCATTTGGAGGACCGCGCAAAAACAAGC
AGATGATGCACAAGATCACTACAGAAAAACCAGCAGGAACAATAGCTGGTGTACAGAAGGAGGAAGACGG
ACCCATTGAATGGAGAGACAAACTGCCACTCTCCTGCCAACTATCAGAAGGTTTGAAGACCCAGTTGGTC
CCGGTGTTGGCCAACATACTG
