# Supplementary material for: Genome and Phylogenetic Analysis of Genes Involved in the Immune System of Solea senegalensis – Potential Applications in Aquaculture
Source: Front Genet. 2019 Jun 11;10:529. doi: 10.3389/fgene.2019.00529 (PMC6579814; doi:10.3389/fgene.2019.00529)
Supplement: DATA SHEET S2 — Saturation indices and plots with transitions (s) and transversions (v) for each gene and in the concatenated sequence. [file Data_Sheet_2.pdf]

<u>calr</u>

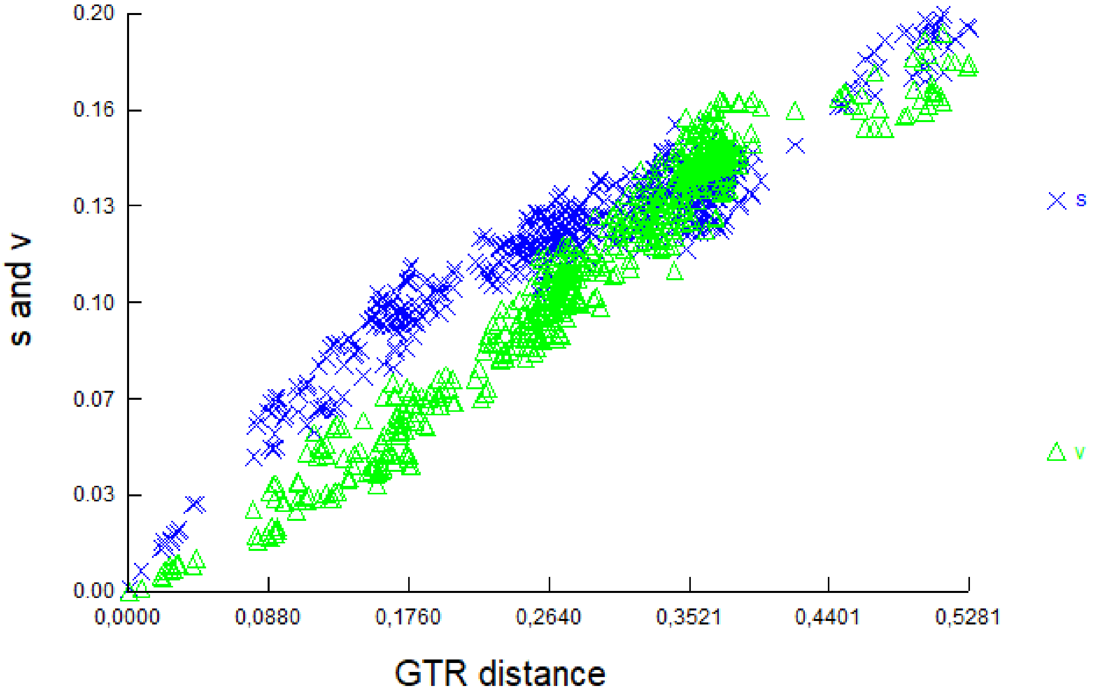

OTU number	Iss	Iss.c	P
4	0,405	0,829	0,0000
8	0,431	0,801	0,0000
16	0,465	0,785	0,0000
32	0,503	0,765	0,0000

<u>ikbke</u>

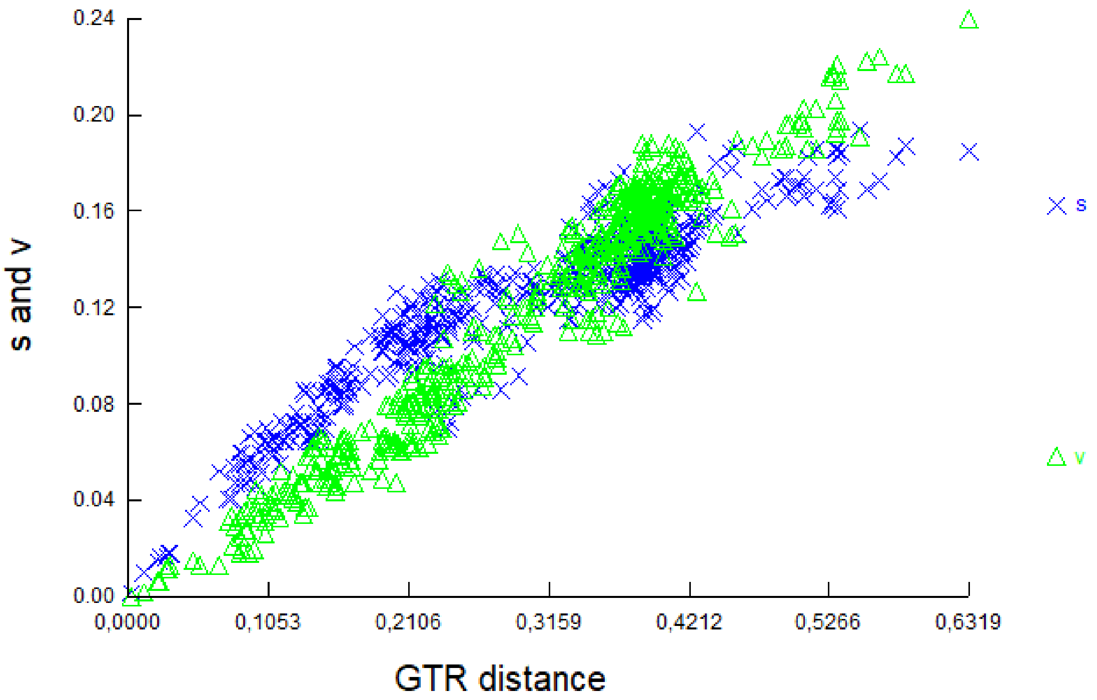

OTU number	Iss	Iss.c	P
4	0,397	0,806	0,0000
8	0,412	0,767	0,0000
16	0,442	0,746	0,0000
32	0,481	0,720	0,0000

nlrc3

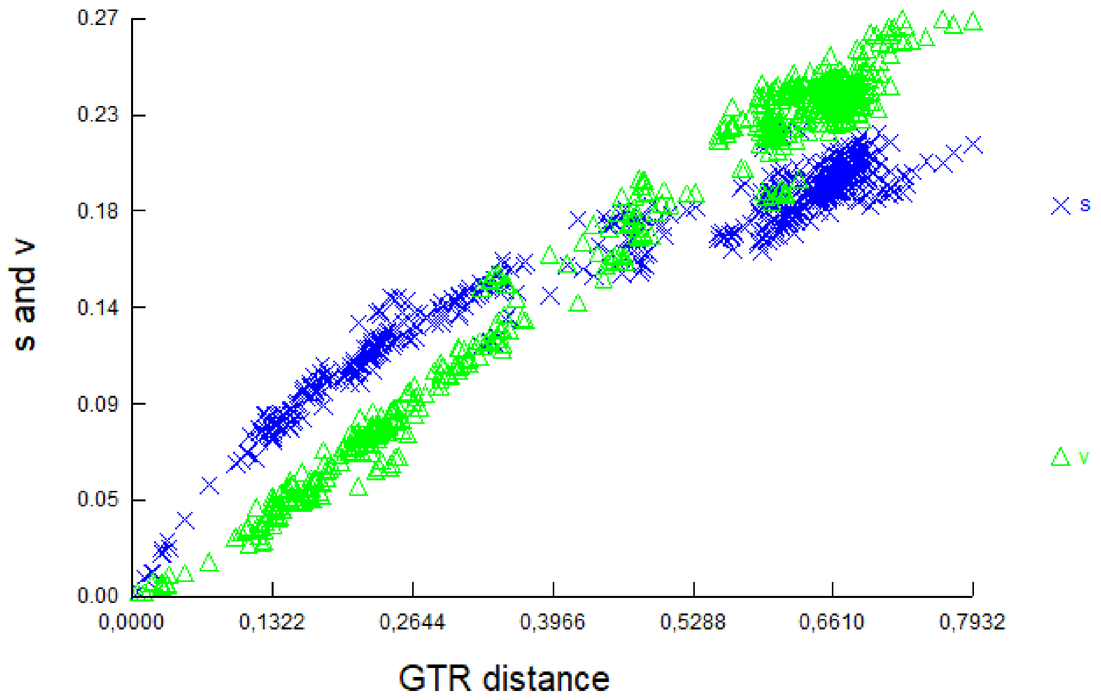

OTU number	Iss	Iss.c	P
4	0,455	0,837	0,0000
8	0,481	0,817	0,0000
16	0,494	0,799	0,0000
32	0,528	0,783	0,0000

<u>tlr3</u>

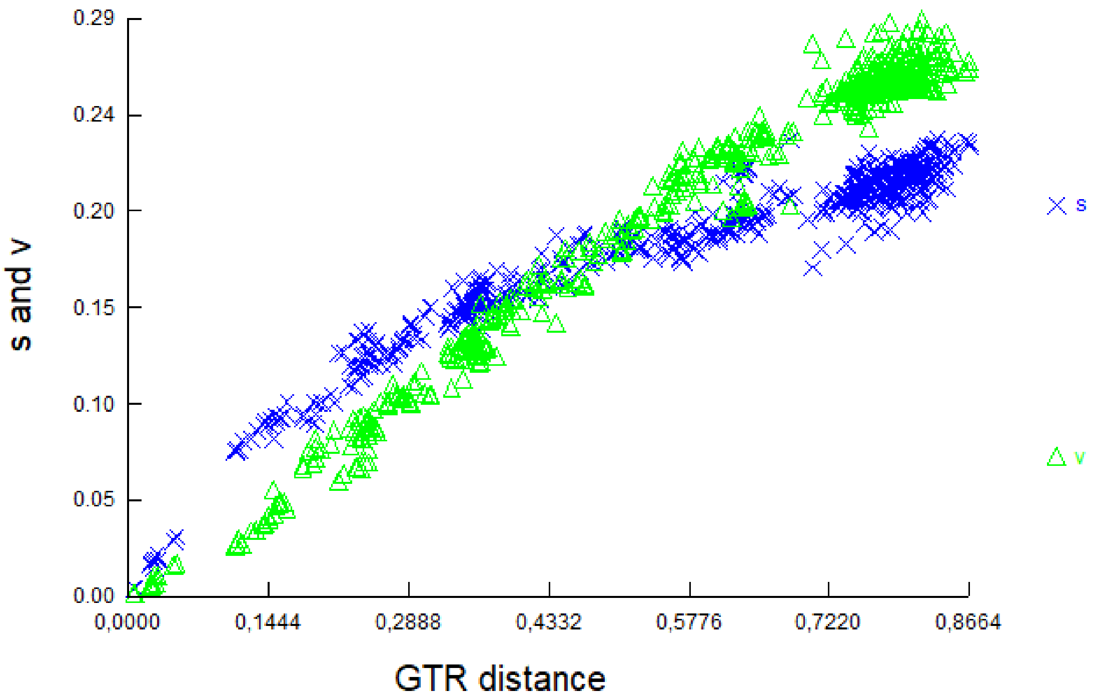

OTU number	Iss	Iss.c	P
4	0,490	0,844	0,0000
8	0,514	0,828	0,0000
16	0,520	0,813	0,0000
32	0,540	0,797	0,0000

<u>tlr8</u>

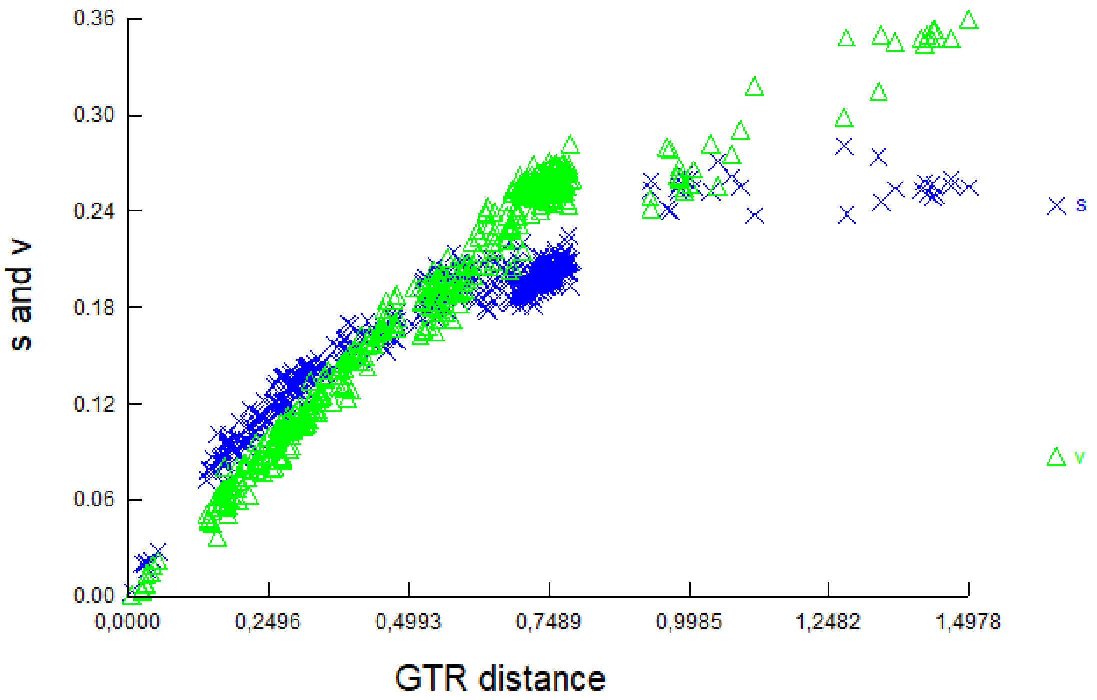

OTU number	Iss	Iss.c	P
4	0,493	0,837	0,0000
8	0,530	0,816	0,0000
16	0,529	0,798	0,0000
32	0,555	0,782	0,0000

tlr3-tlr8-nlrc3-calr-ikbke

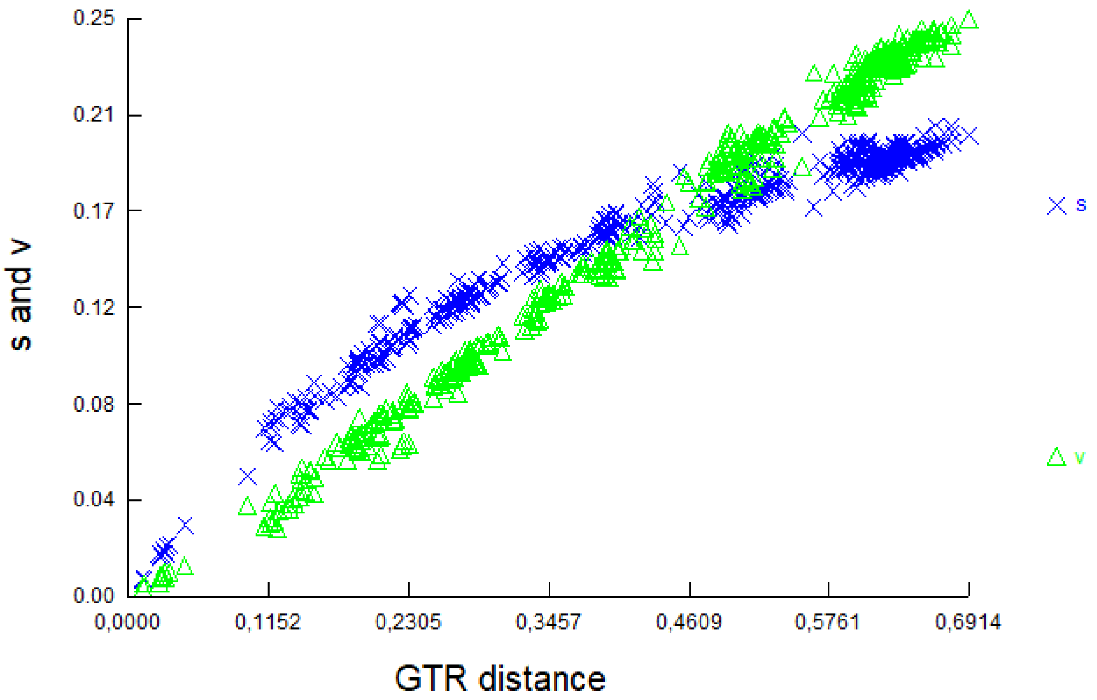

OTU number	Iss	Iss.c	P
4	0,463	0,854	0,0000
8	0,475	0,846	0,0000
16	0,499	0,843	0,0000
32	0,526	0,815	0,0000
